# From Molecular Interactions to Nanocarrier Design: Coarse-Grained Modeling of PEG Self-Assembly and Hindsiilactone Encapsulation

**DOI:** 10.1021/acs.jpcb.5c06860

**Published:** 2026-01-02

**Authors:** Thi H. Ho, Hien Duy Tong, Øivind Wilhelmsen, Thuat T. Trinh

**Affiliations:** † Laboratory for Computational Physics, Institute for Computational Science and Artificial Intelligence, 586268 Van Lang University, Ho Chi Minh City 70000, Vietnam; ‡ Faculty of Mechanical, Electrical, and Computer Engineering, Van Lang School of Technology, 586268 Van Lang University, Ho Chi Minh City 70000, Vietnam; § Faculty of Engineering, Vietnamese-German University (VGU), Thu Dau Mot City, Binh Duong Province 75000, Vietnam; ∥ Porelab, Department of Chemistry, Norwegian University of Science and Technology, Trondheim 7491, Norway

## Abstract

Polyethylene glycol (PEG)-based nanocarriers represent a promising strategy for delivering hydrophobic natural products like Hindsiilactone A (HINA), yet the molecular mechanisms governing polymer–drug interactions and optimal formulation parameters remain poorly understood. This study establishes quantitative design principles for PEG-based nanocarriers through a systematic molecular dynamics investigation of concentration-dependent assembly mechanisms. Martini 3 coarse-grained models were validated against all-atom simulations, achieving structural accuracy while enabling large-scale nanocarrier studies. Comprehensive analysis of PEG–HINA systems reveals distinct mechanistic regimes: optimal encapsulation efficiency (70–76%) occurs at low concentrations with compact particle sizes (8.4–9.8 nm). High HINA concentrations result in a compromised encapsulation efficiency (28.9–58.3%) and increased size heterogeneity (11.8–16.0 nm). The study identifies optimal PEG/HINA ratios (4:1–2:1), concentration windows for reproducible synthesis, and size-loading relationships for therapeutic targeting. This validated computational framework enables the systematic exploration of natural product nanocarriers, providing rational design strategies for sustainable drug delivery system development and clinical translation of bioactive compounds.

## Graphical abstract

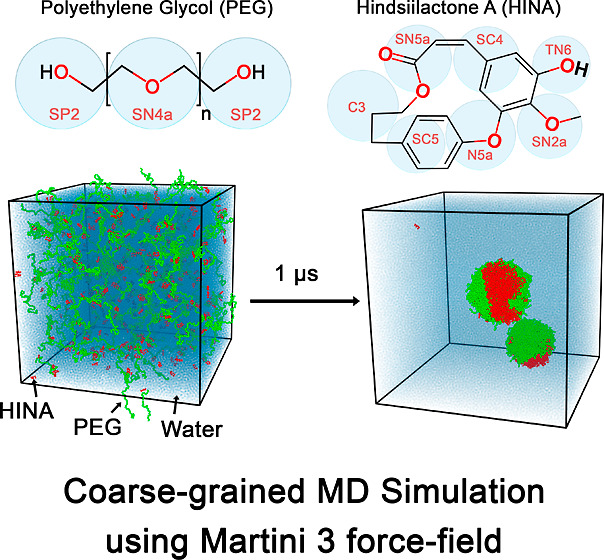

## Introduction

Polyethylene glycol (PEG) is a biocompatible, hydrophilic polymer that has been widely used in drug delivery applications for decades due to its favorable physicochemical properties.^1–3^ By forming a highly hydrated and flexible shell around therapeutic agents, PEG reduces immune recognition and slows systemic clearance, thereby enhancing circulation time.^4^ PEG coatings also protect drug molecules from phagocytic uptake and enzymatic degradation, contributing to improving in vivo stability. One of the most effective applications of PEG is PEGylation, known as the covalent attachment of PEG chains to drugs or nanocarriers, which substantially enhances pharmacological profiles.^5^ Particularly, PEGylation improves aqueous solubility, increases molecular stability, prolongs blood half-life, and reduces immunogenicity, often resulting in less frequent dosing requirements.^6–9^ Consequently, PEGylation significantly improves the pharmacokinetics and biodistribution of therapeutic agents compared to those of their non-PEGylated counterparts.

Beyond simple PEG homopolymers, amphiphilic block copolymers incorporating PEG blocks have become key components of modern biomaterials and drug-delivery platforms, enabling micelles, nanoparticles, and hydrogel scaffolds with tunable mechanical properties, high biocompatibility, and stealth behavior in vivo.^10–12^ Recently, PEG has been widely used as a cosolvent and macromolecular crowder to modulate protein stability. PEG molecular weight and chemistry govern whether PEG stabilizes proteins primarily enthalpically or entropically, and PEG-induced crowding suppresses local breathing motions, thereby altering unfolding pathways and aggregation propensity.^13^ Together, these studies underscore the central role of PEG-based architectures in biomaterials and protein formulations and provide a broader context for the PEG-based nanocarrier design principles developed for hydrophobic small-molecule encapsulation.

Hindsiilactone A (HINA) is a bioactive natural product isolated from *Celastrus hindsii*, a medicinal plant native to Southeast Asia that has been traditionally used for its antioxidant and anti-inflammatory properties.^14–16^ HINA represents one of the promising secondary metabolites derived from this plant, exhibiting significant therapeutic potential with demonstrated anticancer and antioxidant activities.^17–19^ HINA was first characterized by Hu et al. as a new macrocyclic lactone isolated from the 80% ethanol extract of *C. hindsii* stems, together with combretastatin derivatives and other phenolic constituents, and has since been listed among the characteristic secondary metabolites of the species in phytochemical fields.^20,21^ In vitro studies on *C. hindsii* extracts and isolated compounds indicate that HINA contributes to the cytotoxic and antioxidant profile of the plant, with modest antiproliferative effects against several human tumor cell lines (e.g., NCI-H187, HCT116, BC-1, and HuH7) and strong radical-scavenging activity in standard assays. Taken together, these data support HINA as a structurally complex, hydrophobic natural product with promising anticancer and antioxidant potential but also one whose physicochemical properties motivate the development of tailored nanocarrier formulations such as PEG-based assemblies.

Despite their promising biological activity, the therapeutic application of these natural products is frequently limited by poor aqueous solubility, low bioavailability, and inherent chemical instability. Many secondary metabolites from *C. hindsii* are hydrophobic or chemically labile, presenting significant barriers to formulation and effective drug delivery.^22^ To overcome these limitations, PEG-based delivery strategies have emerged as a promising approach.^14^ PEGylation or PEG-encapsulation can significantly enhance the solubility and protect labile molecules from degradation in vivo. Coating drug molecules with PEG forms a hydrophilic barrier that not only improves solubility but also reduces enzymatic degradation and immune system clearance.^22^ Nevertheless, the interaction between PEG and drug molecules can be highly variable—some compounds may exhibit weak binding affinity to PEG, thereby reducing delivery efficiency. Gaining molecular-level insight into these interactions is, therefore, crucial for the rational design of PEGylated drug formulations with optimal performance.

Computational modeling has become an essential tool for investigating polymer–drug systems.^14,23–28^ All-atom molecular dynamics (MD) simulations have provided valuable insights into the behavior of PEG and PEGylated constructs, including chain conformations and the formation of drug delivery systems.^14,25–28^ However, the computational cost of simulating large PEGylated assemblies at an atomic resolution remains a significant limitation. To address this, the Martini coarse-grained (CG) force field offers an efficient and scalable alternative. By grouping atoms into interaction sites or “beads”, the Martini model reduces system complexity and enables simulations that are orders of magnitude faster than atomistic approaches.^29^ Now in its third generation, the Martini 3 force field provides improved accuracy, refined interaction parameters, and expanded chemical space coverage.^30^ Its modular, building-block approach—paired with user-friendly, open-source tools—facilitates the mapping of all-atom structures (such as those derived from AMBER force fields) to CG topologies. This framework enables the exploration of large-scale polymeric assemblies and drug delivery systems on microsecond time scales, which would otherwise be computationally infeasible at full atomic detail. Recently, a residue-based PEG model compatible with the CALVADOS protein force field has also been proposed.^31^ This model uses implicit-solvent, one-bead-per-monomer mapping to describe PEG–protein crowding and phase separation, offering an efficient CG description that captures PEG–protein crowding, conformational compaction, and liquid–liquid phase separation within a unified framework. In the present work, however, our focus on PEG self-assembly and hydrophobic small-molecule encapsulation in water is more naturally addressed within the explicit-solvent Martini 3 framework, which is specifically optimized for polymer solution thermodynamics and self-assembly.

This work employs a systematic multiscale computational approach combining atomistic and CG MD simulations to elucidate the concentration-dependent assembly mechanisms of PEG2000-HINA nanocarriers. Atomistic models developed using the AMBER force field provide detailed molecular interactions, which are subsequently mapped to validated Martini 3 CG representations, enabling microsecond-scale simulations of large polymeric assemblies. Through a comprehensive analysis of structural properties, encapsulation efficiency, and assembly kinetics across varying drug loadings, this study establishes quantitative design principles for optimizing nanocarrier performance. The study reveals critical concentration thresholds, optimal polymer-to-drug ratios, and mechanistic insights into the governing particle size evolution and encapsulation stability. These findings provide a computational framework for rational formulation design, contributing fundamental knowledge for developing effective delivery systems for hydrophobic natural products, and advancing sustainable therapeutic strategies.

## Methods

### Simulation Details

#### All-Atom MD Calculations

All-atom MD simulations were performed using the GROMACS software^32^ to obtain the benchmarking reference for the CG MD simulations. The molecular interactions were described using the GAFF2 force field,^33,34^ which parametrized by the ACPYPE Python package.^35^ The leapfrog integrator was used with a time step of 2 fs to numerically solve Newton’s equations of motion. A total of 5,000,000 time steps were conducted, corresponding to a 10 ns simulation duration. LINCS constraints were applied to all bonds involving hydrogen atoms by using a fourth-order expansion. To maintain translational invariance and avoid center-of-mass drift, the linear center-of-mass motion was removed every 25 steps. Nonbonded interactions were managed using a neighbor list updated every 20 steps with a cutoff radius of 0.8 nm. Electrostatic interactions were computed by using the reaction-field method with a dielectric constant of 80 to mimic the properties of water. The real-space cutoff for Coulomb–Lennard–Jones interactions was set to 1.4 nm. Long-range dispersion corrections for the energy and pressure were applied. Temperature was maintained at 298.15 K using the Nosé–Hoover thermostat, and the pressure was regulated at 1.0 bar using the Parrinello–Rahman barostat with isotropic pressure coupling of 5 ps and a compressibility of 5 × 10^–5^ bar^–1^.

#### CG MD Calculations

CG MD simulations were performed using the GROMACS simulation package^32^ in conjunction with the Martini 3 force field^36^ to investigate the self-assembly and encapsulation behavior of PEG and HINA in aqueous environments. Simulations were carried out for a total of 1 μs. A time step of 30 fs was used for PEG-only systems, while a reduced step of 20 fs was applied to PEG–HINA systems to ensure stability in the presence of more complex polymer–drug interactions.

Nonbonded interactions were handled using the Verlet cutoff scheme, with neighbor lists updated every 40 steps. Electrostatic and van der Waals interactions were computed using the potential-shift-Verlet modifier with a uniform cutoff radius of 1.1 nm. Temperature control was achieved using the velocity-rescale thermostat set to 298 K with a coupling constant of 1.0 ps. Pressure was maintained at 1 bar using the Parrinello–Rahman barostat with isotropic coupling, a time constant of 12.0 ps, and a compressibility of 3 × 10^–4^ bar^–1^.

Each system underwent energy minimization and equilibration prior to production runs to remove steric clashes, achieve proper solvation, and establish a realistic initial density. This preparation ensured a homogeneous molecular distribution and thermodynamic stability at the start of the simulations, allowing for accurate monitoring of self-assembly and encapsulation processes over time.

#### Mapping Schemes Using the Martini 3 Force Field

Figure 1a illustrates the CG mapping of PEG, where each ethylene glycol repeat unit is represented by two Martini 3 bead types: SN4a for the backbone segment and SP2 for the terminal hydroxyl groups. This dual-bead mapping preserves both the backbone flexibility and the terminal polarity that is essential for realistic polymer behavior. It aligns well with previously validated Martini-based models of PEG and poly­(ethylene oxide) (PEO) that successfully match atomistic conformational distributions and thermodynamic properties across a range of molecular weights and concentrations.^37^

**Figure 1:**
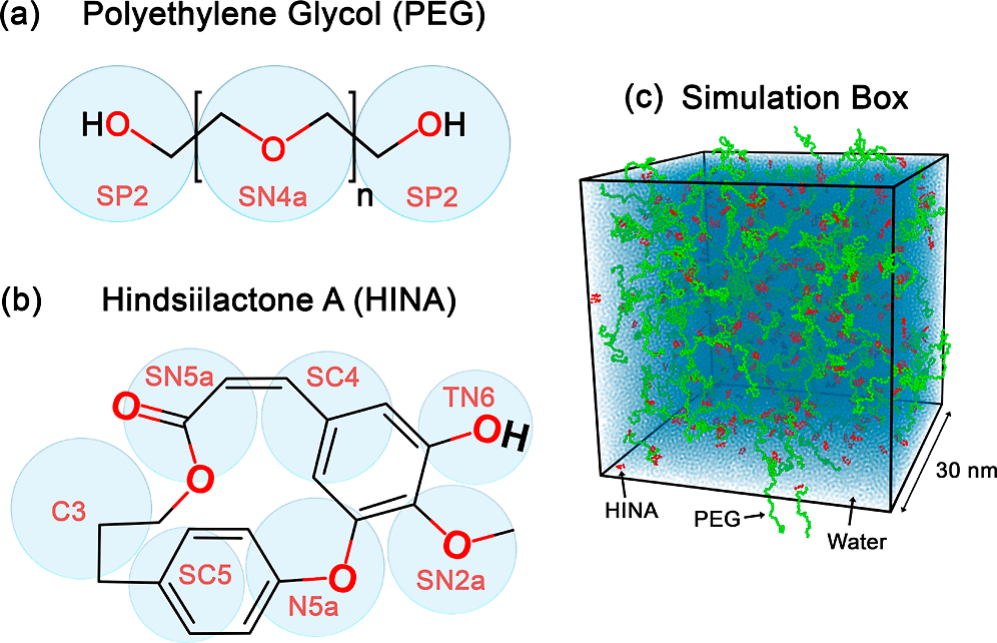
(a,b) Chemical structures and CG representations using the Martini 3 force field for PEG and HINA, respectively. (c) Cubic unit cell in MD simulation, showing the spatial distribution of PEG chains (green), HINA molecules (red), and water (light blue) under the CG model at *t* = 0 μs.

Figure 1b illustrates the CG mapping scheme developed for HINA. Guided by the compound’s chemical structure and functional group properties, the molecule was represented using Martini 3 bead types, including SN2a, TN6, SC4, SC5, SN5a, and N5a. Each bead was carefully chosen to reflect the local polarity, hydrogen bonding propensity, and the surrounding chemical environment. Aromatic and aliphatic ring systems were modeled by using appropriate bead types and constraints to preserve their planarity and rigidity. Oxygen-containing functional groups were assigned to polar beads to retain the solvation behavior and facilitate intermolecular interactions. The overall structure was treated as rigid, with high bond and angle force constants applied to maintain geometric integrity during simulation. This mapping approach ensures a balance between chemical accuracy and computational efficiency, capturing the essential behavior of HINA in the CG MD simulations.

In addition, water molecules were represented using the standard Martini water model,^38^ where each CG water bead corresponds to approximately four real water molecules. This representation effectively captures essential solvation and hydrophobic behavior relevant to PEG–HINA assembly while maintaining computational efficiency.

Figure 1c shows the initial configuration of the simulation system at time *t* = 0 μs. In this setup, PEG chains (green), HINA molecules (red), and water beads (light blue) are uniformly distributed within a 30 nm cubic simulation box. Systems at various concentrations of PEG and HINA were systematically constructed using GROMACS tools with CG topologies derived from all-atom structures via a validated mapping scheme. The detailed compositions of the simulated systems, including the number of PEG chains, HINA molecules, PEG/HINA ratios, water beads for solvation, and the resulting system densities, are summarized in Table 1.

**Table 1:** CG-Mapping of the PEG Molecule Using Martini 3^a^

system	PEG chains	HINA molecules	PEG/HINA	water beads	density (kg/m^3^)
PEG-only	50	0	-	230,293	992.99 ± 0.02
PEG-only	100	0	-	229,602	996.09 ± 0.03
PEG-only	200	0	-	228,117	1002.24 ± 0.05
PEG-only	300	0	-	226,545	1008.25 ± 0.07
PEG-only	400	0	-	225,036	1014.36 ± 0.08
PEG-only	500	0	-	223,626	1020.57 ± 0.09
PEG-only	600	0	-	222,063	1026.27 ± 0.11
PEG-only	700	0	-	220,562	1032.20 ± 0.11
PEG-only	800	0	-	219,163	1038.06 ± 0.09
PEG-only	900	0	-	217,682	1043.83 ± 0.13
PEG-only	1000	0	-	216,011	1050.08 ± 0.11
PEG-only	1100	0	-	214,630	1055.50 ± 0.11
PEG-only	1200	0	-	213,173	1061.14 ± 0.10
PEG + HINA	200	50	4:1	227,952	1002.14 ± 0.06
PEG + HINA	200	100	2:1	227,869	1002.61 ± 0.05
PEG + HINA	200	200	1:1	227,626	1003.78 ± 0.04
PEG + HINA	200	300	2:3	227,454	1004.68 ± 0.04
PEG + HINA	200	400	1:2	227,178	1005.34 ± 0.05
PEG + HINA	200	500	2:5	226,979	1006.24 ± 0.04
PEG + HINA	200	600	1:3	226,904	1007.36 ± 0.05
PEG + HINA	200	700	2:7	226,665	1008.05 ± 0.04
PEG + HINA	200	800	1:4	226,413	1008.97 ± 0.04
PEG + HINA	200	900	2:9	226,156	1009.31 ± 0.07
PEG + HINA	200	1000	1:5	225,991	1010.81 ± 0.04
PEG + HINA	200	1100	2:11	225,862	1011.69 ± 0.04
PEG + HINA	200	1200	1:6	225,602	1012.81 ± 0.04
PEG + HINA	200	1300	2:13	225,313	1013.51 ± 0.04
PEG + HINA	200	1400	1:7	225,097	1014.43 ± 0.04
PEG + HINA	200	1500	2:15	224,926	1015.62 ± 0.04
PEG + HINA	200	1600	1:8	224,696	1016.21 ± 0.04
PEG + HINA	200	1700	2:17	224,453	1017.13 ± 0.04
PEG + HINA	200	1800	1:9	224,256	1018.25 ± 0.03
PEG + HINA	200	1900	2:19	224,274	1018.90 ± 0.05
PEG + HINA	200	2000	1:10	223,902	1019.84 ± 0.04

a The table shows the equivalent CG beads, CG atom types, and corresponding mass distribution for each segment of the molecule.

### Analysis Details

All trajectory analyses were performed using built-in tools provided by GROMACS and the visual MD software package.^39^ Structural and dynamic properties, such as radial distribution functions (RDF) *g*(*r*), solvent-accessible surface area (SASA), and radius of gyration (*R*_g_), were computed by using standard analysis modules. Molecular visualization and rendering were carried out using OVITO.^40^

The density of each system was computed by averaging the instantaneous densities over selected segments of the trajectory obtained from *NPT* simulations. The instantaneous density ρ at a given time point was calculated as follows

1 $$\rho =\frac{M}{V}$$

where *M* is the total mass of the system and *V* is the instantaneous volume of the simulation box at that time. Then, the average density ⟨ρ⟩ was calculated by averaging the instantaneous density values.

The RDF, also known as the pair correlation function, was calculated to quantify the spatial distribution of particle B around a reference particle A, which is defined as

2 $$g_{AB}\left(r\right)=\frac{\left\langle \rho_{B}\left(r\right)\right\rangle }{{\left\langle \rho_{B}\right\rangle }_{local}}$$

3 $$=\frac{1}{4\pi r^{2}{\left\langle \rho_{B}\right\rangle }_{local}N_{A}}\sum_{i\in A}^{N_{A}}\sum_{\begin{array}{l} j\in B\\ j\neq i \end{array}}^{N_{B}}\delta \left(r-r_{ij}\right)$$

where ⟨ρ_B_(*r*)⟩ is the average local density of particle B at a distance *r* from a reference particle A, and ${\left\langle \rho_{B}\right\rangle }_{local}$ represents the average density of B over the system, estimated within spheres centered on A with radii up to half the box length. *N*_A_ and *N*_B_ are the number of reference particles of type A and B, and *r*_*ij*_ is the distance between particles *i* and *j*. In this work, RDFs were computed using the standard GROMACS module *gmx rdf*. This method provides insights into the degree of structural ordering and preferential interactions in PEG–HINA assemblies during the MD simulation.

SASA quantifies the extent of a molecule surface that is exposed to the solvent and serves as a useful indicator of the encapsulation efficiency and structural compaction during self-assembly. SASA is determined by rolling a spherical probe—typically with a radius of 1.4 Å, approximating the size of a water molecule—over the van der Waals surface of the molecule. The total SASA is calculated as

4 $$SASA=\sum_{i=1}^{N}A_{i}\cdot S_{i}$$

where *A*_*i*_ represents the surface area contribution of atom *i*, and *s*_*i*_ is a binary indicator denoting whether the surface patch is accessible to the solvent probe. The summation is carried out over all atoms in the selected group. In this work, SASA was computed frame-by-frame across the simulation trajectory for PEG–HINA. The resulting values were monitored to track the solvent exposure trends and encapsulation behavior across different concentrations.

To gain insight into the compactness of the PEG and PEG–HINA structures, the radius of gyration (*R*_g_) was computed as follows

5 $$R_{g}=\sqrt{\frac{\sum_{i=1}^{N}m_{i}|{\mathrm{r⃗}}_{i}-{\mathrm{r⃗}}_{cm}{|}^{2}}{\sum_{i=1}^{N}m_{i}}}$$

where ${\mathrm{r⃗}}_{i}$ is the position vector of atom *i*, ${\mathrm{r⃗}}_{cm}$ is the position of the center of mass of the chain, and *m*_*i*_ is the mass of atom *i*. The radius of gyration was computed and monitored throughout the simulation trajectory.

## Results

### Model Validation

The reliability of CG simulations for investigating complex polymer–drug systems depends critically on an accurate representation of molecular interactions and structural properties. In order to establish confidence in the large-scale assembly studies, we will first validate the Martini 3-based parametrization by comparing it to all-atom reference simulations.

### PEG Molecule

Validation of the CG parametrization by comparison to atomistic reference data reveals that there is excellent agreement for critical structural properties (Figure 2 and Table 2). Bond length distributions exhibit Gaussian profiles centered at 0.30 nm in both approaches, with deviations of only 0.001 nm. The CG representation produces narrower, more symmetric distributions due to the inherent smoothing of high-frequency fluctuations while preserving the essential geometric features.

**Figure 2:**
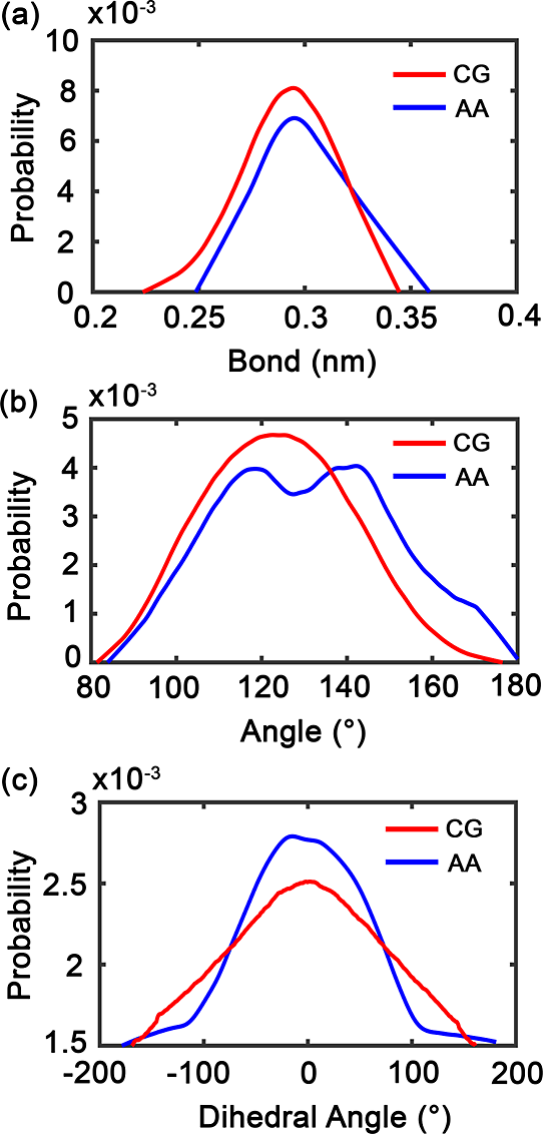
Structural distribution comparison between CG and AA simulations of PEG in water: (a) bond length, (b) torsion angle, and (c) dihedral angle. CG results are shown in red and AA results in blue, illustrating the agreement and deviations between the two models.

**Table 2:** Comparison of Structural and Density Properties from AA and CG PEG Simulations^a^

property	AA	CG	deviation
bond length (nm)	0.295	0.294	0.001
torsion angle (°)	118.32, 142.05	123.94	5.62, 18.11
dihedral angle (°)	–16.32	1.09	17.41
density in water (kg/m^3^)	986.56 ± 0.01	990.04 ± 0.01	3.48

a Deviation for bond, torsion, and dihedral angles is the absolute difference between AA and CG peak positions. Density values are reported as mean ± standard error from the density time series of the trajectory.

Torsional flexibility, a key determinant of polymer conformational behavior, is well-reproduced, with peak positions matching within 5.62–18.11°. The atomistic simulations capture finer conformational substates through multiple peaks between 115° and 140°, whereas the CG approach yields a unified distribution centered at 120°. This averaging behavior is expected and acceptable for capturing ensemble properties relevant to large-scale self-assembly processes.

Dihedral angle distributions show the largest deviation (17.41°), reflecting the challenge of mapping complex rotational degrees of freedom onto simplified interaction sites. However, the CG model successfully reproduces the accessible conformational space, which is sufficient for investigating polymer aggregation phenomena. The density agreement (3.48 kg/m^3^ deviation) validates the thermodynamic consistency of the parametrization.

To make a direct connection to earlier Martini PEG/PEO work, we further validated the structural parametrization for a PEO36 chain, which is the same chain length used in the original Martini PEO study of Lee et al.^41^ In Figure S1 (see Supporting Information), we report the structural distribution of bond length, torsion angle, and dihedral angles for PEO36 in water obtained from all-atom GAFF2 simulations and from the corresponding Martini 3 model. Martini 3 well reproduces the all-atom structure. These deviations are comparable to those reported for the original Martini PEO36 model^41^ and fall within the typical accuracy expected for Martini-type parametrization, confirming that the present Martini 3 PEG mapping provides a reliable starting point for large-scale self-assembly simulations.

In addition to the structural distributions, we benchmarked the overall chain dimensions of our PEO model against those of earlier Martini simulations and neutron-scattering data. We performed single-chain simulations of PEO with *n* = 18, 36, and 76 repeat units in water at the all-atom (GAFF2) and Martini 3 coarse-gain force fields and computed the corresponding radii of gyration *R*_g_. The results are summarized in Table S1 (see Supporting Information), where they are compared to the original Martini PEO model and CHARMM data of Lee et al.^41^ and, for PEO76, to *R*_g_ obtained from neutron scattering.^42^ For the longest chain (*n* = 76), Martini 3 yields *R*_g_ = 18.6 ± 0.6 Å, compared to 19.1 ± 0.7 Å for the original Martini model and 19.7 Å from experiment.^42^ The GAFF2 value (20.6 ± 0.3 Å) is also consistent with the CHARMM result (20.4 ± 0.8 Å).^41^ Overall, the Martini 3 radii of gyration deviate from experimental and previous Martini values by at most 5–6%, which is within the typical accuracy of Martini-type CG parametrization.

To further assess the dynamical realism of our PEG models, we evaluated the ^1^H NMR relaxation rate *R*_1_ for PEG–water mixtures, a quantity that is sensitive to polymer segmental dynamics and PEG–water interactions. Following the work of Gravelle et al.,^10^ we computed *R*_1_ at PEG concentrations *c*_PEG_ = 0.1, 1, and 10 using both the all-atom GAFF2 and the Martini 3 CG models. The resulting *R*_1_ values are summarized in Table S2 (see Supporting Information), where they are compared to the theoretical AA and CG results of Gravelle et al.^10^ and to experimental data from Jora et al.^43^ Both our AA and CG simulations reproduce the monotonic increase in *R*_1_ with PEG concentration and yield values of the correct order of magnitude, supporting the use of our PEG model for capturing concentration-dependent trends in PEG–water segmental dynamics.

These validation metrics establish that the CG representation captures the essential physics of PEG chains while providing the computational efficiency necessary for studying large-scale assembly processes. The systematic averaging of fine structural details represents an acceptable trade-off for accessing extended length and time scales.

### HINA Molecule

The CG parametrization of HINA demonstrates remarkable fidelity to atomistic reference data across multiple structural metrics (Figure 3 and Table 3). Bond length distributions are reproduced with high precision, achieving perfect agreement for the shortest bonds (0.00 nm deviation) and minimal deviations (0.01 nm) for longer bonds. This accuracy is crucial for preserving the rigid lactone core structure that defines HINA’s molecular identity.

**Figure 3:**
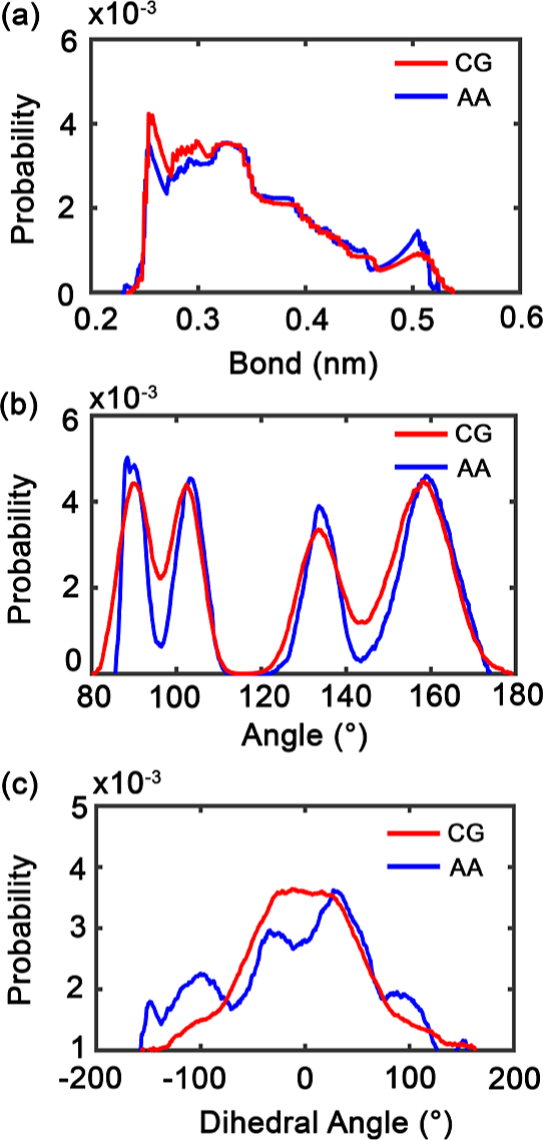
Structural distribution comparison of HINA in water from CG and AA simulations. (a) Bond length, (b) bond angle, and (c) dihedral angle distributions. CG results are shown in red and AA results in blue, highlighting the correspondence and differences between the two models.

**Table 3:** Comparison of Structural and Density Properties from the AA and CG HINA Simulations^a^

property	AA	CG	deviation
bond length (nm)	0.25, 0.29, 0.50	0.25, 0.30, 0.51	0.00, 0.01, 0.01
torsion angle (°)	88.43, 103.32, 133.61, 158.94	89.69, 102.48, 133.45, 158.42	1.26, 0.84, 0.16, 0.52
dihedral angle (°)	–33.57, 26.86	–3.97	29.60, 30.83
density in water (kg/m^3^)	987.72 ± 0.09	993.65 ± 0.05	5.93

a Deviation for bond, torsion, and dihedral angles is the absolute difference between the AA and CG peak positions. Density values are reported as the mean ± standard error from the density time series of the trajectory.

The torsional angle validation reveals outstanding performance as well, with all four major conformational states reproduced within 0.16–1.26° accuracy. This precision stems from the careful mapping of HINA’s complex polycyclic structure onto appropriately chosen interaction sites, maintaining the molecule’s intrinsic conformational preferences. The excellent agreement validates the approach of preserving key pharmacophoric features during the coarse-graining process.

Dihedral angle reproduction presents greater challenges, with deviations reaching 29.60–30.83° for specific rotational states. This reflects the inherent difficulty of mapping complex multiring torsional coupling onto simplified CG sites. However, the overall conformational envelope remains accessible, ensuring that essential drug–polymer interaction geometries are preserved. The density reproduction (5.93 kg/m^3^ deviation) confirms thermodynamic consistency in aqueous environments.

Overall, the excellent agreement gives confidence in the CG HINA model’s ability to capture essential drug properties while enabling computationally efficient exploration of encapsulation mechanisms. The parametrization successfully balances molecular detail with simulation feasibility, providing a reliable foundation for investigating drug–carrier interactions on experimentally relevant scales.

### Structural Properties of PEG Chains in Water at Various Densities

After establishing that the CG models are reliable, we next investigated the concentration-dependent self-assembly behavior of PEG chains in aqueous solution. Understanding these fundamental assembly mechanisms provides the foundation for rational nanocarrier design and optimization of drug encapsulation strategies.

The concentration-dependent structural evolution of PEG assemblies reveals fundamental insights into polymer self-assembly mechanisms (Figure 4). System density increases linearly from 993 to 1061 kg/m^3^ across the concentration range, consistent with experimental PEG2000 data (997–1083 kg/m^3^).^44^ This linear relationship confirms additive mixing behavior and supports our simulation approach for predicting bulk solution properties.

**Figure 4:**
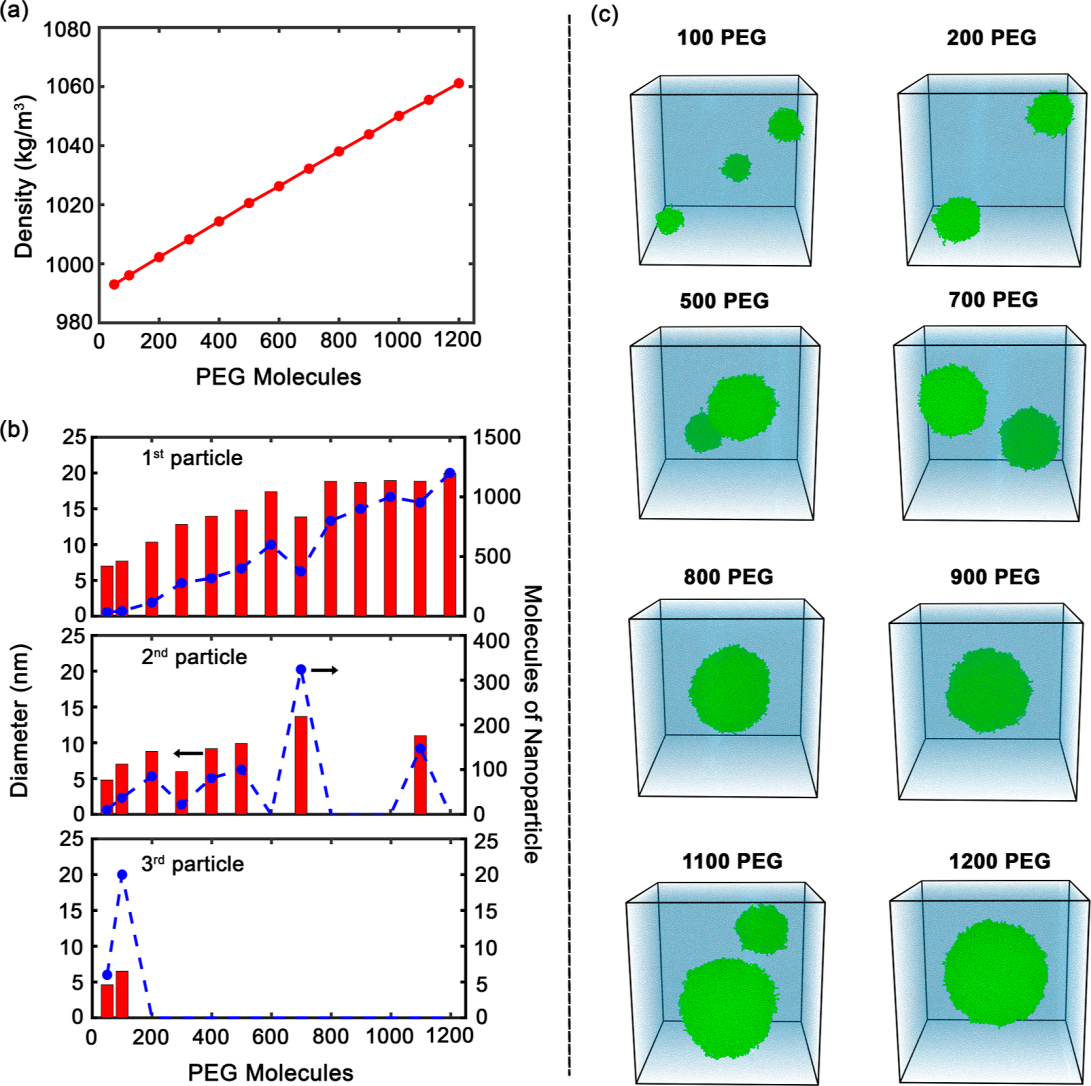
(a) Density as a function of the number of PEG molecules. (b) Size distribution and number of molecules of the three PEG-based nanoparticles formed at different concentrations. Red bars represent the particle diameters, and blue dashed lines indicate the corresponding number of PEG molecules in each nanoparticle. (c) Snapshots at increasing PEG concentrations, demonstrating the aggregation behavior and size evolution of PEG nanoparticles (green) in the water (blue).

The aggregation pathway follows classic nucleation–growth-coalescence kinetics with distinct concentration-dependent regimes. Primary aggregates grow continuously from 4 nm (50 chains) to 20 nm (1200 chains), demonstrating cooperative assembly driven by chain entanglement and excluded volume effects. Secondary aggregates exhibit transient stability, appearing intermittently before they merge with dominant clusters. Tertiary clusters exist only at low concentrations, suggesting a critical concentration threshold for the sustained nucleation. This hierarchical assembly pattern reflects the competition between entropy-driven chain dispersion and enthalpy-favored aggregation, consistent with established polymer physics theory.^45^

Morphological evolution proceeds through three distinct regimes: (i) dilute cluster formation (100–200 chains), (ii) competitive growth phase (500–700 chains), and (iii) coalescence-dominated assembly (>800 chains). This progression reflects the increasing importance of chain–chain interactions relative to chain-solvent interactions, as local PEG density exceeds the overlap concentration. The transition to single-aggregate dominance occurs near 800 molecules, corresponding to a critical volume fraction where intercluster collisions become frequent and thermodynamically favorable.

Assembly kinetics exhibits concentration-dependent acceleration governed by collision frequency and thermodynamic driving forces (Figure 5). At low concentrations (100 chains), nucleation is rate-limiting due to infrequent chain encounters, resulting in multiple small metastable aggregates persisting beyond 1 μs. The system remains kinetically trapped in a local minimum, unable to overcome activation barriers for complete coalescence.

**Figure 5:**
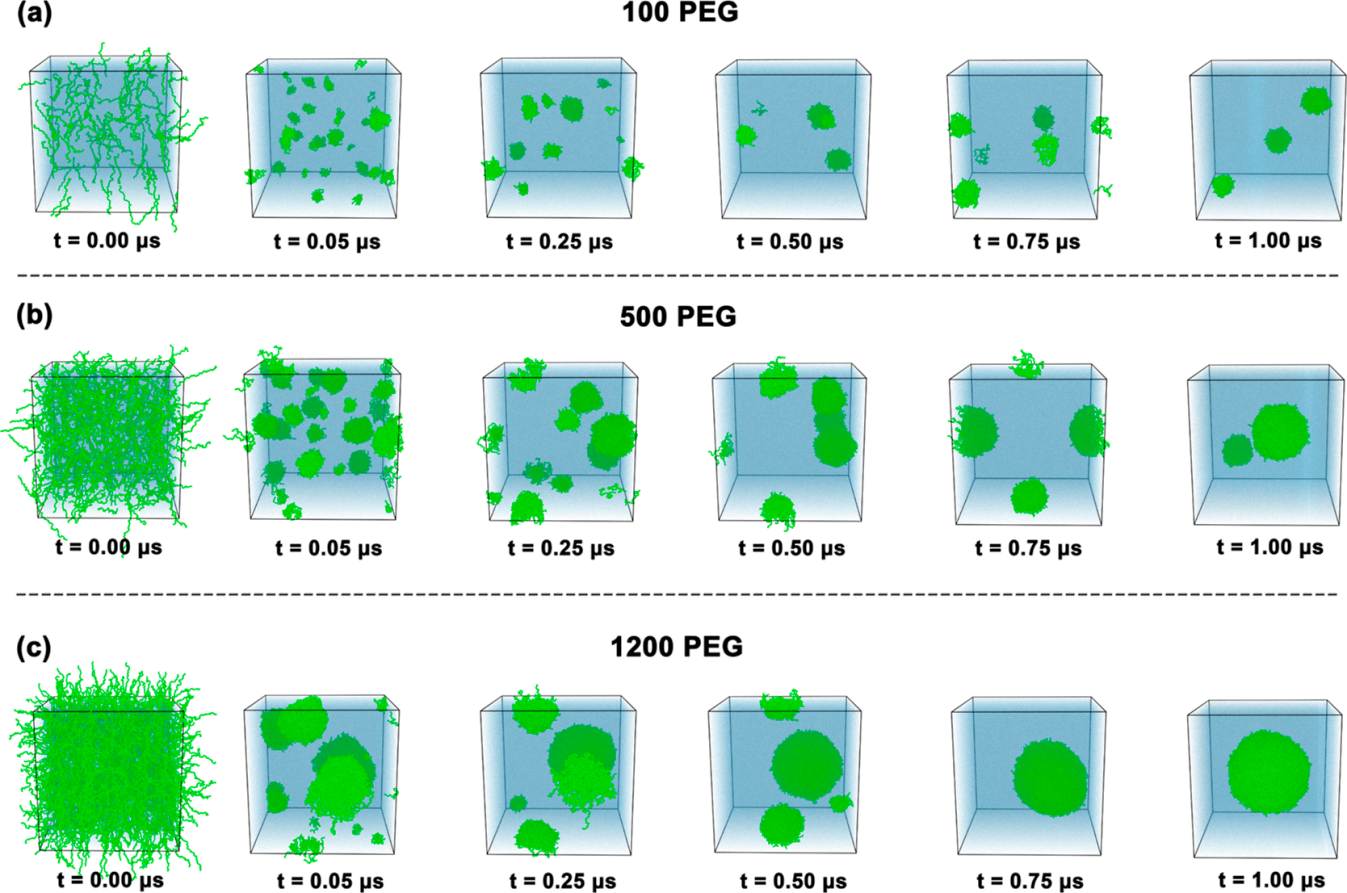
Time evolution of PEG aggregation at different concentrations. Representative MD snapshots at selected time points (0.00–1.00 μs) for systems containing (a) 100, (b) 500, and (c) 1200 PEG molecules. PEG chains (green) gradually self-assemble into nanoparticles over time in water (blue), with higher concentrations leading to faster and larger aggregate formation.

Intermediate concentrations (500 chains) accelerate both nucleation and growth phases through enhanced collision rates. Primary nuclei form within 50 ns and undergo rapid growth through secondary nucleation and Ostwald ripening mechanisms. The final biaggregate state represents a kinetic intermediate approaching thermodynamic equilibrium, where coalescence barriers become comparable to thermal energy.

High concentrations (1200 chains) transition to diffusion-limited aggregation, where instantaneous cluster formation reflects the system’s proximity to the spinodal decomposition regime. Chain entanglement networks span the simulation box from *t* = 0, eliminating nucleation barriers and enabling rapid coalescence. The near-complete assembly within 500 ns demonstrates entropy-driven phase separation characteristic of concentrated polymer solutions above the overlap threshold.

This concentration-dependent transition from nucleation-limited to diffusion-limited kinetics reflects fundamental changes in the aggregation mechanism, with implications for controlling nanocarrier size distributions in experimental formulations. The established assembly pathways provide the framework for understanding how drug incorporation modifies these processes and influences the encapsulation efficiency in PEG-based delivery systems.

Quantitative analysis of the concentration-dependent aggregation reveals systematic trends in particle formation and size distribution (Table 4). The transition from multiple small aggregates at low concentrations to single dominant particles at high concentrations demonstrates the critical role of concentration in controlling the nanostructure formation.

**Table 4:** PEG Concentration–Dependent Aggregation in Water, Showing Particle Numbers, Sizes, Median Diameters, and PEG Distribution Per Aggregate

number of PEG (*n*)	PEG concentration (mM)	number of particles	particle size (nm)	size median (nm)	PEG count (*n*)
50	3.08	3	7.01, 4.80, 4.62	4.80	34, 10, 6
100	6.15	3	7.69, 7.02, 6.52	7.02	43, 37, 20
200	12.30	2	10.34, 8.81	9.58	115, 85
300	18.45	2	12.82, 5.96	9.39	278, 22
400	24.60	2	13.96, 9.19	11.58	319, 81
500	30.75	2	14.81, 9.90	12.36	400, 100
600	36.90	1	17.38	17.38	600
700	43.05	2	13.88, 13.68	13.78	376, 324
800	49.20	1	18.83	18.83	800
900	55.35	1	18.69	18.69	900
1000	61.50	1	18.96	18.96	1000
1100	67.65	2	18.84, 10.97	14.91	953, 147
1200	73.80	1	19.98	19.98	1200

### PEG–HINA Aggregation and Nanostructure Formation

Building on the established PEG self-assembly behavior, we now examine how drug incorporation affects nanocarrier formation and structural organization. The interplay among polymer–polymer, polymer–drug, and drug–drug interactions determines encapsulation efficiency and ultimately governs nanocarrier performance for therapeutic applications.

Drug encapsulation within PEG nanocarriers follows distinct concentration-dependent regimes reflecting fundamental thermodynamic and kinetic limitations (Figure 6 and Table 5). The encapsulation efficiency EE of HINA in the PEG nanocarriers was quantified as follows

6 $$EE\;\left(\%\right)=\frac{N_{encapsulated}}{N_{total}}\times 100$$

where *N*_total_ is the total number of HINA molecules in the system and *N*_encapsulated_ is the number of HINA molecules classified as being inside the PEG particle. Here, PEG chains were first grouped into aggregates using a distance-based clustering criterion (*r*_C_ < 15 Å), and the radius of the PEG particle *r*_PEG_ was obtained from the PEG center-of-mass positions. A HINA molecule was counted as encapsulated if its center of mass lay within the PEG aggregate and belonged to the same cluster as the PEG chains. HINA molecules located near the aggregate surface but not fully embedded were classified as the surface, and HINA molecules outside the aggregate were classified as the outside. The classification was based on the distance between the HINA center of mass and the PEG-particle center of mass *r*_HINA_: inside if *r*_PEG_ < *r*_HINA_ – 3 Å, outside if *r*_HINA_ > *r*_PEG_ + 10 Å, and surface otherwise. Unless stated otherwise, all EE values correspond to the fraction of encapsulated HINA molecules, as the fraction inside reported in Table 5.

**Figure 6:**
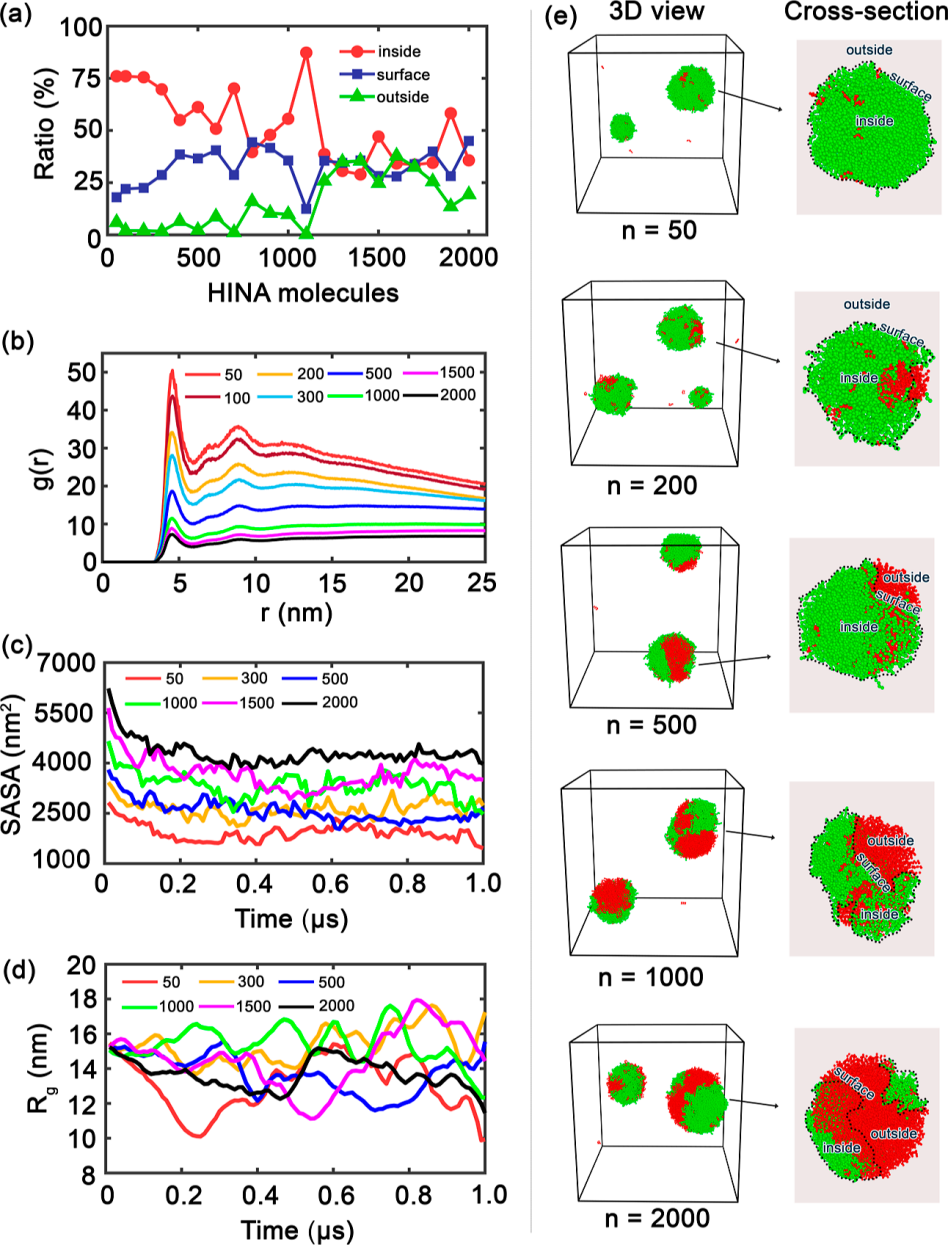
(a) Ratios of HINA molecules located inside, on the surface, or outside the PEG shell across different numbers of HINA molecules. (b) RDFs *g*(*r*) between PEG and HINA molecules. (c) Time evolution of SASA. (d) Radius of gyration (*R*_g_) trajectories over 1 μs of MD simulation. (e) Representative 3D snapshots (left) and cross-sectional views (right) of PEG–HINA for selected cases. Water is omitted for clarity.

**Table 5:** Distribution of HINA in PEG Nanocarriers at Various Total HINA Molecule Counts: Fractions Encapsulated Inside, Adsorbed on the Surface, and Outside, with Particle Size, Median, and Particle Count

HINA total (*n*)	HINA inside (*n*)	HINA on surface (*n*)	HINA outside (*n*)	fraction inside (%)	fraction on surface (%)	fraction outside (%)	particle size (nm)	size median (nm)	number of particles (*n*)
50	38	9	3	76.00	18.00	6.00	11.44, 8.17	9.81	2
100	76	22	2	76.00	22.00	2.00	10.84, 8.32	9.58	2
200	151	45	4	75.50	22.50	2.00	9.99, 9.03, 6.14	8.39	3
300	209	86	5	69.70	28.70	1.70	11.29, 8.33	9.81	2
400	220	154	26	55.00	38.50	6.50	10.73, 9.62	10.18	2
500	306	183	11	61.20	36.60	2.20	11.44, 11.12	11.28	2
600	305	243	52	50.83	40.50	8.67	12.46, 7.95	10.21	2
700	491	201	8	70.14	28.71	1.14	12.65, 7.39, 4.75	8.26	3
800	317	355	128	39.63	44.38	16.00	12.14, 9.58, 6.52	9.42	3
900	431	375	94	47.89	41.67	10.44	11.16, 9.15, 8.72	9.68	3
1000	556	346	98	55.60	35.60	9.80	13.78, 10.47	12.12	2
1100	960	137	3	87.27	12.45	0.27	16.03	16.03	1
1200	464	426	310	38.67	35.50	25.83	13.26, 10.37	11.82	2
1300	398	448	454	30.62	34.46	34.92	12.06, 11.94, 5.96	9.99	3
1400	405	499	496	28.93	35.64	35.43	11.97, 11.76, 8.68	10.81	3
1500	706	423	371	47.06	28.20	24.73	14.58	14.58	1
1600	547	448	605	34.19	28.00	37.81	14.49, 6.72	10.60	2
1700	572	576	552	33.65	33.88	32.47	14.47, 11.06	12.77	2
1800	621	719	460	34.50	39.94	25.55	14.34, 11.63	12.98	2
1900	1107	535	258	58.26	28.15	13.57	14.46, 9.16	11.81	2
2000	714	900	386	35.70	45.00	19.30	15.22, 11.42	13.32	2

At low total HINA molecule counts (*n* = 50–300), HINA molecules achieve >90% association with PEG aggregates, predominantly through core encapsulation (Table 5). This regime represents ideal drug loading, where enthalpic polymer–drug interactions drive spontaneous incorporation without significant competition for binding sites. The high encapsulation efficiency reflects favorable insertion into the hydrophobic PEG chain interior, minimizing unfavorable drug-water contacts.

Intermediate concentrations (*n* = 500–1500) exhibit saturation-limited behavior, where internal binding sites become occupied and subsequent drug molecules preferentially adsorb to aggregate surfaces (Table 5). The plateauing of core-bound drug fractions indicates finite capacity limits imposed by PEG chain packing and conformational constraints. Surface adsorption provides an alternative thermodynamically favorable state, though with potentially different release kinetics.

High total HINA molecule counts (*n* > 1500) demonstrate surface-dominated partitioning with increasing external drug populations, as evident from the distribution data in Table 5. This regime reflects the onset of drug–drug aggregation competing with polymer–drug interactions, ultimately leading to phase separation when local drug concentrations exceed solubility limits within the nanocarrier environment.

Radial distribution analysis confirms strong polymer–drug interactions through pronounced short-range correlations (*r* < 5 nm), with peak intensities scaling with drug concentration. This concentration-dependent enhancement indicates cooperative binding effects rather than simple site saturation, suggesting favorable drug–drug interactions within the polymer matrix that stabilize higher loading states. In addition, we found that finite-size effects do not affect the structural features because all RDFs tend to unity at large distance *r*.

SASA evolution demonstrates progressive drug burial kinetics, with initial rapid encapsulation (0–200 ns), followed by slower equilibration phases. Systems with higher total HINA molecule counts exhibit incomplete SASA reduction, indicating geometric constraints where drug molecules cannot achieve optimal polymer contact due to steric crowding. This behavior quantifies the trade-off between loading efficiency and thermodynamic stability.

Assembly compactness, measured through the radius of gyration, reveals drug-induced structural reorganization of the polymer matrix. Low total HINA molecule counts maintain compact, stable conformations (*R*_g_ ≈ 2.5–3.0 nm), while high total HINA molecule counts drive expansion and conformational fluctuations (*R*_g_ ≈ 3.5–4.5 nm). This swelling behavior reflects the balance between drug-induced polymer chain extension and excluded volume effects with implications for nanocarrier mechanical properties and stability.

Structural visualization reveals the morphological basis of concentration-dependent encapsulation mechanisms. Low-concentration assemblies (*n* = 50 total HINA molecules) exhibit core–shell architectures with complete drug burial, representing the thermodynamically optimal state for dilute systems. Intermediate total HINA molecule counts (*n* = 500–1000) show mixed core-surface distributions with maintained structural integrity, indicating the successful accommodation of moderate drug levels without significant destabilization. High-concentration systems (*n* = 2000 total HINA molecules) display surface-heavy drug distributions with emerging external drug populations, signaling the approach to solubility limits and potential phase separation thresholds. These structural transitions correlate directly with the particle size evolution and distribution patterns detailed in Table 5.

Assembly kinetics reveal pathways dependent on the total HINA molecule count governing nanocarrier formation (Figure 7). Systems with low total HINA molecule counts (100 molecules) follow classical nucleation–growth mechanisms, with PEG-driven cluster formation followed by drug recruitment and organization. The orderly assembly process yields thermodynamically stable core–shell structures with optimal encapsulation efficiency.

**Figure 7:**
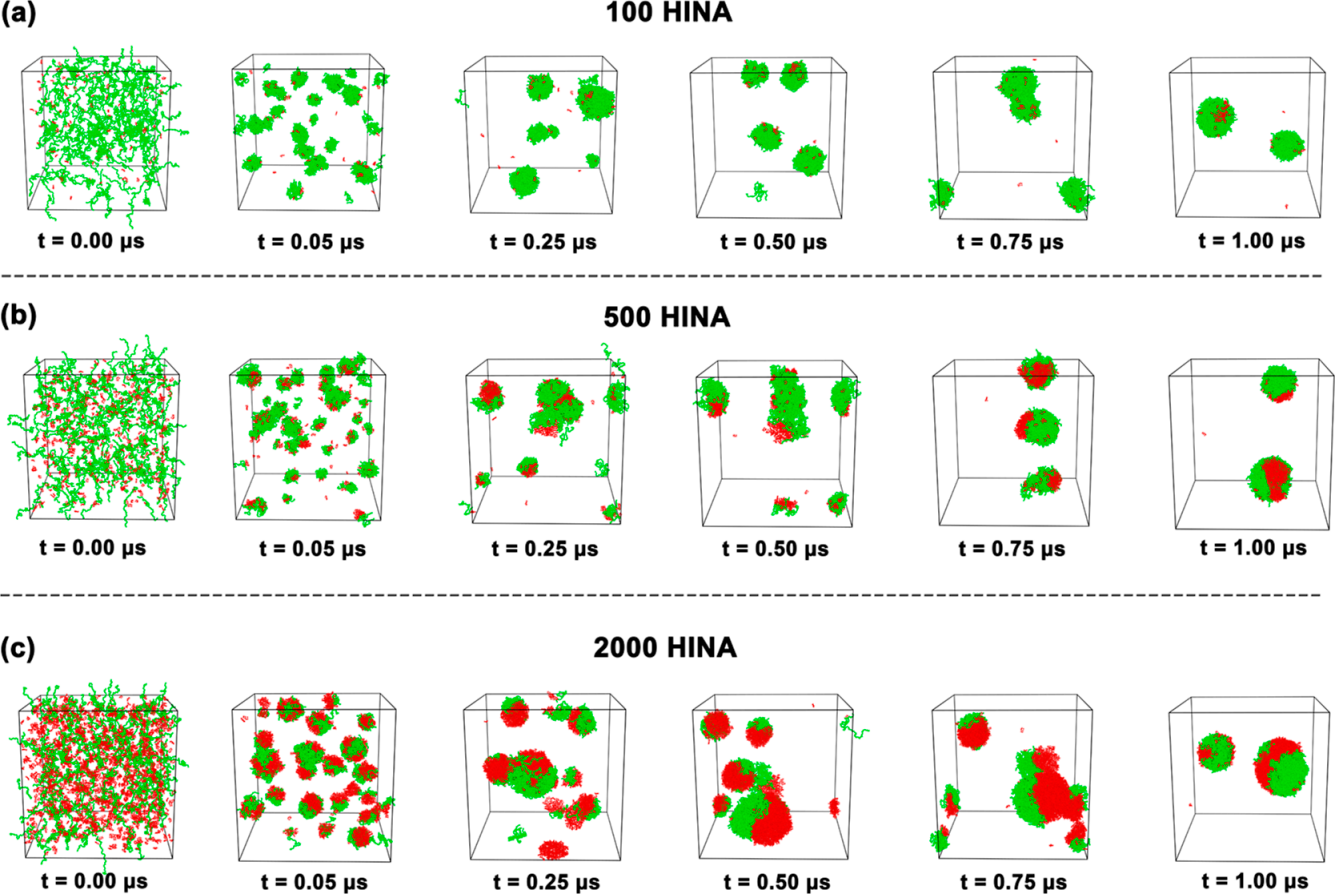
Time evolution of PEG–HINA assemblies at different concentrations. Representative MD snapshots at selected time points (0.00 to 1.00 μs) for systems containing (a) 100, (b) 500, and (c) 2000 HINA molecules. HINA molecules (red) and PEG chains (green) progressively self-assemble into nanoparticle-like aggregates in aqueous solution. Water is omitted for clarity.

Systems with moderate total HINA molecule counts (500 molecules) exhibit cooperative assembly, where drug molecules actively participate in nucleation, accelerating cluster formation through favorable polymer–drug–drug interactions. This regime achieves rapid nanocarrier formation while maintaining structural organization, representing an optimal balance between loading efficiency and assembly kinetics.

Systems with excessive total HINA molecule counts (2000 molecules) transition to competition-dominated assembly, where drug–drug aggregation competes with polymer–drug interactions. The resulting heterogeneous structures with significant external drug populations indicate system limitations and approaching thermodynamic instability. This behavior defines practical thresholds for total HINA molecule counts for maintaining the nanocarrier integrity and function.

These mechanistic insights provide rational design principles for optimizing drug loading while preserving nanocarrier performance with direct implications for pharmaceutical formulation strategies.

## Discussion

Our systematic investigation reveals fundamental concentration-dependent mechanisms governing PEG-based drug delivery systems (Table 5). At low drug loadings, HINA acts as a stabilizing agent, promoting compact PEG structures through favorable polymer–drug interactions driven by hydrophobic matching and hydrogen bonding. This cooperative assembly achieves high encapsulation efficiency (70–76% at low loadings, declining with increasing drug content) while maintaining structural stability.

As loading increases beyond the carrier’s optimal capacity, competing mechanisms emerge. Steric crowding within PEG cores forces HINA molecules toward energetically less favorable surface sites, leading to a decreased encapsulation efficiency and potential aggregate destabilization. The critical transition occurs around 300–400 HINA molecules per 200 PEG chains, representing a drug-to-polymer ratio that balances loading efficiency with system stability.

Particle size evolution with increasing HINA concentration reveals distinct morphological regimes that directly correlate with encapsulation behavior (Table 5). At low drug loadings (50–200 HINA molecules), median particle sizes remain relatively stable around 8.4–9.8 nm, reflecting compact core–shell architectures with efficient drug packing. This size range represents an optimal balance between drug accommodation and structural integrity.

As the HINA concentration increases to intermediate levels (300–600 molecules), median particle sizes show moderate growth to 9.8–11.3 nm, indicating structural expansion to accommodate additional drug molecules while maintaining encapsulation efficiency above 50%. This regime demonstrates the system’s capacity for controlled size modulation through drug loading.

At high concentrations (700–2000 HINA molecules), median particle sizes exhibit greater variability (8.3–16.0 nm) with an overall increasing trend, reaching maximum values of around 12–16 nm at the highest loadings. This size heterogeneity reflects the onset of competing assembly mechanisms where surface saturation and drug–drug interactions drive structural reorganization and potential aggregate destabilization.

These findings provide quantitative design principles for PEG-based nanocarriers: optimal drug loading ratios (PEG/HINA ≈ 4:1 to 2:1, corresponding to 200–400 HINA molecules per 200 PEG chains), predicted encapsulation efficiencies (70–76% for natural products with similar hydrophobicity at optimal loadings), and expected particle size ranges (8–16 nm for therapeutic applications). The concentration-dependent assembly pathways also inform processing strategies, where controlled mixing conditions could exploit the different kinetic regimes to achieve the desired particle characteristics.

An important consideration for translating these computational insights to experimental applications lies in the time scale disparities between MD simulations (microseconds) and real-world pharmaceutical processes (seconds to minutes). However, given the nanometer-scale dimensions of our nanocarrier systems, CG simulations can effectively reach equilibrium states within microsecond time scales, which is sufficient to capture the fundamental assembly dynamics. The rapid equilibration observed in our simulations suggests that fundamental PEG–drug interactions occur on time scales readily accessible during standard pharmaceutical mixing processes, making our predicted optimal formulation parameters directly applicable to experimental protocols.

The validated CG methodology enables exploration of diverse natural products beyond HINA, providing a computational framework for a sustainable drug delivery system design. By capturing the essential polymer–drug interactions while maintaining computational efficiency, this approach bridges molecular-level understanding with practical formulation development, potentially accelerating the translation of natural product therapeutics into clinical applications.

## Conclusions

A systematic analysis of concentration-dependent self-assembly mechanisms from comprehensive MD simulations allowed us to establish fundamental design principles for PEG-based nanocarriers. First, the Martini 3 CG models were compared to all-atom simulations, demonstrating that the CG models were able to capture essential structural properties while achieving computational efficiency gains necessary for large-scale studies of nanocarriers.

Systematic concentration studies revealed distinct mechanistic regimes governing nanocarrier formation and performance. We found that pure PEG systems exhibit nucleation–growth-coalescence kinetics with critical concentration thresholds determining particle size evolution (4–20 nm range) and assembly pathways. The established density relationships (993–1061 kg/m^3^) agree with experimental observations and show that the CG models have excellent predictive capabilities for bulk solution properties.

The drug encapsulation analysis quantified fundamental thermodynamic and kinetic limitations in PEG–HINA systems. At optimal low loadings (50–200 HINA molecules), the systems achieve maximum encapsulation efficiencies of 75.5–76.0% with compact particle sizes ranging from 8.4 to 9.8 nm median diameter. As HINA concentration increases to intermediate levels (300–500 molecules), encapsulation efficiency decreases to 61.2–69.7%, while particle sizes grow to 9.8–11.3 nm, indicating structural expansion to accommodate additional drug molecules. At high loadings, encapsulation efficiency drops significantly to 28.9–58.3% with highly variable particle sizes (11.8–16.0 nm median), reflecting surface saturation and competing drug–drug aggregation mechanisms.

The critical transition occurs at 300–400 drug molecules, where encapsulation efficiency drops from 69.7% to 55.0%, marking the threshold between stable core encapsulation and surface-dominated partitioning. This corresponds to PEG/HINA ratios that transition from optimal 4:1–2:1 to suboptimal conditions. Beyond optimal capacity, surface saturation and drug–drug aggregation compromise both encapsulation efficiency and structural stability, with external drug populations reaching 37.8% at maximum loadings.

The validated computational methodology enables the systematic exploration of diverse natural products beyond HINA, supporting the development of environmentally sustainable nanocarrier platforms. By bridging molecular-level understanding with practical design guidelines, this work contributes to both fundamental polymer physics and translational nanotechnology, establishing a framework for accelerating natural product therapeutics from computational design to clinical applications.

Several future research directions can be pursued to further advance drug delivery and natural product therapeutics. For instance, multidrug coencapsulation strategies should be explored, focusing on cooperative assembly mechanisms. Surface functionalization approaches should also be investigated to enhance the stability and targeting of nanocarriers. Lastly, it is crucial to experimentally validate predicted optimal formulation parameters for the clinical translation of natural product nanocarriers. These research directions hold great potential in expanding the scope of drug delivery and promoting the development of effective therapeutics based on natural products.

## Supplementary Material



## Data Availability

The data supporting the findings of this study are available from the corresponding author upon reasonable request.

## Notes

Probability distributions of bond lengths, angles, and dihedrals for PEO from all-atom and Martini 3 CG models; validation of all-atom and CG simulations against experimental neutron scattering data; NMR relaxation rates of PEG–water mixtures; and input files for MD simulations of PEG and HINA using the General AMBER and Martini 3 force fields, including topology, coordinate, and parameter files (PDF)
