# Supplementary material for: From Molecular Interactions to Nanocarrier Design: Coarse-Grained Modeling of PEG Self-Assembly and Hindsiilactone Encapsulation
Source: J Phys Chem B. 2026 Jan 2;130(2):841–53. doi: 10.1021/acs.jpcb.5c06860 (PMC12814515; doi:10.1021/acs.jpcb.5c06860)
Supplement: Supplementary file 1 [file jp5c06860_si_001.pdf]

Supporting Information

From Molecular Interactions to Nanocarrier Design: Coarse Grained Modeling of PEG Self-Assembly and Hindsiilactone Encapsulation

Thi H. Ho,*,†,‡ Hien Duy Tong,¶ Øivind Wilhelmsen,§ and Thuat T. Trinh*,§

†Laboratory for Computational Physics, Institute for Computational Science and Artificial Intelligence, Van Lang University, Ho Chi Minh City, 70000 Vietnam.

‡Faculty of Mechanical, Electrical, and Computer Engineering, Van Lang School of Technology, Van Lang University, Ho Chi Minh City, 70000 Vietnam.

¶Faculty of Engineering, Vietnamese-German University (VGU), Thu Dau Mot City, Binh Duong Province 75000, Vietnam

§Porelab, Department of Chemistry, Norwegian University of Science and Technology,Trondheim, Norway

E-mail: thi.hohuynh@vlu.edu.vn; thuat.trinh@ntnu.no

Contents

1. Probability distributions of bond lengths, angles, and dihedrals of PEO36 from AA and Martini 3 CG models 3

2. Validation of CG and AA models against experimental neutron scattering data 4

3. NMR relaxation rates R1 of PEG-water mixtures 5

4. Input files for MD simulations of PEG 6

4.1 General AMBER force field 6

1.1.1 Topology files 6

1.1.2 Structure coordinate files 118

1.1.3 Molecular Dynamics Parameter files 151

1.2 Martini 3 force field 157

1.2.1 Topology files 157

1.2.2 Structure coordinate files 163

1.2.3 Molecular Dynamics Parameter files 165

5. Input files for MD simulations of HINA 171

5.1 General AMBER force field 171
1.1.1 Topology files 171
1.2.2 Structure coordinate files 187
1.1.3 Molecular Dynamics Parameter files 189
2.2 Martini 3 force field 195
2.2.1 Topology files 195
2.2.3 Molecular Dynamics Parameter files 197

1. Probability distributions of bond lengths, angles, and dihedrals of PEO36 from AA and Martini 3 CG models

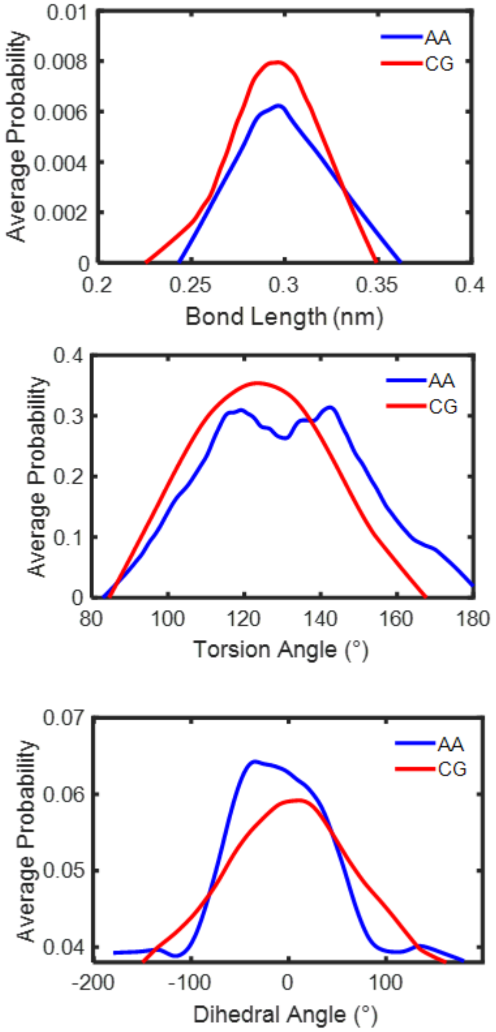

Figure S1. Structural distribution comparison between CG and AA simulations of PEO36 in water: (a) Bond length, (b) torsion angle, and (c) dihedral angle. CG results are shown in red and AA results in blue, illustrating the agreement and deviations between the two models.

2. Validation of CG and AA models against experimental neutron scattering data

Table S1. Radius of gyration R_g(Å) of PEO chains of length n: comparison between CG and AA simulations and experiment.

**Length n**	**R_g (Å)**				
	**Our work**		**Theory[a]**		**Experiment[b]**
	**CG**	**AA (GAFF)**	**CG**	**AA (CHARMM)**	
18	8.5 ± 0.1	9.6 ± 0.3	9.1 ± 0.10	8.8 ± 0.1	-
36	10.6 ± 0.7	12.6 ± 0.1	12.7 ± 0.2	13.2 ± 0.5	-
76	18.6 ± 0.6	20.6 ± 0.3	19.1± 0.7	20.4 ± 0.8	19.7

[a] Hwankyu Lee et al., *J. Phys. Chem. B* **2009,** *113,* 13186–13194.

[b] P. Thiyagarajan et al., *Macromolecules* **1995**, *28*, 7730.

3. NMR relaxation rates R_1 of PEG-water mixtures

Table S2. NMR relaxation rate R_1 for the PEG-water mixtures as a function of the PEG concentration c_{PEG}.

c_{PEG}	R_1 (s^{-1})				
	Our work		Theory[a]		Experiment[b]
	CG	AA (GAFF)	CG	AA (CHARMM)	
0.1	1.19	0.94	0.84	0.72	1.02
1	5.55	4.06	6.58	5.61	4.75
10	7.99	7.36	8.98	8.83	7.34

[a] Simon Gravelle et al., *J. Phys. Chem. B* **2023**, 127, 5601-5608

[b] Manazael Z. Jora et al., *J. Mol. Liq.* **2016**, *222*, 94-100.

4. Input files for MD simulations of PEG

4.1 General AMBER force field

1.1.1 Topology files

Main topology file “PEG_solvated.top”

```
[ defaults ]
; nbfunc        comb-rule       gen-pairs       fudgeLJ fudgeQQ
1               2               yes             0.5     0.8333333333


; Include PEG.itp topology
#include "PEG.itp"


; Ligand position restraints
#ifdef POSRES_PEG
#include "posre_PEG.itp"
#endif


; Include water topology
#include "tip3p.itp"


#ifdef POSRES_WATER
; Position restraint for each water oxygen
[ position_restraints ]
;  i funct       fcx        fcy        fcz
   1    1       1000       1000       1000
#endif


[ system ]
PEG in water
```

```
[ molecules ]
; Compound        nmols
PEG               1
SOL          262068
```

Include topology file "PEG.itp"

```
[ atomtypes ]
;name   bond_type    mass     charge   ptype   sigma        epsilon      Amb
 OW      OW        0.00000  0.00000   A    3.15061e-01   6.36386e-01
 HW      HW        0.00000  0.00000   A    0.00000e+00   0.00000e+00
 oh      oh        0.00000  0.00000   A    3.24287e-01   3.89112e-01 ; 1.82  0.0930
 ho      ho        0.00000  0.00000   A    5.37925e-02   1.96648e-02 ; 0.30  0.0047
 c3      c3        0.00000  0.00000   A    3.39771e-01   4.51035e-01 ; 1.91  0.1078
 h1      h1        0.00000  0.00000   A    2.42200e-01   8.70272e-02 ; 1.36  0.0208
 os      os        0.00000  0.00000   A    3.15610e-01   3.03758e-01 ; 1.77  0.0726

[ Moleculetype ]
;name           nrexcl
 PEG             3

[ atoms ]
;   nr  type  resi  res  atom  cgnr     charge      mass       ; qtot   bond_type
     1   oh     1   PEG    O1    1    -0.636800     16.00000 ; qtot -0.637
     2   ho     1   PEG    H2    2     0.405000      1.00800 ; qtot -0.232
     3   c3     1   PEG    C3    3     0.127400     12.01000 ; qtot -0.104
     4   h1     1   PEG    H4    4     0.035700      1.00800 ; qtot -0.069
     5   h1     1   PEG    H5    5     0.035700      1.00800 ; qtot -0.033
     6   c3     1   PEG    C6    6     0.129400     12.01000 ; qtot 0.096
```

7	h1	1	PEG	H7	7	0.042700	1.00800 ; qtot 0.139
8	h1	1	PEG	H8	8	0.042700	1.00800 ; qtot 0.182
9	os	1	PEG	O9	9	-0.431600	16.00000 ; qtot -0.250
10	c3	1	PEG	C10	10	0.130400	12.01000 ; qtot -0.119
11	h1	1	PEG	H11	11	0.042700	1.00800 ; qtot -0.077
12	h1	1	PEG	H12	12	0.042700	1.00800 ; qtot -0.034
13	c3	1	PEG	C13	13	0.129400	12.01000 ; qtot 0.095
14	h1	1	PEG	H14	14	0.042700	1.00800 ; qtot 0.138
15	h1	1	PEG	H15	15	0.042700	1.00800 ; qtot 0.181
16	os	1	PEG	O16	16	-0.429600	16.00000 ; qtot -0.249
17	c3	1	PEG	C17	17	0.130400	12.01000 ; qtot -0.118
18	h1	1	PEG	H18	18	0.042700	1.00800 ; qtot -0.076
19	h1	1	PEG	H19	19	0.042700	1.00800 ; qtot -0.033
20	c3	1	PEG	C20	20	0.129400	12.01000 ; qtot 0.096
21	h1	1	PEG	H21	21	0.042700	1.00800 ; qtot 0.139
22	h1	1	PEG	H22	22	0.042700	1.00800 ; qtot 0.182
23	os	1	PEG	O23	23	-0.429600	16.00000 ; qtot -0.248
24	c3	1	PEG	C24	24	0.130400	12.01000 ; qtot -0.117
25	h1	1	PEG	H25	25	0.042700	1.00800 ; qtot -0.075
26	h1	1	PEG	H26	26	0.042700	1.00800 ; qtot -0.032
27	c3	1	PEG	C27	27	0.130400	12.01000 ; qtot 0.098
28	h1	1	PEG	H28	28	0.042700	1.00800 ; qtot 0.141
29	h1	1	PEG	H29	29	0.042700	1.00800 ; qtot 0.184
30	os	1	PEG	O30	30	-0.429600	16.00000 ; qtot -0.246
31	c3	1	PEG	C31	31	0.130400	12.01000 ; qtot -0.115
32	h1	1	PEG	H32	32	0.042700	1.00800 ; qtot -0.073
33	h1	1	PEG	H33	33	0.042700	1.00800 ; qtot -0.030
34	c3	1	PEG	C34	34	0.129400	12.01000 ; qtot 0.099
35	h1	1	PEG	H35	35	0.042700	1.00800 ; qtot 0.142

36	h1	1	PEG	H36	36	0.042700	1.00800	; qtot 0.185
37	os	1	PEG	O37	37	-0.429600	16.00000	; qtot -0.245
38	c3	1	PEG	C38	38	0.129900	12.01000	; qtot -0.115
39	h1	1	PEG	H39	39	0.042700	1.00800	; qtot -0.072
40	h1	1	PEG	H40	40	0.042700	1.00800	; qtot -0.029
41	c3	1	PEG	C41	41	0.130400	12.01000	; qtot 0.101
42	h1	1	PEG	H42	42	0.042700	1.00800	; qtot 0.144
43	h1	1	PEG	H43	43	0.042700	1.00800	; qtot 0.186
44	os	1	PEG	O44	44	-0.429600	16.00000	; qtot -0.243
45	c3	1	PEG	C45	45	0.130400	12.01000	; qtot -0.113
46	h1	1	PEG	H46	46	0.042700	1.00800	; qtot -0.070
47	h1	1	PEG	H47	47	0.042700	1.00800	; qtot -0.027
48	c3	1	PEG	C48	48	0.129400	12.01000	; qtot 0.102
49	h1	1	PEG	H49	49	0.042700	1.00800	; qtot 0.145
50	h1	1	PEG	H50	50	0.042700	1.00800	; qtot 0.187
51	os	1	PEG	O51	51	-0.429600	16.00000	; qtot -0.242
52	c3	1	PEG	C52	52	0.129400	12.01000	; qtot -0.113
53	h1	1	PEG	H53	53	0.042700	1.00800	; qtot -0.070
54	h1	1	PEG	H54	54	0.042700	1.00800	; qtot -0.028
55	c3	1	PEG	C55	55	0.130400	12.01000	; qtot 0.103
56	h1	1	PEG	H56	56	0.042700	1.00800	; qtot 0.146
57	h1	1	PEG	H57	57	0.042700	1.00800	; qtot 0.188
58	os	1	PEG	O58	58	-0.429600	16.00000	; qtot -0.241
59	c3	1	PEG	C59	59	0.130400	12.01000	; qtot -0.111
60	h1	1	PEG	H60	60	0.042700	1.00800	; qtot -0.068
61	h1	1	PEG	H61	61	0.042700	1.00800	; qtot -0.026
62	c3	1	PEG	C62	62	0.129400	12.01000	; qtot 0.104
63	h1	1	PEG	H63	63	0.042700	1.00800	; qtot 0.147
64	h1	1	PEG	H64	64	0.042700	1.00800	; qtot 0.189

65	os	1	PEG	O65	65	-0.429600	16.00000	;	qtot	-0.240
66	c3	1	PEG	C66	66	0.129400	12.01000	;	qtot	-0.111
67	h1	1	PEG	H67	67	0.042700	1.00800	;	qtot	-0.068
68	h1	1	PEG	H68	68	0.042700	1.00800	;	qtot	-0.026
69	c3	1	PEG	C69	69	0.130400	12.01000	;	qtot	0.105
70	h1	1	PEG	H70	70	0.042700	1.00800	;	qtot	0.148
71	h1	1	PEG	H71	71	0.042700	1.00800	;	qtot	0.190
72	os	1	PEG	O72	72	-0.429600	16.00000	;	qtot	-0.239
73	c3	1	PEG	C73	73	0.130400	12.01000	;	qtot	-0.109
74	h1	1	PEG	H74	74	0.042700	1.00800	;	qtot	-0.066
75	h1	1	PEG	H75	75	0.042700	1.00800	;	qtot	-0.024
76	c3	1	PEG	C76	76	0.129900	12.01000	;	qtot	0.106
77	h1	1	PEG	H77	77	0.042700	1.00800	;	qtot	0.149
78	h1	1	PEG	H78	78	0.042700	1.00800	;	qtot	0.192
79	os	1	PEG	O79	79	-0.429600	16.00000	;	qtot	-0.238
80	c3	1	PEG	C80	80	0.130400	12.01000	;	qtot	-0.107
81	h1	1	PEG	H81	81	0.042700	1.00800	;	qtot	-0.065
82	h1	1	PEG	H82	82	0.042700	1.00800	;	qtot	-0.022
83	c3	1	PEG	C83	83	0.130400	12.01000	;	qtot	0.108
84	h1	1	PEG	H84	84	0.042700	1.00800	;	qtot	0.151
85	h1	1	PEG	H85	85	0.042700	1.00800	;	qtot	0.194
86	os	1	PEG	O86	86	-0.429600	16.00000	;	qtot	-0.236
87	c3	1	PEG	C87	87	0.130400	12.01000	;	qtot	-0.105
88	h1	1	PEG	H88	88	0.042700	1.00800	;	qtot	-0.063
89	h1	1	PEG	H89	89	0.042700	1.00800	;	qtot	-0.020
90	c3	1	PEG	C90	90	0.130400	12.01000	;	qtot	0.110
91	h1	1	PEG	H91	91	0.042700	1.00800	;	qtot	0.153
92	h1	1	PEG	H92	92	0.042700	1.00800	;	qtot	0.196
93	os	1	PEG	O93	93	-0.429600	16.00000	;	qtot	-0.234

94	c3	1	PEG	C94	94	0.130400	12.01000	; qtot -0.103
95	h1	1	PEG	H95	95	0.042700	1.00800	; qtot -0.061
96	h1	1	PEG	H96	96	0.042700	1.00800	; qtot -0.018
97	c3	1	PEG	C97	97	0.130400	12.01000	; qtot 0.112
98	h1	1	PEG	H98	98	0.042700	1.00800	; qtot 0.155
99	h1	1	PEG	H99	99	0.042700	1.00800	; qtot 0.198
100	os	1	PEG	O100	100	-0.429600	16.00000	; qtot -0.232
101	c3	1	PEG	C101	101	0.130400	12.01000	; qtot -0.101
102	h1	1	PEG	H102	102	0.042700	1.00800	; qtot -0.059
103	h1	1	PEG	H103	103	0.042700	1.00800	; qtot -0.016
104	c3	1	PEG	C104	104	0.130400	12.01000	; qtot 0.114
105	h1	1	PEG	H105	105	0.042700	1.00800	; qtot 0.157
106	h1	1	PEG	H106	106	0.042700	1.00800	; qtot 0.200
107	os	1	PEG	O107	107	-0.429600	16.00000	; qtot -0.230
108	c3	1	PEG	C108	108	0.130400	12.01000	; qtot -0.099
109	h1	1	PEG	H109	109	0.042700	1.00800	; qtot -0.057
110	h1	1	PEG	H110	110	0.042700	1.00800	; qtot -0.014
111	c3	1	PEG	C111	111	0.130400	12.01000	; qtot 0.116
112	h1	1	PEG	H112	112	0.042700	1.00800	; qtot 0.159
113	h1	1	PEG	H113	113	0.042700	1.00800	; qtot 0.202
114	os	1	PEG	O114	114	-0.429600	16.00000	; qtot -0.228
115	c3	1	PEG	C115	115	0.130400	12.01000	; qtot -0.097
116	h1	1	PEG	H116	116	0.042700	1.00800	; qtot -0.055
117	h1	1	PEG	H117	117	0.042700	1.00800	; qtot -0.012
118	c3	1	PEG	C118	118	0.130400	12.01000	; qtot 0.118
119	h1	1	PEG	H119	119	0.042700	1.00800	; qtot 0.161
120	h1	1	PEG	H120	120	0.042700	1.00800	; qtot 0.204
121	os	1	PEG	O121	121	-0.429600	16.00000	; qtot -0.226
122	c3	1	PEG	C122	122	0.130400	12.01000	; qtot -0.095

123	h1	1	PEG	H123	123	0.042700	1.00800	; qtot -0.053
124	h1	1	PEG	H124	124	0.042700	1.00800	; qtot -0.010
125	c3	1	PEG	C125	125	0.130400	12.01000	; qtot 0.120
126	h1	1	PEG	H126	126	0.042700	1.00800	; qtot 0.163
127	h1	1	PEG	H127	127	0.042700	1.00800	; qtot 0.206
128	os	1	PEG	O128	128	-0.429600	16.00000	; qtot -0.224
129	c3	1	PEG	C129	129	0.130400	12.01000	; qtot -0.093
130	h1	1	PEG	H130	130	0.042700	1.00800	; qtot -0.051
131	h1	1	PEG	H131	131	0.042700	1.00800	; qtot -0.008
132	c3	1	PEG	C132	132	0.130400	12.01000	; qtot 0.122
133	h1	1	PEG	H133	133	0.042700	1.00800	; qtot 0.165
134	h1	1	PEG	H134	134	0.042700	1.00800	; qtot 0.208
135	os	1	PEG	O135	135	-0.429600	16.00000	; qtot -0.222
136	c3	1	PEG	C136	136	0.130400	12.01000	; qtot -0.091
137	h1	1	PEG	H137	137	0.042700	1.00800	; qtot -0.049
138	h1	1	PEG	H138	138	0.042700	1.00800	; qtot -0.006
139	c3	1	PEG	C139	139	0.130400	12.01000	; qtot 0.124
140	h1	1	PEG	H140	140	0.042700	1.00800	; qtot 0.167
141	h1	1	PEG	H141	141	0.042700	1.00800	; qtot 0.210
142	os	1	PEG	O142	142	-0.429600	16.00000	; qtot -0.220
143	c3	1	PEG	C143	143	0.130400	12.01000	; qtot -0.089
144	h1	1	PEG	H144	144	0.042700	1.00800	; qtot -0.047
145	h1	1	PEG	H145	145	0.042700	1.00800	; qtot -0.004
146	c3	1	PEG	C146	146	0.130400	12.01000	; qtot 0.126
147	h1	1	PEG	H147	147	0.042700	1.00800	; qtot 0.169
148	h1	1	PEG	H148	148	0.042700	1.00800	; qtot 0.212
149	os	1	PEG	O149	149	-0.429600	16.00000	; qtot -0.218
150	c3	1	PEG	C150	150	0.130400	12.01000	; qtot -0.087
151	h1	1	PEG	H151	151	0.042700	1.00800	; qtot -0.045

152 h1 1 PEG H152 152 0.042700 1.00800 ; qtot -0.002

153 c3 1 PEG C153 153 0.130400 12.01000 ; qtot 0.128

154 h1 1 PEG H154 154 0.042700 1.00800 ; qtot 0.171

155 h1 1 PEG H155 155 0.042700 1.00800 ; qtot 0.214

156 os 1 PEG O156 156 -0.429600 16.00000 ; qtot -0.216

157 c3 1 PEG C157 157 0.130400 12.01000 ; qtot -0.085

158 h1 1 PEG H158 158 0.042700 1.00800 ; qtot -0.043

159 h1 1 PEG H159 159 0.042700 1.00800 ; qtot -0.000

160 c3 1 PEG C160 160 0.130400 12.01000 ; qtot 0.130

161 h1 1 PEG H161 161 0.042700 1.00800 ; qtot 0.173

162 h1 1 PEG H162 162 0.042700 1.00800 ; qtot 0.216

163 os 1 PEG O163 163 -0.429600 16.00000 ; qtot -0.214

164 c3 1 PEG C164 164 0.130400 12.01000 ; qtot -0.083

165 h1 1 PEG H165 165 0.042700 1.00800 ; qtot -0.041

166 h1 1 PEG H166 166 0.042700 1.00800 ; qtot 0.002

167 c3 1 PEG C167 167 0.130400 12.01000 ; qtot 0.132

168 h1 1 PEG H168 168 0.042700 1.00800 ; qtot 0.175

169 h1 1 PEG H169 169 0.042700 1.00800 ; qtot 0.218

170 os 1 PEG O170 170 -0.429600 16.00000 ; qtot -0.212

171 c3 1 PEG C171 171 0.130400 12.01000 ; qtot -0.081

172 h1 1 PEG H172 172 0.042700 1.00800 ; qtot -0.039

173 h1 1 PEG H173 173 0.042700 1.00800 ; qtot 0.004

174 c3 1 PEG C174 174 0.130400 12.01000 ; qtot 0.134

175 h1 1 PEG H175 175 0.042700 1.00800 ; qtot 0.177

176 h1 1 PEG H176 176 0.042700 1.00800 ; qtot 0.220

177 os 1 PEG O177 177 -0.429600 16.00000 ; qtot -0.210

178 c3 1 PEG C178 178 0.130400 12.01000 ; qtot -0.079

179 h1 1 PEG H179 179 0.042700 1.00800 ; qtot -0.037

180 h1 1 PEG H180 180 0.042700 1.00800 ; qtot 0.006

181	c3	1	PEG	C181	181	0.130400	12.01000	; qtot 0.136
182	h1	1	PEG	H182	182	0.042700	1.00800	; qtot 0.179
183	h1	1	PEG	H183	183	0.042700	1.00800	; qtot 0.222
184	os	1	PEG	O184	184	-0.429600	16.00000	; qtot -0.208
185	c3	1	PEG	C185	185	0.130400	12.01000	; qtot -0.077
186	h1	1	PEG	H186	186	0.042700	1.00800	; qtot -0.035
187	h1	1	PEG	H187	187	0.042700	1.00800	; qtot 0.008
188	c3	1	PEG	C188	188	0.130400	12.01000	; qtot 0.138
189	h1	1	PEG	H189	189	0.042700	1.00800	; qtot 0.181
190	h1	1	PEG	H190	190	0.042700	1.00800	; qtot 0.224
191	os	1	PEG	O191	191	-0.429600	16.00000	; qtot -0.206
192	c3	1	PEG	C192	192	0.130400	12.01000	; qtot -0.075
193	h1	1	PEG	H193	193	0.042700	1.00800	; qtot -0.033
194	h1	1	PEG	H194	194	0.042700	1.00800	; qtot 0.010
195	c3	1	PEG	C195	195	0.130400	12.01000	; qtot 0.140
196	h1	1	PEG	H196	196	0.042700	1.00800	; qtot 0.183
197	h1	1	PEG	H197	197	0.042700	1.00800	; qtot 0.226
198	os	1	PEG	O198	198	-0.429600	16.00000	; qtot -0.204
199	c3	1	PEG	C199	199	0.130400	12.01000	; qtot -0.073
200	h1	1	PEG	H200	200	0.042700	1.00800	; qtot -0.031
201	h1	1	PEG	H201	201	0.042700	1.00800	; qtot 0.012
202	c3	1	PEG	C202	202	0.130400	12.01000	; qtot 0.142
203	h1	1	PEG	H203	203	0.042700	1.00800	; qtot 0.185
204	h1	1	PEG	H204	204	0.042700	1.00800	; qtot 0.228
205	os	1	PEG	O205	205	-0.429600	16.00000	; qtot -0.202
206	c3	1	PEG	C206	206	0.130400	12.01000	; qtot -0.071
207	h1	1	PEG	H207	207	0.042700	1.00800	; qtot -0.029
208	h1	1	PEG	H208	208	0.042700	1.00800	; qtot 0.014
209	c3	1	PEG	C209	209	0.130400	12.01000	; qtot 0.144

210	h1	1	PEG	H210	210	0.042700	1.00800	;	qtot	0.187
211	h1	1	PEG	H211	211	0.042700	1.00800	;	qtot	0.230
212	os	1	PEG	O212	212	-0.429600	16.00000	;	qtot	-0.200
213	c3	1	PEG	C213	213	0.130400	12.01000	;	qtot	-0.069
214	h1	1	PEG	H214	214	0.042700	1.00800	;	qtot	-0.027
215	h1	1	PEG	H215	215	0.042700	1.00800	;	qtot	0.016
216	c3	1	PEG	C216	216	0.130400	12.01000	;	qtot	0.146
217	h1	1	PEG	H217	217	0.042700	1.00800	;	qtot	0.189
218	h1	1	PEG	H218	218	0.042700	1.00800	;	qtot	0.232
219	os	1	PEG	O219	219	-0.429600	16.00000	;	qtot	-0.198
220	c3	1	PEG	C220	220	0.130400	12.01000	;	qtot	-0.067
221	h1	1	PEG	H221	221	0.042700	1.00800	;	qtot	-0.025
222	h1	1	PEG	H222	222	0.042700	1.00800	;	qtot	0.018
223	c3	1	PEG	C223	223	0.130400	12.01000	;	qtot	0.148
224	h1	1	PEG	H224	224	0.042700	1.00800	;	qtot	0.191
225	h1	1	PEG	H225	225	0.042700	1.00800	;	qtot	0.234
226	os	1	PEG	O226	226	-0.429600	16.00000	;	qtot	-0.196
227	c3	1	PEG	C227	227	0.130400	12.01000	;	qtot	-0.065
228	h1	1	PEG	H228	228	0.042700	1.00800	;	qtot	-0.023
229	h1	1	PEG	H229	229	0.042700	1.00800	;	qtot	0.020
230	c3	1	PEG	C230	230	0.130400	12.01000	;	qtot	0.150
231	h1	1	PEG	H231	231	0.042700	1.00800	;	qtot	0.193
232	h1	1	PEG	H232	232	0.042700	1.00800	;	qtot	0.236
233	os	1	PEG	O233	233	-0.429600	16.00000	;	qtot	-0.194
234	c3	1	PEG	C234	234	0.130400	12.01000	;	qtot	-0.063
235	h1	1	PEG	H235	235	0.042700	1.00800	;	qtot	-0.021
236	h1	1	PEG	H236	236	0.042700	1.00800	;	qtot	0.022
237	c3	1	PEG	C237	237	0.130400	12.01000	;	qtot	0.152
238	h1	1	PEG	H238	238	0.042700	1.00800	;	qtot	0.195

239 h1 1 PEG H239 239 0.042700 1.00800 ; qtot 0.238

240 os 1 PEG O240 240 -0.429600 16.00000 ; qtot -0.192

241 c3 1 PEG C241 241 0.129900 12.01000 ; qtot -0.062

242 h1 1 PEG H242 242 0.042700 1.00800 ; qtot -0.019

243 h1 1 PEG H243 243 0.042700 1.00800 ; qtot 0.023

244 c3 1 PEG C244 244 0.130400 12.01000 ; qtot 0.154

245 h1 1 PEG H245 245 0.042700 1.00800 ; qtot 0.197

246 h1 1 PEG H246 246 0.042700 1.00800 ; qtot 0.239

247 os 1 PEG O247 247 -0.429600 16.00000 ; qtot -0.190

248 c3 1 PEG C248 248 0.130400 12.01000 ; qtot -0.060

249 h1 1 PEG H249 249 0.042700 1.00800 ; qtot -0.017

250 h1 1 PEG H250 250 0.042700 1.00800 ; qtot 0.025

251 c3 1 PEG C251 251 0.129400 12.01000 ; qtot 0.155

252 h1 1 PEG H252 252 0.042700 1.00800 ; qtot 0.198

253 h1 1 PEG H253 253 0.042700 1.00800 ; qtot 0.240

254 os 1 PEG O254 254 -0.429600 16.00000 ; qtot -0.189

255 c3 1 PEG C255 255 0.129400 12.01000 ; qtot -0.060

256 h1 1 PEG H256 256 0.042700 1.00800 ; qtot -0.017

257 h1 1 PEG H257 257 0.042700 1.00800 ; qtot 0.025

258 c3 1 PEG C258 258 0.130400 12.01000 ; qtot 0.156

259 h1 1 PEG H259 259 0.042700 1.00800 ; qtot 0.199

260 h1 1 PEG H260 260 0.042700 1.00800 ; qtot 0.241

261 os 1 PEG O261 261 -0.429600 16.00000 ; qtot -0.188

262 c3 1 PEG C262 262 0.130400 12.01000 ; qtot -0.058

263 h1 1 PEG H263 263 0.042700 1.00800 ; qtot -0.015

264 h1 1 PEG H264 264 0.042700 1.00800 ; qtot 0.027

265 c3 1 PEG C265 265 0.129400 12.01000 ; qtot 0.157

266 h1 1 PEG H266 266 0.042700 1.00800 ; qtot 0.200

267 h1 1 PEG H267 267 0.042700 1.00800 ; qtot 0.242

268	os	1	PEG	O268	268	-0.429600	16.00000	; qtot -0.187
269	c3	1	PEG	C269	269	0.129400	12.01000	; qtot -0.058
270	h1	1	PEG	H270	270	0.042700	1.00800	; qtot -0.015
271	h1	1	PEG	H271	271	0.042700	1.00800	; qtot 0.027
272	c3	1	PEG	C272	272	0.130400	12.01000	; qtot 0.158
273	h1	1	PEG	H273	273	0.042700	1.00800	; qtot 0.201
274	h1	1	PEG	H274	274	0.042700	1.00800	; qtot 0.243
275	os	1	PEG	O275	275	-0.429600	16.00000	; qtot -0.186
276	c3	1	PEG	C276	276	0.130400	12.01000	; qtot -0.056
277	h1	1	PEG	H277	277	0.042700	1.00800	; qtot -0.013
278	h1	1	PEG	H278	278	0.042700	1.00800	; qtot 0.029
279	c3	1	PEG	C279	279	0.129900	12.01000	; qtot 0.159
280	h1	1	PEG	H280	280	0.042700	1.00800	; qtot 0.202
281	h1	1	PEG	H281	281	0.042700	1.00800	; qtot 0.245
282	os	1	PEG	O282	282	-0.429600	16.00000	; qtot -0.185
283	c3	1	PEG	C283	283	0.129400	12.01000	; qtot -0.055
284	h1	1	PEG	H284	284	0.042700	1.00800	; qtot -0.013
285	h1	1	PEG	H285	285	0.042700	1.00800	; qtot 0.030
286	c3	1	PEG	C286	286	0.130400	12.01000	; qtot 0.160
287	h1	1	PEG	H287	287	0.042700	1.00800	; qtot 0.203
288	h1	1	PEG	H288	288	0.042700	1.00800	; qtot 0.246
289	os	1	PEG	O289	289	-0.429600	16.00000	; qtot -0.184
290	c3	1	PEG	C290	290	0.130400	12.01000	; qtot -0.053
291	h1	1	PEG	H291	291	0.042700	1.00800	; qtot -0.011
292	h1	1	PEG	H292	292	0.042700	1.00800	; qtot 0.032
293	c3	1	PEG	C293	293	0.130400	12.01000	; qtot 0.162
294	h1	1	PEG	H294	294	0.042700	1.00800	; qtot 0.205
295	h1	1	PEG	H295	295	0.042700	1.00800	; qtot 0.248
296	os	1	PEG	O296	296	-0.429600	16.00000	; qtot -0.182

```
297   c3    1   PEG  C297  297     0.129400     12.01000 ; qtot -0.052
298   h1    1   PEG  H298  298     0.042700      1.00800 ; qtot -0.010
299   h1    1   PEG  H299  299     0.042700      1.00800 ; qtot 0.033
300   c3    1   PEG  C300  300     0.130400     12.01000 ; qtot 0.163
301   h1    1   PEG  H301  301     0.042700      1.00800 ; qtot 0.206
302   h1    1   PEG  H302  302     0.042700      1.00800 ; qtot 0.249
303   os    1   PEG  O303  303    -0.429600     16.00000 ; qtot -0.181
304   c3    1   PEG  C304  304     0.129400     12.01000 ; qtot -0.051
305   h1    1   PEG  H305  305     0.042700      1.00800 ; qtot -0.009
306   h1    1   PEG  H306  306     0.042700      1.00800 ; qtot 0.034
307   c3    1   PEG  C307  307     0.130400     12.01000 ; qtot 0.164
308   h1    1   PEG  H308  308     0.042700      1.00800 ; qtot 0.207
309   h1    1   PEG  H309  309     0.042700      1.00800 ; qtot 0.250
310   os    1   PEG  O310  310    -0.431600     16.00000 ; qtot -0.182
311   c3    1   PEG  C311  311     0.129400     12.01000 ; qtot -0.052
312   h1    1   PEG  H312  312     0.042700      1.00800 ; qtot -0.010
313   h1    1   PEG  H313  313     0.042700      1.00800 ; qtot 0.033
314   c3    1   PEG  C314  314     0.127400     12.01000 ; qtot 0.160
315   h1    1   PEG  H315  315     0.035700      1.00800 ; qtot 0.196
316   h1    1   PEG  H316  316     0.035700      1.00800 ; qtot 0.232
317   oh    1   PEG  O317  317    -0.636800     16.00000 ; qtot -0.405
318   ho    1   PEG  H318  318     0.405000      1.00800 ; qtot -0.000

[ bonds ]
;   ai     aj funct   r             k
     1      2   1    9.7250e-02    4.4811e+05 ;     O1 - H2
     1      3   1    1.4242e-01    2.3861e+05 ;     O1 - C3
     3      4   1    1.0969e-01    2.8804e+05 ;     C3 - H4
     3      5   1    1.0969e-01    2.8804e+05 ;     C3 - H5
```

3	6	1	1.5354e-01	1.9154e+05 ;		C3 - C6
6	7	1	1.0969e-01	2.8804e+05 ;		C6 - H7
6	8	1	1.0969e-01	2.8804e+05 ;		C6 - H8
6	9	1	1.4273e-01	2.3620e+05 ;		C6 - O9
9	10	1	1.4273e-01	2.3620e+05 ;		O9 - C10
10	11	1	1.0969e-01	2.8804e+05 ;		C10 - H11
10	12	1	1.0969e-01	2.8804e+05 ;		C10 - H12
10	13	1	1.5354e-01	1.9154e+05 ;		C10 - C13
13	14	1	1.0969e-01	2.8804e+05 ;		C13 - H14
13	15	1	1.0969e-01	2.8804e+05 ;		C13 - H15
13	16	1	1.4273e-01	2.3620e+05 ;		C13 - O16
16	17	1	1.4273e-01	2.3620e+05 ;		O16 - C17
17	18	1	1.0969e-01	2.8804e+05 ;		C17 - H18
17	19	1	1.0969e-01	2.8804e+05 ;		C17 - H19
17	20	1	1.5354e-01	1.9154e+05 ;		C17 - C20
20	21	1	1.0969e-01	2.8804e+05 ;		C20 - H21
20	22	1	1.0969e-01	2.8804e+05 ;		C20 - H22
20	23	1	1.4273e-01	2.3620e+05 ;		C20 - O23
23	24	1	1.4273e-01	2.3620e+05 ;		O23 - C24
24	25	1	1.0969e-01	2.8804e+05 ;		C24 - H25
24	26	1	1.0969e-01	2.8804e+05 ;		C24 - H26
24	27	1	1.5354e-01	1.9154e+05 ;		C24 - C27
27	28	1	1.0969e-01	2.8804e+05 ;		C27 - H28
27	29	1	1.0969e-01	2.8804e+05 ;		C27 - H29
27	30	1	1.4273e-01	2.3620e+05 ;		C27 - O30
30	31	1	1.4273e-01	2.3620e+05 ;		O30 - C31
31	32	1	1.0969e-01	2.8804e+05 ;		C31 - H32
31	33	1	1.0969e-01	2.8804e+05 ;		C31 - H33
31	34	1	1.5354e-01	1.9154e+05 ;		C31 - C34

34	35	1	1.0969e-01	2.8804e+05 ;	C34 - H35
34	36	1	1.0969e-01	2.8804e+05 ;	C34 - H36
34	37	1	1.4273e-01	2.3620e+05 ;	C34 - O37
37	38	1	1.4273e-01	2.3620e+05 ;	O37 - C38
38	39	1	1.0969e-01	2.8804e+05 ;	C38 - H39
38	40	1	1.0969e-01	2.8804e+05 ;	C38 - H40
38	41	1	1.5354e-01	1.9154e+05 ;	C38 - C41
41	42	1	1.0969e-01	2.8804e+05 ;	C41 - H42
41	43	1	1.0969e-01	2.8804e+05 ;	C41 - H43
41	44	1	1.4273e-01	2.3620e+05 ;	C41 - O44
44	45	1	1.4273e-01	2.3620e+05 ;	O44 - C45
45	46	1	1.0969e-01	2.8804e+05 ;	C45 - H46
45	47	1	1.0969e-01	2.8804e+05 ;	C45 - H47
45	48	1	1.5354e-01	1.9154e+05 ;	C45 - C48
48	49	1	1.0969e-01	2.8804e+05 ;	C48 - H49
48	50	1	1.0969e-01	2.8804e+05 ;	C48 - H50
48	51	1	1.4273e-01	2.3620e+05 ;	C48 - O51
51	52	1	1.4273e-01	2.3620e+05 ;	O51 - C52
52	53	1	1.0969e-01	2.8804e+05 ;	C52 - H53
52	54	1	1.0969e-01	2.8804e+05 ;	C52 - H54
52	55	1	1.5354e-01	1.9154e+05 ;	C52 - C55
55	56	1	1.0969e-01	2.8804e+05 ;	C55 - H56
55	57	1	1.0969e-01	2.8804e+05 ;	C55 - H57
55	58	1	1.4273e-01	2.3620e+05 ;	C55 - O58
58	59	1	1.4273e-01	2.3620e+05 ;	O58 - C59
59	60	1	1.0969e-01	2.8804e+05 ;	C59 - H60
59	61	1	1.0969e-01	2.8804e+05 ;	C59 - H61
59	62	1	1.5354e-01	1.9154e+05 ;	C59 - C62
62	63	1	1.0969e-01	2.8804e+05 ;	C62 - H63

62	64	1	1.0969e-01	2.8804e+05 ;	C62 - H64
62	65	1	1.4273e-01	2.3620e+05 ;	C62 - O65
65	66	1	1.4273e-01	2.3620e+05 ;	O65 - C66
66	67	1	1.0969e-01	2.8804e+05 ;	C66 - H67
66	68	1	1.0969e-01	2.8804e+05 ;	C66 - H68
66	69	1	1.5354e-01	1.9154e+05 ;	C66 - C69
69	70	1	1.0969e-01	2.8804e+05 ;	C69 - H70
69	71	1	1.0969e-01	2.8804e+05 ;	C69 - H71
69	72	1	1.4273e-01	2.3620e+05 ;	C69 - O72
72	73	1	1.4273e-01	2.3620e+05 ;	O72 - C73
73	74	1	1.0969e-01	2.8804e+05 ;	C73 - H74
73	75	1	1.0969e-01	2.8804e+05 ;	C73 - H75
73	76	1	1.5354e-01	1.9154e+05 ;	C73 - C76
76	77	1	1.0969e-01	2.8804e+05 ;	C76 - H77
76	78	1	1.0969e-01	2.8804e+05 ;	C76 - H78
76	79	1	1.4273e-01	2.3620e+05 ;	C76 - O79
79	80	1	1.4273e-01	2.3620e+05 ;	O79 - C80
80	81	1	1.0969e-01	2.8804e+05 ;	C80 - H81
80	82	1	1.0969e-01	2.8804e+05 ;	C80 - H82
80	83	1	1.5354e-01	1.9154e+05 ;	C80 - C83
83	84	1	1.0969e-01	2.8804e+05 ;	C83 - H84
83	85	1	1.0969e-01	2.8804e+05 ;	C83 - H85
83	86	1	1.4273e-01	2.3620e+05 ;	C83 - O86
86	87	1	1.4273e-01	2.3620e+05 ;	O86 - C87
87	88	1	1.0969e-01	2.8804e+05 ;	C87 - H88
87	89	1	1.0969e-01	2.8804e+05 ;	C87 - H89
87	90	1	1.5354e-01	1.9154e+05 ;	C87 - C90
90	91	1	1.0969e-01	2.8804e+05 ;	C90 - H91
90	92	1	1.0969e-01	2.8804e+05 ;	C90 - H92

90	93	1	1.4273e-01	2.3620e+05 ;	C90 - O93
93	94	1	1.4273e-01	2.3620e+05 ;	O93 - C94
94	95	1	1.0969e-01	2.8804e+05 ;	C94 - H95
94	96	1	1.0969e-01	2.8804e+05 ;	C94 - H96
94	97	1	1.5354e-01	1.9154e+05 ;	C94 - C97
97	98	1	1.0969e-01	2.8804e+05 ;	C97 - H98
97	99	1	1.0969e-01	2.8804e+05 ;	C97 - H99
97	100	1	1.4273e-01	2.3620e+05 ;	C97 - O100
100	101	1	1.4273e-01	2.3620e+05 ;	O100 - C101
101	102	1	1.0969e-01	2.8804e+05 ;	C101 - H102
101	103	1	1.0969e-01	2.8804e+05 ;	C101 - H103
101	104	1	1.5354e-01	1.9154e+05 ;	C101 - C104
104	105	1	1.0969e-01	2.8804e+05 ;	C104 - H105
104	106	1	1.0969e-01	2.8804e+05 ;	C104 - H106
104	107	1	1.4273e-01	2.3620e+05 ;	C104 - O107
107	108	1	1.4273e-01	2.3620e+05 ;	O107 - C108
108	109	1	1.0969e-01	2.8804e+05 ;	C108 - H109
108	110	1	1.0969e-01	2.8804e+05 ;	C108 - H110
108	111	1	1.5354e-01	1.9154e+05 ;	C108 - C111
111	112	1	1.0969e-01	2.8804e+05 ;	C111 - H112
111	113	1	1.0969e-01	2.8804e+05 ;	C111 - H113
111	114	1	1.4273e-01	2.3620e+05 ;	C111 - O114
114	115	1	1.4273e-01	2.3620e+05 ;	O114 - C115
115	116	1	1.0969e-01	2.8804e+05 ;	C115 - H116
115	117	1	1.0969e-01	2.8804e+05 ;	C115 - H117
115	118	1	1.5354e-01	1.9154e+05 ;	C115 - C118
118	119	1	1.0969e-01	2.8804e+05 ;	C118 - H119
118	120	1	1.0969e-01	2.8804e+05 ;	C118 - H120
118	121	1	1.4273e-01	2.3620e+05 ;	C118 - O121

121	122	1	1.4273e-01	2.3620e+05 ;	O121 - C122
122	123	1	1.0969e-01	2.8804e+05 ;	C122 - H123
122	124	1	1.0969e-01	2.8804e+05 ;	C122 - H124
122	125	1	1.5354e-01	1.9154e+05 ;	C122 - C125
125	126	1	1.0969e-01	2.8804e+05 ;	C125 - H126
125	127	1	1.0969e-01	2.8804e+05 ;	C125 - H127
125	128	1	1.4273e-01	2.3620e+05 ;	C125 - O128
128	129	1	1.4273e-01	2.3620e+05 ;	O128 - C129
129	130	1	1.0969e-01	2.8804e+05 ;	C129 - H130
129	131	1	1.0969e-01	2.8804e+05 ;	C129 - H131
129	132	1	1.5354e-01	1.9154e+05 ;	C129 - C132
132	133	1	1.0969e-01	2.8804e+05 ;	C132 - H133
132	134	1	1.0969e-01	2.8804e+05 ;	C132 - H134
132	135	1	1.4273e-01	2.3620e+05 ;	C132 - O135
135	136	1	1.4273e-01	2.3620e+05 ;	O135 - C136
136	137	1	1.0969e-01	2.8804e+05 ;	C136 - H137
136	138	1	1.0969e-01	2.8804e+05 ;	C136 - H138
136	139	1	1.5354e-01	1.9154e+05 ;	C136 - C139
139	140	1	1.0969e-01	2.8804e+05 ;	C139 - H140
139	141	1	1.0969e-01	2.8804e+05 ;	C139 - H141
139	142	1	1.4273e-01	2.3620e+05 ;	C139 - O142
142	143	1	1.4273e-01	2.3620e+05 ;	O142 - C143
143	144	1	1.0969e-01	2.8804e+05 ;	C143 - H144
143	145	1	1.0969e-01	2.8804e+05 ;	C143 - H145
143	146	1	1.5354e-01	1.9154e+05 ;	C143 - C146
146	147	1	1.0969e-01	2.8804e+05 ;	C146 - H147
146	148	1	1.0969e-01	2.8804e+05 ;	C146 - H148
146	149	1	1.4273e-01	2.3620e+05 ;	C146 - O149
149	150	1	1.4273e-01	2.3620e+05 ;	O149 - C150

150	151	1	1.0969e-01	2.8804e+05 ;	C150 - H151
150	152	1	1.0969e-01	2.8804e+05 ;	C150 - H152
150	153	1	1.5354e-01	1.9154e+05 ;	C150 - C153
153	154	1	1.0969e-01	2.8804e+05 ;	C153 - H154
153	155	1	1.0969e-01	2.8804e+05 ;	C153 - H155
153	156	1	1.4273e-01	2.3620e+05 ;	C153 - O156
156	157	1	1.4273e-01	2.3620e+05 ;	O156 - C157
157	158	1	1.0969e-01	2.8804e+05 ;	C157 - H158
157	159	1	1.0969e-01	2.8804e+05 ;	C157 - H159
157	160	1	1.5354e-01	1.9154e+05 ;	C157 - C160
160	161	1	1.0969e-01	2.8804e+05 ;	C160 - H161
160	162	1	1.0969e-01	2.8804e+05 ;	C160 - H162
160	163	1	1.4273e-01	2.3620e+05 ;	C160 - O163
163	164	1	1.4273e-01	2.3620e+05 ;	O163 - C164
164	165	1	1.0969e-01	2.8804e+05 ;	C164 - H165
164	166	1	1.0969e-01	2.8804e+05 ;	C164 - H166
164	167	1	1.5354e-01	1.9154e+05 ;	C164 - C167
167	168	1	1.0969e-01	2.8804e+05 ;	C167 - H168
167	169	1	1.0969e-01	2.8804e+05 ;	C167 - H169
167	170	1	1.4273e-01	2.3620e+05 ;	C167 - O170
170	171	1	1.4273e-01	2.3620e+05 ;	O170 - C171
171	172	1	1.0969e-01	2.8804e+05 ;	C171 - H172
171	173	1	1.0969e-01	2.8804e+05 ;	C171 - H173
171	174	1	1.5354e-01	1.9154e+05 ;	C171 - C174
174	175	1	1.0969e-01	2.8804e+05 ;	C174 - H175
174	176	1	1.0969e-01	2.8804e+05 ;	C174 - H176
174	177	1	1.4273e-01	2.3620e+05 ;	C174 - O177
177	178	1	1.4273e-01	2.3620e+05 ;	O177 - C178
178	179	1	1.0969e-01	2.8804e+05 ;	C178 - H179

178	180	1	1.0969e-01	2.8804e+05 ;	C178 - H180
178	181	1	1.5354e-01	1.9154e+05 ;	C178 - C181
181	182	1	1.0969e-01	2.8804e+05 ;	C181 - H182
181	183	1	1.0969e-01	2.8804e+05 ;	C181 - H183
181	184	1	1.4273e-01	2.3620e+05 ;	C181 - O184
184	185	1	1.4273e-01	2.3620e+05 ;	O184 - C185
185	186	1	1.0969e-01	2.8804e+05 ;	C185 - H186
185	187	1	1.0969e-01	2.8804e+05 ;	C185 - H187
185	188	1	1.5354e-01	1.9154e+05 ;	C185 - C188
188	189	1	1.0969e-01	2.8804e+05 ;	C188 - H189
188	190	1	1.0969e-01	2.8804e+05 ;	C188 - H190
188	191	1	1.4273e-01	2.3620e+05 ;	C188 - O191
191	192	1	1.4273e-01	2.3620e+05 ;	O191 - C192
192	193	1	1.0969e-01	2.8804e+05 ;	C192 - H193
192	194	1	1.0969e-01	2.8804e+05 ;	C192 - H194
192	195	1	1.5354e-01	1.9154e+05 ;	C192 - C195
195	196	1	1.0969e-01	2.8804e+05 ;	C195 - H196
195	197	1	1.0969e-01	2.8804e+05 ;	C195 - H197
195	198	1	1.4273e-01	2.3620e+05 ;	C195 - O198
198	199	1	1.4273e-01	2.3620e+05 ;	O198 - C199
199	200	1	1.0969e-01	2.8804e+05 ;	C199 - H200
199	201	1	1.0969e-01	2.8804e+05 ;	C199 - H201
199	202	1	1.5354e-01	1.9154e+05 ;	C199 - C202
202	203	1	1.0969e-01	2.8804e+05 ;	C202 - H203
202	204	1	1.0969e-01	2.8804e+05 ;	C202 - H204
202	205	1	1.4273e-01	2.3620e+05 ;	C202 - O205
205	206	1	1.4273e-01	2.3620e+05 ;	O205 - C206
206	207	1	1.0969e-01	2.8804e+05 ;	C206 - H207
206	208	1	1.0969e-01	2.8804e+05 ;	C206 - H208

206	209	1	1.5354e-01	1.9154e+05 ;	C206 - C209
209	210	1	1.0969e-01	2.8804e+05 ;	C209 - H210
209	211	1	1.0969e-01	2.8804e+05 ;	C209 - H211
209	212	1	1.4273e-01	2.3620e+05 ;	C209 - O212
212	213	1	1.4273e-01	2.3620e+05 ;	O212 - C213
213	214	1	1.0969e-01	2.8804e+05 ;	C213 - H214
213	215	1	1.0969e-01	2.8804e+05 ;	C213 - H215
213	216	1	1.5354e-01	1.9154e+05 ;	C213 - C216
216	217	1	1.0969e-01	2.8804e+05 ;	C216 - H217
216	218	1	1.0969e-01	2.8804e+05 ;	C216 - H218
216	219	1	1.4273e-01	2.3620e+05 ;	C216 - O219
219	220	1	1.4273e-01	2.3620e+05 ;	O219 - C220
220	221	1	1.0969e-01	2.8804e+05 ;	C220 - H221
220	222	1	1.0969e-01	2.8804e+05 ;	C220 - H222
220	223	1	1.5354e-01	1.9154e+05 ;	C220 - C223
223	224	1	1.0969e-01	2.8804e+05 ;	C223 - H224
223	225	1	1.0969e-01	2.8804e+05 ;	C223 - H225
223	226	1	1.4273e-01	2.3620e+05 ;	C223 - O226
226	227	1	1.4273e-01	2.3620e+05 ;	O226 - C227
227	228	1	1.0969e-01	2.8804e+05 ;	C227 - H228
227	229	1	1.0969e-01	2.8804e+05 ;	C227 - H229
227	230	1	1.5354e-01	1.9154e+05 ;	C227 - C230
230	231	1	1.0969e-01	2.8804e+05 ;	C230 - H231
230	232	1	1.0969e-01	2.8804e+05 ;	C230 - H232
230	233	1	1.4273e-01	2.3620e+05 ;	C230 - O233
233	234	1	1.4273e-01	2.3620e+05 ;	O233 - C234
234	235	1	1.0969e-01	2.8804e+05 ;	C234 - H235
234	236	1	1.0969e-01	2.8804e+05 ;	C234 - H236
234	237	1	1.5354e-01	1.9154e+05 ;	C234 - C237

237	238	1	1.0969e-01	2.8804e+05 ;	C237 - H238
237	239	1	1.0969e-01	2.8804e+05 ;	C237 - H239
237	240	1	1.4273e-01	2.3620e+05 ;	C237 - O240
240	241	1	1.4273e-01	2.3620e+05 ;	O240 - C241
241	242	1	1.0969e-01	2.8804e+05 ;	C241 - H242
241	243	1	1.0969e-01	2.8804e+05 ;	C241 - H243
241	244	1	1.5354e-01	1.9154e+05 ;	C241 - C244
244	245	1	1.0969e-01	2.8804e+05 ;	C244 - H245
244	246	1	1.0969e-01	2.8804e+05 ;	C244 - H246
244	247	1	1.4273e-01	2.3620e+05 ;	C244 - O247
247	248	1	1.4273e-01	2.3620e+05 ;	O247 - C248
248	249	1	1.0969e-01	2.8804e+05 ;	C248 - H249
248	250	1	1.0969e-01	2.8804e+05 ;	C248 - H250
248	251	1	1.5354e-01	1.9154e+05 ;	C248 - C251
251	252	1	1.0969e-01	2.8804e+05 ;	C251 - H252
251	253	1	1.0969e-01	2.8804e+05 ;	C251 - H253
251	254	1	1.4273e-01	2.3620e+05 ;	C251 - O254
254	255	1	1.4273e-01	2.3620e+05 ;	O254 - C255
255	256	1	1.0969e-01	2.8804e+05 ;	C255 - H256
255	257	1	1.0969e-01	2.8804e+05 ;	C255 - H257
255	258	1	1.5354e-01	1.9154e+05 ;	C255 - C258
258	259	1	1.0969e-01	2.8804e+05 ;	C258 - H259
258	260	1	1.0969e-01	2.8804e+05 ;	C258 - H260
258	261	1	1.4273e-01	2.3620e+05 ;	C258 - O261
261	262	1	1.4273e-01	2.3620e+05 ;	O261 - C262
262	263	1	1.0969e-01	2.8804e+05 ;	C262 - H263
262	264	1	1.0969e-01	2.8804e+05 ;	C262 - H264
262	265	1	1.5354e-01	1.9154e+05 ;	C262 - C265
265	266	1	1.0969e-01	2.8804e+05 ;	C265 - H266

265	267	1	1.0969e-01	2.8804e+05 ;	C265 - H267
265	268	1	1.4273e-01	2.3620e+05 ;	C265 - O268
268	269	1	1.4273e-01	2.3620e+05 ;	O268 - C269
269	270	1	1.0969e-01	2.8804e+05 ;	C269 - H270
269	271	1	1.0969e-01	2.8804e+05 ;	C269 - H271
269	272	1	1.5354e-01	1.9154e+05 ;	C269 - C272
272	273	1	1.0969e-01	2.8804e+05 ;	C272 - H273
272	274	1	1.0969e-01	2.8804e+05 ;	C272 - H274
272	275	1	1.4273e-01	2.3620e+05 ;	C272 - O275
275	276	1	1.4273e-01	2.3620e+05 ;	O275 - C276
276	277	1	1.0969e-01	2.8804e+05 ;	C276 - H277
276	278	1	1.0969e-01	2.8804e+05 ;	C276 - H278
276	279	1	1.5354e-01	1.9154e+05 ;	C276 - C279
279	280	1	1.0969e-01	2.8804e+05 ;	C279 - H280
279	281	1	1.0969e-01	2.8804e+05 ;	C279 - H281
279	282	1	1.4273e-01	2.3620e+05 ;	C279 - O282
282	283	1	1.4273e-01	2.3620e+05 ;	O282 - C283
283	284	1	1.0969e-01	2.8804e+05 ;	C283 - H284
283	285	1	1.0969e-01	2.8804e+05 ;	C283 - H285
283	286	1	1.5354e-01	1.9154e+05 ;	C283 - C286
286	287	1	1.0969e-01	2.8804e+05 ;	C286 - H287
286	288	1	1.0969e-01	2.8804e+05 ;	C286 - H288
286	289	1	1.4273e-01	2.3620e+05 ;	C286 - O289
289	290	1	1.4273e-01	2.3620e+05 ;	O289 - C290
290	291	1	1.0969e-01	2.8804e+05 ;	C290 - H291
290	292	1	1.0969e-01	2.8804e+05 ;	C290 - H292
290	293	1	1.5354e-01	1.9154e+05 ;	C290 - C293
293	294	1	1.0969e-01	2.8804e+05 ;	C293 - H294
293	295	1	1.0969e-01	2.8804e+05 ;	C293 - H295

```
   293    296   1    1.4273e-01    2.3620e+05 ;   C293 - O296
   296    297   1    1.4273e-01    2.3620e+05 ;   O296 - C297
   297    298   1    1.0969e-01    2.8804e+05 ;   C297 - H298
   297    299   1    1.0969e-01    2.8804e+05 ;   C297 - H299
   297    300   1    1.5354e-01    1.9154e+05 ;   C297 - C300
   300    301   1    1.0969e-01    2.8804e+05 ;   C300 - H301
   300    302   1    1.0969e-01    2.8804e+05 ;   C300 - H302
   300    303   1    1.4273e-01    2.3620e+05 ;   C300 - O303
   303    304   1    1.4273e-01    2.3620e+05 ;   O303 - C304
   304    305   1    1.0969e-01    2.8804e+05 ;   C304 - H305
   304    306   1    1.0969e-01    2.8804e+05 ;   C304 - H306
   304    307   1    1.5354e-01    1.9154e+05 ;   C304 - C307
   307    308   1    1.0969e-01    2.8804e+05 ;   C307 - H308
   307    309   1    1.0969e-01    2.8804e+05 ;   C307 - H309
   307    310   1    1.4273e-01    2.3620e+05 ;   C307 - O310
   310    311   1    1.4273e-01    2.3620e+05 ;   O310 - C311
   311    312   1    1.0969e-01    2.8804e+05 ;   C311 - H312
   311    313   1    1.0969e-01    2.8804e+05 ;   C311 - H313
   311    314   1    1.5354e-01    1.9154e+05 ;   C311 - C314
   314    315   1    1.0969e-01    2.8804e+05 ;   C314 - H315
   314    316   1    1.0969e-01    2.8804e+05 ;   C314 - H316
   314    317   1    1.4242e-01    2.3861e+05 ;   C314 - O317
   317    318   1    9.7250e-02    4.4811e+05 ;   O317 - H318

[ pairs ]
;   ai     aj    funct
     1      7      1 ;     O1 - H7
     1      8      1 ;     O1 - H8
     1      9      1 ;     O1 - O9
```

2	4	1 ;	H2 - H4
2	5	1 ;	H2 - H5
2	6	1 ;	H2 - C6
3	10	1 ;	C3 - C10
4	7	1 ;	H4 - H7
4	8	1 ;	H4 - H8
4	9	1 ;	H4 - O9
5	7	1 ;	H5 - H7
5	8	1 ;	H5 - H8
5	9	1 ;	H5 - O9
6	11	1 ;	C6 - H11
6	12	1 ;	C6 - H12
6	13	1 ;	C6 - C13
7	10	1 ;	H7 - C10
8	10	1 ;	H8 - C10
9	14	1 ;	O9 - H14
9	15	1 ;	O9 - H15
9	16	1 ;	O9 - O16
10	17	1 ;	C10 - C17
11	14	1 ;	H11 - H14
11	15	1 ;	H11 - H15
11	16	1 ;	H11 - O16
12	14	1 ;	H12 - H14
12	15	1 ;	H12 - H15
12	16	1 ;	H12 - O16
13	18	1 ;	C13 - H18
13	19	1 ;	C13 - H19
13	20	1 ;	C13 - C20
14	17	1 ;	H14 - C17

15	17	1 ;	H15 - C17
16	21	1 ;	O16 - H21
16	22	1 ;	O16 - H22
16	23	1 ;	O16 - O23
17	24	1 ;	C17 - C24
18	21	1 ;	H18 - H21
18	22	1 ;	H18 - H22
18	23	1 ;	H18 - O23
19	21	1 ;	H19 - H21
19	22	1 ;	H19 - H22
19	23	1 ;	H19 - O23
20	25	1 ;	C20 - H25
20	26	1 ;	C20 - H26
20	27	1 ;	C20 - C27
21	24	1 ;	H21 - C24
22	24	1 ;	H22 - C24
23	28	1 ;	O23 - H28
23	29	1 ;	O23 - H29
23	30	1 ;	O23 - O30
24	31	1 ;	C24 - C31
25	28	1 ;	H25 - H28
25	29	1 ;	H25 - H29
25	30	1 ;	H25 - O30
26	28	1 ;	H26 - H28
26	29	1 ;	H26 - H29
26	30	1 ;	H26 - O30
27	32	1 ;	C27 - H32
27	33	1 ;	C27 - H33
27	34	1 ;	C27 - C34

28	31	1 ;	H28 - C31
29	31	1 ;	H29 - C31
30	35	1 ;	O30 - H35
30	36	1 ;	O30 - H36
30	37	1 ;	O30 - O37
31	38	1 ;	C31 - C38
32	35	1 ;	H32 - H35
32	36	1 ;	H32 - H36
32	37	1 ;	H32 - O37
33	35	1 ;	H33 - H35
33	36	1 ;	H33 - H36
33	37	1 ;	H33 - O37
34	39	1 ;	C34 - H39
34	40	1 ;	C34 - H40
34	41	1 ;	C34 - C41
35	38	1 ;	H35 - C38
36	38	1 ;	H36 - C38
37	42	1 ;	O37 - H42
37	43	1 ;	O37 - H43
37	44	1 ;	O37 - O44
38	45	1 ;	C38 - C45
39	42	1 ;	H39 - H42
39	43	1 ;	H39 - H43
39	44	1 ;	H39 - O44
40	42	1 ;	H40 - H42
40	43	1 ;	H40 - H43
40	44	1 ;	H40 - O44
41	46	1 ;	C41 - H46
41	47	1 ;	C41 - H47

41	48	1 ;	C41 - C48
42	45	1 ;	H42 - C45
43	45	1 ;	H43 - C45
44	49	1 ;	O44 - H49
44	50	1 ;	O44 - H50
44	51	1 ;	O44 - O51
45	52	1 ;	C45 - C52
46	49	1 ;	H46 - H49
46	50	1 ;	H46 - H50
46	51	1 ;	H46 - O51
47	49	1 ;	H47 - H49
47	50	1 ;	H47 - H50
47	51	1 ;	H47 - O51
48	53	1 ;	C48 - H53
48	54	1 ;	C48 - H54
48	55	1 ;	C48 - C55
49	52	1 ;	H49 - C52
50	52	1 ;	H50 - C52
51	56	1 ;	O51 - H56
51	57	1 ;	O51 - H57
51	58	1 ;	O51 - O58
52	59	1 ;	C52 - C59
53	56	1 ;	H53 - H56
53	57	1 ;	H53 - H57
53	58	1 ;	H53 - O58
54	56	1 ;	H54 - H56
54	57	1 ;	H54 - H57
54	58	1 ;	H54 - O58
55	60	1 ;	C55 - H60

55	61	1 ;	C55 - H61
55	62	1 ;	C55 - C62
56	59	1 ;	H56 - C59
57	59	1 ;	H57 - C59
58	63	1 ;	O58 - H63
58	64	1 ;	O58 - H64
58	65	1 ;	O58 - O65
59	66	1 ;	C59 - C66
60	63	1 ;	H60 - H63
60	64	1 ;	H60 - H64
60	65	1 ;	H60 - O65
61	63	1 ;	H61 - H63
61	64	1 ;	H61 - H64
61	65	1 ;	H61 - O65
62	67	1 ;	C62 - H67
62	68	1 ;	C62 - H68
62	69	1 ;	C62 - C69
63	66	1 ;	H63 - C66
64	66	1 ;	H64 - C66
65	70	1 ;	O65 - H70
65	71	1 ;	O65 - H71
65	72	1 ;	O65 - O72
66	73	1 ;	C66 - C73
67	70	1 ;	H67 - H70
67	71	1 ;	H67 - H71
67	72	1 ;	H67 - O72
68	70	1 ;	H68 - H70
68	71	1 ;	H68 - H71
68	72	1 ;	H68 - O72

69	74	1 ;	C69 - H74
69	75	1 ;	C69 - H75
69	76	1 ;	C69 - C76
70	73	1 ;	H70 - C73
71	73	1 ;	H71 - C73
72	77	1 ;	O72 - H77
72	78	1 ;	O72 - H78
72	79	1 ;	O72 - O79
73	80	1 ;	C73 - C80
74	77	1 ;	H74 - H77
74	78	1 ;	H74 - H78
74	79	1 ;	H74 - O79
75	77	1 ;	H75 - H77
75	78	1 ;	H75 - H78
75	79	1 ;	H75 - O79
76	81	1 ;	C76 - H81
76	82	1 ;	C76 - H82
76	83	1 ;	C76 - C83
77	80	1 ;	H77 - C80
78	80	1 ;	H78 - C80
79	84	1 ;	O79 - H84
79	85	1 ;	O79 - H85
79	86	1 ;	O79 - O86
80	87	1 ;	C80 - C87
81	84	1 ;	H81 - H84
81	85	1 ;	H81 - H85
81	86	1 ;	H81 - O86
82	84	1 ;	H82 - H84
82	85	1 ;	H82 - H85

82	86	1 ;	H82 - O86
83	88	1 ;	C83 - H88
83	89	1 ;	C83 - H89
83	90	1 ;	C83 - C90
84	87	1 ;	H84 - C87
85	87	1 ;	H85 - C87
86	91	1 ;	O86 - H91
86	92	1 ;	O86 - H92
86	93	1 ;	O86 - O93
87	94	1 ;	C87 - C94
88	91	1 ;	H88 - H91
88	92	1 ;	H88 - H92
88	93	1 ;	H88 - O93
89	91	1 ;	H89 - H91
89	92	1 ;	H89 - H92
89	93	1 ;	H89 - O93
90	95	1 ;	C90 - H95
90	96	1 ;	C90 - H96
90	97	1 ;	C90 - C97
91	94	1 ;	H91 - C94
92	94	1 ;	H92 - C94
93	98	1 ;	O93 - H98
93	99	1 ;	O93 - H99
93	100	1 ;	O93 - O100
94	101	1 ;	C94 - C101
95	98	1 ;	H95 - H98
95	99	1 ;	H95 - H99
95	100	1 ;	H95 - O100
96	98	1 ;	H96 - H98

96	99	1 ;	H96 - H99
96	100	1 ;	H96 - O100
97	102	1 ;	C97 - H102
97	103	1 ;	C97 - H103
97	104	1 ;	C97 - C104
98	101	1 ;	H98 - C101
99	101	1 ;	H99 - C101
100	105	1 ;	O100 - H105
100	106	1 ;	O100 - H106
100	107	1 ;	O100 - O107
101	108	1 ;	C101 - C108
102	105	1 ;	H102 - H105
102	106	1 ;	H102 - H106
102	107	1 ;	H102 - O107
103	105	1 ;	H103 - H105
103	106	1 ;	H103 - H106
103	107	1 ;	H103 - O107
104	109	1 ;	C104 - H109
104	110	1 ;	C104 - H110
104	111	1 ;	C104 - C111
105	108	1 ;	H105 - C108
106	108	1 ;	H106 - C108
107	112	1 ;	O107 - H112
107	113	1 ;	O107 - H113
107	114	1 ;	O107 - O114
108	115	1 ;	C108 - C115
109	112	1 ;	H109 - H112
109	113	1 ;	H109 - H113
109	114	1 ;	H109 - O114

110	112	1 ;	H110 - H112
110	113	1 ;	H110 - H113
110	114	1 ;	H110 - O114
111	116	1 ;	C111 - H116
111	117	1 ;	C111 - H117
111	118	1 ;	C111 - C118
112	115	1 ;	H112 - C115
113	115	1 ;	H113 - C115
114	119	1 ;	O114 - H119
114	120	1 ;	O114 - H120
114	121	1 ;	O114 - O121
115	122	1 ;	C115 - C122
116	119	1 ;	H116 - H119
116	120	1 ;	H116 - H120
116	121	1 ;	H116 - O121
117	119	1 ;	H117 - H119
117	120	1 ;	H117 - H120
117	121	1 ;	H117 - O121
118	123	1 ;	C118 - H123
118	124	1 ;	C118 - H124
118	125	1 ;	C118 - C125
119	122	1 ;	H119 - C122
120	122	1 ;	H120 - C122
121	126	1 ;	O121 - H126
121	127	1 ;	O121 - H127
121	128	1 ;	O121 - O128
122	129	1 ;	C122 - C129
123	126	1 ;	H123 - H126
123	127	1 ;	H123 - H127

123	128	1 ;	H123 - O128
124	126	1 ;	H124 - H126
124	127	1 ;	H124 - H127
124	128	1 ;	H124 - O128
125	130	1 ;	C125 - H130
125	131	1 ;	C125 - H131
125	132	1 ;	C125 - C132
126	129	1 ;	H126 - C129
127	129	1 ;	H127 - C129
128	133	1 ;	O128 - H133
128	134	1 ;	O128 - H134
128	135	1 ;	O128 - O135
129	136	1 ;	C129 - C136
130	133	1 ;	H130 - H133
130	134	1 ;	H130 - H134
130	135	1 ;	H130 - O135
131	133	1 ;	H131 - H133
131	134	1 ;	H131 - H134
131	135	1 ;	H131 - O135
132	137	1 ;	C132 - H137
132	138	1 ;	C132 - H138
132	139	1 ;	C132 - C139
133	136	1 ;	H133 - C136
134	136	1 ;	H134 - C136
135	140	1 ;	O135 - H140
135	141	1 ;	O135 - H141
135	142	1 ;	O135 - O142
136	143	1 ;	C136 - C143
137	140	1 ;	H137 - H140

137	141	1 ;	H137 - H141
137	142	1 ;	H137 - O142
138	140	1 ;	H138 - H140
138	141	1 ;	H138 - H141
138	142	1 ;	H138 - O142
139	144	1 ;	C139 - H144
139	145	1 ;	C139 - H145
139	146	1 ;	C139 - C146
140	143	1 ;	H140 - C143
141	143	1 ;	H141 - C143
142	147	1 ;	O142 - H147
142	148	1 ;	O142 - H148
142	149	1 ;	O142 - O149
143	150	1 ;	C143 - C150
144	147	1 ;	H144 - H147
144	148	1 ;	H144 - H148
144	149	1 ;	H144 - O149
145	147	1 ;	H145 - H147
145	148	1 ;	H145 - H148
145	149	1 ;	H145 - O149
146	151	1 ;	C146 - H151
146	152	1 ;	C146 - H152
146	153	1 ;	C146 - C153
147	150	1 ;	H147 - C150
148	150	1 ;	H148 - C150
149	154	1 ;	O149 - H154
149	155	1 ;	O149 - H155
149	156	1 ;	O149 - O156
150	157	1 ;	C150 - C157

151	154	1 ;	H151 - H154
151	155	1 ;	H151 - H155
151	156	1 ;	H151 - O156
152	154	1 ;	H152 - H154
152	155	1 ;	H152 - H155
152	156	1 ;	H152 - O156
153	158	1 ;	C153 - H158
153	159	1 ;	C153 - H159
153	160	1 ;	C153 - C160
154	157	1 ;	H154 - C157
155	157	1 ;	H155 - C157
156	161	1 ;	O156 - H161
156	162	1 ;	O156 - H162
156	163	1 ;	O156 - O163
157	164	1 ;	C157 - C164
158	161	1 ;	H158 - H161
158	162	1 ;	H158 - H162
158	163	1 ;	H158 - O163
159	161	1 ;	H159 - H161
159	162	1 ;	H159 - H162
159	163	1 ;	H159 - O163
160	165	1 ;	C160 - H165
160	166	1 ;	C160 - H166
160	167	1 ;	C160 - C167
161	164	1 ;	H161 - C164
162	164	1 ;	H162 - C164
163	168	1 ;	O163 - H168
163	169	1 ;	O163 - H169
163	170	1 ;	O163 - O170

164	171	1 ;	C164 - C171
165	168	1 ;	H165 - H168
165	169	1 ;	H165 - H169
165	170	1 ;	H165 - O170
166	168	1 ;	H166 - H168
166	169	1 ;	H166 - H169
166	170	1 ;	H166 - O170
167	172	1 ;	C167 - H172
167	173	1 ;	C167 - H173
167	174	1 ;	C167 - C174
168	171	1 ;	H168 - C171
169	171	1 ;	H169 - C171
170	175	1 ;	O170 - H175
170	176	1 ;	O170 - H176
170	177	1 ;	O170 - O177
171	178	1 ;	C171 - C178
172	175	1 ;	H172 - H175
172	176	1 ;	H172 - H176
172	177	1 ;	H172 - O177
173	175	1 ;	H173 - H175
173	176	1 ;	H173 - H176
173	177	1 ;	H173 - O177
174	179	1 ;	C174 - H179
174	180	1 ;	C174 - H180
174	181	1 ;	C174 - C181
175	178	1 ;	H175 - C178
176	178	1 ;	H176 - C178
177	182	1 ;	O177 - H182
177	183	1 ;	O177 - H183

177	184	1 ;	O177 - O184
178	185	1 ;	C178 - C185
179	182	1 ;	H179 - H182
179	183	1 ;	H179 - H183
179	184	1 ;	H179 - O184
180	182	1 ;	H180 - H182
180	183	1 ;	H180 - H183
180	184	1 ;	H180 - O184
181	186	1 ;	C181 - H186
181	187	1 ;	C181 - H187
181	188	1 ;	C181 - C188
182	185	1 ;	H182 - C185
183	185	1 ;	H183 - C185
184	189	1 ;	O184 - H189
184	190	1 ;	O184 - H190
184	191	1 ;	O184 - O191
185	192	1 ;	C185 - C192
186	189	1 ;	H186 - H189
186	190	1 ;	H186 - H190
186	191	1 ;	H186 - O191
187	189	1 ;	H187 - H189
187	190	1 ;	H187 - H190
187	191	1 ;	H187 - O191
188	193	1 ;	C188 - H193
188	194	1 ;	C188 - H194
188	195	1 ;	C188 - C195
189	192	1 ;	H189 - C192
190	192	1 ;	H190 - C192
191	196	1 ;	O191 - H196

191	197	1 ;	O191 - H197
191	198	1 ;	O191 - O198
192	199	1 ;	C192 - C199
193	196	1 ;	H193 - H196
193	197	1 ;	H193 - H197
193	198	1 ;	H193 - O198
194	196	1 ;	H194 - H196
194	197	1 ;	H194 - H197
194	198	1 ;	H194 - O198
195	200	1 ;	C195 - H200
195	201	1 ;	C195 - H201
195	202	1 ;	C195 - C202
196	199	1 ;	H196 - C199
197	199	1 ;	H197 - C199
198	203	1 ;	O198 - H203
198	204	1 ;	O198 - H204
198	205	1 ;	O198 - O205
199	206	1 ;	C199 - C206
200	203	1 ;	H200 - H203
200	204	1 ;	H200 - H204
200	205	1 ;	H200 - O205
201	203	1 ;	H201 - H203
201	204	1 ;	H201 - H204
201	205	1 ;	H201 - O205
202	207	1 ;	C202 - H207
202	208	1 ;	C202 - H208
202	209	1 ;	C202 - C209
203	206	1 ;	H203 - C206
204	206	1 ;	H204 - C206

205	210	1 ;	O205 - H210
205	211	1 ;	O205 - H211
205	212	1 ;	O205 - O212
206	213	1 ;	C206 - C213
207	210	1 ;	H207 - H210
207	211	1 ;	H207 - H211
207	212	1 ;	H207 - O212
208	210	1 ;	H208 - H210
208	211	1 ;	H208 - H211
208	212	1 ;	H208 - O212
209	214	1 ;	C209 - H214
209	215	1 ;	C209 - H215
209	216	1 ;	C209 - C216
210	213	1 ;	H210 - C213
211	213	1 ;	H211 - C213
212	217	1 ;	O212 - H217
212	218	1 ;	O212 - H218
212	219	1 ;	O212 - O219
213	220	1 ;	C213 - C220
214	217	1 ;	H214 - H217
214	218	1 ;	H214 - H218
214	219	1 ;	H214 - O219
215	217	1 ;	H215 - H217
215	218	1 ;	H215 - H218
215	219	1 ;	H215 - O219
216	221	1 ;	C216 - H221
216	222	1 ;	C216 - H222
216	223	1 ;	C216 - C223
217	220	1 ;	H217 - C220

218	220	1 ;	H218 - C220
219	224	1 ;	O219 - H224
219	225	1 ;	O219 - H225
219	226	1 ;	O219 - O226
220	227	1 ;	C220 - C227
221	224	1 ;	H221 - H224
221	225	1 ;	H221 - H225
221	226	1 ;	H221 - O226
222	224	1 ;	H222 - H224
222	225	1 ;	H222 - H225
222	226	1 ;	H222 - O226
223	228	1 ;	C223 - H228
223	229	1 ;	C223 - H229
223	230	1 ;	C223 - C230
224	227	1 ;	H224 - C227
225	227	1 ;	H225 - C227
226	231	1 ;	O226 - H231
226	232	1 ;	O226 - H232
226	233	1 ;	O226 - O233
227	234	1 ;	C227 - C234
228	231	1 ;	H228 - H231
228	232	1 ;	H228 - H232
228	233	1 ;	H228 - O233
229	231	1 ;	H229 - H231
229	232	1 ;	H229 - H232
229	233	1 ;	H229 - O233
230	235	1 ;	C230 - H235
230	236	1 ;	C230 - H236
230	237	1 ;	C230 - C237

231	234	1 ;	H231 - C234
232	234	1 ;	H232 - C234
233	238	1 ;	O233 - H238
233	239	1 ;	O233 - H239
233	240	1 ;	O233 - O240
234	241	1 ;	C234 - C241
235	238	1 ;	H235 - H238
235	239	1 ;	H235 - H239
235	240	1 ;	H235 - O240
236	238	1 ;	H236 - H238
236	239	1 ;	H236 - H239
236	240	1 ;	H236 - O240
237	242	1 ;	C237 - H242
237	243	1 ;	C237 - H243
237	244	1 ;	C237 - C244
238	241	1 ;	H238 - C241
239	241	1 ;	H239 - C241
240	245	1 ;	O240 - H245
240	246	1 ;	O240 - H246
240	247	1 ;	O240 - O247
241	248	1 ;	C241 - C248
242	245	1 ;	H242 - H245
242	246	1 ;	H242 - H246
242	247	1 ;	H242 - O247
243	245	1 ;	H243 - H245
243	246	1 ;	H243 - H246
243	247	1 ;	H243 - O247
244	249	1 ;	C244 - H249
244	250	1 ;	C244 - H250

244	251	1 ;	C244 - C251
245	248	1 ;	H245 - C248
246	248	1 ;	H246 - C248
247	252	1 ;	O247 - H252
247	253	1 ;	O247 - H253
247	254	1 ;	O247 - O254
248	255	1 ;	C248 - C255
249	252	1 ;	H249 - H252
249	253	1 ;	H249 - H253
249	254	1 ;	H249 - O254
250	252	1 ;	H250 - H252
250	253	1 ;	H250 - H253
250	254	1 ;	H250 - O254
251	256	1 ;	C251 - H256
251	257	1 ;	C251 - H257
251	258	1 ;	C251 - C258
252	255	1 ;	H252 - C255
253	255	1 ;	H253 - C255
254	259	1 ;	O254 - H259
254	260	1 ;	O254 - H260
254	261	1 ;	O254 - O261
255	262	1 ;	C255 - C262
256	259	1 ;	H256 - H259
256	260	1 ;	H256 - H260
256	261	1 ;	H256 - O261
257	259	1 ;	H257 - H259
257	260	1 ;	H257 - H260
257	261	1 ;	H257 - O261
258	263	1 ;	C258 - H263

258	264	1 ;	C258 - H264
258	265	1 ;	C258 - C265
259	262	1 ;	H259 - C262
260	262	1 ;	H260 - C262
261	266	1 ;	O261 - H266
261	267	1 ;	O261 - H267
261	268	1 ;	O261 - O268
262	269	1 ;	C262 - C269
263	266	1 ;	H263 - H266
263	267	1 ;	H263 - H267
263	268	1 ;	H263 - O268
264	266	1 ;	H264 - H266
264	267	1 ;	H264 - H267
264	268	1 ;	H264 - O268
265	270	1 ;	C265 - H270
265	271	1 ;	C265 - H271
265	272	1 ;	C265 - C272
266	269	1 ;	H266 - C269
267	269	1 ;	H267 - C269
268	273	1 ;	O268 - H273
268	274	1 ;	O268 - H274
268	275	1 ;	O268 - O275
269	276	1 ;	C269 - C276
270	273	1 ;	H270 - H273
270	274	1 ;	H270 - H274
270	275	1 ;	H270 - O275
271	273	1 ;	H271 - H273
271	274	1 ;	H271 - H274
271	275	1 ;	H271 - O275

272	277	1 ;	C272 - H277
272	278	1 ;	C272 - H278
272	279	1 ;	C272 - C279
273	276	1 ;	H273 - C276
274	276	1 ;	H274 - C276
275	280	1 ;	O275 - H280
275	281	1 ;	O275 - H281
275	282	1 ;	O275 - O282
276	283	1 ;	C276 - C283
277	280	1 ;	H277 - H280
277	281	1 ;	H277 - H281
277	282	1 ;	H277 - O282
278	280	1 ;	H278 - H280
278	281	1 ;	H278 - H281
278	282	1 ;	H278 - O282
279	284	1 ;	C279 - H284
279	285	1 ;	C279 - H285
279	286	1 ;	C279 - C286
280	283	1 ;	H280 - C283
281	283	1 ;	H281 - C283
282	287	1 ;	O282 - H287
282	288	1 ;	O282 - H288
282	289	1 ;	O282 - O289
283	290	1 ;	C283 - C290
284	287	1 ;	H284 - H287
284	288	1 ;	H284 - H288
284	289	1 ;	H284 - O289
285	287	1 ;	H285 - H287
285	288	1 ;	H285 - H288

285	289	1 ;	H285 - O289
286	291	1 ;	C286 - H291
286	292	1 ;	C286 - H292
286	293	1 ;	C286 - C293
287	290	1 ;	H287 - C290
288	290	1 ;	H288 - C290
289	294	1 ;	O289 - H294
289	295	1 ;	O289 - H295
289	296	1 ;	O289 - O296
290	297	1 ;	C290 - C297
291	294	1 ;	H291 - H294
291	295	1 ;	H291 - H295
291	296	1 ;	H291 - O296
292	294	1 ;	H292 - H294
292	295	1 ;	H292 - H295
292	296	1 ;	H292 - O296
293	298	1 ;	C293 - H298
293	299	1 ;	C293 - H299
293	300	1 ;	C293 - C300
294	297	1 ;	H294 - C297
295	297	1 ;	H295 - C297
296	301	1 ;	O296 - H301
296	302	1 ;	O296 - H302
296	303	1 ;	O296 - O303
297	304	1 ;	C297 - C304
298	301	1 ;	H298 - H301
298	302	1 ;	H298 - H302
298	303	1 ;	H298 - O303
299	301	1 ;	H299 - H301

299	302	1 ;	H299 - H302
299	303	1 ;	H299 - O303
300	305	1 ;	C300 - H305
300	306	1 ;	C300 - H306
300	307	1 ;	C300 - C307
301	304	1 ;	H301 - C304
302	304	1 ;	H302 - C304
303	308	1 ;	O303 - H308
303	309	1 ;	O303 - H309
303	310	1 ;	O303 - O310
304	311	1 ;	C304 - C311
305	308	1 ;	H305 - H308
305	309	1 ;	H305 - H309
305	310	1 ;	H305 - O310
306	308	1 ;	H306 - H308
306	309	1 ;	H306 - H309
306	310	1 ;	H306 - O310
307	312	1 ;	C307 - H312
307	313	1 ;	C307 - H313
307	314	1 ;	C307 - C314
308	311	1 ;	H308 - C311
309	311	1 ;	H309 - C311
310	315	1 ;	O310 - H315
310	316	1 ;	O310 - H316
310	317	1 ;	O310 - O317
311	318	1 ;	C311 - H318
312	315	1 ;	H312 - H315
312	316	1 ;	H312 - H316
312	317	1 ;	H312 - O317

313 315 1 ; H313 - H315

313 316 1 ; H313 - H316

313 317 1 ; H313 - O317

315 318 1 ; H315 - H318

316 318 1 ; H316 - H318

[angles]

; ai aj ak funct theta cth

1 3 4 1 1.1038e+02 4.7103e+02 ; O1 - C3 - H4

1 3 5 1 1.1038e+02 4.7103e+02 ; O1 - C3 - H5

1 3 6 1 1.0966e+02 6.4258e+02 ; O1 - C3 - C6

2 1 3 1 1.0739e+02 5.4501e+02 ; H2 - O1 - C3

3 6 7 1 1.0959e+02 3.6217e+02 ; C3 - C6 - H7

3 6 8 1 1.0959e+02 3.6217e+02 ; C3 - C6 - H8

3 6 9 1 1.0762e+02 6.4802e+02 ; C3 - C6 - O9

4 3 5 1 1.0855e+02 2.9824e+02 ; H4 - C3 - H5

4 3 6 1 1.0959e+02 3.6217e+02 ; H4 - C3 - C6

5 3 6 1 1.0959e+02 3.6217e+02 ; H5 - C3 - C6

6 9 10 1 1.1360e+02 7.3915e+02 ; C6 - O9 - C10

7 6 8 1 1.0855e+02 2.9824e+02 ; H7 - C6 - H8

7 6 9 1 1.1034e+02 4.7028e+02 ; H7 - C6 - O9

8 6 9 1 1.1034e+02 4.7028e+02 ; H8 - C6 - O9

9 10 11 1 1.1034e+02 4.7028e+02 ; O9 - C10 - H11

9 10 12 1 1.1034e+02 4.7028e+02 ; O9 - C10 - H12

9 10 13 1 1.0762e+02 6.4802e+02 ; O9 - C10 - C13

10 13 14 1 1.0959e+02 3.6217e+02 ; C10 - C13 - H14

10 13 15 1 1.0959e+02 3.6217e+02 ; C10 - C13 - H15

10 13 16 1 1.0762e+02 6.4802e+02 ; C10 - C13 - O16

11 10 12 1 1.0855e+02 2.9824e+02 ; H11 - C10 - H12

11	10	13	1	1.0959e+02	3.6217e+02 ;	H11 - C10	- C13
12	10	13	1	1.0959e+02	3.6217e+02 ;	H12 - C10	- C13
13	16	17	1	1.1360e+02	7.3915e+02 ;	C13 - O16	- C17
14	13	15	1	1.0855e+02	2.9824e+02 ;	H14 - C13	- H15
14	13	16	1	1.1034e+02	4.7028e+02 ;	H14 - C13	- O16
15	13	16	1	1.1034e+02	4.7028e+02 ;	H15 - C13	- O16
16	17	18	1	1.1034e+02	4.7028e+02 ;	O16 - C17	- H18
16	17	19	1	1.1034e+02	4.7028e+02 ;	O16 - C17	- H19
16	17	20	1	1.0762e+02	6.4802e+02 ;	O16 - C17	- C20
17	20	21	1	1.0959e+02	3.6217e+02 ;	C17 - C20	- H21
17	20	22	1	1.0959e+02	3.6217e+02 ;	C17 - C20	- H22
17	20	23	1	1.0762e+02	6.4802e+02 ;	C17 - C20	- O23
18	17	19	1	1.0855e+02	2.9824e+02 ;	H18 - C17	- H19
18	17	20	1	1.0959e+02	3.6217e+02 ;	H18 - C17	- C20
19	17	20	1	1.0959e+02	3.6217e+02 ;	H19 - C17	- C20
20	23	24	1	1.1360e+02	7.3915e+02 ;	C20 - O23	- C24
21	20	22	1	1.0855e+02	2.9824e+02 ;	H21 - C20	- H22
21	20	23	1	1.1034e+02	4.7028e+02 ;	H21 - C20	- O23
22	20	23	1	1.1034e+02	4.7028e+02 ;	H22 - C20	- O23
23	24	25	1	1.1034e+02	4.7028e+02 ;	O23 - C24	- H25
23	24	26	1	1.1034e+02	4.7028e+02 ;	O23 - C24	- H26
23	24	27	1	1.0762e+02	6.4802e+02 ;	O23 - C24	- C27
24	27	28	1	1.0959e+02	3.6217e+02 ;	C24 - C27	- H28
24	27	29	1	1.0959e+02	3.6217e+02 ;	C24 - C27	- H29
24	27	30	1	1.0762e+02	6.4802e+02 ;	C24 - C27	- O30
25	24	26	1	1.0855e+02	2.9824e+02 ;	H25 - C24	- H26
25	24	27	1	1.0959e+02	3.6217e+02 ;	H25 - C24	- C27
26	24	27	1	1.0959e+02	3.6217e+02 ;	H26 - C24	- C27
27	30	31	1	1.1360e+02	7.3915e+02 ;	C27 - O30	- C31

28	27	29	1	1.0855e+02	2.9824e+02 ;	H28 - C27	- H29
28	27	30	1	1.1034e+02	4.7028e+02 ;	H28 - C27	- O30
29	27	30	1	1.1034e+02	4.7028e+02 ;	H29 - C27	- O30
30	31	32	1	1.1034e+02	4.7028e+02 ;	O30 - C31	- H32
30	31	33	1	1.1034e+02	4.7028e+02 ;	O30 - C31	- H33
30	31	34	1	1.0762e+02	6.4802e+02 ;	O30 - C31	- C34
31	34	35	1	1.0959e+02	3.6217e+02 ;	C31 - C34	- H35
31	34	36	1	1.0959e+02	3.6217e+02 ;	C31 - C34	- H36
31	34	37	1	1.0762e+02	6.4802e+02 ;	C31 - C34	- O37
32	31	33	1	1.0855e+02	2.9824e+02 ;	H32 - C31	- H33
32	31	34	1	1.0959e+02	3.6217e+02 ;	H32 - C31	- C34
33	31	34	1	1.0959e+02	3.6217e+02 ;	H33 - C31	- C34
34	37	38	1	1.1360e+02	7.3915e+02 ;	C34 - O37	- C38
35	34	36	1	1.0855e+02	2.9824e+02 ;	H35 - C34	- H36
35	34	37	1	1.1034e+02	4.7028e+02 ;	H35 - C34	- O37
36	34	37	1	1.1034e+02	4.7028e+02 ;	H36 - C34	- O37
37	38	39	1	1.1034e+02	4.7028e+02 ;	O37 - C38	- H39
37	38	40	1	1.1034e+02	4.7028e+02 ;	O37 - C38	- H40
37	38	41	1	1.0762e+02	6.4802e+02 ;	O37 - C38	- C41
38	41	42	1	1.0959e+02	3.6217e+02 ;	C38 - C41	- H42
38	41	43	1	1.0959e+02	3.6217e+02 ;	C38 - C41	- H43
38	41	44	1	1.0762e+02	6.4802e+02 ;	C38 - C41	- O44
39	38	40	1	1.0855e+02	2.9824e+02 ;	H39 - C38	- H40
39	38	41	1	1.0959e+02	3.6217e+02 ;	H39 - C38	- C41
40	38	41	1	1.0959e+02	3.6217e+02 ;	H40 - C38	- C41
41	44	45	1	1.1360e+02	7.3915e+02 ;	C41 - O44	- C45
42	41	43	1	1.0855e+02	2.9824e+02 ;	H42 - C41	- H43
42	41	44	1	1.1034e+02	4.7028e+02 ;	H42 - C41	- O44
43	41	44	1	1.1034e+02	4.7028e+02 ;	H43 - C41	- O44

44	45	46	1	1.1034e+02	4.7028e+02 ;	O44 - C45	- H46
44	45	47	1	1.1034e+02	4.7028e+02 ;	O44 - C45	- H47
44	45	48	1	1.0762e+02	6.4802e+02 ;	O44 - C45	- C48
45	48	49	1	1.0959e+02	3.6217e+02 ;	C45 - C48	- H49
45	48	50	1	1.0959e+02	3.6217e+02 ;	C45 - C48	- H50
45	48	51	1	1.0762e+02	6.4802e+02 ;	C45 - C48	- O51
46	45	47	1	1.0855e+02	2.9824e+02 ;	H46 - C45	- H47
46	45	48	1	1.0959e+02	3.6217e+02 ;	H46 - C45	- C48
47	45	48	1	1.0959e+02	3.6217e+02 ;	H47 - C45	- C48
48	51	52	1	1.1360e+02	7.3915e+02 ;	C48 - O51	- C52
49	48	50	1	1.0855e+02	2.9824e+02 ;	H49 - C48	- H50
49	48	51	1	1.1034e+02	4.7028e+02 ;	H49 - C48	- O51
50	48	51	1	1.1034e+02	4.7028e+02 ;	H50 - C48	- O51
51	52	53	1	1.1034e+02	4.7028e+02 ;	O51 - C52	- H53
51	52	54	1	1.1034e+02	4.7028e+02 ;	O51 - C52	- H54
51	52	55	1	1.0762e+02	6.4802e+02 ;	O51 - C52	- C55
52	55	56	1	1.0959e+02	3.6217e+02 ;	C52 - C55	- H56
52	55	57	1	1.0959e+02	3.6217e+02 ;	C52 - C55	- H57
52	55	58	1	1.0762e+02	6.4802e+02 ;	C52 - C55	- O58
53	52	54	1	1.0855e+02	2.9824e+02 ;	H53 - C52	- H54
53	52	55	1	1.0959e+02	3.6217e+02 ;	H53 - C52	- C55
54	52	55	1	1.0959e+02	3.6217e+02 ;	H54 - C52	- C55
55	58	59	1	1.1360e+02	7.3915e+02 ;	C55 - O58	- C59
56	55	57	1	1.0855e+02	2.9824e+02 ;	H56 - C55	- H57
56	55	58	1	1.1034e+02	4.7028e+02 ;	H56 - C55	- O58
57	55	58	1	1.1034e+02	4.7028e+02 ;	H57 - C55	- O58
58	59	60	1	1.1034e+02	4.7028e+02 ;	O58 - C59	- H60
58	59	61	1	1.1034e+02	4.7028e+02 ;	O58 - C59	- H61
58	59	62	1	1.0762e+02	6.4802e+02 ;	O58 - C59	- C62

59	62	63	1	1.0959e+02	3.6217e+02 ;	C59 - C62	- H63
59	62	64	1	1.0959e+02	3.6217e+02 ;	C59 - C62	- H64
59	62	65	1	1.0762e+02	6.4802e+02 ;	C59 - C62	- O65
60	59	61	1	1.0855e+02	2.9824e+02 ;	H60 - C59	- H61
60	59	62	1	1.0959e+02	3.6217e+02 ;	H60 - C59	- C62
61	59	62	1	1.0959e+02	3.6217e+02 ;	H61 - C59	- C62
62	65	66	1	1.1360e+02	7.3915e+02 ;	C62 - O65	- C66
63	62	64	1	1.0855e+02	2.9824e+02 ;	H63 - C62	- H64
63	62	65	1	1.1034e+02	4.7028e+02 ;	H63 - C62	- O65
64	62	65	1	1.1034e+02	4.7028e+02 ;	H64 - C62	- O65
65	66	67	1	1.1034e+02	4.7028e+02 ;	O65 - C66	- H67
65	66	68	1	1.1034e+02	4.7028e+02 ;	O65 - C66	- H68
65	66	69	1	1.0762e+02	6.4802e+02 ;	O65 - C66	- C69
66	69	70	1	1.0959e+02	3.6217e+02 ;	C66 - C69	- H70
66	69	71	1	1.0959e+02	3.6217e+02 ;	C66 - C69	- H71
66	69	72	1	1.0762e+02	6.4802e+02 ;	C66 - C69	- O72
67	66	68	1	1.0855e+02	2.9824e+02 ;	H67 - C66	- H68
67	66	69	1	1.0959e+02	3.6217e+02 ;	H67 - C66	- C69
68	66	69	1	1.0959e+02	3.6217e+02 ;	H68 - C66	- C69
69	72	73	1	1.1360e+02	7.3915e+02 ;	C69 - O72	- C73
70	69	71	1	1.0855e+02	2.9824e+02 ;	H70 - C69	- H71
70	69	72	1	1.1034e+02	4.7028e+02 ;	H70 - C69	- O72
71	69	72	1	1.1034e+02	4.7028e+02 ;	H71 - C69	- O72
72	73	74	1	1.1034e+02	4.7028e+02 ;	O72 - C73	- H74
72	73	75	1	1.1034e+02	4.7028e+02 ;	O72 - C73	- H75
72	73	76	1	1.0762e+02	6.4802e+02 ;	O72 - C73	- C76
73	76	77	1	1.0959e+02	3.6217e+02 ;	C73 - C76	- H77
73	76	78	1	1.0959e+02	3.6217e+02 ;	C73 - C76	- H78
73	76	79	1	1.0762e+02	6.4802e+02 ;	C73 - C76	- O79

74	73	75	1	1.0855e+02	2.9824e+02 ;	H74 - C73	- H75
74	73	76	1	1.0959e+02	3.6217e+02 ;	H74 - C73	- C76
75	73	76	1	1.0959e+02	3.6217e+02 ;	H75 - C73	- C76
76	79	80	1	1.1360e+02	7.3915e+02 ;	C76 - O79	- C80
77	76	78	1	1.0855e+02	2.9824e+02 ;	H77 - C76	- H78
77	76	79	1	1.1034e+02	4.7028e+02 ;	H77 - C76	- O79
78	76	79	1	1.1034e+02	4.7028e+02 ;	H78 - C76	- O79
79	80	81	1	1.1034e+02	4.7028e+02 ;	O79 - C80	- H81
79	80	82	1	1.1034e+02	4.7028e+02 ;	O79 - C80	- H82
79	80	83	1	1.0762e+02	6.4802e+02 ;	O79 - C80	- C83
80	83	84	1	1.0959e+02	3.6217e+02 ;	C80 - C83	- H84
80	83	85	1	1.0959e+02	3.6217e+02 ;	C80 - C83	- H85
80	83	86	1	1.0762e+02	6.4802e+02 ;	C80 - C83	- O86
81	80	82	1	1.0855e+02	2.9824e+02 ;	H81 - C80	- H82
81	80	83	1	1.0959e+02	3.6217e+02 ;	H81 - C80	- C83
82	80	83	1	1.0959e+02	3.6217e+02 ;	H82 - C80	- C83
83	86	87	1	1.1360e+02	7.3915e+02 ;	C83 - O86	- C87
84	83	85	1	1.0855e+02	2.9824e+02 ;	H84 - C83	- H85
84	83	86	1	1.1034e+02	4.7028e+02 ;	H84 - C83	- O86
85	83	86	1	1.1034e+02	4.7028e+02 ;	H85 - C83	- O86
86	87	88	1	1.1034e+02	4.7028e+02 ;	O86 - C87	- H88
86	87	89	1	1.1034e+02	4.7028e+02 ;	O86 - C87	- H89
86	87	90	1	1.0762e+02	6.4802e+02 ;	O86 - C87	- C90
87	90	91	1	1.0959e+02	3.6217e+02 ;	C87 - C90	- H91
87	90	92	1	1.0959e+02	3.6217e+02 ;	C87 - C90	- H92
87	90	93	1	1.0762e+02	6.4802e+02 ;	C87 - C90	- O93
88	87	89	1	1.0855e+02	2.9824e+02 ;	H88 - C87	- H89
88	87	90	1	1.0959e+02	3.6217e+02 ;	H88 - C87	- C90
89	87	90	1	1.0959e+02	3.6217e+02 ;	H89 - C87	- C90

90	93	94	1	1.1360e+02	7.3915e+02 ;	C90 - O93	- C94
91	90	92	1	1.0855e+02	2.9824e+02 ;	H91 - C90	- H92
91	90	93	1	1.1034e+02	4.7028e+02 ;	H91 - C90	- O93
92	90	93	1	1.1034e+02	4.7028e+02 ;	H92 - C90	- O93
93	94	95	1	1.1034e+02	4.7028e+02 ;	O93 - C94	- H95
93	94	96	1	1.1034e+02	4.7028e+02 ;	O93 - C94	- H96
93	94	97	1	1.0762e+02	6.4802e+02 ;	O93 - C94	- C97
94	97	98	1	1.0959e+02	3.6217e+02 ;	C94 - C97	- H98
94	97	99	1	1.0959e+02	3.6217e+02 ;	C94 - C97	- H99
94	97	100	1	1.0762e+02	6.4802e+02 ;	C94 - C97	- O100
95	94	96	1	1.0855e+02	2.9824e+02 ;	H95 - C94	- H96
95	94	97	1	1.0959e+02	3.6217e+02 ;	H95 - C94	- C97
96	94	97	1	1.0959e+02	3.6217e+02 ;	H96 - C94	- C97
97	100	101	1	1.1360e+02	7.3915e+02 ;	C97 - O100	- C101
98	97	99	1	1.0855e+02	2.9824e+02 ;	H98 - C97	- H99
98	97	100	1	1.1034e+02	4.7028e+02 ;	H98 - C97	- O100
99	97	100	1	1.1034e+02	4.7028e+02 ;	H99 - C97	- O100
100	101	102	1	1.1034e+02	4.7028e+02 ;	O100 - C101	- H102
100	101	103	1	1.1034e+02	4.7028e+02 ;	O100 - C101	- H103
100	101	104	1	1.0762e+02	6.4802e+02 ;	O100 - C101	- C104
101	104	105	1	1.0959e+02	3.6217e+02 ;	C101 - C104	- H105
101	104	106	1	1.0959e+02	3.6217e+02 ;	C101 - C104	- H106
101	104	107	1	1.0762e+02	6.4802e+02 ;	C101 - C104	- O107
102	101	103	1	1.0855e+02	2.9824e+02 ;	H102 - C101	- H103
102	101	104	1	1.0959e+02	3.6217e+02 ;	H102 - C101	- C104
103	101	104	1	1.0959e+02	3.6217e+02 ;	H103 - C101	- C104
104	107	108	1	1.1360e+02	7.3915e+02 ;	C104 - O107	- C108
105	104	106	1	1.0855e+02	2.9824e+02 ;	H105 - C104	- H106
105	104	107	1	1.1034e+02	4.7028e+02 ;	H105 - C104	- O107

106	104	107	1	1.1034e+02	4.7028e+02 ;	H106 - C104 - O107
107	108	109	1	1.1034e+02	4.7028e+02 ;	O107 - C108 - H109
107	108	110	1	1.1034e+02	4.7028e+02 ;	O107 - C108 - H110
107	108	111	1	1.0762e+02	6.4802e+02 ;	O107 - C108 - C111
108	111	112	1	1.0959e+02	3.6217e+02 ;	C108 - C111 - H112
108	111	113	1	1.0959e+02	3.6217e+02 ;	C108 - C111 - H113
108	111	114	1	1.0762e+02	6.4802e+02 ;	C108 - C111 - O114
109	108	110	1	1.0855e+02	2.9824e+02 ;	H109 - C108 - H110
109	108	111	1	1.0959e+02	3.6217e+02 ;	H109 - C108 - C111
110	108	111	1	1.0959e+02	3.6217e+02 ;	H110 - C108 - C111
111	114	115	1	1.1360e+02	7.3915e+02 ;	C111 - O114 - C115
112	111	113	1	1.0855e+02	2.9824e+02 ;	H112 - C111 - H113
112	111	114	1	1.1034e+02	4.7028e+02 ;	H112 - C111 - O114
113	111	114	1	1.1034e+02	4.7028e+02 ;	H113 - C111 - O114
114	115	116	1	1.1034e+02	4.7028e+02 ;	O114 - C115 - H116
114	115	117	1	1.1034e+02	4.7028e+02 ;	O114 - C115 - H117
114	115	118	1	1.0762e+02	6.4802e+02 ;	O114 - C115 - C118
115	118	119	1	1.0959e+02	3.6217e+02 ;	C115 - C118 - H119
115	118	120	1	1.0959e+02	3.6217e+02 ;	C115 - C118 - H120
115	118	121	1	1.0762e+02	6.4802e+02 ;	C115 - C118 - O121
116	115	117	1	1.0855e+02	2.9824e+02 ;	H116 - C115 - H117
116	115	118	1	1.0959e+02	3.6217e+02 ;	H116 - C115 - C118
117	115	118	1	1.0959e+02	3.6217e+02 ;	H117 - C115 - C118
118	121	122	1	1.1360e+02	7.3915e+02 ;	C118 - O121 - C122
119	118	120	1	1.0855e+02	2.9824e+02 ;	H119 - C118 - H120
119	118	121	1	1.1034e+02	4.7028e+02 ;	H119 - C118 - O121
120	118	121	1	1.1034e+02	4.7028e+02 ;	H120 - C118 - O121
121	122	123	1	1.1034e+02	4.7028e+02 ;	O121 - C122 - H123
121	122	124	1	1.1034e+02	4.7028e+02 ;	O121 - C122 - H124

121	122	125	1	1.0762e+02	6.4802e+02 ;	O121 - C122	- C125
122	125	126	1	1.0959e+02	3.6217e+02 ;	C122 - C125	- H126
122	125	127	1	1.0959e+02	3.6217e+02 ;	C122 - C125	- H127
122	125	128	1	1.0762e+02	6.4802e+02 ;	C122 - C125	- O128
123	122	124	1	1.0855e+02	2.9824e+02 ;	H123 - C122	- H124
123	122	125	1	1.0959e+02	3.6217e+02 ;	H123 - C122	- C125
124	122	125	1	1.0959e+02	3.6217e+02 ;	H124 - C122	- C125
125	128	129	1	1.1360e+02	7.3915e+02 ;	C125 - O128	- C129
126	125	127	1	1.0855e+02	2.9824e+02 ;	H126 - C125	- H127
126	125	128	1	1.1034e+02	4.7028e+02 ;	H126 - C125	- O128
127	125	128	1	1.1034e+02	4.7028e+02 ;	H127 - C125	- O128
128	129	130	1	1.1034e+02	4.7028e+02 ;	O128 - C129	- H130
128	129	131	1	1.1034e+02	4.7028e+02 ;	O128 - C129	- H131
128	129	132	1	1.0762e+02	6.4802e+02 ;	O128 - C129	- C132
129	132	133	1	1.0959e+02	3.6217e+02 ;	C129 - C132	- H133
129	132	134	1	1.0959e+02	3.6217e+02 ;	C129 - C132	- H134
129	132	135	1	1.0762e+02	6.4802e+02 ;	C129 - C132	- O135
130	129	131	1	1.0855e+02	2.9824e+02 ;	H130 - C129	- H131
130	129	132	1	1.0959e+02	3.6217e+02 ;	H130 - C129	- C132
131	129	132	1	1.0959e+02	3.6217e+02 ;	H131 - C129	- C132
132	135	136	1	1.1360e+02	7.3915e+02 ;	C132 - O135	- C136
133	132	134	1	1.0855e+02	2.9824e+02 ;	H133 - C132	- H134
133	132	135	1	1.1034e+02	4.7028e+02 ;	H133 - C132	- O135
134	132	135	1	1.1034e+02	4.7028e+02 ;	H134 - C132	- O135
135	136	137	1	1.1034e+02	4.7028e+02 ;	O135 - C136	- H137
135	136	138	1	1.1034e+02	4.7028e+02 ;	O135 - C136	- H138
135	136	139	1	1.0762e+02	6.4802e+02 ;	O135 - C136	- C139
136	139	140	1	1.0959e+02	3.6217e+02 ;	C136 - C139	- H140
136	139	141	1	1.0959e+02	3.6217e+02 ;	C136 - C139	- H141

136	139	142	1	1.0762e+02	6.4802e+02 ;	C136	-	C139	-	O142
137	136	138	1	1.0855e+02	2.9824e+02 ;	H137	-	C136	-	H138
137	136	139	1	1.0959e+02	3.6217e+02 ;	H137	-	C136	-	C139
138	136	139	1	1.0959e+02	3.6217e+02 ;	H138	-	C136	-	C139
139	142	143	1	1.1360e+02	7.3915e+02 ;	C139	-	O142	-	C143
140	139	141	1	1.0855e+02	2.9824e+02 ;	H140	-	C139	-	H141
140	139	142	1	1.1034e+02	4.7028e+02 ;	H140	-	C139	-	O142
141	139	142	1	1.1034e+02	4.7028e+02 ;	H141	-	C139	-	O142
142	143	144	1	1.1034e+02	4.7028e+02 ;	O142	-	C143	-	H144
142	143	145	1	1.1034e+02	4.7028e+02 ;	O142	-	C143	-	H145
142	143	146	1	1.0762e+02	6.4802e+02 ;	O142	-	C143	-	C146
143	146	147	1	1.0959e+02	3.6217e+02 ;	C143	-	C146	-	H147
143	146	148	1	1.0959e+02	3.6217e+02 ;	C143	-	C146	-	H148
143	146	149	1	1.0762e+02	6.4802e+02 ;	C143	-	C146	-	O149
144	143	145	1	1.0855e+02	2.9824e+02 ;	H144	-	C143	-	H145
144	143	146	1	1.0959e+02	3.6217e+02 ;	H144	-	C143	-	C146
145	143	146	1	1.0959e+02	3.6217e+02 ;	H145	-	C143	-	C146
146	149	150	1	1.1360e+02	7.3915e+02 ;	C146	-	O149	-	C150
147	146	148	1	1.0855e+02	2.9824e+02 ;	H147	-	C146	-	H148
147	146	149	1	1.1034e+02	4.7028e+02 ;	H147	-	C146	-	O149
148	146	149	1	1.1034e+02	4.7028e+02 ;	H148	-	C146	-	O149
149	150	151	1	1.1034e+02	4.7028e+02 ;	O149	-	C150	-	H151
149	150	152	1	1.1034e+02	4.7028e+02 ;	O149	-	C150	-	H152
149	150	153	1	1.0762e+02	6.4802e+02 ;	O149	-	C150	-	C153
150	153	154	1	1.0959e+02	3.6217e+02 ;	C150	-	C153	-	H154
150	153	155	1	1.0959e+02	3.6217e+02 ;	C150	-	C153	-	H155
150	153	156	1	1.0762e+02	6.4802e+02 ;	C150	-	C153	-	O156
151	150	152	1	1.0855e+02	2.9824e+02 ;	H151	-	C150	-	H152
151	150	153	1	1.0959e+02	3.6217e+02 ;	H151	-	C150	-	C153

152	150	153	1	1.0959e+02	3.6217e+02 ;	H152 - C150 - C153
153	156	157	1	1.1360e+02	7.3915e+02 ;	C153 - O156 - C157
154	153	155	1	1.0855e+02	2.9824e+02 ;	H154 - C153 - H155
154	153	156	1	1.1034e+02	4.7028e+02 ;	H154 - C153 - O156
155	153	156	1	1.1034e+02	4.7028e+02 ;	H155 - C153 - O156
156	157	158	1	1.1034e+02	4.7028e+02 ;	O156 - C157 - H158
156	157	159	1	1.1034e+02	4.7028e+02 ;	O156 - C157 - H159
156	157	160	1	1.0762e+02	6.4802e+02 ;	O156 - C157 - C160
157	160	161	1	1.0959e+02	3.6217e+02 ;	C157 - C160 - H161
157	160	162	1	1.0959e+02	3.6217e+02 ;	C157 - C160 - H162
157	160	163	1	1.0762e+02	6.4802e+02 ;	C157 - C160 - O163
158	157	159	1	1.0855e+02	2.9824e+02 ;	H158 - C157 - H159
158	157	160	1	1.0959e+02	3.6217e+02 ;	H158 - C157 - C160
159	157	160	1	1.0959e+02	3.6217e+02 ;	H159 - C157 - C160
160	163	164	1	1.1360e+02	7.3915e+02 ;	C160 - O163 - C164
161	160	162	1	1.0855e+02	2.9824e+02 ;	H161 - C160 - H162
161	160	163	1	1.1034e+02	4.7028e+02 ;	H161 - C160 - O163
162	160	163	1	1.1034e+02	4.7028e+02 ;	H162 - C160 - O163
163	164	165	1	1.1034e+02	4.7028e+02 ;	O163 - C164 - H165
163	164	166	1	1.1034e+02	4.7028e+02 ;	O163 - C164 - H166
163	164	167	1	1.0762e+02	6.4802e+02 ;	O163 - C164 - C167
164	167	168	1	1.0959e+02	3.6217e+02 ;	C164 - C167 - H168
164	167	169	1	1.0959e+02	3.6217e+02 ;	C164 - C167 - H169
164	167	170	1	1.0762e+02	6.4802e+02 ;	C164 - C167 - O170
165	164	166	1	1.0855e+02	2.9824e+02 ;	H165 - C164 - H166
165	164	167	1	1.0959e+02	3.6217e+02 ;	H165 - C164 - C167
166	164	167	1	1.0959e+02	3.6217e+02 ;	H166 - C164 - C167
167	170	171	1	1.1360e+02	7.3915e+02 ;	C167 - O170 - C171
168	167	169	1	1.0855e+02	2.9824e+02 ;	H168 - C167 - H169

168	167	170	1	1.1034e+02	4.7028e+02 ;	H168 - C167 - O170
169	167	170	1	1.1034e+02	4.7028e+02 ;	H169 - C167 - O170
170	171	172	1	1.1034e+02	4.7028e+02 ;	O170 - C171 - H172
170	171	173	1	1.1034e+02	4.7028e+02 ;	O170 - C171 - H173
170	171	174	1	1.0762e+02	6.4802e+02 ;	O170 - C171 - C174
171	174	175	1	1.0959e+02	3.6217e+02 ;	C171 - C174 - H175
171	174	176	1	1.0959e+02	3.6217e+02 ;	C171 - C174 - H176
171	174	177	1	1.0762e+02	6.4802e+02 ;	C171 - C174 - O177
172	171	173	1	1.0855e+02	2.9824e+02 ;	H172 - C171 - H173
172	171	174	1	1.0959e+02	3.6217e+02 ;	H172 - C171 - C174
173	171	174	1	1.0959e+02	3.6217e+02 ;	H173 - C171 - C174
174	177	178	1	1.1360e+02	7.3915e+02 ;	C174 - O177 - C178
175	174	176	1	1.0855e+02	2.9824e+02 ;	H175 - C174 - H176
175	174	177	1	1.1034e+02	4.7028e+02 ;	H175 - C174 - O177
176	174	177	1	1.1034e+02	4.7028e+02 ;	H176 - C174 - O177
177	178	179	1	1.1034e+02	4.7028e+02 ;	O177 - C178 - H179
177	178	180	1	1.1034e+02	4.7028e+02 ;	O177 - C178 - H180
177	178	181	1	1.0762e+02	6.4802e+02 ;	O177 - C178 - C181
178	181	182	1	1.0959e+02	3.6217e+02 ;	C178 - C181 - H182
178	181	183	1	1.0959e+02	3.6217e+02 ;	C178 - C181 - H183
178	181	184	1	1.0762e+02	6.4802e+02 ;	C178 - C181 - O184
179	178	180	1	1.0855e+02	2.9824e+02 ;	H179 - C178 - H180
179	178	181	1	1.0959e+02	3.6217e+02 ;	H179 - C178 - C181
180	178	181	1	1.0959e+02	3.6217e+02 ;	H180 - C178 - C181
181	184	185	1	1.1360e+02	7.3915e+02 ;	C181 - O184 - C185
182	181	183	1	1.0855e+02	2.9824e+02 ;	H182 - C181 - H183
182	181	184	1	1.1034e+02	4.7028e+02 ;	H182 - C181 - O184
183	181	184	1	1.1034e+02	4.7028e+02 ;	H183 - C181 - O184
184	185	186	1	1.1034e+02	4.7028e+02 ;	O184 - C185 - H186

184	185	187	1	1.1034e+02	4.7028e+02 ;	O184 - C185 - H187
184	185	188	1	1.0762e+02	6.4802e+02 ;	O184 - C185 - C188
185	188	189	1	1.0959e+02	3.6217e+02 ;	C185 - C188 - H189
185	188	190	1	1.0959e+02	3.6217e+02 ;	C185 - C188 - H190
185	188	191	1	1.0762e+02	6.4802e+02 ;	C185 - C188 - O191
186	185	187	1	1.0855e+02	2.9824e+02 ;	H186 - C185 - H187
186	185	188	1	1.0959e+02	3.6217e+02 ;	H186 - C185 - C188
187	185	188	1	1.0959e+02	3.6217e+02 ;	H187 - C185 - C188
188	191	192	1	1.1360e+02	7.3915e+02 ;	C188 - O191 - C192
189	188	190	1	1.0855e+02	2.9824e+02 ;	H189 - C188 - H190
189	188	191	1	1.1034e+02	4.7028e+02 ;	H189 - C188 - O191
190	188	191	1	1.1034e+02	4.7028e+02 ;	H190 - C188 - O191
191	192	193	1	1.1034e+02	4.7028e+02 ;	O191 - C192 - H193
191	192	194	1	1.1034e+02	4.7028e+02 ;	O191 - C192 - H194
191	192	195	1	1.0762e+02	6.4802e+02 ;	O191 - C192 - C195
192	195	196	1	1.0959e+02	3.6217e+02 ;	C192 - C195 - H196
192	195	197	1	1.0959e+02	3.6217e+02 ;	C192 - C195 - H197
192	195	198	1	1.0762e+02	6.4802e+02 ;	C192 - C195 - O198
193	192	194	1	1.0855e+02	2.9824e+02 ;	H193 - C192 - H194
193	192	195	1	1.0959e+02	3.6217e+02 ;	H193 - C192 - C195
194	192	195	1	1.0959e+02	3.6217e+02 ;	H194 - C192 - C195
195	198	199	1	1.1360e+02	7.3915e+02 ;	C195 - O198 - C199
196	195	197	1	1.0855e+02	2.9824e+02 ;	H196 - C195 - H197
196	195	198	1	1.1034e+02	4.7028e+02 ;	H196 - C195 - O198
197	195	198	1	1.1034e+02	4.7028e+02 ;	H197 - C195 - O198
198	199	200	1	1.1034e+02	4.7028e+02 ;	O198 - C199 - H200
198	199	201	1	1.1034e+02	4.7028e+02 ;	O198 - C199 - H201
198	199	202	1	1.0762e+02	6.4802e+02 ;	O198 - C199 - C202
199	202	203	1	1.0959e+02	3.6217e+02 ;	C199 - C202 - H203

199	202	204	1	1.0959e+02	3.6217e+02 ;	C199 - C202 - H204
199	202	205	1	1.0762e+02	6.4802e+02 ;	C199 - C202 - O205
200	199	201	1	1.0855e+02	2.9824e+02 ;	H200 - C199 - H201
200	199	202	1	1.0959e+02	3.6217e+02 ;	H200 - C199 - C202
201	199	202	1	1.0959e+02	3.6217e+02 ;	H201 - C199 - C202
202	205	206	1	1.1360e+02	7.3915e+02 ;	C202 - O205 - C206
203	202	204	1	1.0855e+02	2.9824e+02 ;	H203 - C202 - H204
203	202	205	1	1.1034e+02	4.7028e+02 ;	H203 - C202 - O205
204	202	205	1	1.1034e+02	4.7028e+02 ;	H204 - C202 - O205
205	206	207	1	1.1034e+02	4.7028e+02 ;	O205 - C206 - H207
205	206	208	1	1.1034e+02	4.7028e+02 ;	O205 - C206 - H208
205	206	209	1	1.0762e+02	6.4802e+02 ;	O205 - C206 - C209
206	209	210	1	1.0959e+02	3.6217e+02 ;	C206 - C209 - H210
206	209	211	1	1.0959e+02	3.6217e+02 ;	C206 - C209 - H211
206	209	212	1	1.0762e+02	6.4802e+02 ;	C206 - C209 - O212
207	206	208	1	1.0855e+02	2.9824e+02 ;	H207 - C206 - H208
207	206	209	1	1.0959e+02	3.6217e+02 ;	H207 - C206 - C209
208	206	209	1	1.0959e+02	3.6217e+02 ;	H208 - C206 - C209
209	212	213	1	1.1360e+02	7.3915e+02 ;	C209 - O212 - C213
210	209	211	1	1.0855e+02	2.9824e+02 ;	H210 - C209 - H211
210	209	212	1	1.1034e+02	4.7028e+02 ;	H210 - C209 - O212
211	209	212	1	1.1034e+02	4.7028e+02 ;	H211 - C209 - O212
212	213	214	1	1.1034e+02	4.7028e+02 ;	O212 - C213 - H214
212	213	215	1	1.1034e+02	4.7028e+02 ;	O212 - C213 - H215
212	213	216	1	1.0762e+02	6.4802e+02 ;	O212 - C213 - C216
213	216	217	1	1.0959e+02	3.6217e+02 ;	C213 - C216 - H217
213	216	218	1	1.0959e+02	3.6217e+02 ;	C213 - C216 - H218
213	216	219	1	1.0762e+02	6.4802e+02 ;	C213 - C216 - O219
214	213	215	1	1.0855e+02	2.9824e+02 ;	H214 - C213 - H215

214	213	216	1	1.0959e+02	3.6217e+02 ;	H214 - C213	- C216
215	213	216	1	1.0959e+02	3.6217e+02 ;	H215 - C213	- C216
216	219	220	1	1.1360e+02	7.3915e+02 ;	C216 - O219	- C220
217	216	218	1	1.0855e+02	2.9824e+02 ;	H217 - C216	- H218
217	216	219	1	1.1034e+02	4.7028e+02 ;	H217 - C216	- O219
218	216	219	1	1.1034e+02	4.7028e+02 ;	H218 - C216	- O219
219	220	221	1	1.1034e+02	4.7028e+02 ;	O219 - C220	- H221
219	220	222	1	1.1034e+02	4.7028e+02 ;	O219 - C220	- H222
219	220	223	1	1.0762e+02	6.4802e+02 ;	O219 - C220	- C223
220	223	224	1	1.0959e+02	3.6217e+02 ;	C220 - C223	- H224
220	223	225	1	1.0959e+02	3.6217e+02 ;	C220 - C223	- H225
220	223	226	1	1.0762e+02	6.4802e+02 ;	C220 - C223	- O226
221	220	222	1	1.0855e+02	2.9824e+02 ;	H221 - C220	- H222
221	220	223	1	1.0959e+02	3.6217e+02 ;	H221 - C220	- C223
222	220	223	1	1.0959e+02	3.6217e+02 ;	H222 - C220	- C223
223	226	227	1	1.1360e+02	7.3915e+02 ;	C223 - O226	- C227
224	223	225	1	1.0855e+02	2.9824e+02 ;	H224 - C223	- H225
224	223	226	1	1.1034e+02	4.7028e+02 ;	H224 - C223	- O226
225	223	226	1	1.1034e+02	4.7028e+02 ;	H225 - C223	- O226
226	227	228	1	1.1034e+02	4.7028e+02 ;	O226 - C227	- H228
226	227	229	1	1.1034e+02	4.7028e+02 ;	O226 - C227	- H229
226	227	230	1	1.0762e+02	6.4802e+02 ;	O226 - C227	- C230
227	230	231	1	1.0959e+02	3.6217e+02 ;	C227 - C230	- H231
227	230	232	1	1.0959e+02	3.6217e+02 ;	C227 - C230	- H232
227	230	233	1	1.0762e+02	6.4802e+02 ;	C227 - C230	- O233
228	227	229	1	1.0855e+02	2.9824e+02 ;	H228 - C227	- H229
228	227	230	1	1.0959e+02	3.6217e+02 ;	H228 - C227	- C230
229	227	230	1	1.0959e+02	3.6217e+02 ;	H229 - C227	- C230
230	233	234	1	1.1360e+02	7.3915e+02 ;	C230 - O233	- C234

231	230	232	1	1.0855e+02	2.9824e+02 ;	H231 - C230	- H232
231	230	233	1	1.1034e+02	4.7028e+02 ;	H231 - C230	- O233
232	230	233	1	1.1034e+02	4.7028e+02 ;	H232 - C230	- O233
233	234	235	1	1.1034e+02	4.7028e+02 ;	O233 - C234	- H235
233	234	236	1	1.1034e+02	4.7028e+02 ;	O233 - C234	- H236
233	234	237	1	1.0762e+02	6.4802e+02 ;	O233 - C234	- C237
234	237	238	1	1.0959e+02	3.6217e+02 ;	C234 - C237	- H238
234	237	239	1	1.0959e+02	3.6217e+02 ;	C234 - C237	- H239
234	237	240	1	1.0762e+02	6.4802e+02 ;	C234 - C237	- O240
235	234	236	1	1.0855e+02	2.9824e+02 ;	H235 - C234	- H236
235	234	237	1	1.0959e+02	3.6217e+02 ;	H235 - C234	- C237
236	234	237	1	1.0959e+02	3.6217e+02 ;	H236 - C234	- C237
237	240	241	1	1.1360e+02	7.3915e+02 ;	C237 - O240	- C241
238	237	239	1	1.0855e+02	2.9824e+02 ;	H238 - C237	- H239
238	237	240	1	1.1034e+02	4.7028e+02 ;	H238 - C237	- O240
239	237	240	1	1.1034e+02	4.7028e+02 ;	H239 - C237	- O240
240	241	242	1	1.1034e+02	4.7028e+02 ;	O240 - C241	- H242
240	241	243	1	1.1034e+02	4.7028e+02 ;	O240 - C241	- H243
240	241	244	1	1.0762e+02	6.4802e+02 ;	O240 - C241	- C244
241	244	245	1	1.0959e+02	3.6217e+02 ;	C241 - C244	- H245
241	244	246	1	1.0959e+02	3.6217e+02 ;	C241 - C244	- H246
241	244	247	1	1.0762e+02	6.4802e+02 ;	C241 - C244	- O247
242	241	243	1	1.0855e+02	2.9824e+02 ;	H242 - C241	- H243
242	241	244	1	1.0959e+02	3.6217e+02 ;	H242 - C241	- C244
243	241	244	1	1.0959e+02	3.6217e+02 ;	H243 - C241	- C244
244	247	248	1	1.1360e+02	7.3915e+02 ;	C244 - O247	- C248
245	244	246	1	1.0855e+02	2.9824e+02 ;	H245 - C244	- H246
245	244	247	1	1.1034e+02	4.7028e+02 ;	H245 - C244	- O247
246	244	247	1	1.1034e+02	4.7028e+02 ;	H246 - C244	- O247

247	248	249	1	1.1034e+02	4.7028e+02 ;	O247	- C248	- H249
247	248	250	1	1.1034e+02	4.7028e+02 ;	O247	- C248	- H250
247	248	251	1	1.0762e+02	6.4802e+02 ;	O247	- C248	- C251
248	251	252	1	1.0959e+02	3.6217e+02 ;	C248	- C251	- H252
248	251	253	1	1.0959e+02	3.6217e+02 ;	C248	- C251	- H253
248	251	254	1	1.0762e+02	6.4802e+02 ;	C248	- C251	- O254
249	248	250	1	1.0855e+02	2.9824e+02 ;	H249	- C248	- H250
249	248	251	1	1.0959e+02	3.6217e+02 ;	H249	- C248	- C251
250	248	251	1	1.0959e+02	3.6217e+02 ;	H250	- C248	- C251
251	254	255	1	1.1360e+02	7.3915e+02 ;	C251	- O254	- C255
252	251	253	1	1.0855e+02	2.9824e+02 ;	H252	- C251	- H253
252	251	254	1	1.1034e+02	4.7028e+02 ;	H252	- C251	- O254
253	251	254	1	1.1034e+02	4.7028e+02 ;	H253	- C251	- O254
254	255	256	1	1.1034e+02	4.7028e+02 ;	O254	- C255	- H256
254	255	257	1	1.1034e+02	4.7028e+02 ;	O254	- C255	- H257
254	255	258	1	1.0762e+02	6.4802e+02 ;	O254	- C255	- C258
255	258	259	1	1.0959e+02	3.6217e+02 ;	C255	- C258	- H259
255	258	260	1	1.0959e+02	3.6217e+02 ;	C255	- C258	- H260
255	258	261	1	1.0762e+02	6.4802e+02 ;	C255	- C258	- O261
256	255	257	1	1.0855e+02	2.9824e+02 ;	H256	- C255	- H257
256	255	258	1	1.0959e+02	3.6217e+02 ;	H256	- C255	- C258
257	255	258	1	1.0959e+02	3.6217e+02 ;	H257	- C255	- C258
258	261	262	1	1.1360e+02	7.3915e+02 ;	C258	- O261	- C262
259	258	260	1	1.0855e+02	2.9824e+02 ;	H259	- C258	- H260
259	258	261	1	1.1034e+02	4.7028e+02 ;	H259	- C258	- O261
260	258	261	1	1.1034e+02	4.7028e+02 ;	H260	- C258	- O261
261	262	263	1	1.1034e+02	4.7028e+02 ;	O261	- C262	- H263
261	262	264	1	1.1034e+02	4.7028e+02 ;	O261	- C262	- H264
261	262	265	1	1.0762e+02	6.4802e+02 ;	O261	- C262	- C265

262	265	266	1	1.0959e+02	3.6217e+02 ;	C262	- C265	- H266
262	265	267	1	1.0959e+02	3.6217e+02 ;	C262	- C265	- H267
262	265	268	1	1.0762e+02	6.4802e+02 ;	C262	- C265	- O268
263	262	264	1	1.0855e+02	2.9824e+02 ;	H263	- C262	- H264
263	262	265	1	1.0959e+02	3.6217e+02 ;	H263	- C262	- C265
264	262	265	1	1.0959e+02	3.6217e+02 ;	H264	- C262	- C265
265	268	269	1	1.1360e+02	7.3915e+02 ;	C265	- O268	- C269
266	265	267	1	1.0855e+02	2.9824e+02 ;	H266	- C265	- H267
266	265	268	1	1.1034e+02	4.7028e+02 ;	H266	- C265	- O268
267	265	268	1	1.1034e+02	4.7028e+02 ;	H267	- C265	- O268
268	269	270	1	1.1034e+02	4.7028e+02 ;	O268	- C269	- H270
268	269	271	1	1.1034e+02	4.7028e+02 ;	O268	- C269	- H271
268	269	272	1	1.0762e+02	6.4802e+02 ;	O268	- C269	- C272
269	272	273	1	1.0959e+02	3.6217e+02 ;	C269	- C272	- H273
269	272	274	1	1.0959e+02	3.6217e+02 ;	C269	- C272	- H274
269	272	275	1	1.0762e+02	6.4802e+02 ;	C269	- C272	- O275
270	269	271	1	1.0855e+02	2.9824e+02 ;	H270	- C269	- H271
270	269	272	1	1.0959e+02	3.6217e+02 ;	H270	- C269	- C272
271	269	272	1	1.0959e+02	3.6217e+02 ;	H271	- C269	- C272
272	275	276	1	1.1360e+02	7.3915e+02 ;	C272	- O275	- C276
273	272	274	1	1.0855e+02	2.9824e+02 ;	H273	- C272	- H274
273	272	275	1	1.1034e+02	4.7028e+02 ;	H273	- C272	- O275
274	272	275	1	1.1034e+02	4.7028e+02 ;	H274	- C272	- O275
275	276	277	1	1.1034e+02	4.7028e+02 ;	O275	- C276	- H277
275	276	278	1	1.1034e+02	4.7028e+02 ;	O275	- C276	- H278
275	276	279	1	1.0762e+02	6.4802e+02 ;	O275	- C276	- C279
276	279	280	1	1.0959e+02	3.6217e+02 ;	C276	- C279	- H280
276	279	281	1	1.0959e+02	3.6217e+02 ;	C276	- C279	- H281
276	279	282	1	1.0762e+02	6.4802e+02 ;	C276	- C279	- O282

277	276	278	1	1.0855e+02	2.9824e+02 ;	H277 -	C276	- H278
277	276	279	1	1.0959e+02	3.6217e+02 ;	H277 -	C276	- C279
278	276	279	1	1.0959e+02	3.6217e+02 ;	H278 -	C276	- C279
279	282	283	1	1.1360e+02	7.3915e+02 ;	C279 -	O282	- C283
280	279	281	1	1.0855e+02	2.9824e+02 ;	H280 -	C279	- H281
280	279	282	1	1.1034e+02	4.7028e+02 ;	H280 -	C279	- O282
281	279	282	1	1.1034e+02	4.7028e+02 ;	H281 -	C279	- O282
282	283	284	1	1.1034e+02	4.7028e+02 ;	O282 -	C283	- H284
282	283	285	1	1.1034e+02	4.7028e+02 ;	O282 -	C283	- H285
282	283	286	1	1.0762e+02	6.4802e+02 ;	O282 -	C283	- C286
283	286	287	1	1.0959e+02	3.6217e+02 ;	C283 -	C286	- H287
283	286	288	1	1.0959e+02	3.6217e+02 ;	C283 -	C286	- H288
283	286	289	1	1.0762e+02	6.4802e+02 ;	C283 -	C286	- O289
284	283	285	1	1.0855e+02	2.9824e+02 ;	H284 -	C283	- H285
284	283	286	1	1.0959e+02	3.6217e+02 ;	H284 -	C283	- C286
285	283	286	1	1.0959e+02	3.6217e+02 ;	H285 -	C283	- C286
286	289	290	1	1.1360e+02	7.3915e+02 ;	C286 -	O289	- C290
287	286	288	1	1.0855e+02	2.9824e+02 ;	H287 -	C286	- H288
287	286	289	1	1.1034e+02	4.7028e+02 ;	H287 -	C286	- O289
288	286	289	1	1.1034e+02	4.7028e+02 ;	H288 -	C286	- O289
289	290	291	1	1.1034e+02	4.7028e+02 ;	O289 -	C290	- H291
289	290	292	1	1.1034e+02	4.7028e+02 ;	O289 -	C290	- H292
289	290	293	1	1.0762e+02	6.4802e+02 ;	O289 -	C290	- C293
290	293	294	1	1.0959e+02	3.6217e+02 ;	C290 -	C293	- H294
290	293	295	1	1.0959e+02	3.6217e+02 ;	C290 -	C293	- H295
290	293	296	1	1.0762e+02	6.4802e+02 ;	C290 -	C293	- O296
291	290	292	1	1.0855e+02	2.9824e+02 ;	H291 -	C290	- H292
291	290	293	1	1.0959e+02	3.6217e+02 ;	H291 -	C290	- C293
292	290	293	1	1.0959e+02	3.6217e+02 ;	H292 -	C290	- C293

293	296	297	1	1.1360e+02	7.3915e+02 ;	C293 - O296 - C297
294	293	295	1	1.0855e+02	2.9824e+02 ;	H294 - C293 - H295
294	293	296	1	1.1034e+02	4.7028e+02 ;	H294 - C293 - O296
295	293	296	1	1.1034e+02	4.7028e+02 ;	H295 - C293 - O296
296	297	298	1	1.1034e+02	4.7028e+02 ;	O296 - C297 - H298
296	297	299	1	1.1034e+02	4.7028e+02 ;	O296 - C297 - H299
296	297	300	1	1.0762e+02	6.4802e+02 ;	O296 - C297 - C300
297	300	301	1	1.0959e+02	3.6217e+02 ;	C297 - C300 - H301
297	300	302	1	1.0959e+02	3.6217e+02 ;	C297 - C300 - H302
297	300	303	1	1.0762e+02	6.4802e+02 ;	C297 - C300 - O303
298	297	299	1	1.0855e+02	2.9824e+02 ;	H298 - C297 - H299
298	297	300	1	1.0959e+02	3.6217e+02 ;	H298 - C297 - C300
299	297	300	1	1.0959e+02	3.6217e+02 ;	H299 - C297 - C300
300	303	304	1	1.1360e+02	7.3915e+02 ;	C300 - O303 - C304
301	300	302	1	1.0855e+02	2.9824e+02 ;	H301 - C300 - H302
301	300	303	1	1.1034e+02	4.7028e+02 ;	H301 - C300 - O303
302	300	303	1	1.1034e+02	4.7028e+02 ;	H302 - C300 - O303
303	304	305	1	1.1034e+02	4.7028e+02 ;	O303 - C304 - H305
303	304	306	1	1.1034e+02	4.7028e+02 ;	O303 - C304 - H306
303	304	307	1	1.0762e+02	6.4802e+02 ;	O303 - C304 - C307
304	307	308	1	1.0959e+02	3.6217e+02 ;	C304 - C307 - H308
304	307	309	1	1.0959e+02	3.6217e+02 ;	C304 - C307 - H309
304	307	310	1	1.0762e+02	6.4802e+02 ;	C304 - C307 - O310
305	304	306	1	1.0855e+02	2.9824e+02 ;	H305 - C304 - H306
305	304	307	1	1.0959e+02	3.6217e+02 ;	H305 - C304 - C307
306	304	307	1	1.0959e+02	3.6217e+02 ;	H306 - C304 - C307
307	310	311	1	1.1360e+02	7.3915e+02 ;	C307 - O310 - C311
308	307	309	1	1.0855e+02	2.9824e+02 ;	H308 - C307 - H309
308	307	310	1	1.1034e+02	4.7028e+02 ;	H308 - C307 - O310

```
  309   307   310     1    1.1034e+02    4.7028e+02 ;   H309 - C307   - O310
  310   311   312     1    1.1034e+02    4.7028e+02 ;   O310 - C311   - H312
  310   311   313     1    1.1034e+02    4.7028e+02 ;   O310 - C311   - H313
  310   311   314     1    1.0762e+02    6.4802e+02 ;   O310 - C311   - C314
  311   314   315     1    1.0959e+02    3.6217e+02 ;   C311 - C314   - H315
  311   314   316     1    1.0959e+02    3.6217e+02 ;   C311 - C314   - H316
  311   314   317     1    1.0966e+02    6.4258e+02 ;   C311 - C314   - O317
  312   311   313     1    1.0855e+02    2.9824e+02 ;   H312 - C311   - H313
  312   311   314     1    1.0959e+02    3.6217e+02 ;   H312 - C311   - C314
  313   311   314     1    1.0959e+02    3.6217e+02 ;   H313 - C311   - C314
  314   317   318     1    1.0739e+02    5.4501e+02 ;   C314 - O317   - H318
  315   314   316     1    1.0855e+02    2.9824e+02 ;   H315 - C314   - H316
  315   314   317     1    1.1038e+02    4.7103e+02 ;   H315 - C314   - O317
  316   314   317     1    1.1038e+02    4.7103e+02 ;   H316 - C314   - O317

[ dihedrals ] ; propers
; for gromacs 4.5 or higher, using funct 9
;    i      j      k      l   func   phase     kd      pn
     1      3      6      7      9     0.00   0.00000   0 ;     O1-    C3-    C6-    H7
     1      3      6      7      9     0.00   1.04600   1 ;     O1-    C3-    C6-    H7
     1      3      6      8      9     0.00   0.00000   0 ;     O1-    C3-    C6-    H8
     1      3      6      8      9     0.00   1.04600   1 ;     O1-    C3-    C6-    H8
     1      3      6      9      9     0.00   0.65084   3 ;     O1-    C3-    C6-    O9
     2      1      3      4      9     0.00   0.51463   3 ;     H2-    O1-    C3-    H4
     2      1      3      5      9     0.00   0.51463   3 ;     H2-    O1-    C3-    H5
     2      1      3      6      9     0.00   0.25104   3 ;     H2-    O1-    C3-    C6
     3      6      9     10      9     0.00   0.33472   2 ;     C3-    C6-    O9-   C10
     3      6      9     10      9     0.00   3.13800   3 ;     C3-    C6-    O9-   C10
     3      6      9     10      9   180.00   3.68192   1 ;     C3-    C6-    O9-   C10
```

4	3	6	7	9	0.00	0.65084	3	;	H4-	C3-	C6-	H7
4	3	6	8	9	0.00	0.65084	3	;	H4-	C3-	C6-	H8
4	3	6	9	9	0.00	0.00000	0	;	H4-	C3-	C6-	O9
4	3	6	9	9	0.00	1.04600	1	;	H4-	C3-	C6-	O9
5	3	6	7	9	0.00	0.65084	3	;	H5-	C3-	C6-	H7
5	3	6	8	9	0.00	0.65084	3	;	H5-	C3-	C6-	H8
5	3	6	9	9	0.00	0.00000	0	;	H5-	C3-	C6-	O9
5	3	6	9	9	0.00	1.04600	1	;	H5-	C3-	C6-	O9
6	9	10	11	9	0.00	1.41001	3	;	C6-	O9-	C10-	H11
6	9	10	12	9	0.00	1.41001	3	;	C6-	O9-	C10-	H12
6	9	10	13	9	0.00	0.33472	2	;	C6-	O9-	C10-	C13
6	9	10	13	9	0.00	3.13800	3	;	C6-	O9-	C10-	C13
6	9	10	13	9	180.00	3.68192	1	;	C6-	O9-	C10-	C13
7	6	9	10	9	0.00	1.41001	3	;	H7-	C6-	O9-	C10
8	6	9	10	9	0.00	1.41001	3	;	H8-	C6-	O9-	C10
9	10	13	14	9	0.00	0.00000	0	;	O9-	C10-	C13-	H14
9	10	13	14	9	0.00	1.04600	1	;	O9-	C10-	C13-	H14
9	10	13	15	9	0.00	0.00000	0	;	O9-	C10-	C13-	H15
9	10	13	15	9	0.00	1.04600	1	;	O9-	C10-	C13-	H15
9	10	13	16	9	0.00	0.75312	1	;	O9-	C10-	C13-	O16
9	10	13	16	9	0.00	2.09200	3	;	O9-	C10-	C13-	O16
9	10	13	16	9	0.00	3.76560	2	;	O9-	C10-	C13-	O16
10	13	16	17	9	0.00	0.33472	2	;	C10-	C13-	O16-	C17
10	13	16	17	9	0.00	3.13800	3	;	C10-	C13-	O16-	C17
10	13	16	17	9	180.00	3.68192	1	;	C10-	C13-	O16-	C17
11	10	13	14	9	0.00	0.65084	3	;	H11-	C10-	C13-	H14
11	10	13	15	9	0.00	0.65084	3	;	H11-	C10-	C13-	H15
11	10	13	16	9	0.00	0.00000	0	;	H11-	C10-	C13-	O16
11	10	13	16	9	0.00	1.04600	1	;	H11-	C10-	C13-	O16

12	10	13	14	9	0.00	0.65084	3 ;	H12-	C10-	C13-	H14
12	10	13	15	9	0.00	0.65084	3 ;	H12-	C10-	C13-	H15
12	10	13	16	9	0.00	0.00000	0 ;	H12-	C10-	C13-	O16
12	10	13	16	9	0.00	1.04600	1 ;	H12-	C10-	C13-	O16
13	16	17	18	9	0.00	1.41001	3 ;	C13-	O16-	C17-	H18
13	16	17	19	9	0.00	1.41001	3 ;	C13-	O16-	C17-	H19
13	16	17	20	9	0.00	0.33472	2 ;	C13-	O16-	C17-	C20
13	16	17	20	9	0.00	3.13800	3 ;	C13-	O16-	C17-	C20
13	16	17	20	9	180.00	3.68192	1 ;	C13-	O16-	C17-	C20
14	13	16	17	9	0.00	1.41001	3 ;	H14-	C13-	O16-	C17
15	13	16	17	9	0.00	1.41001	3 ;	H15-	C13-	O16-	C17
16	17	20	21	9	0.00	0.00000	0 ;	O16-	C17-	C20-	H21
16	17	20	21	9	0.00	1.04600	1 ;	O16-	C17-	C20-	H21
16	17	20	22	9	0.00	0.00000	0 ;	O16-	C17-	C20-	H22
16	17	20	22	9	0.00	1.04600	1 ;	O16-	C17-	C20-	H22
16	17	20	23	9	0.00	0.75312	1 ;	O16-	C17-	C20-	O23
16	17	20	23	9	0.00	2.09200	3 ;	O16-	C17-	C20-	O23
16	17	20	23	9	0.00	3.76560	2 ;	O16-	C17-	C20-	O23
17	20	23	24	9	0.00	0.33472	2 ;	C17-	C20-	O23-	C24
17	20	23	24	9	0.00	3.13800	3 ;	C17-	C20-	O23-	C24
17	20	23	24	9	180.00	3.68192	1 ;	C17-	C20-	O23-	C24
18	17	20	21	9	0.00	0.65084	3 ;	H18-	C17-	C20-	H21
18	17	20	22	9	0.00	0.65084	3 ;	H18-	C17-	C20-	H22
18	17	20	23	9	0.00	0.00000	0 ;	H18-	C17-	C20-	O23
18	17	20	23	9	0.00	1.04600	1 ;	H18-	C17-	C20-	O23
19	17	20	21	9	0.00	0.65084	3 ;	H19-	C17-	C20-	H21
19	17	20	22	9	0.00	0.65084	3 ;	H19-	C17-	C20-	H22
19	17	20	23	9	0.00	0.00000	0 ;	H19-	C17-	C20-	O23
19	17	20	23	9	0.00	1.04600	1 ;	H19-	C17-	C20-	O23

20	23	24	25	9	0.00	1.41001	3 ;	C20-	O23-	C24-	H25
20	23	24	26	9	0.00	1.41001	3 ;	C20-	O23-	C24-	H26
20	23	24	27	9	0.00	0.33472	2 ;	C20-	O23-	C24-	C27
20	23	24	27	9	0.00	3.13800	3 ;	C20-	O23-	C24-	C27
20	23	24	27	9	180.00	3.68192	1 ;	C20-	O23-	C24-	C27
21	20	23	24	9	0.00	1.41001	3 ;	H21-	C20-	O23-	C24
22	20	23	24	9	0.00	1.41001	3 ;	H22-	C20-	O23-	C24
23	24	27	28	9	0.00	0.00000	0 ;	O23-	C24-	C27-	H28
23	24	27	28	9	0.00	1.04600	1 ;	O23-	C24-	C27-	H28
23	24	27	29	9	0.00	0.00000	0 ;	O23-	C24-	C27-	H29
23	24	27	29	9	0.00	1.04600	1 ;	O23-	C24-	C27-	H29
23	24	27	30	9	0.00	0.75312	1 ;	O23-	C24-	C27-	O30
23	24	27	30	9	0.00	2.09200	3 ;	O23-	C24-	C27-	O30
23	24	27	30	9	0.00	3.76560	2 ;	O23-	C24-	C27-	O30
24	27	30	31	9	0.00	0.33472	2 ;	C24-	C27-	O30-	C31
24	27	30	31	9	0.00	3.13800	3 ;	C24-	C27-	O30-	C31
24	27	30	31	9	180.00	3.68192	1 ;	C24-	C27-	O30-	C31
25	24	27	28	9	0.00	0.65084	3 ;	H25-	C24-	C27-	H28
25	24	27	29	9	0.00	0.65084	3 ;	H25-	C24-	C27-	H29
25	24	27	30	9	0.00	0.00000	0 ;	H25-	C24-	C27-	O30
25	24	27	30	9	0.00	1.04600	1 ;	H25-	C24-	C27-	O30
26	24	27	28	9	0.00	0.65084	3 ;	H26-	C24-	C27-	H28
26	24	27	29	9	0.00	0.65084	3 ;	H26-	C24-	C27-	H29
26	24	27	30	9	0.00	0.00000	0 ;	H26-	C24-	C27-	O30
26	24	27	30	9	0.00	1.04600	1 ;	H26-	C24-	C27-	O30
27	30	31	32	9	0.00	1.41001	3 ;	C27-	O30-	C31-	H32
27	30	31	33	9	0.00	1.41001	3 ;	C27-	O30-	C31-	H33
27	30	31	34	9	0.00	0.33472	2 ;	C27-	O30-	C31-	C34
27	30	31	34	9	0.00	3.13800	3 ;	C27-	O30-	C31-	C34

27	30	31	34	9	180.00	3.68192	1	;	C27-	O30-	C31-	C34
28	27	30	31	9	0.00	1.41001	3	;	H28-	C27-	O30-	C31
29	27	30	31	9	0.00	1.41001	3	;	H29-	C27-	O30-	C31
30	31	34	35	9	0.00	0.00000	0	;	O30-	C31-	C34-	H35
30	31	34	35	9	0.00	1.04600	1	;	O30-	C31-	C34-	H35
30	31	34	36	9	0.00	0.00000	0	;	O30-	C31-	C34-	H36
30	31	34	36	9	0.00	1.04600	1	;	O30-	C31-	C34-	H36
30	31	34	37	9	0.00	0.75312	1	;	O30-	C31-	C34-	O37
30	31	34	37	9	0.00	2.09200	3	;	O30-	C31-	C34-	O37
30	31	34	37	9	0.00	3.76560	2	;	O30-	C31-	C34-	O37
31	34	37	38	9	0.00	0.33472	2	;	C31-	C34-	O37-	C38
31	34	37	38	9	0.00	3.13800	3	;	C31-	C34-	O37-	C38
31	34	37	38	9	180.00	3.68192	1	;	C31-	C34-	O37-	C38
32	31	34	35	9	0.00	0.65084	3	;	H32-	C31-	C34-	H35
32	31	34	36	9	0.00	0.65084	3	;	H32-	C31-	C34-	H36
32	31	34	37	9	0.00	0.00000	0	;	H32-	C31-	C34-	O37
32	31	34	37	9	0.00	1.04600	1	;	H32-	C31-	C34-	O37
33	31	34	35	9	0.00	0.65084	3	;	H33-	C31-	C34-	H35
33	31	34	36	9	0.00	0.65084	3	;	H33-	C31-	C34-	H36
33	31	34	37	9	0.00	0.00000	0	;	H33-	C31-	C34-	O37
33	31	34	37	9	0.00	1.04600	1	;	H33-	C31-	C34-	O37
34	37	38	39	9	0.00	1.41001	3	;	C34-	O37-	C38-	H39
34	37	38	40	9	0.00	1.41001	3	;	C34-	O37-	C38-	H40
34	37	38	41	9	0.00	0.33472	2	;	C34-	O37-	C38-	C41
34	37	38	41	9	0.00	3.13800	3	;	C34-	O37-	C38-	C41
34	37	38	41	9	180.00	3.68192	1	;	C34-	O37-	C38-	C41
35	34	37	38	9	0.00	1.41001	3	;	H35-	C34-	O37-	C38
36	34	37	38	9	0.00	1.41001	3	;	H36-	C34-	O37-	C38
37	38	41	42	9	0.00	0.00000	0	;	O37-	C38-	C41-	H42

37	38	41	42	9	0.00	1.04600	1 ;	O37-	C38-	C41-	H42
37	38	41	43	9	0.00	0.00000	0 ;	O37-	C38-	C41-	H43
37	38	41	43	9	0.00	1.04600	1 ;	O37-	C38-	C41-	H43
37	38	41	44	9	0.00	0.75312	1 ;	O37-	C38-	C41-	O44
37	38	41	44	9	0.00	2.09200	3 ;	O37-	C38-	C41-	O44
37	38	41	44	9	0.00	3.76560	2 ;	O37-	C38-	C41-	O44
38	41	44	45	9	0.00	0.33472	2 ;	C38-	C41-	O44-	C45
38	41	44	45	9	0.00	3.13800	3 ;	C38-	C41-	O44-	C45
38	41	44	45	9	180.00	3.68192	1 ;	C38-	C41-	O44-	C45
39	38	41	42	9	0.00	0.65084	3 ;	H39-	C38-	C41-	H42
39	38	41	43	9	0.00	0.65084	3 ;	H39-	C38-	C41-	H43
39	38	41	44	9	0.00	0.00000	0 ;	H39-	C38-	C41-	O44
39	38	41	44	9	0.00	1.04600	1 ;	H39-	C38-	C41-	O44
40	38	41	42	9	0.00	0.65084	3 ;	H40-	C38-	C41-	H42
40	38	41	43	9	0.00	0.65084	3 ;	H40-	C38-	C41-	H43
40	38	41	44	9	0.00	0.00000	0 ;	H40-	C38-	C41-	O44
40	38	41	44	9	0.00	1.04600	1 ;	H40-	C38-	C41-	O44
41	44	45	46	9	0.00	1.41001	3 ;	C41-	O44-	C45-	H46
41	44	45	47	9	0.00	1.41001	3 ;	C41-	O44-	C45-	H47
41	44	45	48	9	0.00	0.33472	2 ;	C41-	O44-	C45-	C48
41	44	45	48	9	0.00	3.13800	3 ;	C41-	O44-	C45-	C48
41	44	45	48	9	180.00	3.68192	1 ;	C41-	O44-	C45-	C48
42	41	44	45	9	0.00	1.41001	3 ;	H42-	C41-	O44-	C45
43	41	44	45	9	0.00	1.41001	3 ;	H43-	C41-	O44-	C45
44	45	48	49	9	0.00	0.00000	0 ;	O44-	C45-	C48-	H49
44	45	48	49	9	0.00	1.04600	1 ;	O44-	C45-	C48-	H49
44	45	48	50	9	0.00	0.00000	0 ;	O44-	C45-	C48-	H50
44	45	48	50	9	0.00	1.04600	1 ;	O44-	C45-	C48-	H50
44	45	48	51	9	0.00	0.75312	1 ;	O44-	C45-	C48-	O51

44	45	48	51	9	0.00	2.09200	3	;	O44-	C45-	C48-	O51
44	45	48	51	9	0.00	3.76560	2	;	O44-	C45-	C48-	O51
45	48	51	52	9	0.00	0.33472	2	;	C45-	C48-	O51-	C52
45	48	51	52	9	0.00	3.13800	3	;	C45-	C48-	O51-	C52
45	48	51	52	9	180.00	3.68192	1	;	C45-	C48-	O51-	C52
46	45	48	49	9	0.00	0.65084	3	;	H46-	C45-	C48-	H49
46	45	48	50	9	0.00	0.65084	3	;	H46-	C45-	C48-	H50
46	45	48	51	9	0.00	0.00000	0	;	H46-	C45-	C48-	O51
46	45	48	51	9	0.00	1.04600	1	;	H46-	C45-	C48-	O51
47	45	48	49	9	0.00	0.65084	3	;	H47-	C45-	C48-	H49
47	45	48	50	9	0.00	0.65084	3	;	H47-	C45-	C48-	H50
47	45	48	51	9	0.00	0.00000	0	;	H47-	C45-	C48-	O51
47	45	48	51	9	0.00	1.04600	1	;	H47-	C45-	C48-	O51
48	51	52	53	9	0.00	1.41001	3	;	C48-	O51-	C52-	H53
48	51	52	54	9	0.00	1.41001	3	;	C48-	O51-	C52-	H54
48	51	52	55	9	0.00	0.33472	2	;	C48-	O51-	C52-	C55
48	51	52	55	9	0.00	3.13800	3	;	C48-	O51-	C52-	C55
48	51	52	55	9	180.00	3.68192	1	;	C48-	O51-	C52-	C55
49	48	51	52	9	0.00	1.41001	3	;	H49-	C48-	O51-	C52
50	48	51	52	9	0.00	1.41001	3	;	H50-	C48-	O51-	C52
51	52	55	56	9	0.00	0.00000	0	;	O51-	C52-	C55-	H56
51	52	55	56	9	0.00	1.04600	1	;	O51-	C52-	C55-	H56
51	52	55	57	9	0.00	0.00000	0	;	O51-	C52-	C55-	H57
51	52	55	57	9	0.00	1.04600	1	;	O51-	C52-	C55-	H57
51	52	55	58	9	0.00	0.75312	1	;	O51-	C52-	C55-	O58
51	52	55	58	9	0.00	2.09200	3	;	O51-	C52-	C55-	O58
51	52	55	58	9	0.00	3.76560	2	;	O51-	C52-	C55-	O58
52	55	58	59	9	0.00	0.33472	2	;	C52-	C55-	O58-	C59
52	55	58	59	9	0.00	3.13800	3	;	C52-	C55-	O58-	C59

52	55	58	59	9	180.00	3.68192	1 ;	C52-	C55-	O58-	C59
53	52	55	56	9	0.00	0.65084	3 ;	H53-	C52-	C55-	H56
53	52	55	57	9	0.00	0.65084	3 ;	H53-	C52-	C55-	H57
53	52	55	58	9	0.00	0.00000	0 ;	H53-	C52-	C55-	O58
53	52	55	58	9	0.00	1.04600	1 ;	H53-	C52-	C55-	O58
54	52	55	56	9	0.00	0.65084	3 ;	H54-	C52-	C55-	H56
54	52	55	57	9	0.00	0.65084	3 ;	H54-	C52-	C55-	H57
54	52	55	58	9	0.00	0.00000	0 ;	H54-	C52-	C55-	O58
54	52	55	58	9	0.00	1.04600	1 ;	H54-	C52-	C55-	O58
55	58	59	60	9	0.00	1.41001	3 ;	C55-	O58-	C59-	H60
55	58	59	61	9	0.00	1.41001	3 ;	C55-	O58-	C59-	H61
55	58	59	62	9	0.00	0.33472	2 ;	C55-	O58-	C59-	C62
55	58	59	62	9	0.00	3.13800	3 ;	C55-	O58-	C59-	C62
55	58	59	62	9	180.00	3.68192	1 ;	C55-	O58-	C59-	C62
56	55	58	59	9	0.00	1.41001	3 ;	H56-	C55-	O58-	C59
57	55	58	59	9	0.00	1.41001	3 ;	H57-	C55-	O58-	C59
58	59	62	63	9	0.00	0.00000	0 ;	O58-	C59-	C62-	H63
58	59	62	63	9	0.00	1.04600	1 ;	O58-	C59-	C62-	H63
58	59	62	64	9	0.00	0.00000	0 ;	O58-	C59-	C62-	H64
58	59	62	64	9	0.00	1.04600	1 ;	O58-	C59-	C62-	H64
58	59	62	65	9	0.00	0.75312	1 ;	O58-	C59-	C62-	O65
58	59	62	65	9	0.00	2.09200	3 ;	O58-	C59-	C62-	O65
58	59	62	65	9	0.00	3.76560	2 ;	O58-	C59-	C62-	O65
59	62	65	66	9	0.00	0.33472	2 ;	C59-	C62-	O65-	C66
59	62	65	66	9	0.00	3.13800	3 ;	C59-	C62-	O65-	C66
59	62	65	66	9	180.00	3.68192	1 ;	C59-	C62-	O65-	C66
60	59	62	63	9	0.00	0.65084	3 ;	H60-	C59-	C62-	H63
60	59	62	64	9	0.00	0.65084	3 ;	H60-	C59-	C62-	H64
60	59	62	65	9	0.00	0.00000	0 ;	H60-	C59-	C62-	O65

60	59	62	65	9	0.00	1.04600	1	;	H60-	C59-	C62-	O65
61	59	62	63	9	0.00	0.65084	3	;	H61-	C59-	C62-	H63
61	59	62	64	9	0.00	0.65084	3	;	H61-	C59-	C62-	H64
61	59	62	65	9	0.00	0.00000	0	;	H61-	C59-	C62-	O65
61	59	62	65	9	0.00	1.04600	1	;	H61-	C59-	C62-	O65
62	65	66	67	9	0.00	1.41001	3	;	C62-	O65-	C66-	H67
62	65	66	68	9	0.00	1.41001	3	;	C62-	O65-	C66-	H68
62	65	66	69	9	0.00	0.33472	2	;	C62-	O65-	C66-	C69
62	65	66	69	9	0.00	3.13800	3	;	C62-	O65-	C66-	C69
62	65	66	69	9	180.00	3.68192	1	;	C62-	O65-	C66-	C69
63	62	65	66	9	0.00	1.41001	3	;	H63-	C62-	O65-	C66
64	62	65	66	9	0.00	1.41001	3	;	H64-	C62-	O65-	C66
65	66	69	70	9	0.00	0.00000	0	;	O65-	C66-	C69-	H70
65	66	69	70	9	0.00	1.04600	1	;	O65-	C66-	C69-	H70
65	66	69	71	9	0.00	0.00000	0	;	O65-	C66-	C69-	H71
65	66	69	71	9	0.00	1.04600	1	;	O65-	C66-	C69-	H71
65	66	69	72	9	0.00	0.75312	1	;	O65-	C66-	C69-	O72
65	66	69	72	9	0.00	2.09200	3	;	O65-	C66-	C69-	O72
65	66	69	72	9	0.00	3.76560	2	;	O65-	C66-	C69-	O72
66	69	72	73	9	0.00	0.33472	2	;	C66-	C69-	O72-	C73
66	69	72	73	9	0.00	3.13800	3	;	C66-	C69-	O72-	C73
66	69	72	73	9	180.00	3.68192	1	;	C66-	C69-	O72-	C73
67	66	69	70	9	0.00	0.65084	3	;	H67-	C66-	C69-	H70
67	66	69	71	9	0.00	0.65084	3	;	H67-	C66-	C69-	H71
67	66	69	72	9	0.00	0.00000	0	;	H67-	C66-	C69-	O72
67	66	69	72	9	0.00	1.04600	1	;	H67-	C66-	C69-	O72
68	66	69	70	9	0.00	0.65084	3	;	H68-	C66-	C69-	H70
68	66	69	71	9	0.00	0.65084	3	;	H68-	C66-	C69-	H71
68	66	69	72	9	0.00	0.00000	0	;	H68-	C66-	C69-	O72

68	66	69	72	9	0.00	1.04600	1	;	H68-	C66-	C69-	O72
69	72	73	74	9	0.00	1.41001	3	;	C69-	O72-	C73-	H74
69	72	73	75	9	0.00	1.41001	3	;	C69-	O72-	C73-	H75
69	72	73	76	9	0.00	0.33472	2	;	C69-	O72-	C73-	C76
69	72	73	76	9	0.00	3.13800	3	;	C69-	O72-	C73-	C76
69	72	73	76	9	180.00	3.68192	1	;	C69-	O72-	C73-	C76
70	69	72	73	9	0.00	1.41001	3	;	H70-	C69-	O72-	C73
71	69	72	73	9	0.00	1.41001	3	;	H71-	C69-	O72-	C73
72	73	76	77	9	0.00	0.00000	0	;	O72-	C73-	C76-	H77
72	73	76	77	9	0.00	1.04600	1	;	O72-	C73-	C76-	H77
72	73	76	78	9	0.00	0.00000	0	;	O72-	C73-	C76-	H78
72	73	76	78	9	0.00	1.04600	1	;	O72-	C73-	C76-	H78
72	73	76	79	9	0.00	0.75312	1	;	O72-	C73-	C76-	O79
72	73	76	79	9	0.00	2.09200	3	;	O72-	C73-	C76-	O79
72	73	76	79	9	0.00	3.76560	2	;	O72-	C73-	C76-	O79
73	76	79	80	9	0.00	0.33472	2	;	C73-	C76-	O79-	C80
73	76	79	80	9	0.00	3.13800	3	;	C73-	C76-	O79-	C80
73	76	79	80	9	180.00	3.68192	1	;	C73-	C76-	O79-	C80
74	73	76	77	9	0.00	0.65084	3	;	H74-	C73-	C76-	H77
74	73	76	78	9	0.00	0.65084	3	;	H74-	C73-	C76-	H78
74	73	76	79	9	0.00	0.00000	0	;	H74-	C73-	C76-	O79
74	73	76	79	9	0.00	1.04600	1	;	H74-	C73-	C76-	O79
75	73	76	77	9	0.00	0.65084	3	;	H75-	C73-	C76-	H77
75	73	76	78	9	0.00	0.65084	3	;	H75-	C73-	C76-	H78
75	73	76	79	9	0.00	0.00000	0	;	H75-	C73-	C76-	O79
75	73	76	79	9	0.00	1.04600	1	;	H75-	C73-	C76-	O79
76	79	80	81	9	0.00	1.41001	3	;	C76-	O79-	C80-	H81
76	79	80	82	9	0.00	1.41001	3	;	C76-	O79-	C80-	H82
76	79	80	83	9	0.00	0.33472	2	;	C76-	O79-	C80-	C83

76	79	80	83	9	0.00	3.13800	3	;	C76-	O79-	C80-	C83
76	79	80	83	9	180.00	3.68192	1	;	C76-	O79-	C80-	C83
77	76	79	80	9	0.00	1.41001	3	;	H77-	C76-	O79-	C80
78	76	79	80	9	0.00	1.41001	3	;	H78-	C76-	O79-	C80
79	80	83	84	9	0.00	0.00000	0	;	O79-	C80-	C83-	H84
79	80	83	84	9	0.00	1.04600	1	;	O79-	C80-	C83-	H84
79	80	83	85	9	0.00	0.00000	0	;	O79-	C80-	C83-	H85
79	80	83	85	9	0.00	1.04600	1	;	O79-	C80-	C83-	H85
79	80	83	86	9	0.00	0.75312	1	;	O79-	C80-	C83-	O86
79	80	83	86	9	0.00	2.09200	3	;	O79-	C80-	C83-	O86
79	80	83	86	9	0.00	3.76560	2	;	O79-	C80-	C83-	O86
80	83	86	87	9	0.00	0.33472	2	;	C80-	C83-	O86-	C87
80	83	86	87	9	0.00	3.13800	3	;	C80-	C83-	O86-	C87
80	83	86	87	9	180.00	3.68192	1	;	C80-	C83-	O86-	C87
81	80	83	84	9	0.00	0.65084	3	;	H81-	C80-	C83-	H84
81	80	83	85	9	0.00	0.65084	3	;	H81-	C80-	C83-	H85
81	80	83	86	9	0.00	0.00000	0	;	H81-	C80-	C83-	O86
81	80	83	86	9	0.00	1.04600	1	;	H81-	C80-	C83-	O86
82	80	83	84	9	0.00	0.65084	3	;	H82-	C80-	C83-	H84
82	80	83	85	9	0.00	0.65084	3	;	H82-	C80-	C83-	H85
82	80	83	86	9	0.00	0.00000	0	;	H82-	C80-	C83-	O86
82	80	83	86	9	0.00	1.04600	1	;	H82-	C80-	C83-	O86
83	86	87	88	9	0.00	1.41001	3	;	C83-	O86-	C87-	H88
83	86	87	89	9	0.00	1.41001	3	;	C83-	O86-	C87-	H89
83	86	87	90	9	0.00	0.33472	2	;	C83-	O86-	C87-	C90
83	86	87	90	9	0.00	3.13800	3	;	C83-	O86-	C87-	C90
83	86	87	90	9	180.00	3.68192	1	;	C83-	O86-	C87-	C90
84	83	86	87	9	0.00	1.41001	3	;	H84-	C83-	O86-	C87
85	83	86	87	9	0.00	1.41001	3	;	H85-	C83-	O86-	C87

86	87	90	91	9	0.00	0.00000	0	;	O86-	C87-	C90-	H91
86	87	90	91	9	0.00	1.04600	1	;	O86-	C87-	C90-	H91
86	87	90	92	9	0.00	0.00000	0	;	O86-	C87-	C90-	H92
86	87	90	92	9	0.00	1.04600	1	;	O86-	C87-	C90-	H92
86	87	90	93	9	0.00	0.75312	1	;	O86-	C87-	C90-	O93
86	87	90	93	9	0.00	2.09200	3	;	O86-	C87-	C90-	O93
86	87	90	93	9	0.00	3.76560	2	;	O86-	C87-	C90-	O93
87	90	93	94	9	0.00	0.33472	2	;	C87-	C90-	O93-	C94
87	90	93	94	9	0.00	3.13800	3	;	C87-	C90-	O93-	C94
87	90	93	94	9	180.00	3.68192	1	;	C87-	C90-	O93-	C94
88	87	90	91	9	0.00	0.65084	3	;	H88-	C87-	C90-	H91
88	87	90	92	9	0.00	0.65084	3	;	H88-	C87-	C90-	H92
88	87	90	93	9	0.00	0.00000	0	;	H88-	C87-	C90-	O93
88	87	90	93	9	0.00	1.04600	1	;	H88-	C87-	C90-	O93
89	87	90	91	9	0.00	0.65084	3	;	H89-	C87-	C90-	H91
89	87	90	92	9	0.00	0.65084	3	;	H89-	C87-	C90-	H92
89	87	90	93	9	0.00	0.00000	0	;	H89-	C87-	C90-	O93
89	87	90	93	9	0.00	1.04600	1	;	H89-	C87-	C90-	O93
90	93	94	95	9	0.00	1.41001	3	;	C90-	O93-	C94-	H95
90	93	94	96	9	0.00	1.41001	3	;	C90-	O93-	C94-	H96
90	93	94	97	9	0.00	0.33472	2	;	C90-	O93-	C94-	C97
90	93	94	97	9	0.00	3.13800	3	;	C90-	O93-	C94-	C97
90	93	94	97	9	180.00	3.68192	1	;	C90-	O93-	C94-	C97
91	90	93	94	9	0.00	1.41001	3	;	H91-	C90-	O93-	C94
92	90	93	94	9	0.00	1.41001	3	;	H92-	C90-	O93-	C94
93	94	97	98	9	0.00	0.00000	0	;	O93-	C94-	C97-	H98
93	94	97	98	9	0.00	1.04600	1	;	O93-	C94-	C97-	H98
93	94	97	99	9	0.00	0.00000	0	;	O93-	C94-	C97-	H99
93	94	97	99	9	0.00	1.04600	1	;	O93-	C94-	C97-	H99

93	94	97	100	9	0.00	0.75312	1	;	O93-	C94-	C97-	O100
93	94	97	100	9	0.00	2.09200	3	;	O93-	C94-	C97-	O100
93	94	97	100	9	0.00	3.76560	2	;	O93-	C94-	C97-	O100
94	97	100	101	9	0.00	0.33472	2	;	C94-	C97-	O100-	C101
94	97	100	101	9	0.00	3.13800	3	;	C94-	C97-	O100-	C101
94	97	100	101	9	180.00	3.68192	1	;	C94-	C97-	O100-	C101
95	94	97	98	9	0.00	0.65084	3	;	H95-	C94-	C97-	H98
95	94	97	99	9	0.00	0.65084	3	;	H95-	C94-	C97-	H99
95	94	97	100	9	0.00	0.00000	0	;	H95-	C94-	C97-	O100
95	94	97	100	9	0.00	1.04600	1	;	H95-	C94-	C97-	O100
96	94	97	98	9	0.00	0.65084	3	;	H96-	C94-	C97-	H98
96	94	97	99	9	0.00	0.65084	3	;	H96-	C94-	C97-	H99
96	94	97	100	9	0.00	0.00000	0	;	H96-	C94-	C97-	O100
96	94	97	100	9	0.00	1.04600	1	;	H96-	C94-	C97-	O100
97	100	101	102	9	0.00	1.41001	3	;	C97-	O100-	C101-	H102
97	100	101	103	9	0.00	1.41001	3	;	C97-	O100-	C101-	H103
97	100	101	104	9	0.00	0.33472	2	;	C97-	O100-	C101-	C104
97	100	101	104	9	0.00	3.13800	3	;	C97-	O100-	C101-	C104
97	100	101	104	9	180.00	3.68192	1	;	C97-	O100-	C101-	C104
98	97	100	101	9	0.00	1.41001	3	;	H98-	C97-	O100-	C101
99	97	100	101	9	0.00	1.41001	3	;	H99-	C97-	O100-	C101
100	101	104	105	9	0.00	0.00000	0	;	O100-	C101-	C104-	H105
100	101	104	105	9	0.00	1.04600	1	;	O100-	C101-	C104-	H105
100	101	104	106	9	0.00	0.00000	0	;	O100-	C101-	C104-	H106
100	101	104	106	9	0.00	1.04600	1	;	O100-	C101-	C104-	H106
100	101	104	107	9	0.00	0.75312	1	;	O100-	C101-	C104-	O107
100	101	104	107	9	0.00	2.09200	3	;	O100-	C101-	C104-	O107
100	101	104	107	9	0.00	3.76560	2	;	O100-	C101-	C104-	O107
101	104	107	108	9	0.00	0.33472	2	;	C101-	C104-	O107-	C108

101	104	107	108	9	0.00	3.13800	3	;	C101-	C104-	O107-	C108
101	104	107	108	9	180.00	3.68192	1	;	C101-	C104-	O107-	C108
102	101	104	105	9	0.00	0.65084	3	;	H102-	C101-	C104-	H105
102	101	104	106	9	0.00	0.65084	3	;	H102-	C101-	C104-	H106
102	101	104	107	9	0.00	0.00000	0	;	H102-	C101-	C104-	O107
102	101	104	107	9	0.00	1.04600	1	;	H102-	C101-	C104-	O107
103	101	104	105	9	0.00	0.65084	3	;	H103-	C101-	C104-	H105
103	101	104	106	9	0.00	0.65084	3	;	H103-	C101-	C104-	H106
103	101	104	107	9	0.00	0.00000	0	;	H103-	C101-	C104-	O107
103	101	104	107	9	0.00	1.04600	1	;	H103-	C101-	C104-	O107
104	107	108	109	9	0.00	1.41001	3	;	C104-	O107-	C108-	H109
104	107	108	110	9	0.00	1.41001	3	;	C104-	O107-	C108-	H110
104	107	108	111	9	0.00	0.33472	2	;	C104-	O107-	C108-	C111
104	107	108	111	9	0.00	3.13800	3	;	C104-	O107-	C108-	C111
104	107	108	111	9	180.00	3.68192	1	;	C104-	O107-	C108-	C111
105	104	107	108	9	0.00	1.41001	3	;	H105-	C104-	O107-	C108
106	104	107	108	9	0.00	1.41001	3	;	H106-	C104-	O107-	C108
107	108	111	112	9	0.00	0.00000	0	;	O107-	C108-	C111-	H112
107	108	111	112	9	0.00	1.04600	1	;	O107-	C108-	C111-	H112
107	108	111	113	9	0.00	0.00000	0	;	O107-	C108-	C111-	H113
107	108	111	113	9	0.00	1.04600	1	;	O107-	C108-	C111-	H113
107	108	111	114	9	0.00	0.75312	1	;	O107-	C108-	C111-	O114
107	108	111	114	9	0.00	2.09200	3	;	O107-	C108-	C111-	O114
107	108	111	114	9	0.00	3.76560	2	;	O107-	C108-	C111-	O114
108	111	114	115	9	0.00	0.33472	2	;	C108-	C111-	O114-	C115
108	111	114	115	9	0.00	3.13800	3	;	C108-	C111-	O114-	C115
108	111	114	115	9	180.00	3.68192	1	;	C108-	C111-	O114-	C115
109	108	111	112	9	0.00	0.65084	3	;	H109-	C108-	C111-	H112
109	108	111	113	9	0.00	0.65084	3	;	H109-	C108-	C111-	H113

109	108	111	114	9	0.00	0.00000	0	;	H109-	C108-	C111-	O114
109	108	111	114	9	0.00	1.04600	1	;	H109-	C108-	C111-	O114
110	108	111	112	9	0.00	0.65084	3	;	H110-	C108-	C111-	H112
110	108	111	113	9	0.00	0.65084	3	;	H110-	C108-	C111-	H113
110	108	111	114	9	0.00	0.00000	0	;	H110-	C108-	C111-	O114
110	108	111	114	9	0.00	1.04600	1	;	H110-	C108-	C111-	O114
111	114	115	116	9	0.00	1.41001	3	;	C111-	O114-	C115-	H116
111	114	115	117	9	0.00	1.41001	3	;	C111-	O114-	C115-	H117
111	114	115	118	9	0.00	0.33472	2	;	C111-	O114-	C115-	C118
111	114	115	118	9	0.00	3.13800	3	;	C111-	O114-	C115-	C118
111	114	115	118	9	180.00	3.68192	1	;	C111-	O114-	C115-	C118
112	111	114	115	9	0.00	1.41001	3	;	H112-	C111-	O114-	C115
113	111	114	115	9	0.00	1.41001	3	;	H113-	C111-	O114-	C115
114	115	118	119	9	0.00	0.00000	0	;	O114-	C115-	C118-	H119
114	115	118	119	9	0.00	1.04600	1	;	O114-	C115-	C118-	H119
114	115	118	120	9	0.00	0.00000	0	;	O114-	C115-	C118-	H120
114	115	118	120	9	0.00	1.04600	1	;	O114-	C115-	C118-	H120
114	115	118	121	9	0.00	0.75312	1	;	O114-	C115-	C118-	O121
114	115	118	121	9	0.00	2.09200	3	;	O114-	C115-	C118-	O121
114	115	118	121	9	0.00	3.76560	2	;	O114-	C115-	C118-	O121
115	118	121	122	9	0.00	0.33472	2	;	C115-	C118-	O121-	C122
115	118	121	122	9	0.00	3.13800	3	;	C115-	C118-	O121-	C122
115	118	121	122	9	180.00	3.68192	1	;	C115-	C118-	O121-	C122
116	115	118	119	9	0.00	0.65084	3	;	H116-	C115-	C118-	H119
116	115	118	120	9	0.00	0.65084	3	;	H116-	C115-	C118-	H120
116	115	118	121	9	0.00	0.00000	0	;	H116-	C115-	C118-	O121
116	115	118	121	9	0.00	1.04600	1	;	H116-	C115-	C118-	O121
117	115	118	119	9	0.00	0.65084	3	;	H117-	C115-	C118-	H119
117	115	118	120	9	0.00	0.65084	3	;	H117-	C115-	C118-	H120

117	115	118	121	9	0.00	0.00000	0 ;	H117- C115- C118- O121
117	115	118	121	9	0.00	1.04600	1 ;	H117- C115- C118- O121
118	121	122	123	9	0.00	1.41001	3 ;	C118- O121- C122- H123
118	121	122	124	9	0.00	1.41001	3 ;	C118- O121- C122- H124
118	121	122	125	9	0.00	0.33472	2 ;	C118- O121- C122- C125
118	121	122	125	9	0.00	3.13800	3 ;	C118- O121- C122- C125
118	121	122	125	9	180.00	3.68192	1 ;	C118- O121- C122- C125
119	118	121	122	9	0.00	1.41001	3 ;	H119- C118- O121- C122
120	118	121	122	9	0.00	1.41001	3 ;	H120- C118- O121- C122
121	122	125	126	9	0.00	0.00000	0 ;	O121- C122- C125- H126
121	122	125	126	9	0.00	1.04600	1 ;	O121- C122- C125- H126
121	122	125	127	9	0.00	0.00000	0 ;	O121- C122- C125- H127
121	122	125	127	9	0.00	1.04600	1 ;	O121- C122- C125- H127
121	122	125	128	9	0.00	0.75312	1 ;	O121- C122- C125- O128
121	122	125	128	9	0.00	2.09200	3 ;	O121- C122- C125- O128
121	122	125	128	9	0.00	3.76560	2 ;	O121- C122- C125- O128
122	125	128	129	9	0.00	0.33472	2 ;	C122- C125- O128- C129
122	125	128	129	9	0.00	3.13800	3 ;	C122- C125- O128- C129
122	125	128	129	9	180.00	3.68192	1 ;	C122- C125- O128- C129
123	122	125	126	9	0.00	0.65084	3 ;	H123- C122- C125- H126
123	122	125	127	9	0.00	0.65084	3 ;	H123- C122- C125- H127
123	122	125	128	9	0.00	0.00000	0 ;	H123- C122- C125- O128
123	122	125	128	9	0.00	1.04600	1 ;	H123- C122- C125- O128
124	122	125	126	9	0.00	0.65084	3 ;	H124- C122- C125- H126
124	122	125	127	9	0.00	0.65084	3 ;	H124- C122- C125- H127
124	122	125	128	9	0.00	0.00000	0 ;	H124- C122- C125- O128
124	122	125	128	9	0.00	1.04600	1 ;	H124- C122- C125- O128
125	128	129	130	9	0.00	1.41001	3 ;	C125- O128- C129- H130
125	128	129	131	9	0.00	1.41001	3 ;	C125- O128- C129- H131

125	128	129	132	9	0.00	0.33472	2 ;	C125- O128- C129- C132
125	128	129	132	9	0.00	3.13800	3 ;	C125- O128- C129- C132
125	128	129	132	9	180.00	3.68192	1 ;	C125- O128- C129- C132
126	125	128	129	9	0.00	1.41001	3 ;	H126- C125- O128- C129
127	125	128	129	9	0.00	1.41001	3 ;	H127- C125- O128- C129
128	129	132	133	9	0.00	0.00000	0 ;	O128- C129- C132- H133
128	129	132	133	9	0.00	1.04600	1 ;	O128- C129- C132- H133
128	129	132	134	9	0.00	0.00000	0 ;	O128- C129- C132- H134
128	129	132	134	9	0.00	1.04600	1 ;	O128- C129- C132- H134
128	129	132	135	9	0.00	0.75312	1 ;	O128- C129- C132- O135
128	129	132	135	9	0.00	2.09200	3 ;	O128- C129- C132- O135
128	129	132	135	9	0.00	3.76560	2 ;	O128- C129- C132- O135
129	132	135	136	9	0.00	0.33472	2 ;	C129- C132- O135- C136
129	132	135	136	9	0.00	3.13800	3 ;	C129- C132- O135- C136
129	132	135	136	9	180.00	3.68192	1 ;	C129- C132- O135- C136
130	129	132	133	9	0.00	0.65084	3 ;	H130- C129- C132- H133
130	129	132	134	9	0.00	0.65084	3 ;	H130- C129- C132- H134
130	129	132	135	9	0.00	0.00000	0 ;	H130- C129- C132- O135
130	129	132	135	9	0.00	1.04600	1 ;	H130- C129- C132- O135
131	129	132	133	9	0.00	0.65084	3 ;	H131- C129- C132- H133
131	129	132	134	9	0.00	0.65084	3 ;	H131- C129- C132- H134
131	129	132	135	9	0.00	0.00000	0 ;	H131- C129- C132- O135
131	129	132	135	9	0.00	1.04600	1 ;	H131- C129- C132- O135
132	135	136	137	9	0.00	1.41001	3 ;	C132- O135- C136- H137
132	135	136	138	9	0.00	1.41001	3 ;	C132- O135- C136- H138
132	135	136	139	9	0.00	0.33472	2 ;	C132- O135- C136- C139
132	135	136	139	9	0.00	3.13800	3 ;	C132- O135- C136- C139
132	135	136	139	9	180.00	3.68192	1 ;	C132- O135- C136- C139
133	132	135	136	9	0.00	1.41001	3 ;	H133- C132- O135- C136

134	132	135	136	9	0.00	1.41001	3 ;	H134-	C132-	O135-	C136
135	136	139	140	9	0.00	0.00000	0 ;	O135-	C136-	C139-	H140
135	136	139	140	9	0.00	1.04600	1 ;	O135-	C136-	C139-	H140
135	136	139	141	9	0.00	0.00000	0 ;	O135-	C136-	C139-	H141
135	136	139	141	9	0.00	1.04600	1 ;	O135-	C136-	C139-	H141
135	136	139	142	9	0.00	0.75312	1 ;	O135-	C136-	C139-	O142
135	136	139	142	9	0.00	2.09200	3 ;	O135-	C136-	C139-	O142
135	136	139	142	9	0.00	3.76560	2 ;	O135-	C136-	C139-	O142
136	139	142	143	9	0.00	0.33472	2 ;	C136-	C139-	O142-	C143
136	139	142	143	9	0.00	3.13800	3 ;	C136-	C139-	O142-	C143
136	139	142	143	9	180.00	3.68192	1 ;	C136-	C139-	O142-	C143
137	136	139	140	9	0.00	0.65084	3 ;	H137-	C136-	C139-	H140
137	136	139	141	9	0.00	0.65084	3 ;	H137-	C136-	C139-	H141
137	136	139	142	9	0.00	0.00000	0 ;	H137-	C136-	C139-	O142
137	136	139	142	9	0.00	1.04600	1 ;	H137-	C136-	C139-	O142
138	136	139	140	9	0.00	0.65084	3 ;	H138-	C136-	C139-	H140
138	136	139	141	9	0.00	0.65084	3 ;	H138-	C136-	C139-	H141
138	136	139	142	9	0.00	0.00000	0 ;	H138-	C136-	C139-	O142
138	136	139	142	9	0.00	1.04600	1 ;	H138-	C136-	C139-	O142
139	142	143	144	9	0.00	1.41001	3 ;	C139-	O142-	C143-	H144
139	142	143	145	9	0.00	1.41001	3 ;	C139-	O142-	C143-	H145
139	142	143	146	9	0.00	0.33472	2 ;	C139-	O142-	C143-	C146
139	142	143	146	9	0.00	3.13800	3 ;	C139-	O142-	C143-	C146
139	142	143	146	9	180.00	3.68192	1 ;	C139-	O142-	C143-	C146
140	139	142	143	9	0.00	1.41001	3 ;	H140-	C139-	O142-	C143
141	139	142	143	9	0.00	1.41001	3 ;	H141-	C139-	O142-	C143
142	143	146	147	9	0.00	0.00000	0 ;	O142-	C143-	C146-	H147
142	143	146	147	9	0.00	1.04600	1 ;	O142-	C143-	C146-	H147
142	143	146	148	9	0.00	0.00000	0 ;	O142-	C143-	C146-	H148

142	143	146	148	9	0.00	1.04600	1 ;	O142- C143- C146- H148
142	143	146	149	9	0.00	0.75312	1 ;	O142- C143- C146- O149
142	143	146	149	9	0.00	2.09200	3 ;	O142- C143- C146- O149
142	143	146	149	9	0.00	3.76560	2 ;	O142- C143- C146- O149
143	146	149	150	9	0.00	0.33472	2 ;	C143- C146- O149- C150
143	146	149	150	9	0.00	3.13800	3 ;	C143- C146- O149- C150
143	146	149	150	9	180.00	3.68192	1 ;	C143- C146- O149- C150
144	143	146	147	9	0.00	0.65084	3 ;	H144- C143- C146- H147
144	143	146	148	9	0.00	0.65084	3 ;	H144- C143- C146- H148
144	143	146	149	9	0.00	0.00000	0 ;	H144- C143- C146- O149
144	143	146	149	9	0.00	1.04600	1 ;	H144- C143- C146- O149
145	143	146	147	9	0.00	0.65084	3 ;	H145- C143- C146- H147
145	143	146	148	9	0.00	0.65084	3 ;	H145- C143- C146- H148
145	143	146	149	9	0.00	0.00000	0 ;	H145- C143- C146- O149
145	143	146	149	9	0.00	1.04600	1 ;	H145- C143- C146- O149
146	149	150	151	9	0.00	1.41001	3 ;	C146- O149- C150- H151
146	149	150	152	9	0.00	1.41001	3 ;	C146- O149- C150- H152
146	149	150	153	9	0.00	0.33472	2 ;	C146- O149- C150- C153
146	149	150	153	9	0.00	3.13800	3 ;	C146- O149- C150- C153
146	149	150	153	9	180.00	3.68192	1 ;	C146- O149- C150- C153
147	146	149	150	9	0.00	1.41001	3 ;	H147- C146- O149- C150
148	146	149	150	9	0.00	1.41001	3 ;	H148- C146- O149- C150
149	150	153	154	9	0.00	0.00000	0 ;	O149- C150- C153- H154
149	150	153	154	9	0.00	1.04600	1 ;	O149- C150- C153- H154
149	150	153	155	9	0.00	0.00000	0 ;	O149- C150- C153- H155
149	150	153	155	9	0.00	1.04600	1 ;	O149- C150- C153- H155
149	150	153	156	9	0.00	0.75312	1 ;	O149- C150- C153- O156
149	150	153	156	9	0.00	2.09200	3 ;	O149- C150- C153- O156
149	150	153	156	9	0.00	3.76560	2 ;	O149- C150- C153- O156

150	153	156	157	9	0.00	0.33472	2 ;	C150- C153- O156- C157
150	153	156	157	9	0.00	3.13800	3 ;	C150- C153- O156- C157
150	153	156	157	9	180.00	3.68192	1 ;	C150- C153- O156- C157
151	150	153	154	9	0.00	0.65084	3 ;	H151- C150- C153- H154
151	150	153	155	9	0.00	0.65084	3 ;	H151- C150- C153- H155
151	150	153	156	9	0.00	0.00000	0 ;	H151- C150- C153- O156
151	150	153	156	9	0.00	1.04600	1 ;	H151- C150- C153- O156
152	150	153	154	9	0.00	0.65084	3 ;	H152- C150- C153- H154
152	150	153	155	9	0.00	0.65084	3 ;	H152- C150- C153- H155
152	150	153	156	9	0.00	0.00000	0 ;	H152- C150- C153- O156
152	150	153	156	9	0.00	1.04600	1 ;	H152- C150- C153- O156
153	156	157	158	9	0.00	1.41001	3 ;	C153- O156- C157- H158
153	156	157	159	9	0.00	1.41001	3 ;	C153- O156- C157- H159
153	156	157	160	9	0.00	0.33472	2 ;	C153- O156- C157- C160
153	156	157	160	9	0.00	3.13800	3 ;	C153- O156- C157- C160
153	156	157	160	9	180.00	3.68192	1 ;	C153- O156- C157- C160
154	153	156	157	9	0.00	1.41001	3 ;	H154- C153- O156- C157
155	153	156	157	9	0.00	1.41001	3 ;	H155- C153- O156- C157
156	157	160	161	9	0.00	0.00000	0 ;	O156- C157- C160- H161
156	157	160	161	9	0.00	1.04600	1 ;	O156- C157- C160- H161
156	157	160	162	9	0.00	0.00000	0 ;	O156- C157- C160- H162
156	157	160	162	9	0.00	1.04600	1 ;	O156- C157- C160- H162
156	157	160	163	9	0.00	0.75312	1 ;	O156- C157- C160- O163
156	157	160	163	9	0.00	2.09200	3 ;	O156- C157- C160- O163
156	157	160	163	9	0.00	3.76560	2 ;	O156- C157- C160- O163
157	160	163	164	9	0.00	0.33472	2 ;	C157- C160- O163- C164
157	160	163	164	9	0.00	3.13800	3 ;	C157- C160- O163- C164
157	160	163	164	9	180.00	3.68192	1 ;	C157- C160- O163- C164
158	157	160	161	9	0.00	0.65084	3 ;	H158- C157- C160- H161

158	157	160	162	9	0.00	0.65084	3	;	H158-	C157-	C160-	H162
158	157	160	163	9	0.00	0.00000	0	;	H158-	C157-	C160-	O163
158	157	160	163	9	0.00	1.04600	1	;	H158-	C157-	C160-	O163
159	157	160	161	9	0.00	0.65084	3	;	H159-	C157-	C160-	H161
159	157	160	162	9	0.00	0.65084	3	;	H159-	C157-	C160-	H162
159	157	160	163	9	0.00	0.00000	0	;	H159-	C157-	C160-	O163
159	157	160	163	9	0.00	1.04600	1	;	H159-	C157-	C160-	O163
160	163	164	165	9	0.00	1.41001	3	;	C160-	O163-	C164-	H165
160	163	164	166	9	0.00	1.41001	3	;	C160-	O163-	C164-	H166
160	163	164	167	9	0.00	0.33472	2	;	C160-	O163-	C164-	C167
160	163	164	167	9	0.00	3.13800	3	;	C160-	O163-	C164-	C167
160	163	164	167	9	180.00	3.68192	1	;	C160-	O163-	C164-	C167
161	160	163	164	9	0.00	1.41001	3	;	H161-	C160-	O163-	C164
162	160	163	164	9	0.00	1.41001	3	;	H162-	C160-	O163-	C164
163	164	167	168	9	0.00	0.00000	0	;	O163-	C164-	C167-	H168
163	164	167	168	9	0.00	1.04600	1	;	O163-	C164-	C167-	H168
163	164	167	169	9	0.00	0.00000	0	;	O163-	C164-	C167-	H169
163	164	167	169	9	0.00	1.04600	1	;	O163-	C164-	C167-	H169
163	164	167	170	9	0.00	0.75312	1	;	O163-	C164-	C167-	O170
163	164	167	170	9	0.00	2.09200	3	;	O163-	C164-	C167-	O170
163	164	167	170	9	0.00	3.76560	2	;	O163-	C164-	C167-	O170
164	167	170	171	9	0.00	0.33472	2	;	C164-	C167-	O170-	C171
164	167	170	171	9	0.00	3.13800	3	;	C164-	C167-	O170-	C171
164	167	170	171	9	180.00	3.68192	1	;	C164-	C167-	O170-	C171
165	164	167	168	9	0.00	0.65084	3	;	H165-	C164-	C167-	H168
165	164	167	169	9	0.00	0.65084	3	;	H165-	C164-	C167-	H169
165	164	167	170	9	0.00	0.00000	0	;	H165-	C164-	C167-	O170
165	164	167	170	9	0.00	1.04600	1	;	H165-	C164-	C167-	O170
166	164	167	168	9	0.00	0.65084	3	;	H166-	C164-	C167-	H168

166	164	167	169	9	0.00	0.65084	3 ;	H166- C164- C167- H169
166	164	167	170	9	0.00	0.00000	0 ;	H166- C164- C167- O170
166	164	167	170	9	0.00	1.04600	1 ;	H166- C164- C167- O170
167	170	171	172	9	0.00	1.41001	3 ;	C167- O170- C171- H172
167	170	171	173	9	0.00	1.41001	3 ;	C167- O170- C171- H173
167	170	171	174	9	0.00	0.33472	2 ;	C167- O170- C171- C174
167	170	171	174	9	0.00	3.13800	3 ;	C167- O170- C171- C174
167	170	171	174	9	180.00	3.68192	1 ;	C167- O170- C171- C174
168	167	170	171	9	0.00	1.41001	3 ;	H168- C167- O170- C171
169	167	170	171	9	0.00	1.41001	3 ;	H169- C167- O170- C171
170	171	174	175	9	0.00	0.00000	0 ;	O170- C171- C174- H175
170	171	174	175	9	0.00	1.04600	1 ;	O170- C171- C174- H175
170	171	174	176	9	0.00	0.00000	0 ;	O170- C171- C174- H176
170	171	174	176	9	0.00	1.04600	1 ;	O170- C171- C174- H176
170	171	174	177	9	0.00	0.75312	1 ;	O170- C171- C174- O177
170	171	174	177	9	0.00	2.09200	3 ;	O170- C171- C174- O177
170	171	174	177	9	0.00	3.76560	2 ;	O170- C171- C174- O177
171	174	177	178	9	0.00	0.33472	2 ;	C171- C174- O177- C178
171	174	177	178	9	0.00	3.13800	3 ;	C171- C174- O177- C178
171	174	177	178	9	180.00	3.68192	1 ;	C171- C174- O177- C178
172	171	174	175	9	0.00	0.65084	3 ;	H172- C171- C174- H175
172	171	174	176	9	0.00	0.65084	3 ;	H172- C171- C174- H176
172	171	174	177	9	0.00	0.00000	0 ;	H172- C171- C174- O177
172	171	174	177	9	0.00	1.04600	1 ;	H172- C171- C174- O177
173	171	174	175	9	0.00	0.65084	3 ;	H173- C171- C174- H175
173	171	174	176	9	0.00	0.65084	3 ;	H173- C171- C174- H176
173	171	174	177	9	0.00	0.00000	0 ;	H173- C171- C174- O177
173	171	174	177	9	0.00	1.04600	1 ;	H173- C171- C174- O177
174	177	178	179	9	0.00	1.41001	3 ;	C174- O177- C178- H179

174	177	178	180	9	0.00	1.41001	3	;	C174-	O177-	C178-	H180
174	177	178	181	9	0.00	0.33472	2	;	C174-	O177-	C178-	C181
174	177	178	181	9	0.00	3.13800	3	;	C174-	O177-	C178-	C181
174	177	178	181	9	180.00	3.68192	1	;	C174-	O177-	C178-	C181
175	174	177	178	9	0.00	1.41001	3	;	H175-	C174-	O177-	C178
176	174	177	178	9	0.00	1.41001	3	;	H176-	C174-	O177-	C178
177	178	181	182	9	0.00	0.00000	0	;	O177-	C178-	C181-	H182
177	178	181	182	9	0.00	1.04600	1	;	O177-	C178-	C181-	H182
177	178	181	183	9	0.00	0.00000	0	;	O177-	C178-	C181-	H183
177	178	181	183	9	0.00	1.04600	1	;	O177-	C178-	C181-	H183
177	178	181	184	9	0.00	0.75312	1	;	O177-	C178-	C181-	O184
177	178	181	184	9	0.00	2.09200	3	;	O177-	C178-	C181-	O184
177	178	181	184	9	0.00	3.76560	2	;	O177-	C178-	C181-	O184
178	181	184	185	9	0.00	0.33472	2	;	C178-	C181-	O184-	C185
178	181	184	185	9	0.00	3.13800	3	;	C178-	C181-	O184-	C185
178	181	184	185	9	180.00	3.68192	1	;	C178-	C181-	O184-	C185
179	178	181	182	9	0.00	0.65084	3	;	H179-	C178-	C181-	H182
179	178	181	183	9	0.00	0.65084	3	;	H179-	C178-	C181-	H183
179	178	181	184	9	0.00	0.00000	0	;	H179-	C178-	C181-	O184
179	178	181	184	9	0.00	1.04600	1	;	H179-	C178-	C181-	O184
180	178	181	182	9	0.00	0.65084	3	;	H180-	C178-	C181-	H182
180	178	181	183	9	0.00	0.65084	3	;	H180-	C178-	C181-	H183
180	178	181	184	9	0.00	0.00000	0	;	H180-	C178-	C181-	O184
180	178	181	184	9	0.00	1.04600	1	;	H180-	C178-	C181-	O184
181	184	185	186	9	0.00	1.41001	3	;	C181-	O184-	C185-	H186
181	184	185	187	9	0.00	1.41001	3	;	C181-	O184-	C185-	H187
181	184	185	188	9	0.00	0.33472	2	;	C181-	O184-	C185-	C188
181	184	185	188	9	0.00	3.13800	3	;	C181-	O184-	C185-	C188
181	184	185	188	9	180.00	3.68192	1	;	C181-	O184-	C185-	C188

182	181	184	185	9	0.00	1.41001	3	;	H182-	C181-	O184-	C185
183	181	184	185	9	0.00	1.41001	3	;	H183-	C181-	O184-	C185
184	185	188	189	9	0.00	0.00000	0	;	O184-	C185-	C188-	H189
184	185	188	189	9	0.00	1.04600	1	;	O184-	C185-	C188-	H189
184	185	188	190	9	0.00	0.00000	0	;	O184-	C185-	C188-	H190
184	185	188	190	9	0.00	1.04600	1	;	O184-	C185-	C188-	H190
184	185	188	191	9	0.00	0.75312	1	;	O184-	C185-	C188-	O191
184	185	188	191	9	0.00	2.09200	3	;	O184-	C185-	C188-	O191
184	185	188	191	9	0.00	3.76560	2	;	O184-	C185-	C188-	O191
185	188	191	192	9	0.00	0.33472	2	;	C185-	C188-	O191-	C192
185	188	191	192	9	0.00	3.13800	3	;	C185-	C188-	O191-	C192
185	188	191	192	9	180.00	3.68192	1	;	C185-	C188-	O191-	C192
186	185	188	189	9	0.00	0.65084	3	;	H186-	C185-	C188-	H189
186	185	188	190	9	0.00	0.65084	3	;	H186-	C185-	C188-	H190
186	185	188	191	9	0.00	0.00000	0	;	H186-	C185-	C188-	O191
186	185	188	191	9	0.00	1.04600	1	;	H186-	C185-	C188-	O191
187	185	188	189	9	0.00	0.65084	3	;	H187-	C185-	C188-	H189
187	185	188	190	9	0.00	0.65084	3	;	H187-	C185-	C188-	H190
187	185	188	191	9	0.00	0.00000	0	;	H187-	C185-	C188-	O191
187	185	188	191	9	0.00	1.04600	1	;	H187-	C185-	C188-	O191
188	191	192	193	9	0.00	1.41001	3	;	C188-	O191-	C192-	H193
188	191	192	194	9	0.00	1.41001	3	;	C188-	O191-	C192-	H194
188	191	192	195	9	0.00	0.33472	2	;	C188-	O191-	C192-	C195
188	191	192	195	9	0.00	3.13800	3	;	C188-	O191-	C192-	C195
188	191	192	195	9	180.00	3.68192	1	;	C188-	O191-	C192-	C195
189	188	191	192	9	0.00	1.41001	3	;	H189-	C188-	O191-	C192
190	188	191	192	9	0.00	1.41001	3	;	H190-	C188-	O191-	C192
191	192	195	196	9	0.00	0.00000	0	;	O191-	C192-	C195-	H196
191	192	195	196	9	0.00	1.04600	1	;	O191-	C192-	C195-	H196

191	192	195	197	9	0.00	0.00000	0 ;	O191-	C192-	C195-	H197
191	192	195	197	9	0.00	1.04600	1 ;	O191-	C192-	C195-	H197
191	192	195	198	9	0.00	0.75312	1 ;	O191-	C192-	C195-	O198
191	192	195	198	9	0.00	2.09200	3 ;	O191-	C192-	C195-	O198
191	192	195	198	9	0.00	3.76560	2 ;	O191-	C192-	C195-	O198
192	195	198	199	9	0.00	0.33472	2 ;	C192-	C195-	O198-	C199
192	195	198	199	9	0.00	3.13800	3 ;	C192-	C195-	O198-	C199
192	195	198	199	9	180.00	3.68192	1 ;	C192-	C195-	O198-	C199
193	192	195	196	9	0.00	0.65084	3 ;	H193-	C192-	C195-	H196
193	192	195	197	9	0.00	0.65084	3 ;	H193-	C192-	C195-	H197
193	192	195	198	9	0.00	0.00000	0 ;	H193-	C192-	C195-	O198
193	192	195	198	9	0.00	1.04600	1 ;	H193-	C192-	C195-	O198
194	192	195	196	9	0.00	0.65084	3 ;	H194-	C192-	C195-	H196
194	192	195	197	9	0.00	0.65084	3 ;	H194-	C192-	C195-	H197
194	192	195	198	9	0.00	0.00000	0 ;	H194-	C192-	C195-	O198
194	192	195	198	9	0.00	1.04600	1 ;	H194-	C192-	C195-	O198
195	198	199	200	9	0.00	1.41001	3 ;	C195-	O198-	C199-	H200
195	198	199	201	9	0.00	1.41001	3 ;	C195-	O198-	C199-	H201
195	198	199	202	9	0.00	0.33472	2 ;	C195-	O198-	C199-	C202
195	198	199	202	9	0.00	3.13800	3 ;	C195-	O198-	C199-	C202
195	198	199	202	9	180.00	3.68192	1 ;	C195-	O198-	C199-	C202
196	195	198	199	9	0.00	1.41001	3 ;	H196-	C195-	O198-	C199
197	195	198	199	9	0.00	1.41001	3 ;	H197-	C195-	O198-	C199
198	199	202	203	9	0.00	0.00000	0 ;	O198-	C199-	C202-	H203
198	199	202	203	9	0.00	1.04600	1 ;	O198-	C199-	C202-	H203
198	199	202	204	9	0.00	0.00000	0 ;	O198-	C199-	C202-	H204
198	199	202	204	9	0.00	1.04600	1 ;	O198-	C199-	C202-	H204
198	199	202	205	9	0.00	0.75312	1 ;	O198-	C199-	C202-	O205
198	199	202	205	9	0.00	2.09200	3 ;	O198-	C199-	C202-	O205

198	199	202	205	9	0.00	3.76560	2	;	O198-	C199-	C202-	O205
199	202	205	206	9	0.00	0.33472	2	;	C199-	C202-	O205-	C206
199	202	205	206	9	0.00	3.13800	3	;	C199-	C202-	O205-	C206
199	202	205	206	9	180.00	3.68192	1	;	C199-	C202-	O205-	C206
200	199	202	203	9	0.00	0.65084	3	;	H200-	C199-	C202-	H203
200	199	202	204	9	0.00	0.65084	3	;	H200-	C199-	C202-	H204
200	199	202	205	9	0.00	0.00000	0	;	H200-	C199-	C202-	O205
200	199	202	205	9	0.00	1.04600	1	;	H200-	C199-	C202-	O205
201	199	202	203	9	0.00	0.65084	3	;	H201-	C199-	C202-	H203
201	199	202	204	9	0.00	0.65084	3	;	H201-	C199-	C202-	H204
201	199	202	205	9	0.00	0.00000	0	;	H201-	C199-	C202-	O205
201	199	202	205	9	0.00	1.04600	1	;	H201-	C199-	C202-	O205
202	205	206	207	9	0.00	1.41001	3	;	C202-	O205-	C206-	H207
202	205	206	208	9	0.00	1.41001	3	;	C202-	O205-	C206-	H208
202	205	206	209	9	0.00	0.33472	2	;	C202-	O205-	C206-	C209
202	205	206	209	9	0.00	3.13800	3	;	C202-	O205-	C206-	C209
202	205	206	209	9	180.00	3.68192	1	;	C202-	O205-	C206-	C209
203	202	205	206	9	0.00	1.41001	3	;	H203-	C202-	O205-	C206
204	202	205	206	9	0.00	1.41001	3	;	H204-	C202-	O205-	C206
205	206	209	210	9	0.00	0.00000	0	;	O205-	C206-	C209-	H210
205	206	209	210	9	0.00	1.04600	1	;	O205-	C206-	C209-	H210
205	206	209	211	9	0.00	0.00000	0	;	O205-	C206-	C209-	H211
205	206	209	211	9	0.00	1.04600	1	;	O205-	C206-	C209-	H211
205	206	209	212	9	0.00	0.75312	1	;	O205-	C206-	C209-	O212
205	206	209	212	9	0.00	2.09200	3	;	O205-	C206-	C209-	O212
205	206	209	212	9	0.00	3.76560	2	;	O205-	C206-	C209-	O212
206	209	212	213	9	0.00	0.33472	2	;	C206-	C209-	O212-	C213
206	209	212	213	9	0.00	3.13800	3	;	C206-	C209-	O212-	C213
206	209	212	213	9	180.00	3.68192	1	;	C206-	C209-	O212-	C213

207	206	209	210	9	0.00	0.65084	3	;	H207-	C206-	C209-	H210
207	206	209	211	9	0.00	0.65084	3	;	H207-	C206-	C209-	H211
207	206	209	212	9	0.00	0.00000	0	;	H207-	C206-	C209-	O212
207	206	209	212	9	0.00	1.04600	1	;	H207-	C206-	C209-	O212
208	206	209	210	9	0.00	0.65084	3	;	H208-	C206-	C209-	H210
208	206	209	211	9	0.00	0.65084	3	;	H208-	C206-	C209-	H211
208	206	209	212	9	0.00	0.00000	0	;	H208-	C206-	C209-	O212
208	206	209	212	9	0.00	1.04600	1	;	H208-	C206-	C209-	O212
209	212	213	214	9	0.00	1.41001	3	;	C209-	O212-	C213-	H214
209	212	213	215	9	0.00	1.41001	3	;	C209-	O212-	C213-	H215
209	212	213	216	9	0.00	0.33472	2	;	C209-	O212-	C213-	C216
209	212	213	216	9	0.00	3.13800	3	;	C209-	O212-	C213-	C216
209	212	213	216	9	180.00	3.68192	1	;	C209-	O212-	C213-	C216
210	209	212	213	9	0.00	1.41001	3	;	H210-	C209-	O212-	C213
211	209	212	213	9	0.00	1.41001	3	;	H211-	C209-	O212-	C213
212	213	216	217	9	0.00	0.00000	0	;	O212-	C213-	C216-	H217
212	213	216	217	9	0.00	1.04600	1	;	O212-	C213-	C216-	H217
212	213	216	218	9	0.00	0.00000	0	;	O212-	C213-	C216-	H218
212	213	216	218	9	0.00	1.04600	1	;	O212-	C213-	C216-	H218
212	213	216	219	9	0.00	0.75312	1	;	O212-	C213-	C216-	O219
212	213	216	219	9	0.00	2.09200	3	;	O212-	C213-	C216-	O219
212	213	216	219	9	0.00	3.76560	2	;	O212-	C213-	C216-	O219
213	216	219	220	9	0.00	0.33472	2	;	C213-	C216-	O219-	C220
213	216	219	220	9	0.00	3.13800	3	;	C213-	C216-	O219-	C220
213	216	219	220	9	180.00	3.68192	1	;	C213-	C216-	O219-	C220
214	213	216	217	9	0.00	0.65084	3	;	H214-	C213-	C216-	H217
214	213	216	218	9	0.00	0.65084	3	;	H214-	C213-	C216-	H218
214	213	216	219	9	0.00	0.00000	0	;	H214-	C213-	C216-	O219
214	213	216	219	9	0.00	1.04600	1	;	H214-	C213-	C216-	O219

215	213	216	217	9	0.00	0.65084	3 ;	H215- C213- C216- H217
215	213	216	218	9	0.00	0.65084	3 ;	H215- C213- C216- H218
215	213	216	219	9	0.00	0.00000	0 ;	H215- C213- C216- O219
215	213	216	219	9	0.00	1.04600	1 ;	H215- C213- C216- O219
216	219	220	221	9	0.00	1.41001	3 ;	C216- O219- C220- H221
216	219	220	222	9	0.00	1.41001	3 ;	C216- O219- C220- H222
216	219	220	223	9	0.00	0.33472	2 ;	C216- O219- C220- C223
216	219	220	223	9	0.00	3.13800	3 ;	C216- O219- C220- C223
216	219	220	223	9	180.00	3.68192	1 ;	C216- O219- C220- C223
217	216	219	220	9	0.00	1.41001	3 ;	H217- C216- O219- C220
218	216	219	220	9	0.00	1.41001	3 ;	H218- C216- O219- C220
219	220	223	224	9	0.00	0.00000	0 ;	O219- C220- C223- H224
219	220	223	224	9	0.00	1.04600	1 ;	O219- C220- C223- H224
219	220	223	225	9	0.00	0.00000	0 ;	O219- C220- C223- H225
219	220	223	225	9	0.00	1.04600	1 ;	O219- C220- C223- H225
219	220	223	226	9	0.00	0.75312	1 ;	O219- C220- C223- O226
219	220	223	226	9	0.00	2.09200	3 ;	O219- C220- C223- O226
219	220	223	226	9	0.00	3.76560	2 ;	O219- C220- C223- O226
220	223	226	227	9	0.00	0.33472	2 ;	C220- C223- O226- C227
220	223	226	227	9	0.00	3.13800	3 ;	C220- C223- O226- C227
220	223	226	227	9	180.00	3.68192	1 ;	C220- C223- O226- C227
221	220	223	224	9	0.00	0.65084	3 ;	H221- C220- C223- H224
221	220	223	225	9	0.00	0.65084	3 ;	H221- C220- C223- H225
221	220	223	226	9	0.00	0.00000	0 ;	H221- C220- C223- O226
221	220	223	226	9	0.00	1.04600	1 ;	H221- C220- C223- O226
222	220	223	224	9	0.00	0.65084	3 ;	H222- C220- C223- H224
222	220	223	225	9	0.00	0.65084	3 ;	H222- C220- C223- H225
222	220	223	226	9	0.00	0.00000	0 ;	H222- C220- C223- O226
222	220	223	226	9	0.00	1.04600	1 ;	H222- C220- C223- O226

223	226	227	228	9	0.00	1.41001	3 ;	C223-	O226-	C227-	H228
223	226	227	229	9	0.00	1.41001	3 ;	C223-	O226-	C227-	H229
223	226	227	230	9	0.00	0.33472	2 ;	C223-	O226-	C227-	C230
223	226	227	230	9	0.00	3.13800	3 ;	C223-	O226-	C227-	C230
223	226	227	230	9	180.00	3.68192	1 ;	C223-	O226-	C227-	C230
224	223	226	227	9	0.00	1.41001	3 ;	H224-	C223-	O226-	C227
225	223	226	227	9	0.00	1.41001	3 ;	H225-	C223-	O226-	C227
226	227	230	231	9	0.00	0.00000	0 ;	O226-	C227-	C230-	H231
226	227	230	231	9	0.00	1.04600	1 ;	O226-	C227-	C230-	H231
226	227	230	232	9	0.00	0.00000	0 ;	O226-	C227-	C230-	H232
226	227	230	232	9	0.00	1.04600	1 ;	O226-	C227-	C230-	H232
226	227	230	233	9	0.00	0.75312	1 ;	O226-	C227-	C230-	O233
226	227	230	233	9	0.00	2.09200	3 ;	O226-	C227-	C230-	O233
226	227	230	233	9	0.00	3.76560	2 ;	O226-	C227-	C230-	O233
227	230	233	234	9	0.00	0.33472	2 ;	C227-	C230-	O233-	C234
227	230	233	234	9	0.00	3.13800	3 ;	C227-	C230-	O233-	C234
227	230	233	234	9	180.00	3.68192	1 ;	C227-	C230-	O233-	C234
228	227	230	231	9	0.00	0.65084	3 ;	H228-	C227-	C230-	H231
228	227	230	232	9	0.00	0.65084	3 ;	H228-	C227-	C230-	H232
228	227	230	233	9	0.00	0.00000	0 ;	H228-	C227-	C230-	O233
228	227	230	233	9	0.00	1.04600	1 ;	H228-	C227-	C230-	O233
229	227	230	231	9	0.00	0.65084	3 ;	H229-	C227-	C230-	H231
229	227	230	232	9	0.00	0.65084	3 ;	H229-	C227-	C230-	H232
229	227	230	233	9	0.00	0.00000	0 ;	H229-	C227-	C230-	O233
229	227	230	233	9	0.00	1.04600	1 ;	H229-	C227-	C230-	O233
230	233	234	235	9	0.00	1.41001	3 ;	C230-	O233-	C234-	H235
230	233	234	236	9	0.00	1.41001	3 ;	C230-	O233-	C234-	H236
230	233	234	237	9	0.00	0.33472	2 ;	C230-	O233-	C234-	C237
230	233	234	237	9	0.00	3.13800	3 ;	C230-	O233-	C234-	C237

230	233	234	237	9	180.00	3.68192	1 ;	C230-	O233-	C234-	C237
231	230	233	234	9	0.00	1.41001	3 ;	H231-	C230-	O233-	C234
232	230	233	234	9	0.00	1.41001	3 ;	H232-	C230-	O233-	C234
233	234	237	238	9	0.00	0.00000	0 ;	O233-	C234-	C237-	H238
233	234	237	238	9	0.00	1.04600	1 ;	O233-	C234-	C237-	H238
233	234	237	239	9	0.00	0.00000	0 ;	O233-	C234-	C237-	H239
233	234	237	239	9	0.00	1.04600	1 ;	O233-	C234-	C237-	H239
233	234	237	240	9	0.00	0.75312	1 ;	O233-	C234-	C237-	O240
233	234	237	240	9	0.00	2.09200	3 ;	O233-	C234-	C237-	O240
233	234	237	240	9	0.00	3.76560	2 ;	O233-	C234-	C237-	O240
234	237	240	241	9	0.00	0.33472	2 ;	C234-	C237-	O240-	C241
234	237	240	241	9	0.00	3.13800	3 ;	C234-	C237-	O240-	C241
234	237	240	241	9	180.00	3.68192	1 ;	C234-	C237-	O240-	C241
235	234	237	238	9	0.00	0.65084	3 ;	H235-	C234-	C237-	H238
235	234	237	239	9	0.00	0.65084	3 ;	H235-	C234-	C237-	H239
235	234	237	240	9	0.00	0.00000	0 ;	H235-	C234-	C237-	O240
235	234	237	240	9	0.00	1.04600	1 ;	H235-	C234-	C237-	O240
236	234	237	238	9	0.00	0.65084	3 ;	H236-	C234-	C237-	H238
236	234	237	239	9	0.00	0.65084	3 ;	H236-	C234-	C237-	H239
236	234	237	240	9	0.00	0.00000	0 ;	H236-	C234-	C237-	O240
236	234	237	240	9	0.00	1.04600	1 ;	H236-	C234-	C237-	O240
237	240	241	242	9	0.00	1.41001	3 ;	C237-	O240-	C241-	H242
237	240	241	243	9	0.00	1.41001	3 ;	C237-	O240-	C241-	H243
237	240	241	244	9	0.00	0.33472	2 ;	C237-	O240-	C241-	C244
237	240	241	244	9	0.00	3.13800	3 ;	C237-	O240-	C241-	C244
237	240	241	244	9	180.00	3.68192	1 ;	C237-	O240-	C241-	C244
238	237	240	241	9	0.00	1.41001	3 ;	H238-	C237-	O240-	C241
239	237	240	241	9	0.00	1.41001	3 ;	H239-	C237-	O240-	C241
240	241	244	245	9	0.00	0.00000	0 ;	O240-	C241-	C244-	H245

240	241	244	245	9	0.00	1.04600	1	;	O240-	C241-	C244-	H245
240	241	244	246	9	0.00	0.00000	0	;	O240-	C241-	C244-	H246
240	241	244	246	9	0.00	1.04600	1	;	O240-	C241-	C244-	H246
240	241	244	247	9	0.00	0.75312	1	;	O240-	C241-	C244-	O247
240	241	244	247	9	0.00	2.09200	3	;	O240-	C241-	C244-	O247
240	241	244	247	9	0.00	3.76560	2	;	O240-	C241-	C244-	O247
241	244	247	248	9	0.00	0.33472	2	;	C241-	C244-	O247-	C248
241	244	247	248	9	0.00	3.13800	3	;	C241-	C244-	O247-	C248
241	244	247	248	9	180.00	3.68192	1	;	C241-	C244-	O247-	C248
242	241	244	245	9	0.00	0.65084	3	;	H242-	C241-	C244-	H245
242	241	244	246	9	0.00	0.65084	3	;	H242-	C241-	C244-	H246
242	241	244	247	9	0.00	0.00000	0	;	H242-	C241-	C244-	O247
242	241	244	247	9	0.00	1.04600	1	;	H242-	C241-	C244-	O247
243	241	244	245	9	0.00	0.65084	3	;	H243-	C241-	C244-	H245
243	241	244	246	9	0.00	0.65084	3	;	H243-	C241-	C244-	H246
243	241	244	247	9	0.00	0.00000	0	;	H243-	C241-	C244-	O247
243	241	244	247	9	0.00	1.04600	1	;	H243-	C241-	C244-	O247
244	247	248	249	9	0.00	1.41001	3	;	C244-	O247-	C248-	H249
244	247	248	250	9	0.00	1.41001	3	;	C244-	O247-	C248-	H250
244	247	248	251	9	0.00	0.33472	2	;	C244-	O247-	C248-	C251
244	247	248	251	9	0.00	3.13800	3	;	C244-	O247-	C248-	C251
244	247	248	251	9	180.00	3.68192	1	;	C244-	O247-	C248-	C251
245	244	247	248	9	0.00	1.41001	3	;	H245-	C244-	O247-	C248
246	244	247	248	9	0.00	1.41001	3	;	H246-	C244-	O247-	C248
247	248	251	252	9	0.00	0.00000	0	;	O247-	C248-	C251-	H252
247	248	251	252	9	0.00	1.04600	1	;	O247-	C248-	C251-	H252
247	248	251	253	9	0.00	0.00000	0	;	O247-	C248-	C251-	H253
247	248	251	253	9	0.00	1.04600	1	;	O247-	C248-	C251-	H253
247	248	251	254	9	0.00	0.75312	1	;	O247-	C248-	C251-	O254

247	248	251	254	9	0.00	2.09200	3	;	O247-	C248-	C251-	O254
247	248	251	254	9	0.00	3.76560	2	;	O247-	C248-	C251-	O254
248	251	254	255	9	0.00	0.33472	2	;	C248-	C251-	O254-	C255
248	251	254	255	9	0.00	3.13800	3	;	C248-	C251-	O254-	C255
248	251	254	255	9	180.00	3.68192	1	;	C248-	C251-	O254-	C255
249	248	251	252	9	0.00	0.65084	3	;	H249-	C248-	C251-	H252
249	248	251	253	9	0.00	0.65084	3	;	H249-	C248-	C251-	H253
249	248	251	254	9	0.00	0.00000	0	;	H249-	C248-	C251-	O254
249	248	251	254	9	0.00	1.04600	1	;	H249-	C248-	C251-	O254
250	248	251	252	9	0.00	0.65084	3	;	H250-	C248-	C251-	H252
250	248	251	253	9	0.00	0.65084	3	;	H250-	C248-	C251-	H253
250	248	251	254	9	0.00	0.00000	0	;	H250-	C248-	C251-	O254
250	248	251	254	9	0.00	1.04600	1	;	H250-	C248-	C251-	O254
251	254	255	256	9	0.00	1.41001	3	;	C251-	O254-	C255-	H256
251	254	255	257	9	0.00	1.41001	3	;	C251-	O254-	C255-	H257
251	254	255	258	9	0.00	0.33472	2	;	C251-	O254-	C255-	C258
251	254	255	258	9	0.00	3.13800	3	;	C251-	O254-	C255-	C258
251	254	255	258	9	180.00	3.68192	1	;	C251-	O254-	C255-	C258
252	251	254	255	9	0.00	1.41001	3	;	H252-	C251-	O254-	C255
253	251	254	255	9	0.00	1.41001	3	;	H253-	C251-	O254-	C255
254	255	258	259	9	0.00	0.00000	0	;	O254-	C255-	C258-	H259
254	255	258	259	9	0.00	1.04600	1	;	O254-	C255-	C258-	H259
254	255	258	260	9	0.00	0.00000	0	;	O254-	C255-	C258-	H260
254	255	258	260	9	0.00	1.04600	1	;	O254-	C255-	C258-	H260
254	255	258	261	9	0.00	0.75312	1	;	O254-	C255-	C258-	O261
254	255	258	261	9	0.00	2.09200	3	;	O254-	C255-	C258-	O261
254	255	258	261	9	0.00	3.76560	2	;	O254-	C255-	C258-	O261
255	258	261	262	9	0.00	0.33472	2	;	C255-	C258-	O261-	C262
255	258	261	262	9	0.00	3.13800	3	;	C255-	C258-	O261-	C262

255	258	261	262	9	180.00	3.68192	1 ;	C255-	C258-	O261-	C262
256	255	258	259	9	0.00	0.65084	3 ;	H256-	C255-	C258-	H259
256	255	258	260	9	0.00	0.65084	3 ;	H256-	C255-	C258-	H260
256	255	258	261	9	0.00	0.00000	0 ;	H256-	C255-	C258-	O261
256	255	258	261	9	0.00	1.04600	1 ;	H256-	C255-	C258-	O261
257	255	258	259	9	0.00	0.65084	3 ;	H257-	C255-	C258-	H259
257	255	258	260	9	0.00	0.65084	3 ;	H257-	C255-	C258-	H260
257	255	258	261	9	0.00	0.00000	0 ;	H257-	C255-	C258-	O261
257	255	258	261	9	0.00	1.04600	1 ;	H257-	C255-	C258-	O261
258	261	262	263	9	0.00	1.41001	3 ;	C258-	O261-	C262-	H263
258	261	262	264	9	0.00	1.41001	3 ;	C258-	O261-	C262-	H264
258	261	262	265	9	0.00	0.33472	2 ;	C258-	O261-	C262-	C265
258	261	262	265	9	0.00	3.13800	3 ;	C258-	O261-	C262-	C265
258	261	262	265	9	180.00	3.68192	1 ;	C258-	O261-	C262-	C265
259	258	261	262	9	0.00	1.41001	3 ;	H259-	C258-	O261-	C262
260	258	261	262	9	0.00	1.41001	3 ;	H260-	C258-	O261-	C262
261	262	265	266	9	0.00	0.00000	0 ;	O261-	C262-	C265-	H266
261	262	265	266	9	0.00	1.04600	1 ;	O261-	C262-	C265-	H266
261	262	265	267	9	0.00	0.00000	0 ;	O261-	C262-	C265-	H267
261	262	265	267	9	0.00	1.04600	1 ;	O261-	C262-	C265-	H267
261	262	265	268	9	0.00	0.75312	1 ;	O261-	C262-	C265-	O268
261	262	265	268	9	0.00	2.09200	3 ;	O261-	C262-	C265-	O268
261	262	265	268	9	0.00	3.76560	2 ;	O261-	C262-	C265-	O268
262	265	268	269	9	0.00	0.33472	2 ;	C262-	C265-	O268-	C269
262	265	268	269	9	0.00	3.13800	3 ;	C262-	C265-	O268-	C269
262	265	268	269	9	180.00	3.68192	1 ;	C262-	C265-	O268-	C269
263	262	265	266	9	0.00	0.65084	3 ;	H263-	C262-	C265-	H266
263	262	265	267	9	0.00	0.65084	3 ;	H263-	C262-	C265-	H267
263	262	265	268	9	0.00	0.00000	0 ;	H263-	C262-	C265-	O268

263	262	265	268	9	0.00	1.04600	1	;	H263-	C262-	C265-	O268
264	262	265	266	9	0.00	0.65084	3	;	H264-	C262-	C265-	H266
264	262	265	267	9	0.00	0.65084	3	;	H264-	C262-	C265-	H267
264	262	265	268	9	0.00	0.00000	0	;	H264-	C262-	C265-	O268
264	262	265	268	9	0.00	1.04600	1	;	H264-	C262-	C265-	O268
265	268	269	270	9	0.00	1.41001	3	;	C265-	O268-	C269-	H270
265	268	269	271	9	0.00	1.41001	3	;	C265-	O268-	C269-	H271
265	268	269	272	9	0.00	0.33472	2	;	C265-	O268-	C269-	C272
265	268	269	272	9	0.00	3.13800	3	;	C265-	O268-	C269-	C272
265	268	269	272	9	180.00	3.68192	1	;	C265-	O268-	C269-	C272
266	265	268	269	9	0.00	1.41001	3	;	H266-	C265-	O268-	C269
267	265	268	269	9	0.00	1.41001	3	;	H267-	C265-	O268-	C269
268	269	272	273	9	0.00	0.00000	0	;	O268-	C269-	C272-	H273
268	269	272	273	9	0.00	1.04600	1	;	O268-	C269-	C272-	H273
268	269	272	274	9	0.00	0.00000	0	;	O268-	C269-	C272-	H274
268	269	272	274	9	0.00	1.04600	1	;	O268-	C269-	C272-	H274
268	269	272	275	9	0.00	0.75312	1	;	O268-	C269-	C272-	O275
268	269	272	275	9	0.00	2.09200	3	;	O268-	C269-	C272-	O275
268	269	272	275	9	0.00	3.76560	2	;	O268-	C269-	C272-	O275
269	272	275	276	9	0.00	0.33472	2	;	C269-	C272-	O275-	C276
269	272	275	276	9	0.00	3.13800	3	;	C269-	C272-	O275-	C276
269	272	275	276	9	180.00	3.68192	1	;	C269-	C272-	O275-	C276
270	269	272	273	9	0.00	0.65084	3	;	H270-	C269-	C272-	H273
270	269	272	274	9	0.00	0.65084	3	;	H270-	C269-	C272-	H274
270	269	272	275	9	0.00	0.00000	0	;	H270-	C269-	C272-	O275
270	269	272	275	9	0.00	1.04600	1	;	H270-	C269-	C272-	O275
271	269	272	273	9	0.00	0.65084	3	;	H271-	C269-	C272-	H273
271	269	272	274	9	0.00	0.65084	3	;	H271-	C269-	C272-	H274
271	269	272	275	9	0.00	0.00000	0	;	H271-	C269-	C272-	O275

271	269	272	275	9	0.00	1.04600	1 ;	H271- C269- C272- O275
272	275	276	277	9	0.00	1.41001	3 ;	C272- O275- C276- H277
272	275	276	278	9	0.00	1.41001	3 ;	C272- O275- C276- H278
272	275	276	279	9	0.00	0.33472	2 ;	C272- O275- C276- C279
272	275	276	279	9	0.00	3.13800	3 ;	C272- O275- C276- C279
272	275	276	279	9	180.00	3.68192	1 ;	C272- O275- C276- C279
273	272	275	276	9	0.00	1.41001	3 ;	H273- C272- O275- C276
274	272	275	276	9	0.00	1.41001	3 ;	H274- C272- O275- C276
275	276	279	280	9	0.00	0.00000	0 ;	O275- C276- C279- H280
275	276	279	280	9	0.00	1.04600	1 ;	O275- C276- C279- H280
275	276	279	281	9	0.00	0.00000	0 ;	O275- C276- C279- H281
275	276	279	281	9	0.00	1.04600	1 ;	O275- C276- C279- H281
275	276	279	282	9	0.00	0.75312	1 ;	O275- C276- C279- O282
275	276	279	282	9	0.00	2.09200	3 ;	O275- C276- C279- O282
275	276	279	282	9	0.00	3.76560	2 ;	O275- C276- C279- O282
276	279	282	283	9	0.00	0.33472	2 ;	C276- C279- O282- C283
276	279	282	283	9	0.00	3.13800	3 ;	C276- C279- O282- C283
276	279	282	283	9	180.00	3.68192	1 ;	C276- C279- O282- C283
277	276	279	280	9	0.00	0.65084	3 ;	H277- C276- C279- H280
277	276	279	281	9	0.00	0.65084	3 ;	H277- C276- C279- H281
277	276	279	282	9	0.00	0.00000	0 ;	H277- C276- C279- O282
277	276	279	282	9	0.00	1.04600	1 ;	H277- C276- C279- O282
278	276	279	280	9	0.00	0.65084	3 ;	H278- C276- C279- H280
278	276	279	281	9	0.00	0.65084	3 ;	H278- C276- C279- H281
278	276	279	282	9	0.00	0.00000	0 ;	H278- C276- C279- O282
278	276	279	282	9	0.00	1.04600	1 ;	H278- C276- C279- O282
279	282	283	284	9	0.00	1.41001	3 ;	C279- O282- C283- H284
279	282	283	285	9	0.00	1.41001	3 ;	C279- O282- C283- H285
279	282	283	286	9	0.00	0.33472	2 ;	C279- O282- C283- C286

279	282	283	286	9	0.00	3.13800	3	;	C279-	O282-	C283-	C286
279	282	283	286	9	180.00	3.68192	1	;	C279-	O282-	C283-	C286
280	279	282	283	9	0.00	1.41001	3	;	H280-	C279-	O282-	C283
281	279	282	283	9	0.00	1.41001	3	;	H281-	C279-	O282-	C283
282	283	286	287	9	0.00	0.00000	0	;	O282-	C283-	C286-	H287
282	283	286	287	9	0.00	1.04600	1	;	O282-	C283-	C286-	H287
282	283	286	288	9	0.00	0.00000	0	;	O282-	C283-	C286-	H288
282	283	286	288	9	0.00	1.04600	1	;	O282-	C283-	C286-	H288
282	283	286	289	9	0.00	0.75312	1	;	O282-	C283-	C286-	O289
282	283	286	289	9	0.00	2.09200	3	;	O282-	C283-	C286-	O289
282	283	286	289	9	0.00	3.76560	2	;	O282-	C283-	C286-	O289
283	286	289	290	9	0.00	0.33472	2	;	C283-	C286-	O289-	C290
283	286	289	290	9	0.00	3.13800	3	;	C283-	C286-	O289-	C290
283	286	289	290	9	180.00	3.68192	1	;	C283-	C286-	O289-	C290
284	283	286	287	9	0.00	0.65084	3	;	H284-	C283-	C286-	H287
284	283	286	288	9	0.00	0.65084	3	;	H284-	C283-	C286-	H288
284	283	286	289	9	0.00	0.00000	0	;	H284-	C283-	C286-	O289
284	283	286	289	9	0.00	1.04600	1	;	H284-	C283-	C286-	O289
285	283	286	287	9	0.00	0.65084	3	;	H285-	C283-	C286-	H287
285	283	286	288	9	0.00	0.65084	3	;	H285-	C283-	C286-	H288
285	283	286	289	9	0.00	0.00000	0	;	H285-	C283-	C286-	O289
285	283	286	289	9	0.00	1.04600	1	;	H285-	C283-	C286-	O289
286	289	290	291	9	0.00	1.41001	3	;	C286-	O289-	C290-	H291
286	289	290	292	9	0.00	1.41001	3	;	C286-	O289-	C290-	H292
286	289	290	293	9	0.00	0.33472	2	;	C286-	O289-	C290-	C293
286	289	290	293	9	0.00	3.13800	3	;	C286-	O289-	C290-	C293
286	289	290	293	9	180.00	3.68192	1	;	C286-	O289-	C290-	C293
287	286	289	290	9	0.00	1.41001	3	;	H287-	C286-	O289-	C290
288	286	289	290	9	0.00	1.41001	3	;	H288-	C286-	O289-	C290

289	290	293	294	9	0.00	0.00000	0	;	O289-	C290-	C293-	H294
289	290	293	294	9	0.00	1.04600	1	;	O289-	C290-	C293-	H294
289	290	293	295	9	0.00	0.00000	0	;	O289-	C290-	C293-	H295
289	290	293	295	9	0.00	1.04600	1	;	O289-	C290-	C293-	H295
289	290	293	296	9	0.00	0.75312	1	;	O289-	C290-	C293-	O296
289	290	293	296	9	0.00	2.09200	3	;	O289-	C290-	C293-	O296
289	290	293	296	9	0.00	3.76560	2	;	O289-	C290-	C293-	O296
290	293	296	297	9	0.00	0.33472	2	;	C290-	C293-	O296-	C297
290	293	296	297	9	0.00	3.13800	3	;	C290-	C293-	O296-	C297
290	293	296	297	9	180.00	3.68192	1	;	C290-	C293-	O296-	C297
291	290	293	294	9	0.00	0.65084	3	;	H291-	C290-	C293-	H294
291	290	293	295	9	0.00	0.65084	3	;	H291-	C290-	C293-	H295
291	290	293	296	9	0.00	0.00000	0	;	H291-	C290-	C293-	O296
291	290	293	296	9	0.00	1.04600	1	;	H291-	C290-	C293-	O296
292	290	293	294	9	0.00	0.65084	3	;	H292-	C290-	C293-	H294
292	290	293	295	9	0.00	0.65084	3	;	H292-	C290-	C293-	H295
292	290	293	296	9	0.00	0.00000	0	;	H292-	C290-	C293-	O296
292	290	293	296	9	0.00	1.04600	1	;	H292-	C290-	C293-	O296
293	296	297	298	9	0.00	1.41001	3	;	C293-	O296-	C297-	H298
293	296	297	299	9	0.00	1.41001	3	;	C293-	O296-	C297-	H299
293	296	297	300	9	0.00	0.33472	2	;	C293-	O296-	C297-	C300
293	296	297	300	9	0.00	3.13800	3	;	C293-	O296-	C297-	C300
293	296	297	300	9	180.00	3.68192	1	;	C293-	O296-	C297-	C300
294	293	296	297	9	0.00	1.41001	3	;	H294-	C293-	O296-	C297
295	293	296	297	9	0.00	1.41001	3	;	H295-	C293-	O296-	C297
296	297	300	301	9	0.00	0.00000	0	;	O296-	C297-	C300-	H301
296	297	300	301	9	0.00	1.04600	1	;	O296-	C297-	C300-	H301
296	297	300	302	9	0.00	0.00000	0	;	O296-	C297-	C300-	H302
296	297	300	302	9	0.00	1.04600	1	;	O296-	C297-	C300-	H302

296	297	300	303	9	0.00	0.75312	1	;	O296-	C297-	C300-	O303
296	297	300	303	9	0.00	2.09200	3	;	O296-	C297-	C300-	O303
296	297	300	303	9	0.00	3.76560	2	;	O296-	C297-	C300-	O303
297	300	303	304	9	0.00	0.33472	2	;	C297-	C300-	O303-	C304
297	300	303	304	9	0.00	3.13800	3	;	C297-	C300-	O303-	C304
297	300	303	304	9	180.00	3.68192	1	;	C297-	C300-	O303-	C304
298	297	300	301	9	0.00	0.65084	3	;	H298-	C297-	C300-	H301
298	297	300	302	9	0.00	0.65084	3	;	H298-	C297-	C300-	H302
298	297	300	303	9	0.00	0.00000	0	;	H298-	C297-	C300-	O303
298	297	300	303	9	0.00	1.04600	1	;	H298-	C297-	C300-	O303
299	297	300	301	9	0.00	0.65084	3	;	H299-	C297-	C300-	H301
299	297	300	302	9	0.00	0.65084	3	;	H299-	C297-	C300-	H302
299	297	300	303	9	0.00	0.00000	0	;	H299-	C297-	C300-	O303
299	297	300	303	9	0.00	1.04600	1	;	H299-	C297-	C300-	O303
300	303	304	305	9	0.00	1.41001	3	;	C300-	O303-	C304-	H305
300	303	304	306	9	0.00	1.41001	3	;	C300-	O303-	C304-	H306
300	303	304	307	9	0.00	0.33472	2	;	C300-	O303-	C304-	C307
300	303	304	307	9	0.00	3.13800	3	;	C300-	O303-	C304-	C307
300	303	304	307	9	180.00	3.68192	1	;	C300-	O303-	C304-	C307
301	300	303	304	9	0.00	1.41001	3	;	H301-	C300-	O303-	C304
302	300	303	304	9	0.00	1.41001	3	;	H302-	C300-	O303-	C304
303	304	307	308	9	0.00	0.00000	0	;	O303-	C304-	C307-	H308
303	304	307	308	9	0.00	1.04600	1	;	O303-	C304-	C307-	H308
303	304	307	309	9	0.00	0.00000	0	;	O303-	C304-	C307-	H309
303	304	307	309	9	0.00	1.04600	1	;	O303-	C304-	C307-	H309
303	304	307	310	9	0.00	0.75312	1	;	O303-	C304-	C307-	O310
303	304	307	310	9	0.00	2.09200	3	;	O303-	C304-	C307-	O310
303	304	307	310	9	0.00	3.76560	2	;	O303-	C304-	C307-	O310
304	307	310	311	9	0.00	0.33472	2	;	C304-	C307-	O310-	C311

304	307	310	311	9	0.00	3.13800	3 ;	C304- C307- O310- C311
304	307	310	311	9	180.00	3.68192	1 ;	C304- C307- O310- C311
305	304	307	308	9	0.00	0.65084	3 ;	H305- C304- C307- H308
305	304	307	309	9	0.00	0.65084	3 ;	H305- C304- C307- H309
305	304	307	310	9	0.00	0.00000	0 ;	H305- C304- C307- O310
305	304	307	310	9	0.00	1.04600	1 ;	H305- C304- C307- O310
306	304	307	308	9	0.00	0.65084	3 ;	H306- C304- C307- H308
306	304	307	309	9	0.00	0.65084	3 ;	H306- C304- C307- H309
306	304	307	310	9	0.00	0.00000	0 ;	H306- C304- C307- O310
306	304	307	310	9	0.00	1.04600	1 ;	H306- C304- C307- O310
307	310	311	312	9	0.00	1.41001	3 ;	C307- O310- C311- H312
307	310	311	313	9	0.00	1.41001	3 ;	C307- O310- C311- H313
307	310	311	314	9	0.00	0.33472	2 ;	C307- O310- C311- C314
307	310	311	314	9	0.00	3.13800	3 ;	C307- O310- C311- C314
307	310	311	314	9	180.00	3.68192	1 ;	C307- O310- C311- C314
308	307	310	311	9	0.00	1.41001	3 ;	H308- C307- O310- C311
309	307	310	311	9	0.00	1.41001	3 ;	H309- C307- O310- C311
310	311	314	315	9	0.00	0.00000	0 ;	O310- C311- C314- H315
310	311	314	315	9	0.00	1.04600	1 ;	O310- C311- C314- H315
310	311	314	316	9	0.00	0.00000	0 ;	O310- C311- C314- H316
310	311	314	316	9	0.00	1.04600	1 ;	O310- C311- C314- H316
310	311	314	317	9	0.00	0.65084	3 ;	O310- C311- C314- O317
311	314	317	318	9	0.00	0.25104	3 ;	C311- C314- O317- H318
312	311	314	315	9	0.00	0.65084	3 ;	H312- C311- C314- H315
312	311	314	316	9	0.00	0.65084	3 ;	H312- C311- C314- H316
312	311	314	317	9	0.00	0.00000	0 ;	H312- C311- C314- O317
312	311	314	317	9	0.00	1.04600	1 ;	H312- C311- C314- O317
313	311	314	315	9	0.00	0.65084	3 ;	H313- C311- C314- H315
313	311	314	316	9	0.00	0.65084	3 ;	H313- C311- C314- H316

313	311	314	317	9	0.00	0.00000	0	;	H313-	C311-	C314-	O317
313	311	314	317	9	0.00	1.04600	1	;	H313-	C311-	C314-	O317
315	314	317	318	9	0.00	0.51463	3	;	H315-	C314-	O317-	H318
316	314	317	318	9	0.00	0.51463	3	;	H316-	C314-	O317-	H318

Include topology file "posre_PEG.itp"

[position_restraints]

; atom	type	fx	fy	fz
1	1	1000	1000	1000
3	1	1000	1000	1000
6	1	1000	1000	1000
9	1	1000	1000	1000
10	1	1000	1000	1000
13	1	1000	1000	1000
16	1	1000	1000	1000
17	1	1000	1000	1000
20	1	1000	1000	1000
23	1	1000	1000	1000
24	1	1000	1000	1000
27	1	1000	1000	1000
30	1	1000	1000	1000
31	1	1000	1000	1000
34	1	1000	1000	1000
37	1	1000	1000	1000
38	1	1000	1000	1000
41	1	1000	1000	1000
44	1	1000	1000	1000
45	1	1000	1000	1000
48	1	1000	1000	1000
51	1	1000	1000	1000

52	1	1000	1000	1000
55	1	1000	1000	1000
58	1	1000	1000	1000
59	1	1000	1000	1000
62	1	1000	1000	1000
65	1	1000	1000	1000
66	1	1000	1000	1000
69	1	1000	1000	1000
72	1	1000	1000	1000
73	1	1000	1000	1000
76	1	1000	1000	1000
79	1	1000	1000	1000
80	1	1000	1000	1000
83	1	1000	1000	1000
86	1	1000	1000	1000
87	1	1000	1000	1000
90	1	1000	1000	1000
93	1	1000	1000	1000
94	1	1000	1000	1000
97	1	1000	1000	1000
100	1	1000	1000	1000
101	1	1000	1000	1000
104	1	1000	1000	1000
107	1	1000	1000	1000
108	1	1000	1000	1000
111	1	1000	1000	1000
114	1	1000	1000	1000
115	1	1000	1000	1000
118	1	1000	1000	1000

121	1	1000	1000	1000
122	1	1000	1000	1000
125	1	1000	1000	1000
128	1	1000	1000	1000
129	1	1000	1000	1000
132	1	1000	1000	1000
135	1	1000	1000	1000
136	1	1000	1000	1000
139	1	1000	1000	1000
142	1	1000	1000	1000
143	1	1000	1000	1000
146	1	1000	1000	1000
149	1	1000	1000	1000
150	1	1000	1000	1000
153	1	1000	1000	1000
156	1	1000	1000	1000
157	1	1000	1000	1000
160	1	1000	1000	1000
163	1	1000	1000	1000
164	1	1000	1000	1000
167	1	1000	1000	1000
170	1	1000	1000	1000
171	1	1000	1000	1000
174	1	1000	1000	1000
177	1	1000	1000	1000
178	1	1000	1000	1000
181	1	1000	1000	1000
184	1	1000	1000	1000
185	1	1000	1000	1000

188	1	1000	1000	1000
191	1	1000	1000	1000
192	1	1000	1000	1000
195	1	1000	1000	1000
198	1	1000	1000	1000
199	1	1000	1000	1000
202	1	1000	1000	1000
205	1	1000	1000	1000
206	1	1000	1000	1000
209	1	1000	1000	1000
212	1	1000	1000	1000
213	1	1000	1000	1000
216	1	1000	1000	1000
219	1	1000	1000	1000
220	1	1000	1000	1000
223	1	1000	1000	1000
226	1	1000	1000	1000
227	1	1000	1000	1000
230	1	1000	1000	1000
233	1	1000	1000	1000
234	1	1000	1000	1000
237	1	1000	1000	1000
240	1	1000	1000	1000
241	1	1000	1000	1000
244	1	1000	1000	1000
247	1	1000	1000	1000
248	1	1000	1000	1000
251	1	1000	1000	1000
254	1	1000	1000	1000

255	1	1000	1000	1000
258	1	1000	1000	1000
261	1	1000	1000	1000
262	1	1000	1000	1000
265	1	1000	1000	1000
268	1	1000	1000	1000
269	1	1000	1000	1000
272	1	1000	1000	1000
275	1	1000	1000	1000
276	1	1000	1000	1000
279	1	1000	1000	1000
282	1	1000	1000	1000
283	1	1000	1000	1000
286	1	1000	1000	1000
289	1	1000	1000	1000
290	1	1000	1000	1000
293	1	1000	1000	1000
296	1	1000	1000	1000
297	1	1000	1000	1000
300	1	1000	1000	1000
303	1	1000	1000	1000
304	1	1000	1000	1000
307	1	1000	1000	1000
310	1	1000	1000	1000
311	1	1000	1000	1000
314	1	1000	1000	1000
317	1	1000	1000	1000

Include topology file "tip3p.itp"

[moleculetype]

```
; molname       nrexcl
SOL             2

[ atoms ]
; id  at type     res nr  res name  at name  cg nr  charge    mass
  1   OW          1       SOL       OW       1      -0.834    16.00000
  2   HW          1       SOL       HW1      1       0.417     1.00800
  3   HW          1       SOL       HW2      1       0.417     1.00800

#ifndef FLEXIBLE

[ settles ]
; OW    funct    doh      dhh
1       1        0.09572 0.15139

[ exclusions ]
1       2       3
2       1       3
3       1       2

#else

[ bonds ]
; i    j      funct   length  force_constant
1      2      1       0.09572 502416.0   0.09572       502416.0
1      3      1       0.09572 502416.0   0.09572       502416.0



[ angles ]
```

; i   j   k   funct   angle   force_constant

2   1   3   1   104.52 628.02   104.52 628.02

#endif

1.1.2 Structure coordinate files

Structure/coordinate file “PEG.gro”

```
PEG structure
 318
    1  PEG   O1    1  -7.928   0.028   0.000
    1  PEG   H2    2  -8.006  -0.031  -0.000
    1  PEG   C3    3  -7.807  -0.050  -0.000
    1  PEG   H4    4  -7.804  -0.114   0.090
    1  PEG   H5    5  -7.804  -0.113  -0.091
    1  PEG   C6    6  -7.696   0.055   0.000
    1  PEG   H7    7  -7.699   0.118   0.091
    1  PEG   H8    8  -7.699   0.119  -0.090
    1  PEG   O9    9  -7.575  -0.025   0.000
    1  PEG  C10   10  -7.455   0.055   0.000
    1  PEG  H11   11  -7.451   0.118  -0.090
    1  PEG  H12   12  -7.451   0.118   0.091
    1  PEG  C13   13  -7.344  -0.052  -0.000
    1  PEG  H14   14  -7.348  -0.115  -0.091
    1  PEG  H15   15  -7.348  -0.115   0.090
    1  PEG  O16   16  -7.223   0.028   0.000
    1  PEG  C17   17  -7.103  -0.052  -0.000
    1  PEG  H18   18  -7.099  -0.116   0.090
    1  PEG  H19   19  -7.099  -0.115  -0.091
    1  PEG  C20   20  -6.992   0.054   0.000
    1  PEG  H21   21  -6.995   0.117   0.091
```

1	PEG	H22	22	-6.995	0.117	-0.090
1	PEG	O23	23	-6.871	-0.026	0.000
1	PEG	C24	24	-6.750	0.053	0.000
1	PEG	H25	25	-6.747	0.117	-0.090
1	PEG	H26	26	-6.747	0.116	0.091
1	PEG	C27	27	-6.639	-0.053	-0.000
1	PEG	H28	28	-6.643	-0.116	-0.091
1	PEG	H29	29	-6.643	-0.116	0.090
1	PEG	O30	30	-6.519	0.027	0.000
1	PEG	C31	31	-6.398	-0.053	-0.000
1	PEG	H32	32	-6.395	-0.116	0.090
1	PEG	H33	33	-6.395	-0.116	-0.091
1	PEG	C34	34	-6.287	0.053	0.000
1	PEG	H35	35	-6.290	0.116	0.091
1	PEG	H36	36	-6.290	0.117	-0.090
1	PEG	O37	37	-6.166	-0.027	-0.000
1	PEG	C38	38	-6.046	0.053	0.000
1	PEG	H39	39	-6.042	0.116	-0.090
1	PEG	H40	40	-6.042	0.116	0.091
1	PEG	C41	41	-5.935	-0.053	-0.000
1	PEG	H42	42	-5.938	-0.116	-0.091
1	PEG	H43	43	-5.938	-0.117	0.090
1	PEG	O44	44	-5.814	0.027	0.000
1	PEG	C45	45	-5.693	-0.053	-0.000
1	PEG	H46	46	-5.690	-0.117	0.090
1	PEG	H47	47	-5.690	-0.116	-0.091
1	PEG	C48	48	-5.583	0.053	0.000
1	PEG	H49	49	-5.586	0.116	0.091
1	PEG	H50	50	-5.586	0.116	-0.090

1	PEG	O51	51	-5.462	-0.027	0.000
1	PEG	C52	52	-5.341	0.053	0.000
1	PEG	H53	53	-5.338	0.116	-0.090
1	PEG	H54	54	-5.338	0.116	0.091
1	PEG	C55	55	-5.230	-0.053	0.000
1	PEG	H56	56	-5.234	-0.116	-0.090
1	PEG	H57	57	-5.234	-0.117	0.090
1	PEG	O58	58	-5.109	0.027	0.000
1	PEG	C59	59	-4.989	-0.053	0.000
1	PEG	H60	60	-4.985	-0.117	0.090
1	PEG	H61	61	-4.985	-0.116	-0.090
1	PEG	C62	62	-4.878	0.053	0.000
1	PEG	H63	63	-4.881	0.116	0.091
1	PEG	H64	64	-4.881	0.116	-0.090
1	PEG	O65	65	-4.757	-0.027	0.000
1	PEG	C66	66	-4.636	0.053	0.000
1	PEG	H67	67	-4.633	0.116	-0.090
1	PEG	H68	68	-4.633	0.116	0.091
1	PEG	C69	69	-4.526	-0.053	0.000
1	PEG	H70	70	-4.529	-0.117	-0.090
1	PEG	H71	71	-4.529	-0.117	0.091
1	PEG	O72	72	-4.405	0.027	0.000
1	PEG	C73	73	-4.284	-0.053	0.000
1	PEG	H74	74	-4.281	-0.117	0.091
1	PEG	H75	75	-4.281	-0.117	-0.090
1	PEG	C76	76	-4.173	0.052	0.000
1	PEG	H77	77	-4.176	0.116	0.091
1	PEG	H78	78	-4.176	0.116	-0.090
1	PEG	O79	79	-4.052	-0.028	0.000

1	PEG	C80	80	-3.932	0.052	-0.000
1	PEG	H81	81	-3.928	0.116	-0.090
1	PEG	H82	82	-3.928	0.116	0.090
1	PEG	C83	83	-3.821	-0.054	-0.000
1	PEG	H84	84	-3.824	-0.117	-0.090
1	PEG	H85	85	-3.824	-0.117	0.090
1	PEG	O86	86	-3.700	0.026	-0.001
1	PEG	C87	87	-3.579	-0.054	-0.001
1	PEG	H88	88	-3.576	-0.117	0.090
1	PEG	H89	89	-3.576	-0.117	-0.091
1	PEG	C90	90	-3.468	0.052	-0.001
1	PEG	H91	91	-3.472	0.116	0.089
1	PEG	H92	92	-3.472	0.115	-0.092
1	PEG	O93	93	-3.348	-0.028	-0.001
1	PEG	C94	94	-3.227	0.052	-0.002
1	PEG	H95	95	-3.224	0.115	-0.092
1	PEG	H96	96	-3.224	0.116	0.089
1	PEG	C97	97	-3.116	-0.054	-0.001
1	PEG	H98	98	-3.120	-0.118	-0.091
1	PEG	H99	99	-3.119	-0.117	0.089
1	PEG	O100	100	-2.995	0.026	-0.002
1	PEG	C101	101	-2.875	-0.054	-0.002
1	PEG	H102	102	-2.871	-0.116	0.089
1	PEG	H103	103	-2.871	-0.118	-0.092
1	PEG	C104	104	-2.764	0.052	-0.002
1	PEG	H105	105	-2.768	0.117	0.087
1	PEG	H106	106	-2.767	0.115	-0.093
1	PEG	O107	107	-2.643	-0.027	-0.001
1	PEG	C108	108	-2.522	0.053	-0.002

1	PEG	H109	109	-2.519	0.115	-0.093
1	PEG	H110	110	-2.519	0.117	0.088
1	PEG	C111	111	-2.411	-0.053	-0.000
1	PEG	H112	112	-2.414	-0.117	-0.090
1	PEG	H113	113	-2.415	-0.115	0.091
1	PEG	O114	114	-2.291	0.027	-0.001
1	PEG	C115	115	-2.170	-0.052	0.001
1	PEG	H116	116	-2.167	-0.115	0.092
1	PEG	H117	117	-2.166	-0.117	-0.089
1	PEG	C118	118	-2.059	0.054	0.000
1	PEG	H119	119	-2.063	0.118	0.090
1	PEG	H120	120	-2.062	0.116	-0.091
1	PEG	O121	121	-1.938	-0.026	0.002
1	PEG	C122	122	-1.817	0.053	0.001
1	PEG	H123	123	-1.814	0.116	-0.090
1	PEG	H124	124	-1.814	0.118	0.091
1	PEG	C125	125	-1.707	-0.052	0.003
1	PEG	H126	126	-1.710	-0.117	-0.087
1	PEG	H127	127	-1.710	-0.115	0.094
1	PEG	O128	128	-1.586	0.027	0.002
1	PEG	C129	129	-1.465	-0.053	0.003
1	PEG	H130	130	-1.462	-0.115	0.094
1	PEG	H131	131	-1.462	-0.117	-0.087
1	PEG	C132	132	-1.354	0.053	0.002
1	PEG	H133	133	-1.357	0.117	0.091
1	PEG	H134	134	-1.358	0.115	-0.089
1	PEG	O135	135	-1.233	-0.027	0.002
1	PEG	C136	136	-1.113	0.053	0.000
1	PEG	H137	137	-1.110	0.115	-0.091

1	PEG	H138	138	-1.109	0.117	0.090
1	PEG	C139	139	-1.002	-0.053	0.000
1	PEG	H140	140	-1.006	-0.117	-0.089
1	PEG	H141	141	-1.004	-0.116	0.091
1	PEG	O142	142	-0.881	0.027	-0.001
1	PEG	C143	143	-0.760	-0.053	-0.001
1	PEG	H144	144	-0.756	-0.116	0.090
1	PEG	H145	145	-0.757	-0.117	-0.091
1	PEG	C146	146	-0.649	0.053	-0.002
1	PEG	H147	147	-0.653	0.117	0.088
1	PEG	H148	148	-0.653	0.115	-0.093
1	PEG	O149	149	-0.529	-0.027	-0.002
1	PEG	C150	150	-0.408	0.053	-0.003
1	PEG	H151	151	-0.405	0.115	-0.094
1	PEG	H152	152	-0.404	0.117	0.087
1	PEG	C153	153	-0.297	-0.053	-0.002
1	PEG	H154	154	-0.300	-0.117	-0.092
1	PEG	H155	155	-0.300	-0.116	0.089
1	PEG	O156	156	-0.176	0.027	-0.003
1	PEG	C157	157	-0.055	-0.053	-0.002
1	PEG	H158	158	-0.053	-0.116	0.089
1	PEG	H159	159	-0.052	-0.117	-0.091
1	PEG	C160	160	0.055	0.053	-0.002
1	PEG	H161	161	0.052	0.117	0.088
1	PEG	H162	162	0.053	0.115	-0.093
1	PEG	O163	163	0.176	-0.027	-0.000
1	PEG	C164	164	0.297	0.053	-0.001
1	PEG	H165	165	0.301	0.115	-0.091
1	PEG	H166	166	0.300	0.117	0.089

1	PEG	C167	167	0.408	-0.053	0.001
1	PEG	H168	168	0.405	-0.117	-0.089
1	PEG	H169	169	0.404	-0.115	0.092
1	PEG	O170	170	0.529	0.027	0.001
1	PEG	C171	171	0.650	-0.053	0.002
1	PEG	H172	172	0.652	-0.115	0.093
1	PEG	H173	173	0.653	-0.117	-0.088
1	PEG	C174	174	0.760	0.053	0.002
1	PEG	H175	175	0.757	0.117	0.092
1	PEG	H176	176	0.757	0.116	-0.089
1	PEG	O177	177	0.881	-0.026	0.003
1	PEG	C178	178	1.002	0.054	0.002
1	PEG	H179	179	1.005	0.117	-0.089
1	PEG	H180	180	1.005	0.117	0.092
1	PEG	C181	181	1.113	-0.052	0.003
1	PEG	H182	182	1.109	-0.116	-0.088
1	PEG	H183	183	1.109	-0.115	0.093
1	PEG	O184	184	1.233	0.028	0.002
1	PEG	C185	185	1.354	-0.052	0.002
1	PEG	H186	186	1.357	-0.115	0.093
1	PEG	H187	187	1.357	-0.116	-0.088
1	PEG	C188	188	1.465	0.054	0.002
1	PEG	H189	189	1.462	0.117	0.092
1	PEG	H190	190	1.462	0.117	-0.089
1	PEG	O191	191	1.586	-0.027	0.001
1	PEG	C192	192	1.707	0.053	0.001
1	PEG	H193	193	1.710	0.116	-0.090
1	PEG	H194	194	1.710	0.117	0.091
1	PEG	C195	195	1.817	-0.053	0.001

1	PEG	H196	196	1.814	-0.116	-0.090
1	PEG	H197	197	1.814	-0.116	0.091
1	PEG	O198	198	1.938	0.027	0.000
1	PEG	C199	199	2.059	-0.053	-0.000
1	PEG	H200	200	2.062	-0.116	0.090
1	PEG	H201	201	2.062	-0.116	-0.090
1	PEG	C202	202	2.170	0.053	-0.000
1	PEG	H203	203	2.167	0.116	0.090
1	PEG	H204	204	2.166	0.116	-0.091
1	PEG	O205	205	2.291	-0.027	-0.000
1	PEG	C206	206	2.411	0.053	-0.001
1	PEG	H207	207	2.415	0.116	-0.091
1	PEG	H208	208	2.415	0.116	0.090
1	PEG	C209	209	2.522	-0.053	-0.001
1	PEG	H210	210	2.519	-0.116	-0.091
1	PEG	H211	211	2.519	-0.117	0.090
1	PEG	O212	212	2.643	0.027	-0.001
1	PEG	C213	213	2.764	-0.053	-0.001
1	PEG	H214	214	2.767	-0.116	0.090
1	PEG	H215	215	2.767	-0.116	-0.091
1	PEG	C216	216	2.875	0.053	-0.000
1	PEG	H217	217	2.871	0.116	0.090
1	PEG	H218	218	2.871	0.116	-0.091
1	PEG	O219	219	2.995	-0.027	-0.001
1	PEG	C220	220	3.116	0.053	-0.000
1	PEG	H221	221	3.119	0.117	-0.091
1	PEG	H222	222	3.119	0.116	0.090
1	PEG	C223	223	3.227	-0.053	-0.000
1	PEG	H224	224	3.224	-0.116	-0.091

1	PEG	H225	225	3.224	-0.116	0.090
1	PEG	O226	226	3.348	0.027	-0.000
1	PEG	C227	227	3.469	-0.053	-0.000
1	PEG	H228	228	3.472	-0.116	0.090
1	PEG	H229	229	3.472	-0.116	-0.091
1	PEG	C230	230	3.579	0.053	-0.000
1	PEG	H231	231	3.576	0.116	0.091
1	PEG	H232	232	3.576	0.117	-0.090
1	PEG	O233	233	3.700	-0.027	-0.000
1	PEG	C234	234	3.821	0.053	0.000
1	PEG	H235	235	3.824	0.117	-0.090
1	PEG	H236	236	3.824	0.116	0.091
1	PEG	C237	237	3.932	-0.053	-0.000
1	PEG	H238	238	3.928	-0.116	-0.091
1	PEG	H239	239	3.928	-0.116	0.090
1	PEG	O240	240	4.052	0.027	0.000
1	PEG	C241	241	4.173	-0.053	-0.000
1	PEG	H242	242	4.176	-0.116	0.090
1	PEG	H243	243	4.176	-0.116	-0.091
1	PEG	C244	244	4.284	0.053	0.000
1	PEG	H245	245	4.281	0.116	0.091
1	PEG	H246	246	4.281	0.117	-0.090
1	PEG	O247	247	4.405	-0.027	-0.000
1	PEG	C248	248	4.526	0.053	0.000
1	PEG	H249	249	4.529	0.117	-0.090
1	PEG	H250	250	4.529	0.116	0.091
1	PEG	C251	251	4.636	-0.053	-0.000
1	PEG	H252	252	4.633	-0.116	-0.091
1	PEG	H253	253	4.633	-0.116	0.090

1	PEG	O254	254	4.757	0.027	0.000
1	PEG	C255	255	4.878	-0.052	-0.000
1	PEG	H256	256	4.881	-0.116	0.090
1	PEG	H257	257	4.881	-0.116	-0.091
1	PEG	C258	258	4.989	0.053	0.000
1	PEG	H259	259	4.985	0.117	0.091
1	PEG	H260	260	4.985	0.117	-0.090
1	PEG	O261	261	5.109	-0.027	-0.000
1	PEG	C262	262	5.230	0.053	0.000
1	PEG	H263	263	5.234	0.117	-0.090
1	PEG	H264	264	5.234	0.117	0.091
1	PEG	C265	265	5.341	-0.053	-0.000
1	PEG	H266	266	5.338	-0.116	-0.091
1	PEG	H267	267	5.338	-0.116	0.090
1	PEG	O268	268	5.462	0.027	-0.000
1	PEG	C269	269	5.583	-0.053	-0.000
1	PEG	H270	270	5.586	-0.116	0.090
1	PEG	H271	271	5.586	-0.116	-0.091
1	PEG	C272	272	5.693	0.053	0.000
1	PEG	H273	273	5.690	0.116	0.091
1	PEG	H274	274	5.690	0.117	-0.090
1	PEG	O275	275	5.814	-0.027	-0.000
1	PEG	C276	276	5.935	0.053	0.000
1	PEG	H277	277	5.938	0.117	-0.090
1	PEG	H278	278	5.938	0.116	0.091
1	PEG	C279	279	6.046	-0.053	-0.000
1	PEG	H280	280	6.042	-0.116	-0.091
1	PEG	H281	281	6.042	-0.116	0.090
1	PEG	O282	282	6.166	0.027	-0.000

1	PEG	C283	283	6.287	-0.053	-0.000
1	PEG	H284	284	6.290	-0.117	0.090
1	PEG	H285	285	6.290	-0.116	-0.091
1	PEG	C286	286	6.398	0.053	0.000
1	PEG	H287	287	6.395	0.116	0.091
1	PEG	H288	288	6.395	0.117	-0.090
1	PEG	O289	289	6.519	-0.027	-0.000
1	PEG	C290	290	6.639	0.053	0.000
1	PEG	H291	291	6.643	0.116	-0.090
1	PEG	H292	292	6.643	0.116	0.091
1	PEG	C293	293	6.750	-0.053	-0.000
1	PEG	H294	294	6.747	-0.116	-0.091
1	PEG	H295	295	6.747	-0.117	0.090
1	PEG	O296	296	6.871	0.026	-0.000
1	PEG	C297	297	6.992	-0.054	-0.000
1	PEG	H298	298	6.995	-0.117	0.090
1	PEG	H299	299	6.995	-0.117	-0.091
1	PEG	C300	300	7.103	0.052	0.000
1	PEG	H301	301	7.099	0.115	0.091
1	PEG	H302	302	7.099	0.116	-0.090
1	PEG	O303	303	7.223	-0.028	-0.000
1	PEG	C304	304	7.344	0.052	0.000
1	PEG	H305	305	7.348	0.115	-0.090
1	PEG	H306	306	7.348	0.115	0.091
1	PEG	C307	307	7.455	-0.055	-0.000
1	PEG	H308	308	7.451	-0.118	-0.091
1	PEG	H309	309	7.451	-0.118	0.090
1	PEG	O310	310	7.575	0.025	-0.000
1	PEG	C311	311	7.696	-0.055	-0.000

```
    1  PEG H312  312   7.699  -0.119   0.090
    1  PEG H313  313   7.699  -0.118  -0.091
    1  PEG C314  314   7.807   0.050   0.000
    1  PEG H315  315   7.804   0.113   0.091
    1  PEG H316  316   7.804   0.114  -0.090
    1  PEG O317  317   7.928  -0.028   0.000
    1  PEG H318  318   8.006   0.031   0.000
  320.24000     4.74800     3.75800
```

Structure/coordinate file "water.gro"

```
216H2O,WATJP01,SPC216,SPC-MODEL,300K,BOX(M)=1.86206NM,WFVG,MAR. 1984
  648
    1SOL     OW    1    .230    .628    .113
    1SOL    HW1    2    .137    .626    .150
    1SOL    HW2    3    .231    .589    .021
    2SOL     OW    4    .225    .275   -.866
    2SOL    HW1    5    .260    .258   -.774
    2SOL    HW2    6    .137    .230   -.878
    3SOL     OW    7    .019    .368    .647
    3SOL    HW1    8   -.063    .411    .686
    3SOL    HW2    9   -.009    .295    .584
    4SOL     OW   10    .569   -.587   -.697
    4SOL    HW1   11    .476   -.594   -.734
    4SOL    HW2   12    .580   -.498   -.653
    5SOL     OW   13   -.307   -.351    .703
    5SOL    HW1   14   -.364   -.367    .784
    5SOL    HW2   15   -.366   -.341    .623
    6SOL     OW   16   -.119    .618    .856
    6SOL    HW1   17   -.086    .712    .856
    6SOL    HW2   18   -.068    .564    .922
```

7SOL	OW	19	-.727	.703	.717
7SOL	HW1	20	-.670	.781	.692
7SOL	HW2	21	-.787	.729	.793
8SOL	OW	22	-.107	.607	.231
8SOL	HW1	23	-.119	.594	.132
8SOL	HW2	24	-.137	.526	.280
9SOL	OW	25	.768	-.718	-.839
9SOL	HW1	26	.690	-.701	-.779
9SOL	HW2	27	.802	-.631	-.875
10SOL	OW	28	.850	.798	-.039
10SOL	HW1	29	.846	.874	.026
10SOL	HW2	30	.872	.834	-.130
11SOL	OW	31	.685	-.850	.665
11SOL	HW1	32	.754	-.866	.735
11SOL	HW2	33	.612	-.793	.703
12SOL	OW	34	.686	-.701	-.059
12SOL	HW1	35	.746	-.622	-.045
12SOL	HW2	36	.600	-.670	-.100
13SOL	OW	37	.335	-.427	-.801
13SOL	HW1	38	.257	-.458	-.854
13SOL	HW2	39	.393	-.369	-.858
14SOL	OW	40	-.402	-.357	-.523
14SOL	HW1	41	-.378	-.263	-.497
14SOL	HW2	42	-.418	-.411	-.441
15SOL	OW	43	.438	.392	-.363
15SOL	HW1	44	.520	.336	-.354
15SOL	HW2	45	.357	.334	-.359
16SOL	OW	46	-.259	.447	.737
16SOL	HW1	47	-.333	.493	.687

16SOL	HW2	48	-.208	.515	.790
17SOL	OW	49	.231	-.149	.483
17SOL	HW1	50	.265	-.072	.537
17SOL	HW2	51	.275	-.149	.393
18SOL	OW	52	-.735	-.521	-.172
18SOL	HW1	53	-.688	-.521	-.084
18SOL	HW2	54	-.783	-.608	-.183
19SOL	OW	55	.230	-.428	.538
19SOL	HW1	56	.204	-.332	.538
19SOL	HW2	57	.159	-.482	.583
20SOL	OW	58	.240	-.771	.886
20SOL	HW1	59	.254	-.855	.938
20SOL	HW2	60	.185	-.707	.941
21SOL	OW	61	.620	-.076	-.423
21SOL	HW1	62	.528	-.093	-.388
21SOL	HW2	63	.648	.016	-.397
22SOL	OW	64	.606	-.898	.123
22SOL	HW1	65	.613	-.814	.069
22SOL	HW2	66	.652	-.885	.211
23SOL	OW	67	-.268	.114	-.382
23SOL	HW1	68	-.286	.181	-.454
23SOL	HW2	69	-.271	.160	-.293
24SOL	OW	70	.122	.643	.563
24SOL	HW1	71	.077	.555	.580
24SOL	HW2	72	.121	.697	.647
25SOL	OW	73	-.020	-.095	.359
25SOL	HW1	74	.034	-.124	.439
25SOL	HW2	75	.010	-.005	.330
26SOL	OW	76	.027	-.266	.117

26SOL	HW1	77	.008	-.362	.138
26SOL	HW2	78	-.006	-.208	.192
27SOL	OW	79	-.173	.922	.612
27SOL	HW1	80	-.078	.893	.620
27SOL	HW2	81	-.181	.987	.537
28SOL	OW	82	-.221	-.754	.432
28SOL	HW1	83	-.135	-.752	.380
28SOL	HW2	84	-.207	-.707	.520
29SOL	OW	85	.113	.737	-.265
29SOL	HW1	86	.201	.724	-.220
29SOL	HW2	87	.100	.834	-.287
30SOL	OW	88	.613	-.497	.726
30SOL	HW1	89	.564	-.584	.735
30SOL	HW2	90	.590	-.454	.639
31SOL	OW	91	-.569	-.634	-.439
31SOL	HW1	92	-.532	-.707	-.497
31SOL	HW2	93	-.517	-.629	-.354
32SOL	OW	94	.809	.004	.502
32SOL	HW1	95	.849	.095	.493
32SOL	HW2	96	.709	.012	.508
33SOL	OW	97	.197	-.886	-.598
33SOL	HW1	98	.286	-.931	-.612
33SOL	HW2	99	.124	-.951	-.617
34SOL	OW	100	-.337	-.863	.190
34SOL	HW1	101	-.400	-.939	.203
34SOL	HW2	102	-.289	-.845	.276
35SOL	OW	103	-.675	-.070	-.246
35SOL	HW1	104	-.651	-.010	-.322
35SOL	HW2	105	-.668	-.165	-.276

36SOL	OW	106	.317	.251	-.061
36SOL	HW1	107	.388	.322	-.055
36SOL	HW2	108	.229	.290	-.033
37SOL	OW	109	-.396	-.445	-.909
37SOL	HW1	110	-.455	-.439	-.829
37SOL	HW2	111	-.411	-.533	-.955
38SOL	OW	112	-.195	-.148	.572
38SOL	HW1	113	-.236	-.171	.484
38SOL	HW2	114	-.213	-.222	.637
39SOL	OW	115	.598	.729	.270
39SOL	HW1	116	.622	.798	.202
39SOL	HW2	117	.520	.762	.324
40SOL	OW	118	-.581	.345	-.918
40SOL	HW1	119	-.667	.295	-.931
40SOL	HW2	120	-.519	.291	-.862
41SOL	OW	121	-.286	-.200	.307
41SOL	HW1	122	-.197	-.154	.310
41SOL	HW2	123	-.307	-.224	.212
42SOL	OW	124	.807	.605	-.397
42SOL	HW1	125	.760	.602	-.308
42SOL	HW2	126	.756	.550	-.463
43SOL	OW	127	-.468	.469	-.188
43SOL	HW1	128	-.488	.512	-.100
43SOL	HW2	129	-.390	.407	-.179
44SOL	OW	130	-.889	.890	-.290
44SOL	HW1	131	-.843	.806	-.319
44SOL	HW2	132	-.945	.924	-.365
45SOL	OW	133	-.871	.410	-.620
45SOL	HW1	134	-.948	.444	-.566

45SOL	HW2	135	-.905	.359	-.699
46SOL	OW	136	-.821	.701	.429
46SOL	HW1	137	-.795	.697	.525
46SOL	HW2	138	-.906	.650	.415
47SOL	OW	139	.076	.811	.789
47SOL	HW1	140	.175	.799	.798
47SOL	HW2	141	.052	.906	.810
48SOL	OW	142	.130	-.041	-.291
48SOL	HW1	143	.120	-.056	-.192
48SOL	HW2	144	.044	-.005	-.327
49SOL	OW	145	.865	.348	.195
49SOL	HW1	146	.924	.411	.146
49SOL	HW2	147	.884	.254	.166
50SOL	OW	148	-.143	.585	-.031
50SOL	HW1	149	-.169	.674	-.067
50SOL	HW2	150	-.145	.517	-.104
51SOL	OW	151	-.500	-.718	.545
51SOL	HW1	152	-.417	-.747	.497
51SOL	HW2	153	-.549	-.651	.489
52SOL	OW	154	.550	.196	.885
52SOL	HW1	155	.545	.191	.985
52SOL	HW2	156	.552	.292	.856
53SOL	OW	157	-.854	-.406	.477
53SOL	HW1	158	-.900	-.334	.425
53SOL	HW2	159	-.858	-.386	.575
54SOL	OW	160	.351	-.061	.853
54SOL	HW1	161	.401	-.147	.859
54SOL	HW2	162	.416	.016	.850
55SOL	OW	163	-.067	-.796	.873

55SOL	HW1	164	-.129	-.811	.797
55SOL	HW2	165	-.119	-.785	.958
56SOL	OW	166	-.635	-.312	-.356
56SOL	HW1	167	-.629	-.389	-.292
56SOL	HW2	168	-.687	-.338	-.436
57SOL	OW	169	.321	-.919	.242
57SOL	HW1	170	.403	-.880	.200
57SOL	HW2	171	.294	-1.001	.193
58SOL	OW	172	-.404	.735	.728
58SOL	HW1	173	-.409	.670	.803
58SOL	HW2	174	-.324	.794	.741
59SOL	OW	175	.461	-.596	-.135
59SOL	HW1	176	.411	-.595	-.221
59SOL	HW2	177	.398	-.614	-.059
60SOL	OW	178	-.751	-.086	.237
60SOL	HW1	179	-.811	-.148	.287
60SOL	HW2	180	-.720	-.130	.152
61SOL	OW	181	.202	.285	-.364
61SOL	HW1	182	.122	.345	-.377
61SOL	HW2	183	.192	.236	-.278
62SOL	OW	184	-.230	-.485	.081
62SOL	HW1	185	-.262	-.391	.071
62SOL	HW2	186	-.306	-.548	.069
63SOL	OW	187	.464	-.119	.323
63SOL	HW1	188	.497	-.080	.409
63SOL	HW2	189	.540	-.126	.258
64SOL	OW	190	-.462	.107	.426
64SOL	HW1	191	-.486	.070	.336
64SOL	HW2	192	-.363	.123	.430

65SOL	OW	193	.249	-.077	-.621
65SOL	HW1	194	.306	-.142	-.571
65SOL	HW2	195	.233	-.110	-.714
66SOL	OW	196	-.922	-.164	.904
66SOL	HW1	197	-.842	-.221	.925
66SOL	HW2	198	-.971	-.204	.827
67SOL	OW	199	.382	.700	.480
67SOL	HW1	200	.427	.610	.477
67SOL	HW2	201	.288	.689	.513
68SOL	OW	202	-.315	.222	-.133
68SOL	HW1	203	-.320	.259	-.041
68SOL	HW2	204	-.387	.153	-.145
69SOL	OW	205	.614	.122	.117
69SOL	HW1	206	.712	.100	.124
69SOL	HW2	207	.583	.105	.024
70SOL	OW	208	.781	.264	-.113
70SOL	HW1	209	.848	.203	-.070
70SOL	HW2	210	.708	.283	-.048
71SOL	OW	211	.888	-.348	-.667
71SOL	HW1	212	.865	-.373	-.761
71SOL	HW2	213	.949	-.417	-.628
72SOL	OW	214	-.511	.590	-.429
72SOL	HW1	215	-.483	.547	-.344
72SOL	HW2	216	-.486	.686	-.428
73SOL	OW	217	.803	-.460	.924
73SOL	HW1	218	.893	-.446	.882
73SOL	HW2	219	.732	-.458	.853
74SOL	OW	220	.922	.503	.899
74SOL	HW1	221	.897	.494	.803

74SOL	HW2	222	.970	.421	.930
75SOL	OW	223	.539	.064	.512
75SOL	HW1	224	.458	.065	.570
75SOL	HW2	225	.542	.147	.457
76SOL	OW	226	-.428	-.674	.041
76SOL	HW1	227	-.396	-.750	.098
76SOL	HW2	228	-.520	-.647	.071
77SOL	OW	229	.297	.035	.171
77SOL	HW1	230	.346	.119	.150
77SOL	HW2	231	.359	-.030	.216
78SOL	OW	232	-.927	.236	.480
78SOL	HW1	233	-.975	.277	.402
78SOL	HW2	234	-.828	.234	.461
79SOL	OW	235	-.786	.683	-.398
79SOL	HW1	236	-.866	.622	-.395
79SOL	HW2	237	-.705	.630	-.422
80SOL	OW	238	-.635	-.292	.793
80SOL	HW1	239	-.614	-.218	.728
80SOL	HW2	240	-.567	-.292	.866
81SOL	OW	241	.459	-.710	.741
81SOL	HW1	242	.388	-.737	.806
81SOL	HW2	243	.433	-.738	.648
82SOL	OW	244	-.591	-.065	.591
82SOL	HW1	245	-.547	-.001	.527
82SOL	HW2	246	-.641	-.013	.661
83SOL	OW	247	-.830	.549	.016
83SOL	HW1	248	-.871	.631	-.023
83SOL	HW2	249	-.766	.575	.089
84SOL	OW	250	.078	.556	-.476

84SOL	HW1	251	.170	.555	-.517
84SOL	HW2	252	.072	.630	-.409
85SOL	OW	253	.561	.222	-.715
85SOL	HW1	254	.599	.138	-.678
85SOL	HW2	255	.473	.241	-.671
86SOL	OW	256	.866	.454	.642
86SOL	HW1	257	.834	.526	.580
86SOL	HW2	258	.890	.373	.589
87SOL	OW	259	-.845	.039	.753
87SOL	HW1	260	-.917	.044	.684
87SOL	HW2	261	-.869	-.030	.822
88SOL	OW	262	-.433	-.689	.867
88SOL	HW1	263	-.488	-.773	.860
88SOL	HW2	264	-.407	-.660	.775
89SOL	OW	265	-.396	.590	-.870
89SOL	HW1	266	-.426	.495	-.863
89SOL	HW2	267	-.323	.606	-.804
90SOL	OW	268	-.005	.833	.377
90SOL	HW1	269	.037	.769	.441
90SOL	HW2	270	-.043	.782	.299
91SOL	OW	271	.488	-.477	.174
91SOL	HW1	272	.401	-.492	.221
91SOL	HW2	273	.471	-.451	.079
92SOL	OW	274	-.198	-.582	.657
92SOL	HW1	275	-.099	-.574	.671
92SOL	HW2	276	-.243	-.498	.688
93SOL	OW	277	-.472	.575	.078
93SOL	HW1	278	-.526	.554	.159
93SOL	HW2	279	-.381	.534	.087

94SOL	OW	280	.527	.256	.328
94SOL	HW1	281	.554	.197	.253
94SOL	HW2	282	.527	.351	.297
95SOL	OW	283	-.108	-.639	-.274
95SOL	HW1	284	-.017	-.678	-.287
95SOL	HW2	285	-.100	-.543	-.250
96SOL	OW	286	-.798	-.515	-.522
96SOL	HW1	287	-.878	-.538	-.467
96SOL	HW2	288	-.715	-.541	-.473
97SOL	OW	289	-.270	-.233	-.237
97SOL	HW1	290	-.243	-.199	-.327
97SOL	HW2	291	-.191	-.271	-.191
98SOL	OW	292	-.751	-.667	-.762
98SOL	HW1	293	-.791	-.623	-.681
98SOL	HW2	294	-.792	-.630	-.845
99SOL	OW	295	-.224	-.763	-.783
99SOL	HW1	296	-.219	-.682	-.724
99SOL	HW2	297	-.310	-.761	-.834
100SOL	OW	298	.915	.089	-.460
100SOL	HW1	299	.940	.069	-.555
100SOL	HW2	300	.987	.145	-.418
101SOL	OW	301	-.882	-.746	-.143
101SOL	HW1	302	-.981	-.740	-.133
101SOL	HW2	303	-.859	-.826	-.199
102SOL	OW	304	.705	-.812	.368
102SOL	HW1	305	.691	-.805	.467
102SOL	HW2	306	.789	-.863	.350
103SOL	OW	307	.410	.813	-.611
103SOL	HW1	308	.496	.825	-.561

103SOL	HW2	309	.368	.726	-.584
104SOL	OW	310	-.588	.386	-.600
104SOL	HW1	311	-.567	.460	-.536
104SOL	HW2	312	-.677	.403	-.643
105SOL	OW	313	.064	-.298	-.531
105SOL	HW1	314	.018	-.216	-.565
105SOL	HW2	315	.162	-.279	-.522
106SOL	OW	316	.367	-.762	.501
106SOL	HW1	317	.360	-.679	.445
106SOL	HW2	318	.371	-.842	.441
107SOL	OW	319	.566	.537	.865
107SOL	HW1	320	.578	.603	.791
107SOL	HW2	321	.612	.571	.948
108SOL	OW	322	-.610	-.514	.388
108SOL	HW1	323	-.560	-.437	.428
108SOL	HW2	324	-.705	-.512	.420
109SOL	OW	325	-.590	-.417	-.720
109SOL	HW1	326	-.543	-.404	-.633
109SOL	HW2	327	-.656	-.491	-.711
110SOL	OW	328	-.280	.639	.472
110SOL	HW1	329	-.311	.700	.545
110SOL	HW2	330	-.230	.691	.403
111SOL	OW	331	.354	-.352	-.533
111SOL	HW1	332	.333	-.396	-.620
111SOL	HW2	333	.451	-.326	-.530
112SOL	OW	334	.402	.751	-.264
112SOL	HW1	335	.470	.806	-.311
112SOL	HW2	336	.442	.663	-.237
113SOL	OW	337	-.275	.779	-.192

113SOL	HW1	338	-.367	.817	-.197
113SOL	HW2	339	-.215	.826	-.257
114SOL	OW	340	-.849	.105	-.092
114SOL	HW1	341	-.843	.190	-.144
114SOL	HW2	342	-.817	.029	-.149
115SOL	OW	343	.504	.050	-.122
115SOL	HW1	344	.462	-.007	-.192
115SOL	HW2	345	.438	.119	-.090
116SOL	OW	346	.573	.870	-.833
116SOL	HW1	347	.617	.959	-.842
116SOL	HW2	348	.510	.870	-.756
117SOL	OW	349	-.502	.862	-.817
117SOL	HW1	350	-.577	.862	-.883
117SOL	HW2	351	-.465	.770	-.808
118SOL	OW	352	-.653	.525	.275
118SOL	HW1	353	-.640	.441	.329
118SOL	HW2	354	-.682	.599	.335
119SOL	OW	355	.307	.213	-.631
119SOL	HW1	356	.284	.250	-.541
119SOL	HW2	357	.277	.118	-.637
120SOL	OW	358	.037	-.552	-.580
120SOL	HW1	359	.090	-.601	-.512
120SOL	HW2	360	.059	-.454	-.575
121SOL	OW	361	.732	.634	-.798
121SOL	HW1	362	.791	.608	-.874
121SOL	HW2	363	.704	.730	-.809
122SOL	OW	364	-.134	-.927	-.008
122SOL	HW1	365	-.180	-.934	-.097
122SOL	HW2	366	-.196	-.883	.058

123SOL	OW	367	.307	.063	.618
123SOL	HW1	368	.296	.157	.651
123SOL	HW2	369	.302	-.000	.695
124SOL	OW	370	-.240	.367	.374
124SOL	HW1	371	-.238	.291	.438
124SOL	HW2	372	-.288	.444	.414
125SOL	OW	373	-.839	.766	-.896
125SOL	HW1	374	-.824	.787	-.800
125SOL	HW2	375	-.869	.671	-.905
126SOL	OW	376	-.882	-.289	-.162
126SOL	HW1	377	-.902	-.245	-.250
126SOL	HW2	378	-.843	-.380	-.178
127SOL	OW	379	-.003	-.344	-.257
127SOL	HW1	380	.011	-.317	-.352
127SOL	HW2	381	.080	-.322	-.204
128SOL	OW	382	.350	.898	-.058
128SOL	HW1	383	.426	.942	-.010
128SOL	HW2	384	.385	.851	-.140
129SOL	OW	385	-.322	.274	.125
129SOL	HW1	386	-.383	.199	.148
129SOL	HW2	387	-.300	.326	.208
130SOL	OW	388	-.559	.838	.042
130SOL	HW1	389	-.525	.745	.057
130SOL	HW2	390	-.541	.865	-.053
131SOL	OW	391	-.794	-.529	.849
131SOL	HW1	392	-.787	-.613	.794
131SOL	HW2	393	-.732	-.460	.813
132SOL	OW	394	.319	.810	-.913
132SOL	HW1	395	.412	.846	-.908

132SOL	HW2	396	.313	.725	-.861
133SOL	OW	397	.339	.509	-.856
133SOL	HW1	398	.287	.426	-.873
133SOL	HW2	399	.416	.514	-.920
134SOL	OW	400	.511	.415	-.054
134SOL	HW1	401	.493	.460	.034
134SOL	HW2	402	.553	.480	-.117
135SOL	OW	403	-.724	.380	-.184
135SOL	HW1	404	-.769	.443	-.120
135SOL	HW2	405	-.631	.411	-.201
136SOL	OW	406	-.702	.207	-.385
136SOL	HW1	407	-.702	.271	-.308
136SOL	HW2	408	-.674	.255	-.468
137SOL	OW	409	.008	-.536	.200
137SOL	HW1	410	-.085	-.515	.169
137SOL	HW2	411	.018	-.635	.213
138SOL	OW	412	.088	-.061	.927
138SOL	HW1	413	.046	-.147	.900
138SOL	HW2	414	.182	-.058	.893
139SOL	OW	415	.504	-.294	.910
139SOL	HW1	416	.570	-.220	.919
139SOL	HW2	417	.548	-.373	.868
140SOL	OW	418	-.860	.796	-.624
140SOL	HW1	419	-.819	.764	-.538
140SOL	HW2	420	-.956	.769	-.627
141SOL	OW	421	.040	.544	-.748
141SOL	HW1	422	.125	.511	-.789
141SOL	HW2	423	.053	.559	-.650
142SOL	OW	424	.189	.520	-.140

142SOL	HW1	425	.248	.480	-.210
142SOL	HW2	426	.131	.591	-.181
143SOL	OW	427	-.493	-.912	-.202
143SOL	HW1	428	-.454	-.823	-.182
143SOL	HW2	429	-.483	-.932	-.299
144SOL	OW	430	.815	.572	.325
144SOL	HW1	431	.822	.483	.279
144SOL	HW2	432	.721	.606	.317
145SOL	OW	433	-.205	.604	-.656
145SOL	HW1	434	-.243	.535	-.594
145SOL	HW2	435	-.123	.568	-.700
146SOL	OW	436	.252	-.298	-.118
146SOL	HW1	437	.222	-.241	-.042
146SOL	HW2	438	.245	-.395	-.092
147SOL	OW	439	.671	.464	-.593
147SOL	HW1	440	.637	.375	-.623
147SOL	HW2	441	.697	.518	-.673
148SOL	OW	442	.930	-.184	-.397
148SOL	HW1	443	.906	-.202	-.492
148SOL	HW2	444	.960	-.090	-.387
149SOL	OW	445	.473	.500	.191
149SOL	HW1	446	.534	.580	.195
149SOL	HW2	447	.378	.531	.198
150SOL	OW	448	.159	-.725	-.396
150SOL	HW1	449	.181	-.786	-.320
150SOL	HW2	450	.169	-.774	-.482
151SOL	OW	451	-.515	-.803	-.628
151SOL	HW1	452	-.491	-.866	-.702
151SOL	HW2	453	-.605	-.763	-.646

152SOL	OW	454	-.560	.855	.309
152SOL	HW1	455	-.646	.824	.351
152SOL	HW2	456	-.564	.841	.210
153SOL	OW	457	-.103	-.115	-.708
153SOL	HW1	458	-.042	-.085	-.781
153SOL	HW2	459	-.141	-.204	-.730
154SOL	OW	460	-.610	-.131	-.734
154SOL	HW1	461	-.526	-.126	-.788
154SOL	HW2	462	-.633	-.227	-.716
155SOL	OW	463	.083	-.604	-.840
155SOL	HW1	464	.078	-.605	-.740
155SOL	HW2	465	.000	-.645	-.878
156SOL	OW	466	.688	-.200	-.146
156SOL	HW1	467	.632	-.119	-.137
156SOL	HW2	468	.740	-.196	-.232
157SOL	OW	469	.903	.086	.133
157SOL	HW1	470	.954	.087	.047
157SOL	HW2	471	.959	.044	.204
158SOL	OW	472	-.136	.135	.523
158SOL	HW1	473	-.063	.118	.456
158SOL	HW2	474	-.167	.048	.561
159SOL	OW	475	-.474	-.289	.477
159SOL	HW1	476	-.407	-.277	.403
159SOL	HW2	477	-.514	-.200	.500
160SOL	OW	478	.130	-.068	-.011
160SOL	HW1	479	.089	-.142	.042
160SOL	HW2	480	.194	-.017	.047
161SOL	OW	481	-.582	.927	.672
161SOL	HW1	482	-.522	.846	.674

161SOL	HW2	483	-.542	.996	.612
162SOL	OW	484	.830	-.589	-.440
162SOL	HW1	485	.825	-.556	-.345
162SOL	HW2	486	.744	-.570	-.486
163SOL	OW	487	.672	-.246	.154
163SOL	HW1	488	.681	-.236	.055
163SOL	HW2	489	.632	-.335	.175
164SOL	OW	490	-.212	-.142	-.468
164SOL	HW1	491	-.159	-.132	-.552
164SOL	HW2	492	-.239	-.052	-.434
165SOL	OW	493	-.021	.175	-.899
165SOL	HW1	494	.018	.090	-.935
165SOL	HW2	495	-.119	.177	-.918
166SOL	OW	496	.263	.326	.720
166SOL	HW1	497	.184	.377	.686
166SOL	HW2	498	.254	.311	.818
167SOL	OW	499	-.668	-.250	.031
167SOL	HW1	500	-.662	-.343	.068
167SOL	HW2	501	-.727	-.250	-.049
168SOL	OW	502	.822	-.860	-.490
168SOL	HW1	503	.862	-.861	-.582
168SOL	HW2	504	.832	-.768	-.450
169SOL	OW	505	.916	.910	.291
169SOL	HW1	506	.979	.948	.223
169SOL	HW2	507	.956	.827	.330
170SOL	OW	508	-.358	-.255	.044
170SOL	HW1	509	-.450	-.218	.051
170SOL	HW2	510	-.320	-.235	-.046
171SOL	OW	511	.372	-.574	-.372

171SOL	HW1	512	.359	-.481	-.406
171SOL	HW2	513	.288	-.626	-.385
172SOL	OW	514	-.248	-.570	-.573
172SOL	HW1	515	-.188	-.567	-.493
172SOL	HW2	516	-.323	-.506	-.560
173SOL	OW	517	-.823	-.764	.696
173SOL	HW1	518	-.893	-.811	.750
173SOL	HW2	519	-.764	-.832	.653
174SOL	OW	520	-.848	.236	-.891
174SOL	HW1	521	-.856	.200	-.984
174SOL	HW2	522	-.850	.160	-.826
175SOL	OW	523	.590	-.375	.491
175SOL	HW1	524	.632	-.433	.421
175SOL	HW2	525	.546	-.296	.447
176SOL	OW	526	-.153	.385	-.481
176SOL	HW1	527	-.080	.454	-.477
176SOL	HW2	528	-.125	.310	-.540
177SOL	OW	529	.255	-.514	.290
177SOL	HW1	530	.159	-.513	.263
177SOL	HW2	531	.267	-.461	.374
178SOL	OW	532	.105	-.849	-.136
178SOL	HW1	533	.028	-.882	-.082
178SOL	HW2	534	.190	-.879	-.094
179SOL	OW	535	.672	.203	-.373
179SOL	HW1	536	.762	.187	-.413
179SOL	HW2	537	.680	.208	-.274
180SOL	OW	538	.075	.345	.033
180SOL	HW1	539	-.017	.317	.004
180SOL	HW2	540	.106	.422	-.023

181SOL	OW	541	-.422	.856	-.464
181SOL	HW1	542	-.479	.908	-.527
181SOL	HW2	543	-.326	.868	-.488
182SOL	OW	544	.072	.166	.318
182SOL	HW1	545	.055	.249	.264
182SOL	HW2	546	.162	.129	.296
183SOL	OW	547	-.679	-.527	.119
183SOL	HW1	548	-.778	-.538	.121
183SOL	HW2	549	-.645	-.512	.212
184SOL	OW	550	.613	.842	-.431
184SOL	HW1	551	.669	.923	-.448
184SOL	HW2	552	.672	.762	-.428
185SOL	OW	553	-.369	-.095	-.903
185SOL	HW1	554	-.336	-.031	-.972
185SOL	HW2	555	-.303	-.101	-.828
186SOL	OW	556	.716	.565	-.154
186SOL	HW1	557	.735	.630	-.080
186SOL	HW2	558	.776	.485	-.145
187SOL	OW	559	-.412	-.642	-.229
187SOL	HW1	560	-.421	-.652	-.130
187SOL	HW2	561	-.316	-.649	-.255
188SOL	OW	562	.390	-.121	-.302
188SOL	HW1	563	.299	-.080	-.304
188SOL	HW2	564	.383	-.215	-.270
189SOL	OW	565	-.188	.883	-.608
189SOL	HW1	566	-.215	.794	-.645
189SOL	HW2	567	-.187	.951	-.681
190SOL	OW	568	-.637	.325	.449
190SOL	HW1	569	-.572	.251	.438

190SOL	HW2	570	-.617	.375	.533
191SOL	OW	571	.594	.745	.652
191SOL	HW1	572	.644	.830	.633
191SOL	HW2	573	.506	.747	.604
192SOL	OW	574	-.085	.342	-.220
192SOL	HW1	575	-.102	.373	-.314
192SOL	HW2	576	-.169	.305	-.182
193SOL	OW	577	-.132	-.928	-.345
193SOL	HW1	578	-.094	-.837	-.330
193SOL	HW2	579	-.140	-.945	-.444
194SOL	OW	580	.859	-.488	.016
194SOL	HW1	581	.813	-.473	.104
194SOL	HW2	582	.903	-.403	-.014
195SOL	OW	583	.661	-.072	-.909
195SOL	HW1	584	.615	.016	-.922
195SOL	HW2	585	.760	-.060	-.916
196SOL	OW	586	-.454	-.011	-.142
196SOL	HW1	587	-.550	-.022	-.169
196SOL	HW2	588	-.398	-.078	-.190
197SOL	OW	589	.859	-.906	.861
197SOL	HW1	590	.913	-.975	.909
197SOL	HW2	591	.827	-.837	.927
198SOL	OW	592	-.779	-.878	.087
198SOL	HW1	593	-.802	-.825	.005
198SOL	HW2	594	-.698	-.934	.068
199SOL	OW	595	-.001	-.293	.851
199SOL	HW1	596	-.072	-.305	.781
199SOL	HW2	597	.000	-.372	.911
200SOL	OW	598	.221	-.548	-.018

200SOL	HW1	599	.156	-.621	-.039
200SOL	HW2	600	.225	-.534	.080
201SOL	OW	601	.079	-.622	.653
201SOL	HW1	602	.078	-.669	.741
201SOL	HW2	603	.161	-.650	.602
202SOL	OW	604	.672	-.471	-.238
202SOL	HW1	605	.594	-.521	-.200
202SOL	HW2	606	.669	-.376	-.207
203SOL	OW	607	-.038	.192	-.635
203SOL	HW1	608	-.042	.102	-.591
203SOL	HW2	609	-.035	.181	-.734
204SOL	OW	610	.428	.424	.520
204SOL	HW1	611	.458	.352	.458
204SOL	HW2	612	.389	.384	.603
205SOL	OW	613	-.157	-.375	-.758
205SOL	HW1	614	-.250	-.400	-.785
205SOL	HW2	615	-.131	-.425	-.676
206SOL	OW	616	.317	.547	-.582
206SOL	HW1	617	.355	.488	-.510
206SOL	HW2	618	.357	.521	-.670
207SOL	OW	619	.812	-.276	.687
207SOL	HW1	620	.844	-.266	.593
207SOL	HW2	621	.733	-.338	.689
208SOL	OW	622	-.438	.214	-.750
208SOL	HW1	623	-.386	.149	-.695
208SOL	HW2	624	-.487	.277	-.689
209SOL	OW	625	-.861	.034	-.708
209SOL	HW1	626	-.924	-.038	-.739
209SOL	HW2	627	-.768	-.002	-.708

```
210SOL    OW  628   .770  -.532   .301
210SOL   HW1  629   .724  -.619   .318
210SOL   HW2  630   .861  -.535   .342
211SOL    OW  631   .618  -.295  -.578
211SOL   HW1  632   .613  -.213  -.521
211SOL   HW2  633   .707  -.298  -.623
212SOL    OW  634  -.510   .052   .168
212SOL   HW1  635  -.475   .011   .084
212SOL   HW2  636  -.600   .014   .188
213SOL    OW  637  -.562   .453   .691
213SOL   HW1  638  -.621   .533   .695
213SOL   HW2  639  -.547   .418   .784
214SOL    OW  640  -.269   .221   .882
214SOL   HW1  641  -.353   .220   .936
214SOL   HW2  642  -.267   .304   .826
215SOL    OW  643   .039  -.785   .300
215SOL   HW1  644   .138  -.796   .291
215SOL   HW2  645  -.001  -.871   .332
216SOL    OW  646   .875  -.216   .337
216SOL   HW1  647   .798  -.251   .283
216SOL   HW2  648   .843  -.145   .399
 1.86206  1.86206  1.86206
```

1.1.3 Molecular Dynamics Parameter files

Molecular dynamics parameter file for equilibrating positions: "em.mdp"

```
integrator      = steep     ; steepest descent E minimization
nsteps          = 500       ; steps
emtol           = 100       ; max force for convergence


; Bond constraints
```

```
constraints             = none              ;
constraint_algorithm    = lincs             ; default
lincs_order             = 4                 ; default

; X/V/F/E outputs
nstxout                 = 250000            ; pos out   ---  500  ps
nstvout                 = 250000            ; vel out   ---  500  ps
nstfout                 = 0                 ; force out ---  no
nstlog                  = 5000              ; energies to log (10 ps)
nstenergy               = 5000              ; energies to energy file
nstxout-compressed      = 5000              ; xtc, 10 ps
compressed-x-precision  = 1000

; Neighbour list
nstlist                 = 20                ; Freq. to update neighbour list
rlist                   = 0.8               ; nm (cutoff for short-range NL)

; Coulomb interactions
coulombtype             = Reaction-field    ;
epsilon_rf              = 80                ; water
;epsilon_rf             = 4.8               ; CHCl3 (CRC Handbook)
rcoulomb                = 1.4               ; nm (direct space sum cut-off)

; van der Waals interactions
vdwtype                 = Cut-off           ; Van der Waals interactions
rvdw                    = 1.4               ; nm (LJ cut-off)
DispCorr                = EnerPres          ; use dispersion correction

; Temperature coupling
```

```
Tcoupl                  = no                ; This is ignored with sd integrator

; Energy monitoring
energygrps              = System

; Pressure coupling
Pcoupl                  = no

; Generate velocites in the beginning
continuation            = yes               ; continue from npt equilibration
gen_vel                 = no                ; continue from npt equilibration
gen_temp                = 298.0
gen_seed                = -1                ; -1 = the seed is calculated from the process ID number
```

Molecular dynamics parameter file equilibrating under constant NPT conditions "eq.mdp"

```
integrator              = md                ;
dt                      = 0.001             ; ps
nsteps                  = 250000            ; total time: 0.250 ns
tinit                   = 0                 ; initial time, ps
nstcomm                 = 20                ; freq. for cm-motion removal
ld_seed                 = -1

; Bond constraints
constraints             = h-bonds           ; constrain all bonds
constraint_algorithm    = lincs             ; default
lincs_order             = 4                 ; default
lincs-iter              = 1                 ; accuracy of lincs algorithm

; X/V/F/E outputs
```

```
nstxout                = 500000           ; pos out   ---  1000  ps
nstvout                = 500000           ; vel out   ---  1000  ps
nstfout                = 0                ; force out ---  no
nstlog                 = 10000            ; energies to log (20 ps)
nstenergy              = 10000            ; energies to energy file
nstxout-compressed     = 10000            ; xtc, 10 ps
compressed-x-precision  = 1000

; Neighbour list
nstlist                = 20               ; Freq. to update neighbour list
rlist                  = 0.8              ; nm (cutoff for short-range NL)

; Coulomb interactions
coulombtype            = Reaction-field    ;
epsilon_rf             = 80               ; water
;epsilon_rf            = 4.8              ; CHCl3 (CRC Handbook)
rcoulomb               = 1.4              ; nm (direct space sum cut-off)

; van der Waals interactions
vdwtype                = Cut-off          ; Van der Waals interactions
rvdw                   = 1.4              ; nm (LJ cut-off)
DispCorr               = EnerPres         ; use dispersion correction

; Temperature coupling
Tcoupl                 = Berendsen        ; This is ignored with sd integrator
tc-grps                = System           ;
tau_t                  = 0.1              ; ps, recommended value for sd
ref_t                  = 298.15           ; K
```

; Energy monitoring

energygrps = System

; Pressure coupling

Pcoupl = Berendsen ;

Pcoupltype = isotropic ;

tau_p = 0.5 ; ps

compressibility = 5e-5 ; 1/bar

ref_p = 1.0 ; bar

refcoord_scaling = all

; Generate velocites in the beginning

continuation = no ; continue from npt equilibration

gen_vel = yes ; continue from npt equilibration

gen_temp = 298.0

gen_seed = -1 ; -1 = the seed is calculated from the process ID number

Molecular dynamics parameter file for production run under constant NVT condidtions "run.mdp"

integrator = md ;

dt = 0.002 ; ps

nsteps = 5000000 ; total time: 10 ns

tinit = 0 ; initial time, ps

nstcomm = 25 ; freq. for cm-motion removal

ld_seed = -1

; Bond constraints

constraints = h-bonds ; constrain H bonds

constraint_algorithm = lincs ; default

lincs_order = 4 ; default

```
lincs-iter              = 1                 ; accuracy of lincs algorithm

; X/V/F/E outputs
nstxout                 = 500000            ; pos out   ---  1000  ps
nstvout                 = 500000            ; vel out   ---  1000  ps
nstfout                 = 0                 ; force out ---  no
nstlog                  = 10000             ; energies to log (20 ps)
nstenergy               = 10000             ; energies to energy file
nstxout-compressed      = 10000             ; xtc, 10 ps
compressed-x-precision  = 1000

; Neighbour list
nstlist                 = 20                ; Freq. to update neighbour list
rlist                   = 0.8               ; nm (cutoff for short-range NL)

; Coulomb interactions
coulombtype             = Reaction-field    ;
epsilon_rf              = 80                ; water
;epsilon_rf             = 4.8               ; CHCl3 (CRC Handbook)
rcoulomb                = 1.4               ; nm (direct space sum cut-off)

; van der Waals interactions
vdwtype                 = Cut-off           ; Van der Waals interactions
rvdw                    = 1.4               ; nm (LJ cut-off)
DispCorr                = EnerPres          ; use dispersion correction

; Temperature coupling
Tcoupl                  = Nose-Hoover
tc-grps                 = System            ;
```

```
tau_t                = 1.0          ; ps
ref_t                = 298.15        ; K

; Energy monitoring
energygrps           = System

; Pressure coupling
Pcoupl               = Parrinello-Rahman
Pcoupltype           = isotropic       ;
tau_p                = 5.0          ; ps
compressibility      = 5e-5          ; 1/bar
ref_p                = 1.0          ; bar
refcoord_scaling     = all

; Generate velocites in the beginning
continuation         = yes          ; continue from npt equilibration
gen_vel              = no           ; continue from npt equilibration
gen_temp             = 298.0
gen_seed             = -1           ; -1 = the seed is calculated from the process ID number
```

1.2 Martini 3 force field

1.2.1 Topology files

Main topology file "PEG_solvated.top"

```
#include "martini_v3.0.0.itp"
#include "martini_v3.0.0_solvents_v1.itp"

; Include chain topologies
#include "PEG.itp"
```

[system]

PEG System in water

[molecules]

; Compound #mols

PEG 800

W 219163

Include topology file "martini_v3.0.0.itp" and "martini_v3.0.0_solvents_v1.itp"

Files are downloaded from https://cgmartini.nl/

Include topology file "PEG.itp"

[moleculetype]

; molname nrexcl

PEG 1

[atoms]

; nr type resnr residue atom cgnr charge mass

1 SP2 0 PEG C1 1 0 54

2 SN4a 0 PEG R1 2 0 54

3 SN4a 0 PEG R2 3 0 54

4 SN4a 0 PEG R3 4 0 54

5 SN4a 0 PEG R4 5 0 54

6 SN4a 0 PEG R5 6 0 54

7 SN4a 0 PEG R6 7 0 54

8 SN4a 0 PEG R7 8 0 54

9 SN4a 0 PEG R8 9 0 54

10 SN4a 0 PEG R9 10 0 54

11 SN4a 0 PEG R10 11 0 54

12	SN4a	0	PEG	R11	12	0	54
13	SN4a	0	PEG	R12	13	0	54
14	SN4a	0	PEG	R13	14	0	54
15	SN4a	0	PEG	R14	15	0	54
16	SN4a	0	PEG	R15	16	0	54
17	SN4a	0	PEG	R16	17	0	54
18	SN4a	0	PEG	R17	18	0	54
19	SN4a	0	PEG	R18	19	0	54
20	SN4a	0	PEG	R19	20	0	54
21	SN4a	0	PEG	R20	21	0	54
22	SN4a	0	PEG	R21	22	0	54
23	SN4a	0	PEG	R22	23	0	54
24	SN4a	0	PEG	R23	24	0	54
25	SN4a	0	PEG	R24	25	0	54
26	SN4a	0	PEG	R25	26	0	54
27	SN4a	0	PEG	R26	27	0	54
28	SN4a	0	PEG	R27	28	0	54
29	SN4a	0	PEG	R28	29	0	54
30	SN4a	0	PEG	R29	30	0	54
31	SN4a	0	PEG	R30	31	0	54
32	SN4a	0	PEG	R31	32	0	54
33	SN4a	0	PEG	R32	33	0	54
34	SN4a	0	PEG	R33	34	0	54
35	SN4a	0	PEG	R34	35	0	54
36	SN4a	0	PEG	R35	36	0	54
37	SN4a	0	PEG	R36	37	0	54
38	SN4a	0	PEG	R37	38	0	54
39	SN4a	0	PEG	R38	39	0	54
40	SN4a	0	PEG	R39	40	0	54

```
 41  SN4a  0   PEG   R40   41   0    54
 42  SN4a  0   PEG   R41   42   0    54
 43  SN4a  0   PEG   R42   43   0    54
 44  SN4a  0   PEG   R43   44   0    54
 45  SN4a  0   PEG   R44   45   0    54
 46  SP2   0   PEG   C2    46   0    54

[bonds]
; i j  funct   length   force.c.
 2 1   1     0.267300     5094
 3 2   1     0.309360     5055
 4 3   1     0.303390     5055
 5 4   1     0.305580     5055
 6 5   1     0.307870     5055
 7 6   1     0.302680     5055
 8 7   1     0.303350     5055
 9 8   1     0.306640     5055
 10 9   1     0.300900     5055
 11 10   1     0.309190     5055
 12 11   1     0.304880     5055
 13 12   1     0.308970     5055
 14 13   1     0.303120     5055
 15 14   1     0.307030     5055
 16 15   1     0.304550     5055
 17 16   1     0.305820     5055
 18 17   1     0.307740     5055
 19 18   1     0.304000     5055
 20 19   1     0.309750     5055
 21 20   1     0.302840     5055
```

22 21 1 0.303280 5055

23 22 1 0.302290 5055

24 23 1 0.306470 5055

25 24 1 0.300970 5055

26 25 1 0.307490 5055

27 26 1 0.305070 5055

28 27 1 0.297270 5055

29 28 1 0.303440 5055

30 29 1 0.302480 5055

31 30 1 0.303280 5055

32 31 1 0.301910 5055

33 32 1 0.301730 5055

34 33 1 0.306720 5055

35 34 1 0.303590 5055

36 35 1 0.303320 5055

37 36 1 0.304710 5055

38 37 1 0.303590 5055

39 38 1 0.310530 5055

40 39 1 0.300800 5055

41 40 1 0.301210 5055

42 41 1 0.309060 5055

43 42 1 0.305420 5055

44 43 1 0.305570 5055

45 44 1 0.305510 5055

46 45 1 0.268580 5094

[angles]

; i j k funct angle force_constant

3 2 1 1 131.351000 20.0

4	3	2	1	133.130000	20.0
5	4	3	1	127.169000	20.0
6	5	4	1	124.860000	20.0
7	6	5	1	129.266000	20.0
8	7	6	1	132.962000	20.0
9	8	7	1	134.692000	20.0
10	9	8	1	129.982000	20.0
11	10	9	1	129.608000	20.0
12	11	10	1	130.403000	20.0
13	12	11	1	132.421000	20.0
14	13	12	1	131.017000	20.0
15	14	13	1	132.868000	20.0
16	15	14	1	134.331000	20.0
17	16	15	1	131.207000	20.0
18	17	16	1	127.288000	20.0
19	18	17	1	127.985000	20.0
20	19	18	1	127.601000	20.0
21	20	19	1	129.599000	20.0
22	21	20	1	127.240000	20.0
23	22	21	1	125.202000	20.0
24	23	22	1	124.829000	20.0
25	24	23	1	131.094000	20.0
26	25	24	1	128.958000	20.0
27	26	25	1	132.562000	20.0
28	27	26	1	125.714000	20.0
29	28	27	1	133.299000	20.0
30	29	28	1	135.413000	20.0
31	30	29	1	130.417000	20.0
32	31	30	1	128.890000	20.0

33	32	31	1	128.703000	20.0
34	33	32	1	131.322000	20.0
35	34	33	1	131.412000	20.0
36	35	34	1	129.797000	20.0
37	36	35	1	132.205000	20.0
38	37	36	1	133.420000	20.0
39	38	37	1	133.691000	20.0
40	39	38	1	127.839000	20.0
41	40	39	1	126.839000	20.0
42	41	40	1	127.027000	20.0
43	42	41	1	131.874000	20.0
44	43	42	1	128.883000	20.0
45	44	43	1	130.421000	20.0
46	45	44	1	132.636000	20.0

1.2.2 Structure coordinate files

Structure/coordinate file “PEG.gro”

Generated with cgbuilder

46

0PEG	C1	1	7.870	0.056	0.000
0PEG	R1	2	7.575	-0.080	-0.000
0PEG	R2	3	7.223	0.077	0.000
0PEG	R3	4	6.871	-0.078	-0.000
0PEG	R4	5	6.519	0.077	0.000
0PEG	R5	6	6.166	-0.078	-0.000
0PEG	R6	7	5.814	0.078	0.000
0PEG	R7	8	5.462	-0.077	-0.000
0PEG	R8	9	5.109	0.078	0.000
0PEG	R9	10	4.757	-0.077	-0.000

0PEG	R10	11	4.405	0.078	0.000
0PEG	R11	12	4.052	-0.077	-0.000
0PEG	R12	13	3.700	0.078	0.000
0PEG	R13	14	3.348	-0.077	-0.000
0PEG	R14	15	2.995	0.078	-0.000
0PEG	R15	16	2.643	-0.078	-0.001
0PEG	R16	17	2.291	0.077	-0.000
0PEG	R17	18	1.938	-0.078	0.000
0PEG	R18	19	1.586	0.078	0.001
0PEG	R19	20	1.233	-0.077	0.002
0PEG	R20	21	0.881	0.078	0.002
0PEG	R21	22	0.529	-0.077	0.002
0PEG	R22	23	0.176	0.077	-0.001
0PEG	R23	24	-0.176	-0.078	-0.002
0PEG	R24	25	-0.529	0.077	-0.003
0PEG	R25	26	-0.881	-0.078	-0.000
0PEG	R26	27	-1.233	0.078	0.001
0PEG	R27	28	-1.586	-0.077	0.003
0PEG	R28	29	-1.938	0.078	0.000
0PEG	R29	30	-2.290	-0.077	0.001
0PEG	R30	31	-2.643	0.077	-0.002
0PEG	R31	32	-2.995	-0.079	-0.001
0PEG	R32	33	-3.348	0.077	-0.001
0PEG	R33	34	-3.700	-0.078	-0.000
0PEG	R34	35	-4.052	0.077	0.000
0PEG	R35	36	-4.405	-0.078	0.000
0PEG	R36	37	-4.757	0.077	0.000
0PEG	R37	38	-5.109	-0.078	0.000
0PEG	R38	39	-5.462	0.077	0.000

0PEG   R39   40 -5.814 -0.078 -0.000

0PEG   R40   41 -6.166   0.078   0.000

0PEG   R41   42 -6.519 -0.077 -0.000

0PEG   R42   43 -6.871   0.078   0.000

0PEG   R43   44 -7.223 -0.077 -0.000

0PEG   R44   45 -7.575   0.080   0.000

0PEG   C2   46 -7.870 -0.056 -0.000

10 10 10

Structure/coordinate file for water "box_CG_W_eq.gro"

File is downloaded from https://cgmartini.nl/

1.2.3 Molecular Dynamics Parameter files

Molecular dynamics parameter file for equilibrating positions: "em.mdp"

define = -DFLEXIBLE

integrator = steep

nsteps = 2000

nstcomm = 100

comm-grps =

nstxout = 0

nstvout = 0

nstfout = 0

nstlog = 1000

nstenergy = 100

nstxout-compressed = 1000

compressed-x-precision = 100

compressed-x-grps = System

energygrps = System

```
cutoff-scheme           = Verlet
nstlist                 = 20
ns_type                 = grid
pbc                     = xyz
verlet-buffer-tolerance  = 0.005

coulombtype             = cutoff
coulomb-modifier        = Potential-shift-verlet
rcoulomb                = 1.1
epsilon_r               = 15 ; 2.5 (with polarizable water)
vdw_type                = cutoff
vdw-modifier            = Potential-shift-verlet
rvdw                    = 1.1
tcoupl                  = no
pcoupl                  = no
gen_vel                 = no
gen_temp                = 298
gen_seed                = -1

constraints             = none
constraint_algorithm    = Lincs
```

Molecular dynamics parameter file equilibrating under constant NPT conditions "eq.mdp"

```
integrator              = md                ;
dt                      = 0.001             ; ps
nsteps                  = 250000            ; total time: 0.250 ns
tinit                   = 0                 ; initial time, ps
nstcomm                 = 20                ; freq. for cm-motion removal
ld_seed                 = -1
```

```
; Bond constraints
constraints             = h-bonds           ; constrain all bonds
constraint_algorithm    = lincs             ; default
lincs_order             = 4                 ; default
lincs-iter              = 1                 ; accuracy of lincs algorithm

; X/V/F/E outputs
nstxout                 = 500000            ; pos out   ---  1000  ps
nstvout                 = 500000            ; vel out   ---  1000  ps
nstfout                 = 0                 ; force out ---  no
nstlog                  = 10000             ; energies to log (20 ps)
nstenergy               = 10000             ; energies to energy file
nstxout-compressed      = 10000             ; xtc, 10 ps
compressed-x-precision  = 1000

; Neighbour list
nstlist                 = 20                ; Freq. to update neighbour list
rlist                   = 0.8               ; nm (cutoff for short-range NL)

; Coulomb interactions
coulombtype             = Reaction-field    ;
epsilon_rf              = 80                ; water
;epsilon_rf             = 4.8               ; CHCl3 (CRC Handbook)
rcoulomb                = 1.4               ; nm (direct space sum cut-off)

; van der Waals interactions
vdwtype                 = Cut-off           ; Van der Waals interactions
rvdw                    = 1.4               ; nm (LJ cut-off)
```

```
DispCorr                = EnerPres          ; use dispersion correction

; Temperature coupling
Tcoupl                  = Berendsen         ; This is ignored with sd integrator
tc-grps                 = System            ;
tau_t                   = 0.1               ; ps, recommended value for sd
ref_t                   = 298.15            ; K

; Energy monitoring
energygrps              = System

; Pressure coupling
Pcoupl                  = Berendsen         ;
Pcoupltype              = isotropic         ;
tau_p                   = 0.5               ; ps
compressibility         = 5e-5              ; 1/bar
ref_p                   = 1.0               ; bar
refcoord_scaling        = all

; Generate velocites in the beginning
continuation            = no                ; continue from npt equilibration
gen_vel                 = yes               ; continue from npt equilibration
gen_temp                = 298.0
gen_seed                = -1                ; -1 = the seed is calculated from the process ID number
```

Molecular dynamics parameter file for production run under constant NVT condidtions "run.mdp"

```
integrator              = md
dt                      = 0.03    ; ps
```

```
nsteps                 = 33333333 ; 1 micro second
nstcomm                = 100
comm-grps                       =

nstxout                = 0
nstvout                = 0
nstfout                = 0
nstlog                 = 1000
nstenergy              = 100
nstxout-compressed     = 1000
compressed-x-precision = 100
compressed-x-grps      = System
energygrps             = System

cutoff-scheme          = Verlet
nstlist                = 20
ns_type                = grid
pbc                    = xyz
verlet-buffer-tolerance = 0.005

coulombtype            = cutoff
coulomb-modifier       = Potential-shift-verlet
rcoulomb               = 1.1
epsilon_r              = 15 ; 2.5 (with polarizable water)
vdw_type               = cutoff
vdw-modifier           = Potential-shift-verlet
rvdw                   = 1.1
tcoupl                 = v-rescale
tc-grps                = System
```

```
tau_t                    = 1.0
ref_t                    = 298
Pcoupl                   = parrinello-rahman
Pcoupltype               = isotropic
tau_p                    = 12.0  ;parrinello-rahman is more stable with larger tau-p, DdJ, 20130422
compressibility          = 3e-4
ref_p                    =    1

gen_vel                  = no
gen_temp                 = 298
gen_seed                 = -1

constraints              = none
constraint_algorithm     = Lincs
```

5. Input files for MD simulations of HINA

5.1 General AMBER force field

1.1.1 Topology files

Main topology file “HINA_solvated.top”

```
[ defaults ]
; nbfunc      comb-rule     gen-pairs     fudgeLJ fudgeQQ
1             2             yes           0.5     0.8333333333

; Include HINA.itp topology
#include "HINA.itp"

; Ligand position restraints
#ifdef POSRES_HINA
#include "posre_HINA.itp"
#endif

; Include water topology
#include "tip3p.itp"

#ifdef POSRES_WATER
; Position restraint for each water oxygen
[ position_restraints ]
;  i funct     fcx       fcy       fcz
   1    1      1000      1000      1000
#endif

[ system ]
DRG in water
```

```
[ molecules ]
; Compound        nmols
DRG                1
 SOL             1686
```

Include topology file "HINA.itp"

```
[ atomtypes ]
;name   bond_type     mass     charge   ptype   sigma         epsilon       Amb
 OW       OW          0.00000  0.00000   A     3.15061e-01   6.36386e-01
 HW       HW          0.00000  0.00000   A     0.00000e+00   0.00000e+00
 c3       c3          0.00000  0.00000   A     3.39771e-01   4.51035e-01 ; 1.91  0.1078
 os       os          0.00000  0.00000   A     3.15610e-01   3.03758e-01 ; 1.77  0.0726
 h1       h1          0.00000  0.00000   A     2.42200e-01   8.70272e-02 ; 1.36  0.0208
 ca       ca          0.00000  0.00000   A     3.31521e-01   4.13379e-01 ; 1.86  0.0988
 ha       ha          0.00000  0.00000   A     2.62548e-01   6.73624e-02 ; 1.47  0.0161
 ce       ce          0.00000  0.00000   A     3.31521e-01   4.13379e-01 ; 1.86  0.0988
 cf       cf          0.00000  0.00000   A     3.31521e-01   4.13379e-01 ; 1.86  0.0988
 c        c           0.00000  0.00000   A     3.31521e-01   4.13379e-01 ; 1.86  0.0988
 o        o           0.00000  0.00000   A     3.04812e-01   6.12119e-01 ; 1.71  0.1463
 hc       hc          0.00000  0.00000   A     2.60018e-01   8.70272e-02 ; 1.46  0.0208
 oh       oh          0.00000  0.00000   A     3.24287e-01   3.89112e-01 ; 1.82  0.0930
 ho       ho          0.00000  0.00000   A     5.37925e-02   1.96648e-02 ; 0.30  0.0047

[ moleculetype ]
;name            nrexcl
 DRG              3

[ atoms ]
;   nr  type  resi  res  atom  cgnr     charge      mass       ; qtot   bond_type
```

1	c3	1	DRG	C	1	0.112700	12.01000	; qtot	0.113
2	os	1	DRG	O	2	-0.308900	16.00000	; qtot	-0.196
3	h1	1	DRG	H	3	0.046700	1.00800	; qtot	-0.149
4	h1	1	DRG	H1	4	0.046700	1.00800	; qtot	-0.103
5	h1	1	DRG	H2	5	0.046700	1.00800	; qtot	-0.056
6	ca	1	DRG	C1	6	0.072100	12.01000	; qtot	0.016
7	ca	1	DRG	C2	7	0.135100	12.01000	; qtot	0.151
8	ca	1	DRG	C3	8	0.112100	12.01000	; qtot	0.263
9	ca	1	DRG	C4	9	-0.201000	12.01000	; qtot	0.062
10	os	1	DRG	O1	10	-0.242200	16.00000	; qtot	-0.180
11	ca	1	DRG	C5	11	-0.199000	12.01000	; qtot	-0.379
12	ca	1	DRG	C6	12	-0.032800	12.01000	; qtot	-0.412
13	ha	1	DRG	H3	13	0.145000	1.00800	; qtot	-0.267
14	ca	1	DRG	C7	14	0.074100	12.01000	; qtot	-0.193
15	ha	1	DRG	H4	15	0.154000	1.00800	; qtot	-0.039
16	ce	1	DRG	C8	16	-0.015200	12.01000	; qtot	-0.054
17	ca	1	DRG	C9	17	-0.130000	12.01000	; qtot	-0.184
18	ca	1	DRG	C10	18	-0.130000	12.01000	; qtot	-0.314
19	cf	1	DRG	C11	19	-0.256200	12.01000	; qtot	-0.570
20	ha	1	DRG	H5	20	0.154000	1.00800	; qtot	-0.416
21	ca	1	DRG	C12	21	-0.124500	12.01000	; qtot	-0.541
22	ha	1	DRG	H6	22	0.155500	1.00800	; qtot	-0.385
23	ca	1	DRG	C13	23	-0.124500	12.01000	; qtot	-0.510
24	ha	1	DRG	H7	24	0.155500	1.00800	; qtot	-0.354
25	c	1	DRG	C14	25	0.633300	12.01000	; qtot	0.279
26	ha	1	DRG	H8	26	0.151000	1.00800	; qtot	0.430
27	ca	1	DRG	C15	27	-0.086300	12.01000	; qtot	0.344
28	ha	1	DRG	H9	28	0.141000	1.00800	; qtot	0.485
29	ha	1	DRG	H10	29	0.141000	1.00800	; qtot	0.626

```
   30   os     1   DRG    O2   30    -0.419900     16.00000 ; qtot 0.206
   31    o     1   DRG    O3   31    -0.487000     16.00000 ; qtot -0.281
   32   c3     1   DRG   C16   32    -0.037100     12.01000 ; qtot -0.318
   33   c3     1   DRG   C17   33     0.131400     12.01000 ; qtot -0.187
   34   c3     1   DRG   C18   34    -0.106400     12.01000 ; qtot -0.293
   35   hc     1   DRG   H11   35     0.056700      1.00800 ; qtot -0.236
   36   hc     1   DRG   H12   36     0.056700      1.00800 ; qtot -0.180
   37   h1     1   DRG   H13   37     0.057200      1.00800 ; qtot -0.123
   38   h1     1   DRG   H14   38     0.057200      1.00800 ; qtot -0.065
   39   hc     1   DRG   H15   39     0.058700      1.00800 ; qtot -0.007
   40   hc     1   DRG   H16   40     0.058700      1.00800 ; qtot 0.052
   41   oh     1   DRG    O4   41    -0.473100     16.00000 ; qtot -0.421
   42   ho     1   DRG   H17   42     0.421000      1.00800 ; qtot 0.000

[ bonds ]
;   ai     aj funct   r             k
     1      2   1   1.4273e-01    2.3620e+05 ;      C - O
     1      3   1   1.0969e-01    2.8804e+05 ;      C - H
     1      4   1   1.0969e-01    2.8804e+05 ;      C - H1
     1      5   1   1.0969e-01    2.8804e+05 ;      C - H2
     2      6   1   1.3688e-01    2.8731e+05 ;      O - C1
     6      7   1   1.3986e-01    2.9644e+05 ;     C1 - C2
     6      8   1   1.3986e-01    2.9644e+05 ;     C1 - C3
     7      9   1   1.3986e-01    2.9644e+05 ;     C2 - C4
     7     41   1    1.3644e-01    2.9167e+05 ;     C2 - O4
     8     10   1    1.3688e-01    2.8731e+05 ;     C3 - O1
     8     11   1    1.3986e-01    2.9644e+05 ;     C3 - C5
     9     12   1    1.3986e-01    2.9644e+05 ;     C4 - C6
     9     13   1    1.0860e-01    3.0183e+05 ;     C4 - H3
```

10	14	1	1.3688e-01	2.8731e+05 ;	O1 - C7
11	12	1	1.3986e-01	2.9644e+05 ;	C5 - C6
11	15	1	1.0860e-01	3.0183e+05 ;	C5 - H4
12	16	1	1.4800e-01	2.2748e+05 ;	C6 - C8
14	17	1	1.3986e-01	2.9644e+05 ;	C7 - C9
14	18	1	1.3986e-01	2.9644e+05 ;	C7 - C10
16	19	1	1.3561e-01	3.4249e+05 ;	C8 - C11
16	20	1	1.0880e-01	2.9923e+05 ;	C8 - H5
17	21	1	1.3986e-01	2.9644e+05 ;	C9 - C12
17	22	1	1.0860e-01	3.0183e+05 ;	C9 - H6
18	23	1	1.3986e-01	2.9644e+05 ;	C10 - C13
18	24	1	1.0860e-01	3.0183e+05 ;	C10 - H7
19	25	1	1.4819e-01	2.2612e+05 ;	C11 - C14
19	26	1	1.0885e-01	2.9859e+05 ;	C11 - H8
21	27	1	1.3986e-01	2.9644e+05 ;	C12 - C15
21	28	1	1.0860e-01	3.0183e+05 ;	C12 - H9
23	27	1	1.3986e-01	2.9644e+05 ;	C13 - C15
23	29	1	1.0860e-01	3.0183e+05 ;	C13 - H10
25	30	1	1.3621e-01	2.9398e+05 ;	C14 - O2
25	31	1	1.2190e-01	4.9421e+05 ;	C14 - O3
27	32	1	1.5147e-01	2.0410e+05 ;	C15 - C16
30	33	1	1.4273e-01	2.3620e+05 ;	O2 - C17
32	34	1	1.5354e-01	1.9154e+05 ;	C16 - C18
32	35	1	1.0962e-01	2.8891e+05 ;	C16 - H11
32	36	1	1.0962e-01	2.8891e+05 ;	C16 - H12
33	34	1	1.5354e-01	1.9154e+05 ;	C17 - C18
33	37	1	1.0969e-01	2.8804e+05 ;	C17 - H13
33	38	1	1.0969e-01	2.8804e+05 ;	C17 - H14
34	39	1	1.0962e-01	2.8891e+05 ;	C18 - H15

34 40 1 1.0962e-01 2.8891e+05 ; C18 - H16

41 42 1 9.7250e-02 4.4811e+05 ; O4 - H17

[pairs]

; ai aj funct

1 7 1 ; C - C2

1 8 1 ; C - C3

2 9 1 ; O - C4

2 10 1 ; O - O1

2 11 1 ; O - C5

2 41 1 ; O - O4

3 6 1 ; H - C1

4 6 1 ; H1 - C1

5 6 1 ; H2 - C1

6 12 1 ; C1 - C6

6 13 1 ; C1 - H3

6 14 1 ; C1 - C7

6 15 1 ; C1 - H4

6 42 1 ; C1 - H17

7 10 1 ; C2 - O1

7 11 1 ; C2 - C5

7 16 1 ; C2 - C8

8 9 1 ; C3 - C4

8 16 1 ; C3 - C8

8 17 1 ; C3 - C9

8 18 1 ; C3 - C10

8 41 1 ; C3 - O4

9 15 1 ; C4 - H4

9 19 1 ; C4 - C11

9	20	1 ;	C4 - H5
9	42	1 ;	C4 - H17
10	12	1 ;	O1 - C6
10	15	1 ;	O1 - H4
10	21	1 ;	O1 - C12
10	22	1 ;	O1 - H6
10	23	1 ;	O1 - C13
10	24	1 ;	O1 - H7
11	13	1 ;	C5 - H3
11	14	1 ;	C5 - C7
11	19	1 ;	C5 - C11
11	20	1 ;	C5 - H5
12	25	1 ;	C6 - C14
12	26	1 ;	C6 - H8
12	41	1 ;	C6 - O4
13	16	1 ;	H3 - C8
13	41	1 ;	H3 - O4
14	27	1 ;	C7 - C15
14	28	1 ;	C7 - H9
14	29	1 ;	C7 - H10
15	16	1 ;	H4 - C8
16	30	1 ;	C8 - O2
16	31	1 ;	C8 - O3
17	23	1 ;	C9 - C13
17	24	1 ;	C9 - H7
17	32	1 ;	C9 - C16
18	21	1 ;	C10 - C12
18	22	1 ;	C10 - H6
18	32	1 ;	C10 - C16

19	33	1 ;	C11 - C17
20	25	1 ;	H5 - C14
20	26	1 ;	H5 - H8
21	29	1 ;	C12 - H10
21	34	1 ;	C12 - C18
21	35	1 ;	C12 - H11
21	36	1 ;	C12 - H12
22	27	1 ;	H6 - C15
22	28	1 ;	H6 - H9
23	28	1 ;	C13 - H9
23	34	1 ;	C13 - C18
23	35	1 ;	C13 - H11
23	36	1 ;	C13 - H12
24	27	1 ;	H7 - C15
24	29	1 ;	H7 - H10
25	34	1 ;	C14 - C18
25	37	1 ;	C14 - H13
25	38	1 ;	C14 - H14
26	30	1 ;	H8 - O2
26	31	1 ;	H8 - O3
27	33	1 ;	C15 - C17
27	39	1 ;	C15 - H15
27	40	1 ;	C15 - H16
28	32	1 ;	H9 - C16
29	32	1 ;	H10 - C16
30	32	1 ;	O2 - C16
30	39	1 ;	O2 - H15
30	40	1 ;	O2 - H16
31	33	1 ;	O3 - C17

32 37 1 ; C16 - H13

32 38 1 ; C16 - H14

33 35 1 ; C17 - H11

33 36 1 ; C17 - H12

35 39 1 ; H11 - H15

35 40 1 ; H11 - H16

36 39 1 ; H12 - H15

36 40 1 ; H12 - H16

37 39 1 ; H13 - H15

37 40 1 ; H13 - H16

38 39 1 ; H14 - H15

38 40 1 ; H14 - H16

[angles]

; ai aj ak funct theta cth

1 2 6 1 1.1839e+02 7.3856e+02 ; C - O - C1

2 1 3 1 1.1034e+02 4.7028e+02 ; O - C - H

2 1 4 1 1.1034e+02 4.7028e+02 ; O - C - H1

2 1 5 1 1.1034e+02 4.7028e+02 ; O - C - H2

2 6 7 1 1.1911e+02 6.6107e+02 ; O - C1 - C2

2 6 8 1 1.1911e+02 6.6107e+02 ; O - C1 - C3

3 1 4 1 1.0855e+02 2.9824e+02 ; H - C - H1

3 1 5 1 1.0855e+02 2.9824e+02 ; H - C - H2

4 1 5 1 1.0855e+02 2.9824e+02 ; H1 - C - H2

6 7 9 1 1.2002e+02 5.3279e+02 ; C1 - C2 - C4

6 7 41 1 1.1990e+02 6.5990e+02 ; C1 - C2 - O4

6 8 10 1 1.1911e+02 6.6107e+02 ; C1 - C3 - O1

6 8 11 1 1.2002e+02 5.3279e+02 ; C1 - C3 - C5

7 6 8 1 1.2002e+02 5.3279e+02 ; C2 - C1 - C3

7	9	12	1	1.2002e+02	5.3279e+02 ;	C2	-	C4	-	C6		
7	9	13	1	1.1988e+02	3.7572e+02 ;	C2	-	C4	-	H3		
7	41	42	1	1.0858e+02	5.6409e+02 ;	C2	-	O4	-	H17		
8	10	14	1	1.1987e+02	7.5036e+02 ;	C3	-	O1	-	C7		
8	11	12	1	1.2002e+02	5.3279e+02 ;	C3	-	C5	-	C6		
8	11	15	1	1.1988e+02	3.7572e+02 ;	C3	-	C5	-	H4		
9	7	41	1	1.1990e+02	6.5990e+02 ;	C4	-	C2	-	O4		
9	12	11	1	1.2002e+02	5.3279e+02 ;	C4	-	C6	-	C5		
9	12	16	1	1.2079e+02	5.1530e+02 ;	C4	-	C6	-	C8		
10	8	11	1	1.1911e+02	6.6107e+02 ;	O1	-	C3	-	C5		
10	14	17	1	1.1911e+02	6.6107e+02 ;	O1	-	C7	-	C9		
10	14	18	1	1.1911e+02	6.6107e+02 ;	O1	-	C7	-	C10		
11	12	16	1	1.2079e+02	5.1530e+02 ;	C5	-	C6	-	C8		
12	9	13	1	1.1988e+02	3.7572e+02 ;	C6	-	C4	-	H3		
12	11	15	1	1.1988e+02	3.7572e+02 ;	C6	-	C5	-	H4		
12	16	19	1	1.2742e+02	5.0810e+02 ;	C6	-	C8	-	C11		
12	16	20	1	1.1503e+02	3.6560e+02 ;	C6	-	C8	-	H5		
14	17	21	1	1.2002e+02	5.3279e+02 ;	C7	-	C9	-	C12		
14	17	22	1	1.1988e+02	3.7572e+02 ;	C7	-	C9	-	H6		
14	18	23	1	1.2002e+02	5.3279e+02 ;	C7	-	C10	-	C13		
14	18	24	1	1.1988e+02	3.7572e+02 ;	C7	-	C10	-	H7		
16	19	25	1	1.2162e+02	5.1965e+02 ;	C8	-	C11	-	C14		
16	19	26	1	1.1812e+02	3.8769e+02 ;	C8	-	C11	-	H8		
17	14	18	1	1.2002e+02	5.3279e+02 ;	C9	-	C7	-	C10		
17	21	27	1	1.2002e+02	5.3279e+02 ;	C9	-	C12	-	C15		
17	21	28	1	1.1988e+02	3.7572e+02 ;	C9	-	C12	-	H9		
18	23	27	1	1.2002e+02	5.3279e+02 ;	C10	-	C13	-	C15		
18	23	29	1	1.1988e+02	3.7572e+02 ;	C10	-	C13	-	H10		
19	16	20	1	1.1843e+02	3.8727e+02 ;	C11	-	C8	-	H5		

19	25	30	1	1.1067e+02	6.6509e+02 ;	C11 - C14	- O2
19	25	31	1	1.2300e+02	6.5421e+02 ;	C11 - C14	- O3
21	17	22	1	1.1988e+02	3.7572e+02 ;	C12 - C9	- H6
21	27	23	1	1.2002e+02	5.3279e+02 ;	C12 - C15	- C13
21	27	32	1	1.2083e+02	5.0827e+02 ;	C12 - C15	- C16
23	18	24	1	1.1988e+02	3.7572e+02 ;	C13 - C10	- H7
23	27	32	1	1.2083e+02	5.0827e+02 ;	C13 - C15	- C16
25	19	26	1	1.1664e+02	3.6267e+02 ;	C14 - C11	- H8
25	30	33	1	1.1582e+02	7.4835e+02 ;	C14 - O2	- C17
27	21	28	1	1.1988e+02	3.7572e+02 ;	C15 - C12	- H9
27	23	29	1	1.1988e+02	3.7572e+02 ;	C15 - C13	- H10
27	32	34	1	1.1242e+02	5.0484e+02 ;	C15 - C16	- C18
27	32	35	1	1.1063e+02	3.6493e+02 ;	C15 - C16	- H11
27	32	36	1	1.1063e+02	3.6493e+02 ;	C15 - C16	- H12
30	25	31	1	1.2320e+02	8.4734e+02 ;	O2 - C14	- O3
30	33	34	1	1.0762e+02	6.4802e+02 ;	O2 - C17	- C18
30	33	37	1	1.1034e+02	4.7028e+02 ;	O2 - C17	- H13
30	33	38	1	1.1034e+02	4.7028e+02 ;	O2 - C17	- H14
32	34	33	1	1.1263e+02	5.0099e+02 ;	C16 - C18	- C17
32	34	39	1	1.0968e+02	3.6208e+02 ;	C16 - C18	- H15
32	34	40	1	1.0968e+02	3.6208e+02 ;	C16 - C18	- H16
33	34	39	1	1.0968e+02	3.6208e+02 ;	C17 - C18	- H15
33	34	40	1	1.0968e+02	3.6208e+02 ;	C17 - C18	- H16
34	32	35	1	1.0968e+02	3.6208e+02 ;	C18 - C16	- H11
34	32	36	1	1.0968e+02	3.6208e+02 ;	C18 - C16	- H12
34	33	37	1	1.0959e+02	3.6217e+02 ;	C18 - C17	- H13
34	33	38	1	1.0959e+02	3.6217e+02 ;	C18 - C17	- H14
35	32	36	1	1.0773e+02	2.9957e+02 ;	H11 - C16	- H12
37	33	38	1	1.0855e+02	2.9824e+02 ;	H13 - C17	- H14

```
   39    34    40     1    1.0773e+02    2.9957e+02 ;    H15 - C18    - H16

[ dihedrals ] ; propers
; for gromacs 4.5 or higher, using funct 9
;    i     j     k     l   func   phase     kd      pn
     1     2     6     7     9   180.00   6.94544   2 ;     C-    O-    C1-    C2
     1     2     6     8     9   180.00   6.94544   2 ;     C-    O-    C1-    C3
     2     6     7     9     9   180.00  15.16700   2 ;     O-    C1-    C2-    C4
     2     6     7    41     9   180.00  15.16700   2 ;     O-    C1-    C2-    O4
     2     6     8    10     9   180.00  15.16700   2 ;     O-    C1-    C3-    O1
     2     6     8    11     9   180.00  15.16700   2 ;     O-    C1-    C3-    C5
     3     1     2     6     9     0.00   1.60387   3 ;     H-    C-    O-    C1
     4     1     2     6     9     0.00   1.60387   3 ;    H1-    C-    O-    C1
     5     1     2     6     9     0.00   1.60387   3 ;    H2-    C-    O-    C1
     6     7     9    12     9   180.00  15.16700   2 ;    C1-    C2-    C4-    C6
     6     7     9    13     9   180.00  15.16700   2 ;    C1-    C2-    C4-    H3
     6     7    41    42     9   180.00   3.57732   2 ;    C1-    C2-    O4-   H17
     6     8    10    14     9   180.00   3.76560   2 ;    C1-    C3-    O1-    C7
     6     8    11    12     9   180.00  15.16700   2 ;    C1-    C3-    C5-    C6
     6     8    11    15     9   180.00  15.16700   2 ;    C1-    C3-    C5-    H4
     7     6     8    10     9   180.00  15.16700   2 ;    C2-    C1-    C3-    O1
     7     6     8    11     9   180.00  15.16700   2 ;    C2-    C1-    C3-    C5
     7     9    12    11     9   180.00  15.16700   2 ;    C2-    C4-    C6-    C5
     7     9    12    16     9   180.00  15.16700   2 ;    C2-    C4-    C6-    C8
     8     6     7     9     9   180.00  15.16700   2 ;    C3-    C1-    C2-    C4
     8     6     7    41     9   180.00  15.16700   2 ;    C3-    C1-    C2-    O4
     8    10    14    17     9   180.00   3.76560   2 ;    C3-    O1-    C7-    C9
     8    10    14    18     9   180.00   3.76560   2 ;    C3-    O1-    C7-   C10
     8    11    12     9     9   180.00  15.16700   2 ;    C3-    C5-    C6-    C4
```

8	11	12	16	9	180.00	15.16700	2	;	C3-	C5-	C6-	C8
9	7	41	42	9	180.00	3.57732	2	;	C4-	C2-	O4-	H17
9	12	11	15	9	180.00	15.16700	2	;	C4-	C6-	C5-	H4
9	12	16	19	9	180.00	2.44764	2	;	C4-	C6-	C8-	C11
9	12	16	20	9	180.00	2.92880	2	;	C4-	C6-	C8-	H5
10	8	11	12	9	180.00	15.16700	2	;	O1-	C3-	C5-	C6
10	8	11	15	9	180.00	15.16700	2	;	O1-	C3-	C5-	H4
10	14	17	21	9	180.00	15.16700	2	;	O1-	C7-	C9-	C12
10	14	17	22	9	180.00	15.16700	2	;	O1-	C7-	C9-	H6
10	14	18	23	9	180.00	15.16700	2	;	O1-	C7-	C10-	C13
10	14	18	24	9	180.00	15.16700	2	;	O1-	C7-	C10-	H7
11	8	10	14	9	180.00	3.76560	2	;	C5-	C3-	O1-	C7
11	12	9	13	9	180.00	15.16700	2	;	C5-	C6-	C4-	H3
11	12	16	19	9	180.00	2.44764	2	;	C5-	C6-	C8-	C11
11	12	16	20	9	180.00	2.92880	2	;	C5-	C6-	C8-	H5
12	9	7	41	9	180.00	15.16700	2	;	C6-	C4-	C2-	O4
12	16	19	25	9	180.00	27.82360	2	;	C6-	C8-	C11-	C14
12	16	19	26	9	180.00	27.82360	2	;	C6-	C8-	C11-	H8
13	9	7	41	9	180.00	15.16700	2	;	H3-	C4-	C2-	O4
13	9	12	16	9	180.00	15.16700	2	;	H3-	C4-	C6-	C8
14	17	21	27	9	180.00	15.16700	2	;	C7-	C9-	C12-	C15
14	17	21	28	9	180.00	15.16700	2	;	C7-	C9-	C12-	H9
14	18	23	27	9	180.00	15.16700	2	;	C7-	C10-	C13-	C15
14	18	23	29	9	180.00	15.16700	2	;	C7-	C10-	C13-	H10
15	11	12	16	9	180.00	15.16700	2	;	H4-	C5-	C6-	C8
16	19	25	30	9	180.00	9.10020	2	;	C8-	C11-	C14-	O2
16	19	25	31	9	180.00	9.10020	2	;	C8-	C11-	C14-	O3
17	14	18	23	9	180.00	15.16700	2	;	C9-	C7-	C10-	C13
17	14	18	24	9	180.00	15.16700	2	;	C9-	C7-	C10-	H7

17	21	27	23	9	180.00	15.16700	2	;	C9-	C12-	C15-	C13
17	21	27	32	9	180.00	15.16700	2	;	C9-	C12-	C15-	C16
18	14	17	21	9	180.00	15.16700	2	;	C10-	C7-	C9-	C12
18	14	17	22	9	180.00	15.16700	2	;	C10-	C7-	C9-	H6
18	23	27	21	9	180.00	15.16700	2	;	C10-	C13-	C15-	C12
18	23	27	32	9	180.00	15.16700	2	;	C10-	C13-	C15-	C16
19	25	30	33	9	180.00	11.29680	2	;	C11-	C14-	O2-	C17
20	16	19	25	9	180.00	27.82360	2	;	H5-	C8-	C11-	C14
20	16	19	26	9	180.00	27.82360	2	;	H5-	C8-	C11-	H8
21	27	23	29	9	180.00	15.16700	2	;	C12-	C15-	C13-	H10
21	27	32	34	9	180.00	1.00416	2	;	C12-	C15-	C16-	C18
21	27	32	35	9	0.00	0.00000	0	;	C12-	C15-	C16-	H11
21	27	32	36	9	0.00	0.00000	0	;	C12-	C15-	C16-	H12
22	17	21	27	9	180.00	15.16700	2	;	H6-	C9-	C12-	C15
22	17	21	28	9	180.00	15.16700	2	;	H6-	C9-	C12-	H9
23	27	21	28	9	180.00	15.16700	2	;	C13-	C15-	C12-	H9
23	27	32	34	9	180.00	1.00416	2	;	C13-	C15-	C16-	C18
23	27	32	35	9	0.00	0.00000	0	;	C13-	C15-	C16-	H11
23	27	32	36	9	0.00	0.00000	0	;	C13-	C15-	C16-	H12
24	18	23	27	9	180.00	15.16700	2	;	H7-	C10-	C13-	C15
24	18	23	29	9	180.00	15.16700	2	;	H7-	C10-	C13-	H10
25	30	33	34	9	0.00	1.60247	3	;	C14-	O2-	C17-	C18
25	30	33	34	9	180.00	3.34720	1	;	C14-	O2-	C17-	C18
25	30	33	37	9	0.00	1.60387	3	;	C14-	O2-	C17-	H13
25	30	33	38	9	0.00	1.60387	3	;	C14-	O2-	C17-	H14
26	19	25	30	9	180.00	9.10020	2	;	H8-	C11-	C14-	O2
26	19	25	31	9	180.00	9.10020	2	;	H8-	C11-	C14-	O3
27	32	34	33	9	0.00	0.65084	3	;	C15-	C16-	C18-	C17
27	32	34	39	9	0.00	0.65084	3	;	C15-	C16-	C18-	H15

```
27   32   34   40   9   0.00  0.65084  3 ;  C15-  C16-  C18-  H16
28   21   27   32   9  180.00  15.16700  2 ;  H9-  C12-  C15-  C16
29   23   27   32   9  180.00  15.16700  2 ;  H10-  C13-  C15-  C16
30   33   34   32   9   0.00  0.65084  3 ;  O2-  C17-  C18-  C16
30   33   34   39   9   0.00  0.00000  0 ;  O2-  C17-  C18-  H15
30   33   34   39   9   0.00  1.04600  1 ;  O2-  C17-  C18-  H15
30   33   34   40   9   0.00  0.00000  0 ;  O2-  C17-  C18-  H16
30   33   34   40   9   0.00  1.04600  1 ;  O2-  C17-  C18-  H16
31   25   30   33   9  180.00   5.85760  1 ;  O3-  C14-  O2-  C17
31   25   30   33   9  180.00  11.29680  2 ;  O3-  C14-  O2-  C17
32   34   33   37   9   0.00  0.65084  3 ;  C16-  C18-  C17-  H13
32   34   33   38   9   0.00  0.65084  3 ;  C16-  C18-  C17-  H14
33   34   32   35   9   0.00  0.54392  3 ;  C17-  C18-  C16-  H11
33   34   32   36   9   0.00  0.54392  3 ;  C17-  C18-  C16-  H12
35   32   34   39   9   0.00  0.50208  3 ;  H11-  C16-  C18-  H15
35   32   34   40   9   0.00  0.50208  3 ;  H11-  C16-  C18-  H16
36   32   34   39   9   0.00  0.50208  3 ;  H12-  C16-  C18-  H15
36   32   34   40   9   0.00  0.50208  3 ;  H12-  C16-  C18-  H16
37   33   34   39   9   0.00  0.65084  3 ;  H13-  C17-  C18-  H15
37   33   34   40   9   0.00  0.65084  3 ;  H13-  C17-  C18-  H16
38   33   34   39   9   0.00  0.65084  3 ;  H14-  C17-  C18-  H15
38   33   34   40   9   0.00  0.65084  3 ;  H14-  C17-  C18-  H16

[ dihedrals ] ; impropers
; treated as propers in GROMACS to use correct AMBER analytical function
;   i    j    k    l  func  phase   kd    pn
   6    9    7   41   4  180.00  4.60240  2 ;  C1-  C4-  C2-  O4
   6   11    8   10   4  180.00  4.60240  2 ;  C1-  C5-  C3-  O1
   7    8    6    2   4  180.00  4.60240  2 ;  C2-  C3-  C1-  O
```

7	12	9	13	4	180.00	4.60240	2	;	C2-	C6-	C4-	H3
8	12	11	15	4	180.00	4.60240	2	;	C3-	C6-	C5-	H4
9	11	12	16	4	180.00	4.60240	2	;	C4-	C5-	C6-	C8
12	19	16	20	4	180.00	4.60240	2	;	C6-	C11-	C8-	H5
14	21	17	22	4	180.00	4.60240	2	;	C7-	C12-	C9-	H6
14	23	18	24	4	180.00	4.60240	2	;	C7-	C13-	C10-	H7
17	18	14	10	4	180.00	4.60240	2	;	C9-	C10-	C7-	O1
17	27	21	28	4	180.00	4.60240	2	;	C9-	C15-	C12-	H9
18	27	23	29	4	180.00	4.60240	2	;	C10-	C15-	C13-	H10
19	31	25	30	4	180.00	4.60240	2	;	C11-	O3-	C14-	O2
21	23	27	32	4	180.00	4.60240	2	;	C12-	C13-	C15-	C16
25	16	19	26	4	180.00	4.60240	2	;	C14-	C8-	C11-	H8

Include topology file "posre_HINA.itp"

[position_restraints]

; atom	type	fx	fy	fz
1	1	1000	1000	1000
2	1	1000	1000	1000
6	1	1000	1000	1000
7	1	1000	1000	1000
8	1	1000	1000	1000
9	1	1000	1000	1000
10	1	1000	1000	1000
11	1	1000	1000	1000
12	1	1000	1000	1000
14	1	1000	1000	1000
16	1	1000	1000	1000
17	1	1000	1000	1000
18	1	1000	1000	1000

```
19    1  1000  1000  1000
21    1  1000  1000  1000
23    1  1000  1000  1000
25    1  1000  1000  1000
27    1  1000  1000  1000
30    1  1000  1000  1000
31    1  1000  1000  1000
32    1  1000  1000  1000
33    1  1000  1000  1000
34    1  1000  1000  1000
41    1  1000  1000  1000
```

1.2.2 Structure coordinate files

Structure/coordinate file "HINA.gro"

```
HINA structure
 42
    1  DRG    C    1   0.484  -0.154  -0.065
    1  DRG    O    2   0.442  -0.056   0.036
    1  DRG    H    3   0.413  -0.236  -0.071
    1  DRG   H1    4   0.580  -0.190  -0.029
    1  DRG   H2    5   0.496  -0.106  -0.162
    1  DRG   C1    6   0.326   0.017   0.012
    1  DRG   C2    7   0.333   0.157   0.022
    1  DRG   C3    8   0.201  -0.041  -0.013
    1  DRG   C4    9   0.218   0.237   0.011
    1  DRG   O1   10   0.190  -0.183  -0.018
    1  DRG   C5   11   0.089   0.039  -0.029
    1  DRG   C6   12   0.093   0.177  -0.013
    1  DRG   H3   13   0.226   0.344   0.024
```

1	DRG	C7	14	0.055	-0.227	-0.004
1	DRG	H4	15	-0.005	-0.008	-0.055
1	DRG	C8	16	-0.034	0.252	-0.018
1	DRG	C9	17	-0.022	-0.251	-0.118
1	DRG	C10	18	-0.004	-0.224	0.123
1	DRG	C11	19	-0.153	0.192	0.004
1	DRG	H5	20	-0.031	0.358	-0.041
1	DRG	C12	21	-0.162	-0.256	-0.105
1	DRG	H6	22	0.025	-0.255	-0.214
1	DRG	C13	23	-0.143	-0.230	0.134
1	DRG	H7	24	0.059	-0.209	0.210
1	DRG	C14	25	-0.283	0.257	-0.010
1	DRG	H8	26	-0.154	0.089	0.034
1	DRG	C15	27	-0.224	-0.238	0.019
1	DRG	H9	28	-0.222	-0.268	-0.194
1	DRG	H10	29	-0.188	-0.222	0.231
1	DRG	O2	30	-0.395	0.172	-0.020
1	DRG	O3	31	-0.303	0.379	-0.011
1	DRG	C16	32	-0.374	-0.212	0.031
1	DRG	C17	33	-0.385	0.030	-0.059
1	DRG	C18	34	-0.406	-0.062	0.061
1	DRG	H11	35	-0.415	-0.273	0.112
1	DRG	H12	36	-0.425	-0.244	-0.060
1	DRG	H13	37	-0.464	0.017	-0.132
1	DRG	H14	38	-0.289	0.011	-0.108
1	DRG	H15	39	-0.343	-0.029	0.144
1	DRG	H16	40	-0.509	-0.053	0.094
1	DRG	O4	41	0.458	0.212	0.045
1	DRG	H17	42	0.454	0.308	0.055

21.79400 13.02600 8.91600

1.1.3 Molecular Dynamics Parameter files

Molecular dynamics parameter file for equilibrating positions: “em.mdp”

```
integrator            = steep          ; steepest descent E minimization
nsteps                = 500            ; steps
emtol                 = 100            ; max force for convergence

; Bond constraints
constraints           = none           ;
constraint_algorithm  = lincs          ; default
lincs_order           = 4              ; default

; X/V/F/E outputs
nstxout               = 250000         ; pos out   ---  500  ps
nstvout               = 250000         ; vel out   ---  500  ps
nstfout               = 0              ; force out ---  no
nstlog                = 5000           ; energies to log (10 ps)
nstenergy             = 5000           ; energies to energy file
nstxout-compressed    = 5000           ; xtc, 10 ps
compressed-x-precision  = 1000

; Neighbour list
nstlist               = 20             ; Freq. to update neighbour list
rlist                 = 0.8            ; nm (cutoff for short-range NL)

; Coulomb interactions
coulombtype           = Reaction-field    ;
epsilon_rf            = 80             ; water
;epsilon_rf           = 4.8            ; CHCl3 (CRC Handbook)
```

```
rcoulomb                = 1.4             ; nm (direct space sum cut-off)

; van der Waals interactions
vdwtype                 = Cut-off         ; Van der Waals interactions
rvdw                    = 1.4             ; nm (LJ cut-off)
DispCorr                = EnerPres        ; use dispersion correction

; Temperature coupling
Tcoupl                  = no              ; This is ignored with sd integrator

; Energy monitoring
energygrps              = System

; Pressure coupling
Pcoupl                  = no

; Generate velocites in the beginning
continuation            = yes             ; continue from npt equilibration
gen_vel                 = no              ; continue from npt equilibration
gen_temp                = 298.0
gen_seed                = -1              ; -1 = the seed is calculated from the process ID number
```

Molecular dynamics parameter file equilibrating under constant NPT conditions "eq.mdp"

```
integrator              = md              ;
dt                      = 0.001           ; ps
nsteps                  = 250000          ; total time: 0.250 ns
tinit                   = 0               ; initial time, ps
nstcomm                 = 20              ; freq. for cm-motion removal
ld_seed                 = -1
```

```
; Bond constraints
constraints              = h-bonds          ; constrain all bonds
constraint_algorithm    = lincs             ; default
lincs_order             = 4                 ; default
lincs-iter              = 1                 ; accuracy of lincs algorithm

; X/V/F/E outputs
nstxout                 = 500000           ; pos out   ---  1000  ps
nstvout                 = 500000           ; vel out   ---  1000  ps
nstfout                 = 0                 ; force out ---  no
nstlog                  = 10000            ; energies to log (20 ps)
nstenergy               = 10000            ; energies to energy file
nstxout-compressed      = 10000            ; xtc, 10 ps
compressed-x-precision  = 1000

; Neighbour list
nstlist                 = 20               ; Freq. to update neighbour list
rlist                   = 0.8              ; nm (cutoff for short-range NL)

; Coulomb interactions
coulombtype             = Reaction-field    ;
epsilon_rf              = 80               ; water
;epsilon_rf             = 4.8              ; CHCl3 (CRC Handbook)
rcoulomb                = 1.4              ; nm (direct space sum cut-off)

; van der Waals interactions
vdwtype                 = Cut-off          ; Van der Waals interactions
rvdw                    = 1.4              ; nm (LJ cut-off)
```

```
DispCorr                = EnerPres          ; use dispersion correction

; Temperature coupling
Tcoupl                  = Berendsen         ; This is ignored with sd integrator
tc-grps                 = System            ;
tau_t                   = 0.1               ; ps, recommended value for sd
ref_t                   = 298.15            ; K

; Energy monitoring
energygrps              = System

; Pressure coupling
Pcoupl                  = Berendsen         ;
Pcoupltype              = isotropic         ;
tau_p                   = 0.5               ; ps
compressibility         = 5e-5              ; 1/bar
ref_p                   = 1.0               ; bar
refcoord_scaling        = all

; Generate velocites in the beginning
continuation            = no                ; continue from npt equilibration
gen_vel                 = yes               ; continue from npt equilibration
gen_temp                = 298.0
gen_seed                = -1                ; -1 = the seed is calculated from the process ID number
```

Molecular dynamics parameter file for production run under constant NVT condidtions "run.mdp"

```
integrator              = md                ;
dt                      = 0.002             ; ps
nsteps                  =  5000000          ; total time:  10 ns
```

```
tinit                   = 0                 ; initial time, ps
nstcomm                 = 25                ; freq. for cm-motion removal
ld_seed                 = -1

; Bond constraints
constraints             = h-bonds           ; constrain H bonds
constraint_algorithm    = lincs             ; default
lincs_order             = 4                 ; default
lincs-iter              = 1                 ; accuracy of lincs algorithm

; X/V/F/E outputs
nstxout                 = 500000            ; pos out   ---  1000  ps
nstvout                 = 500000            ; vel out   ---  1000  ps
nstfout                 = 0                 ; force out ---  no
nstlog                  = 10000             ; energies to log (20 ps)
nstenergy               = 10000             ; energies to energy file
nstxout-compressed      = 10000             ; xtc, 10 ps
compressed-x-precision  = 1000

; Neighbour list
nstlist                 = 20                ; Freq. to update neighbour list
rlist                   = 0.8               ; nm (cutoff for short-range NL)

; Coulomb interactions
coulombtype             = Reaction-field    ;
epsilon_rf              = 80                ; water
;epsilon_rf             = 4.8               ; CHCl3 (CRC Handbook)
rcoulomb                = 1.4               ; nm (direct space sum cut-off)
```

```
; van der Waals interactions
vdwtype              = Cut-off        ; Van der Waals interactions
rvdw                 = 1.4            ; nm (LJ cut-off)
DispCorr             = EnerPres       ; use dispersion correction

; Temperature coupling
Tcoupl               = Nose-Hoover
tc-grps              = System         ;
tau_t                = 1.0            ; ps
ref_t                = 298.15         ; K

; Energy monitoring
energygrps           = System

; Pressure coupling
Pcoupl               = Parrinello-Rahman
Pcoupltype           = isotropic      ;
tau_p                = 5.0            ; ps
compressibility      = 5e-5           ; 1/bar
ref_p                = 1.0            ; bar
refcoord_scaling     = all

; Generate velocites in the beginning
continuation         = yes            ; continue from npt equilibration
gen_vel              = no             ; continue from npt equilibration
gen_temp             = 298.0
gen_seed             = -1             ; -1 = the seed is calculated from the process ID number
```

2.2 Martini 3 force field

2.2.1 Topology files

Main topology file “HINA_solvated.top”

```
#include "martini_v3.0.0.itp"

#include "martini_v3.0.0_solvents_v1.itp"


; Include chain topologies

#include "PEG.itp"

#include "HINA.itp"


[ system ]

PEG-DRUG System in water


[ molecules ]

; Compound        #mols

PEG               200

DRG                400

W              227178
```

Include topology file "HINA.itp"

```
;;;;;; DRG


[ moleculetype ]

; molname    nrexcl

  DRG        1


[ atoms ]

; nr type resnr residue atom cgnr charge mass
```

```
  1  SN5a   0    DRG    D1    1    0     54
  2  SC4    0    DRG    D2    2    0     54
  3  TN6    0    DRG    D3    3    0     36
  4  SN2a   0    DRG    D4    4    0     54
  5  N5a    0    DRG    D5    5    0     72
  6  SC5    0    DRG    D6    6    0     54
  7  C3     0    DRG    D7    7    0     72

[bonds]
; i j  funct    length   force.c.
  4 3   1      0.27589     12310.17
  5 4   1      0.34518     11989.47
  7 1   1      0.33814     18215.38
  6 1   1      0.49359      6593.49

[constraints]
; i j  funct    length
  2 1   1      0.39021     69913.13
  3 2   1      0.31932     45991.86
  6 5   1      0.27118     315210.23
  7 6   1      0.26949     30966.74
  5 2   1      0.36106     32558.01

[angles]
; i     j       k       funct   angle   force_constant
  3     2       1         1     157.501    129.57
  4     3       2         1     89.8227    782.71
  5     4       3         1     103.214   1063.19
  6     5       4         1     161.101    103.12
```

```
 7     6       5          1     134.076    473.85
```

Structure/coordinate file "HINA.gro"

```
HINA structure
   7
   1DRG    D1    1   2.833   0.591   2.218
   1DRG    D2    2   2.547   0.673   2.470
   1DRG    D3    3   2.429   0.683   2.770
   1DRG    D4    4   2.221   0.506   2.682
   1DRG    D5    5   2.324   0.406   2.395
   1DRG    D6    6   2.489   0.242   2.256
   1DRG    D7    7   2.734   0.271   2.148
  3.72616   3.72616   3.72616
```

2.2.3 Molecular Dynamics Parameter files

Molecular dynamics parameter file for equilibrating positions: "em.mdp"

```
define                 = -DFLEXIBLE
integrator               = steep
nsteps                 = 2000
nstcomm                  = 100
comm-grps                        =


nstxout                 = 0
nstvout                 = 0
nstfout                = 0
nstlog                 = 1000
nstenergy               = 100
nstxout-compressed       = 1000
compressed-x-precision   = 100
compressed-x-grps        = System
```

```
energygrps              = System

cutoff-scheme           = Verlet
nstlist                 = 10
ns_type                 = grid
pbc                     = xyz
verlet-buffer-tolerance  = 0.005

coulombtype             = cutoff
coulomb-modifier        = Potential-shift-verlet
rcoulomb                = 1.1
epsilon_r               = 15 ; 2.5 (with polarizable water)
vdw_type                = cutoff
vdw-modifier            = Potential-shift-verlet
rvdw                    = 1.1
tcoupl                  = no
pcoupl                  = no
gen_vel                 = no
gen_temp                = 298
gen_seed                = -1

constraints             = none
constraint_algorithm    = Lincs
```

Molecular dynamics parameter file equilibrating under constant NPT conditions "eq.mdp"

```
integrator              = md            ;
dt                      = 0.004         ; ps
nsteps                  = 250000        ; total time: 0.250 ns * 4 = 1.0 ns
tinit                   = 0             ; initial time, ps
```

```
nstcomm                 = 20               ; freq. for cm-motion removal
ld_seed                 = -1

; Bond constraints
constraints             = h-bonds          ; constrain all bonds
constraint_algorithm    = lincs            ; default
lincs_order             = 4                ; default
lincs-iter              = 1                ; accuracy of lincs algorithm

; X/V/F/E outputs
nstxout                 = 500000           ; pos out   ---  1000  ps
nstvout                 = 500000           ; vel out   ---  1000  ps
nstfout                 = 0                ; force out ---  no
nstlog                  = 10000            ; energies to log (20 ps)
nstenergy               = 10000            ; energies to energy file
nstxout-compressed      = 10000            ; xtc, 10 ps
compressed-x-precision  = 1000

; Neighbour list
nstlist                 = 40               ; Freq. to update neighbour list
rlist                   = 0.8              ; nm (cutoff for short-range NL)

; Coulomb interactions
coulombtype             = Reaction-field   ;
epsilon_rf              = 80               ; water
;epsilon_rf             = 4.8              ; CHCl3 (CRC Handbook)
rcoulomb                = 1.4              ; nm (direct space sum cut-off)

; van der Waals interactions
```

```
vdwtype             = Cut-off       ; Van der Waals interactions
rvdw                = 1.4           ; nm (LJ cut-off)
DispCorr            = EnerPres      ; use dispersion correction

; Temperature coupling
Tcoupl              = Berendsen     ; This is ignored with sd integrator
tc-grps             = System        ;
tau_t               = 0.1           ; ps, recommended value for sd
ref_t               = 298.15        ; K

; Energy monitoring
energygrps          = System

; Pressure coupling
Pcoupl              = Berendsen     ;
Pcoupltype          = isotropic     ;
tau_p               = 0.5           ; ps
compressibility     = 5e-5          ; 1/bar
ref_p               = 1.0           ; bar
refcoord_scaling    = all

; Generate velocites in the beginning
continuation        = no            ; continue from npt equilibration
gen_vel             = yes           ; continue from npt equilibration
gen_temp            = 298.0
gen_seed            = -1            ; -1 = the seed is calculated from the process ID number
```

Molecular dynamics parameter file for production run under constant NVT condidtions “run.mdp”

```
integrator          = md
```

```
dt                      = 0.02    ; ps
nsteps                  = 50000000 ; 1 micro second
nstcomm                 = 20
comm-grps               =

nstxout                 = 0
nstvout                 = 0
nstfout                 = 0
nstlog                  = 10000
nstenergy               = 1000
nstxout-compressed      = 10000
compressed-x-precision  = 1000
compressed-x-grps       = System
energygrps              = System

cutoff-scheme           = Verlet
nstlist                 = 40
ns_type                 = grid
pbc                     = xyz
verlet-buffer-tolerance = 0.005

coulombtype             = cutoff
coulomb-modifier        = Potential-shift-verlet
rcoulomb                = 1.1
epsilon_r               = 15 ; 2.5 (with polarizable water)
vdw_type                = cutoff
vdw-modifier            = Potential-shift-verlet
rvdw                    = 1.1
```

```
tcoupl                  = v-rescale
tc-grps                 = System
tau_t                   = 1.0
ref_t                   = 298
Pcoupl                  = parrinello-rahman
Pcoupltype              = isotropic
tau_p                   = 12.0  ;parrinello-rahman is more stable with larger tau-p, DdJ, 20130422
compressibility         = 3e-4
ref_p                   =    1

gen_vel                 = no
gen_temp                = 298
gen_seed                = -1

constraints = h-bonds
constraint_algorithm = lincs
lincs_iter = 2
lincs_order = 4
```
